# SInCRe—structural interactome computational resource for *Mycobacterium tuberculosis*

**DOI:** 10.1093/database/bav060

**Published:** 2015-06-30

**Authors:** Rahul Metri, Sridhar Hariharaputran, Gayatri Ramakrishnan, Praveen Anand, Upadhyayula S. Raghavender, Bernardo Ochoa-Montaño, Alicia P. Higueruelo, Ramanathan Sowdhamini, Nagasuma R. Chandra, Tom L. Blundell, Narayanaswamy Srinivasan

**Affiliations:** ^1^Department of Biochemistry and; ^2^Indian Institute of Science Mathematics Initiative, Indian Institute of Science, Bangalore, India,; ^3^National Centre for Biological Sciences, TIFR, UAS-GKVK Campus, Bellary Road, Bangalore, India,; ^4^Molecular Biophysics Unit, Indian Institute of Science, Bangalore, India, and; ^5^Department of Biochemistry, University of Cambridge, Tennis Court Road, Cambridge, UK

## Abstract

We have developed an integrated database for *Mycobacterium tuberculosis* H37Rv (Mtb) that collates information on protein sequences, domain assignments, functional annotation and 3D structural information along with protein–protein and protein–small molecule interactions. SInCRe (Structural Interactome Computational Resource) is developed out of CamBan (Cambridge and Bangalore) collaboration. The motivation for development of this database is to provide an integrated platform to allow easily access and interpretation of data and results obtained by all the groups in CamBan in the field of Mtb informatics. In-house algorithms and databases developed independently by various academic groups in CamBan are used to generate Mtb-specific datasets and are integrated in this database to provide a structural dimension to studies on tuberculosis. The SInCRe database readily provides information on identification of functional domains, genome-scale modelling of structures of Mtb proteins and characterization of the small-molecule binding sites within Mtb. The resource also provides structure-based function annotation, information on small-molecule binders including FDA (Food and Drug Administration)-approved drugs, protein–protein interactions (PPIs) and natural compounds that bind to pathogen proteins potentially and result in weakening or elimination of host–pathogen protein–protein interactions. Together they provide prerequisites for identification of off-target binding.

**Database URL:** http://proline.biochem.iisc.ernet.in/sincre

## Introduction

*Mycobacterium tuberculosis* H37Rv (Mtb), a causative agent of tuberculosis (TB), has remained a major health concern globally. Based on the World Health Organization (WHO) latest reports, it is estimated that there have been 8.6 million new cases of TB reported in 2012 and a total of 1.3 million TB deaths (1). Most patients are treated for TB using first-line drugs, rifampicin and isoniazid. Together with the other first-line drugs, ethambutol and pyrazinamide, these two drugs form the basic ingredients of combination chemotherapy followed by the WHO directly observed treatment short course strategy (2). Second-line drugs, such as fluoroquinolones, and injectables like kanamycin, capreomycin and amikacin, are relied upon when the first-line drugs fail to control the disease. However in recent times, many antibiotic-resistant strains of *Mtb* have been reported. Multi-drug resistant TB is an Mtb strain resistant to rifampicin and isoniazid. Furthermore, acquisition of resistance towards fluoroquinolone, along with at least one of the injectable drugs, causes extensively drug resistant TB (3). The emergence of resistant strains to the first- and second-line drugs currently used poses a mammoth challenge for control of TB and cure of the infected.

Off-target effects, which are often discovered at the later stages of drug discovery research, have led to failure of many new medicines. Thus, there is an urgent need to discover ways of identifying off-target sites for drugs at an early stage in research. Detailed structural knowledge of the interactions between molecules in the cell provides one way of approaching this problem. The objective would be to define the structural interactome, an inventory of the various interactions between macromolecules and both natural and synthetic small molecules. The structural interactome can augment molecular-level interaction networks and provide a rich source of information on interactions between biological molecules and natural or synthetic ligands. Information on interactions between host and pathogen proteins will be helpful in identifying targets among pathogen proteins.

Integrated databases defining the structural interactome, bringing together information on protein sequences and structures, binding site properties, small molecules and their interactions, provide a valuable resource. Existing integrated databases on TB, TB Database (4, 5) and Tuberculist (6), provide information on genome, proteome, expression as well as corresponding references in the scientific literature but provide no information on structural interactomics comprising of binding sites, small molecules, druggability analysis of targets and functional domain assignments. This work from the CamBan (Cambridge—Bangalore) collaboration, involving four independent research groups from Cambridge and Bangalore, brings together various resources developed by these research groups and elsewhere to provide an extended Mtb structural-interactome resource. Each group has contributed towards the data specific to TB using in-house algorithms and databases developed and established individually over the years. The algorithms used to generate the data are designed to address and enrich sequence and structural data along with various small-molecule interactions. The database also incorporates systems-based analysis and provides list of high-confidence targets.

Sensitive profile-based techniques such as hmmscan of HMMER3.0 (7), Reverse PSI-BLAST (Reverse Position-Specific Iterative Basic Local Alignment Search Tool) (8) and HHblits (9) were used to achieve enhanced domain annotation for the proteins. Structural annotation of *M.* *tuberculosis* proteome (10) and CHOPIN (11) database provided structural data for many proteins. PocketDepth (12), PocketMatch (13) and PocketAlign (14) algorithms are used for binding site prediction and comparison. Protein domain analysis of unannotated genes was pursued using a computationally intensive bioinformatics pipeline called PURE (Prediction of Unassigned Regions) (15). The dataset from CREDO (16), a protein–ligand interaction database for drug discovery, TIMBAL (17), a database of small molecules disrupting protein–protein interactions and TIBLE (http://mordred.bioc.cam.ac.uk/tible/), a database of small molecules against *Mtb* and ligand-based off-target predictions, are connected in structural interactome computational resource (SInCRe). High-confidence drug targets derived from targetTB (18) have been included in the database. Drug targets have also been identified by a sequence-based approach with the help of FDA-approved drugs and are incorporated into the database. An interface has also been provided to integrate the data available from other external resources like STRING (19), STITCH (20) and Tuberculist (6). Future works from these groups will be mapped on to Mtb-specific dataset, and SInCRe will be updated on a regular basis.

## Database

**Figure 1.**
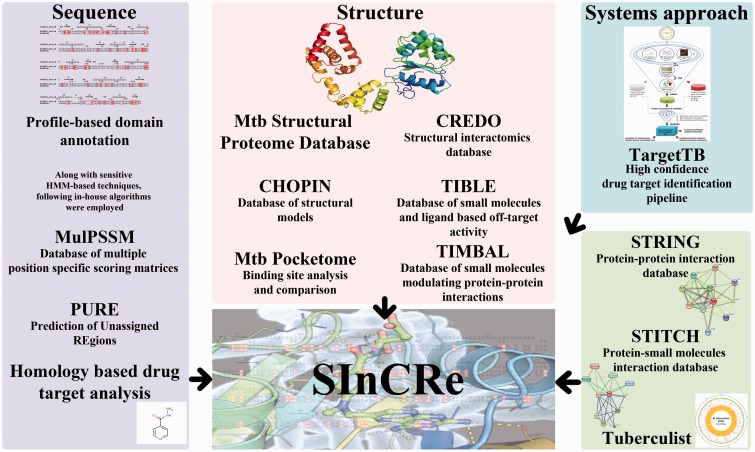
Various data resources contributing to SInCRe.

The integrated suite of databases was developed to provide detailed sequence and structure-based dimensionality to aid in drug-discovery pipelines. The data integrated here were obtained from the databases and webservers developed individually by the four research groups in CamBan. In-house algorithms and databases are the primary resources for the database. Figure 1 details the various data types.

### Sequence-based analysis

#### Algorithms and datasets used.

The development of similarity-search procedures with the use of profiles such as Position Specific Scoring Matrices (PSSMs) (8, 21), Environment-Specific Substitution Tables (22) and Hidden Markov Models (HMMs) (7) has proven to be sensitive in detecting remote homologues reliably. Combination of such sensitive profile-based techniques resulted in the structural and functional annotations for ∼95% of the Mtb proteome. Sensitive approaches such as hmmscan available through HMMER3.0 package, RPS-BLAST and HHblits were employed against sequence and structural profiles of various domain families obtained from Pfam (23), SUPERFAMILY (24), MulPSSM (25, 26) and the HH-suite database (ftp://toolkit.genzentrum.lmu.de/pub/HHsuite/databases/hhsuite_dbs/).

MulPSSM, developed by one of our research groups, is a searchable database of multiple PSSM profiles. The multiple profiles for a given domain family correspond to an alignment, wherein multiple sequences from that family are used as reference. The current version comprises 403 107 profiles for 14 831 Pfam domain families (Pfam v.27) and 14 235 profiles corresponding to 3856 structural families in PALI (Phylogeny and ALIgnment of homologous protein structures) (27) database based on SCOP (Structural Classification Of Proteins) (version 1.75).

#### Assessment of structure and function predictions.

Each of the associations made was assessed based on e-value and alignment length. For associations made using RPS-BLAST against MulPSSM database, an e-value cut-off of 0.01 was used in addition to a profile-coverage threshold of 70%. For domain assignments made using hmmscan against the Pfam database, profile-specific gathering threshold cut-offs were used to extract reliable hits. For hits identified by HHblits, an e-value threshold of 0.001 was used to associate domain families. In the searches against SUPERFAMILY HMM database using hmmscan software, the hits with e-values better than 0.0001 were considered to be reliable and are included in the database. Domain assignments for all the proteins were manually curated to maximize residue (64%) and sequence (89%) coverage.

Structural/functional domains for un-annotated proteins as well as those with unassigned regions were determined with help of the computationally intensive pipeline, PURE (15), developed by one of our groups. Cases where all the earlier approaches were unsuccessful in recognition of structural or functional domains, a fold-recognition algorithm PHYRE2 (28) was employed. A confidence cut-off of 90% was considered to retrieve folds reliably. This exercise was essentially an attempt to assess the foldability of the protein in question. Also, transfer of function based on homology was pursued using HHblits against non-redundant sequence database at an e-value cut-off of 0.001 and query coverage threshold of 60%.

All the hits of the earlier approaches were coupled with manual intervention to ensure maximum residue and sequence coverage of Mtb proteome (29).

#### Drug target identification based on sequence information.

Repurposing drugs has been regarded as a promising strategy mainly due to the reduced cost and time involved. A target identification methodology which essentially integrates homology and pharmacological information (G. Ramakrishnan, N. Chandra and N. Srinivasan, in preparation) facilitated recognition of 132 FDA-approved drugs which could be repurposed for 56 potential targets in *Mtb.* This methodology comprises three steps: exploration of evolutionary relationship between targets of known FDA-approved drugs and *Mtb* proteins, structural elucidation of binding sites of *Mtb* proteins homologous to known targets and evaluation of predicted binding sites with the help of protein–ligand docking. Evolutionary relationships were explored with the help of a sensitive profile-based iterative search tool, jackhmmer(30), at an e-value threshold of 0.0001. An initial filtering step to eliminate drugs known to act on human proteins ensured that the ‘anti-targets’ in host are not picked up. The reliably identified relationships picked were further probed for the conservation of ligand-binding site residues across known targets and the *Mtb* proteins homologous to these targets. Structural information was taken from Protein Data Bank (PDB) (31) for *Mtb* and high-confidence structural models obtained from ModBase (32) for proteins with no known structure were used to assess the binding pockets. A structural alignment algorithm, TM-align (33), was effective in identification of highly similar local structural matches (TM-score > 0.50) between targets and their corresponding homologues in *Mtb.* Finally, the shortlisted proteins in *Mtb* predicted to serve as potential targets were evaluated using FDA-approved drugs with the help of an efficient protein–ligand docking tool, Glide (34–36) (http://www.schrodinger.com/Glide). A total of 132 FDA-approved drugs (Supplementary Table S1) were thus identified, which could be repurposed for 56 potential target proteins in *Mtb.*

### Structure-based analysis

#### Structural proteome of Mtb.

Structural annotation of the *M.* *tuberculosis* proteome was carried out by one of the groups (10). PDB holds a total of 324 crystal structures of Mtb proteins and comparative models were generated for 2737 proteins, thus giving structure availability for 70% of the Mtb proteome. Structural models were generated using Modpipe, a software suite along with ModBase (32), a database of models generated using comparative modelling. The structural models need to be of high confidence and reliability as they play a central role to all the further analysis carried out. To assess the reliability of the protein structural models, various structure verification methods including statistical scoring potential (37, 38), secondary structure compatibility (39) and stereochemical quality check (40) were used. In the case of multi-domain proteins 3D models of individual domains are presented. Only those binding sites that were detected within the domains are analysed.

The CHOPIN (11) database (http://structure.bioc.cam.ac.uk/chopin) assigns structural domains and generates homology models for 2911 sequences, corresponding to ∼73% of the proteome. Conformational states, characteristic of different oligomeric states and ligand binding, reflect various functional states of the proteins. Additionally, CHOPIN includes structural analyses of mutations potentially associated with drug resistance. The model number, sequence coverage and zscore are displayed on the SInCRe result page with links provided to CHOPIN webpage (http://mordred.bioc.cam.ac.uk/chopin/about) that provides model details and an option to download the models.

#### Detection of binding sites.

Computational methods for binding site detection can be classified into three broad categories based on their approaches: (i) evolutionary methods based on structure–sequence alignment (ii) energy-based methods using chemical probes and (iii) geometric approaches that scan the 3D structure of the protein to detect pockets. Each of these methods has its own strengths and limitations with respect to different aspects such as accuracy in detection and prediction, computational time, complexity and features captured. All the three methods were used in this study to minimize the prediction error and increase the confidence. The methods used are, a grid-based geometric method, PocketDepth (12), evolutionary method, Ligsite (41) and energy-based method, SiteHound. PocketDepth is an in-house method that uses depth-based clustering algorithm for detecting putative binding sites in the given protein structures. The idea that depth is defined by the centrality of empty subspaces in a protein structure is used to identify the pockets from all the protein structures. The PocketDepth algorithm was later combined with LIGSITEcsc, which uses Connolly’s surface (42) to identify surface–solvent–surface events that involves grooves and then detects binding sites in a given protein by mapping the degree of conservation of the residues in the selected surface. All the pockets detected by PocketDepth that are within 5 Å radius of the predicted LIGSITEcsc pockets were selected. SiteHound (43), an energy method that searches for interaction zones favourable for a methyl probe within the protein, was used on all the pockets identified as a filter to fetch out final set of consensus ligand binding sites.

Other than the binding sites identified by these methods, pockets were also selected based on the experimentally characterized binding site residues in each protein in the proteome or in their homologues. This was done by fetching entries from the database using respective general feature format files obtained from UniProt database (44). Possible binding sites were identified by scanning each protein sequence in the proteome with known binding motifs from the Prosite (45) database to make sure they were not missed out by other methods in the workflow (46). The binding sites detected can be viewed using Jmol plugin and also co-ordinates of these binding pockets can be downloaded in pdb format.

#### Drug binding site database and comparison.

DrugBank (47) and DrugPort were used to prepare a combined list of drugs or drug-like compounds; these included approved and experimental drugs and nutraceuticals. XML data files were obtained from these two databases and later parsed to extract information on proteins complexed with any of these drugs present in PDB. The binding sites were then extracted from these complexes. Residues of all atoms that lie within 4.5Å of any atom in the drug molecule were extracted as part of the binding site. Ten thousand six hundred and fifty-eight (from Drugbank) (Supplementary Table S2) + 2516 (from Drugport) (Supplementary Table S3) drug-binding sites were obtained from PDB through this process. High-confidence targets from Mtb were scanned using these known drug-binding sites, and also drug-binding sites were scanned for similarities against different binding site clusters.

#### Structural interactome.

The structural interactomics database CREDO (16) provides details of pairwise atomic interactions of intermolecular and intramolecular contacts between ligands and macromolecule for the structures in PDB. The PDB codes in the database are linked to the results of CREDO. This database stores interaction between atoms as structural interaction fingerprints as implemented by Deng *et al*. (48). Thirteen different interaction types such as hydrogen bonds, halogen bonds, carbonyl interactions and more are currently implemented in CREDO. Polypeptide-residue mapping is done onto UniProt. This allows identification of modified, non-standard or mutated proteins in the PDB compared with sequence in UniProt. Further, small-molecule and protein interaction details are provided in the database. Physico-chemical properties are calculated for all the small molecules in PDB and these properties are important for evaluating its drug-likeness. Topological similarities of the small molecules based on 2D and 3D descriptors are also retrieved from the database. With these data, CREDO provides major structural interaction details to study small-molecule binding properties. The PDB structures used as templates for building models in SInCRe are linked to the CREDO database.

#### Structure binding molecules.

TIMBAL (17), a database of small molecules disrupting protein–protein interactions, provides us with a list of small molecules relevant to the proteins of *Mtb*. Previously constructed by manual curation, now TIMBAL is automated to identify a list of protein–protein interaction modulators. The PPI targets and their orthologs are identified by UniProt identifiers. Small molecules related to these proteins are searched using UniProt identifiers in ChEMBL database. The homologues of known protein–protein interactions to the proteins in Mtb are identified and corresponding small molecules are listed. Totally 21 Mtb proteins are homologous to proteins in TIMBAL database corresponding to 11 targets.

#### Ligand-based off-target prediction and small-molecule data.

There are two main approaches to predict off-target activity. The structure-based approach relies on the similarity of the targets binding pockets, whereas the ligand-based approach connects targets based on the similarity of their ligands. The two methodologies complement each other (49). TIBLE (http://mordred.bioc.cam.ac.uk/tible/) collects small-molecule data (Minimal Inhibitory Concentration (MIC) for mycobacterium and binding to isolated Mtb targets) from the ChEMBL database (50) and the CDD (51). There are 75 Mtb targets with small-molecule binding data. For each of these targets, three independent algorithms—SEA (52), PharmMapper (53) and PASS (54) are used to derive off-target ligand-based predictions. Link from TIBLE to PharmMapper offers pharmacophore-matching platform for potential target identification. The details of small molecules and ligand-based off-target are integrated into the SInCRe database and also linked to the TIBLE page for detailed information.

### Systems-based target identification

Identification of high confidence drug targets is a primary factor for efficient drug treatment. TargetTB (18), a comprehensive *in silico* target identification pipeline, was developed by one of the groups. The pipeline is built by incorporating network-based analysis of the protein–protein interactions, a flux-balance analysis of the reactome, phenotype-essentiality data derived from experiments, targetability assessment based on sequence and structure analysis using in-house novel algorithms. Initially proteins that are important for the survival of *Mtb* were identified using flux balance and network analyses. Subsequently comparative genomics with the host was carried out. Finally the viability of a protein to be a potential drug target was assessed using novel methods for structural analysis of binding sites. Further, expression-data analysis, providing correlation and non-similarity measures of target proteins to gut flora proteins and also to ‘anti-target’ proteins in the host, was analysed extensively. Four hundred and fifty-one high-confidence entries were identified by this analysis pipeline. These short-listed targets have been further analysed through phylogenetic profiling against 228 pathogen genomes to identify antibiotic targets of broad spectrum especially those specific to TB. Target proteins significant to mycobacterial persistence and drug resistance mechanisms have also been analysed and reported. The details of the targets identified through TargetTB pipeline has been integrated into this database.

### Other resources

External data from STRING (19), a database of known and predicted protein–protein interactions, STITCH (20), a database of protein–small molecule interactions and Tuberculist (6) for primary details about each Mtb protein are integrated into the SInCRe database.

## Coverage of the *M. tuberculosis* proteome in the database

**Figure 2.**
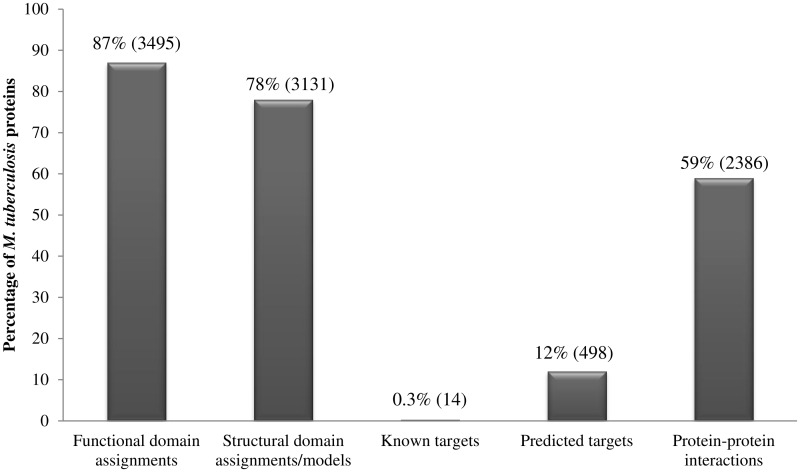
Percentage coverage of *M. tuberculosis* proteins in the database. Numbers in brackets denote absolute values.

Our analysis of the repertoire of *M. tuberculosis* proteins, using a multitude of sensitive techniques, has generated a resource of information including structural and functional domain assignments, potential drug-targets and small-molecule binders including FDA-approved drugs. Figure 2 summarizes the percentage coverage achieved for *M. tuberculosis* proteins and indicates that 3495 of 4018 proteins could be associated with at least one functional domain (Pfam domain) assignment while 3131 proteins could either be associated with structural domains (SCOP domains) or with proteins of known structure. In terms of domain assignment alone, a total of 3566 proteins (89%) could be associated with at least one structural or functional domain. Due to the combined use of sensitive profile-based techniques, the percentage of *M. tuberculosis* proteins associated with functional domains is 3% higher than the annotations available in databases such as Pfam; and the percentage coverage achieved in terms of structural domains is 8% higher than the structural annotations available in databases such as SUPERFAMILY.

Systematic means to identify potential drug targets in *M. tuberculosis* has resulted in recognition of 498 high-confidence targets, constituting 12% of the proteome. The SInCRe database also includes information on protein–protein interactions within *M. tuberculosis* as documented in resources such as STRING. Approximately 23 000 known or predicted protein–protein interactions in *M. tuberculosis* are mediated by 2386 (59%) proteins.

Our attempt to integrate information from diverse resources provides a unified platform to explore and investigate the usefulness of a predicted target or a small molecule in the context of drug development and drug discovery for TB.

## Database and web interface

The SInCRe database is created by integrating resources from various other databases for 4018 Mtb proteins. This database has been developed on the Linux-Apache-MySQL-PHP platform. Sequence- and structure-level datasets have been stored in efficiently designed relational database schema. The web interface is developed using BootStrap (http://twitter.github.com/bootstrap). This provides cascading style sheets framework and javascript functionality. CytoscapeWeb (55), a java plugin, is used for interactive display of protein–protein and protein–small molecules interaction networks. Protein structures are represented in 3D using JSmol, a JavaScript-based molecular viewer from Jmol, an open-source Java viewer for chemical structures in 3D (http://www.jmol.org/). The modelled structures and sequences can be downloaded in PDB and FASTA formats, respectively. The tables in webpages are sortable and searchable, giving the user ease of acquiring data of interest.

The database can be queried using Rv IDs, gene name, UniProt ID, Pfam ID and Tuberculist functional classification. The dataset can be browsed for information available based on a few methods for limited list of Rv IDs.

## Prediction of drug interactions using SInCRe

Protein kinases and phosphatases constitute important classes of drug targets due to the critical roles played by them in reversible protein phosphorylation that regulates many biological processes. There are many studies that report the development of potent inhibitors for these enzymes involved in protein phosphorylation to treat different types of cancer and autoimmune diseases (56). Serine/threonine protein kinases (STPKs) are one such class of kinases that specifically phosphorylate the hydroxyl group of one or more serine and threonine residues in the substrate protein. *Mycobacterium tuberculosis* (Mtb) genome houses 11 of such STPK genes and all of these are known to regulate crucial signalling processes, playing an important role in regulating physiology and virulence of the pathogen (57).

Of the 11 STPKs in Mtb, nine (PknA, PknB, PknD, PknE, PknF, PknH, PknI, PknJ and PknL) are receptors containing a transmembrane helix with extracellular sensory domain and intracellular kinase domain, thus acting as signal transducers. The other two kinases (PknG and PknK) are cytoplasmic containing a regulatory domain and could hence play a role in intracellular responses. Here, we explore the role of one such STPK – PknD (Rv0931c), as a putative drug target through the information present in SInCRe database. PknD acts a receptor kinase with extracellular sensory domain adopting a six-bladed β propeller structure (PDB ID: 1RWL, 1RWI) (58), and an intracellular kinase domain. The 3D structure of intracellular kinase domain could be derived using homology modelling using the crystal structure of PknE (PDB ID: 2H34) kinase domain as the template which share 59.7% sequence identity with the target. Although the substrate and the ligand for the PknD is yet to be discovered, the gene neighbourhood analysis reveals that it could play an essential role in phosphate transport. This is complemented by the fact that the growth of Δ*pknD* strain is compromised in a phosphate deficient medium (59). Recently, PknD has been observed to phosphorylate the N-terminal domain of Rv0516c, a putative regulator of sigma factor SigF (60). These three genes—PknD, Rv0516c and SigF play an important role in osmosensory signalling pathway (61). Moreover, a screen for identifying important genes for central nervous system infection by Mtb also identified PknD to be essential as Δ*pknD* strain was observed to be defective for invasion of central nervous system (62).

**Figure 3.**
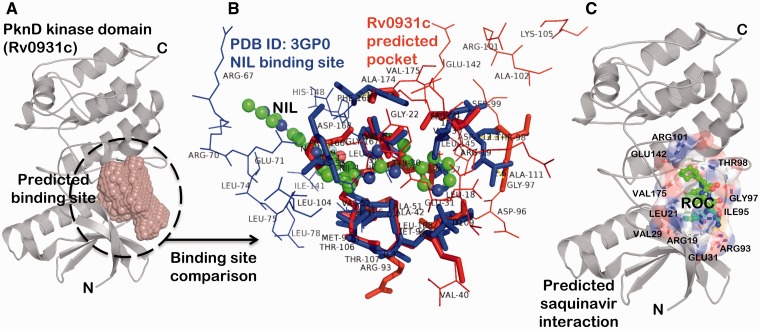
Example for predicted drug interactions using SInCRe. (**A**) A predicted binding site for PknD, a STPK, is depicted in the form of spacefill. (**B**) The alignment of predicted binding site from PknD (Rv0931c, in red) with the NIL binding site from Human Mitogen Activated Protein Kinase (PDB ID: 3GP0). The corresponding residues are highlighted in sticks, whereas unique residues with no correspondences are represented as wireframe. These distinguishing residues can be targeted to achieve the selectivity. (**C**) The best pose derived from computational docking depicting the interaction of saquinavir (ROC, shown as green ball and stick model) with the residues (represented as sticks) of the predicted binding site in PknD.

The binding site prediction exercise carried out on a proteome-scale involving a consensus of different types of algorithm (46) identified a putative binding site present at the interface of N-terminal and C-terminal lobe of kinase domain in PknD (Figure 3A). A systematic binding site comparison of this predicted pocket against a database of approved drug-binding sites yielded nilotinib (NIL) binding site from human mitogen activated protein kinase 11 protein (PDB ID: 3GP0) as the topmost hit with binding site similarity score (PMAX) (13) of 0.703. A binding site alignment of the predicted pocket with this known NIL binding site using PocketAlign algorithm (14) reveals the observed similarity and the differences in the binding sites (Figure 3B). Although the similarity of these protein kinases with the human counterparts can increase the risk of toxicity, there are supporting evidences in the literature that have successfully exploited the ATP-binding sites to achieve the selectivity. There are FDA-approved drugs that selectively bind to active and inactive conformations of the protein kinases to achieve the selectivity (56). The differences in kinase inhibitor binding sites (depicted as wireframe in Figure 3B) could be used as anchor points in fragment-based drug discovery to achieve the selectivity towards Mtb protein kinases. Interestingly, the binding sites of many of the anti-retroviral protease inhibitors like nelfinavir and lopinavir were also observed to have similarity to the predicted binding site in PknD. These observations are supported by the fact that nelfinavir is found to have anti-cancerous property attributed to its ability to weakly inhibit multiple protein kinases (63). One such anti-retroviral protease inhibitor—saquinavir (Ligand code: ROC), having high binding site similarity with the predicted binding site in PknD was explored further through computational docking using AutoDock Vina (Figure 3C) (64). The computationally predicted binding affinity (−8.1 kcal/mol) was found to be comparable to the native saquinavir complexed with HIV-protease (−9.4 kcal/mol). The best pose obtained through computational docking predicted the residues—ARG101, GLU142, ARG93 and GLU31 present in the predicted binding site to have crucial interaction with the saquinavir. These interesting drug associations can be readily obtained from the ‘protein–small molecule associations’ tab presented in the SInCRe database. The SInCRe database can thus, be used to generate readily testable hypothesis for anti-tubercular drug discovery.

## Conclusion

SInCRe is an integrated suite of databases that provides the outcome of extensive sequence and structural studies of Mtb proteins. Sequence-based domain assignment and structural analysis of binding sites act as a resource to help in the identification off-target interactions of drug molecules, knowledge of which is useful in the design of novel drugs for *M. tuberculosis.* Future updates will include incorporation of other resources from Cambridge and Bangalore.

## Supplementary Data

Supplementary data are available at *Database* Online.

Supplementary Data
