# Supplementary material for: SInCRe—structural interactome computational resource for Mycobacterium tuberculosis
Source: Database (Oxford). 2015 Jun 30;2015:bav060. doi: 10.1093/database/bav060 (PMC4485431; doi:10.1093/database/bav060)
Supplement: Supplementary Data [file supp_2015_bav060_index.html]

SInCRe—structural interactome computational resource for Mycobacterium tuberculosis — Supplementary Data 

# SInCRe—structural interactome computational resource for *Mycobacterium tuberculosis*

## Supplementary Data

files

- Supplementary Data - zip file
