# Supplementary material for: SInCRe—structural interactome computational resource for Mycobacterium tuberculosis
Source: Database (Oxford). 2015 Jun 30;2015:bav060. doi: 10.1093/database/bav060 (PMC4485431; doi:10.1093/database/bav060)
Supplement: Supplementary Data [file supp_bav060_suppl_data.zip › SInCRe_supplementary_Table2.docx]

Supplementary Table 2: List of 10658 drugs (DrugBank) binding sites obtained from PDB.

| PDB_ID | LIG_ID | CHAINID | RESNO |
| --- | --- | --- | --- |
| 11ba | UPA | A | 125 |
| 11bg | U2G | A | 130 |
| 13gs | SAS | A | 210 |
| 182l | BZF | A | 401 |
| 183l | DEN | A | 400 |
| 184l | I4B | A | 401 |
| 185l | IND | A | 400 |
| 186l | N4B | A | 400 |
| 187l | PXY | A | 400 |
| 188l | OXE | A | 400 |
| 1a05 | IPM | A | 401 |
| 1a0i | ATP | A | 1 |
| 1a0q | HEP | H | 214 |
| 1a0r | FAR | G | 72 |
| 1a0t | SUC | P | 1 |
| 1a25 | PSE | B | 1 |
| 1a26 | CNA | A | 200 |
| 1a3w | FBP | A | 1007 |
| 1a42 | BZU | A | 555 |
| 1a49 | ATP | A | 535 |
| 1a4i | NDP | A | 302 |
| 1a4k | FRA | B | 3083 |
| 1a4r | GNH | B | 400 |
| 1a54 | MDC | A | 323 |
| 1a5z | FBP | A | 353 |
| 1a5z | FBP | A | 354 |
| 1a65 | PYE | A | 900 |
| 1a6v | NPC | H | 430 |
| 1a72 | PAD | A | 378 |
| 1a78 | TDG | A | 135 |
| 1a7a | ADC | A | 435 |
| 1a82 | ATP | A | 802 |
| 1a8a | GSE | A | 997 |
| 1a8b | GPE | A | 999 |
| 1a8i | GLS | A | 998 |
| 1a8j | PME | H | 217 |
| 1a8r | GTP | A | 401 |
| 1a8s | PPI | A | 278 |
| 1a95 | GUN | B | 304 |
| 1a95 | PCP | B | 301 |
| 1a96 | XAN | B | 304 |
| 1a9p | 9DI | A | 290 |
| 1a9t | R1P | A | 291 |
| 1aa1 | 3PG | B | 477 |
| 1aax | BPM | A | 323 |
| 1ab8 | FOK | A | 1 |
| 1abr | MAN | B | 276 |
| 1abr | NDG | B | 268 |
| 1abr | NDG | B | 273 |
| 1aco | TRA | A | 755 |
| 1add | 1DA | A | 353 |
| 1adl | ACD | A | 135 |
| 1adl | PPI | A | 136 |
| 1ae8 | AZL | H | 600 |
| 1aee | ANL | A | 296 |
| 1aet | 1MZ | A | 296 |
| 1af6 | FRU | A | 423 |
| 1af7 | SAH | A | 287 |
| 1afa | MBG | 1 | 1 |
| 1afk | PAP | A | 125 |
| 1afl | ATR | A | 125 |
| 1afl | CIT | A | 999 |
| 1agw | SU2 | A | 1001 |
| 1ahf | IOP | A | 411 |
| 1ahp | MAL | A1006 |  |
| 1ahv | NCR | A | 601 |
| 1ahz | EPT | A | 602 |
| 1ai2 | ICA | A | 418 |
| 1ai4 | DHY | B | 559 |
| 1aj0 | SAN | A | 561 |
| 1aj8 | CIT | A | 1000 |
| 1ajn | AAN | B | 559 |
| 1ajp | OMD | A | 210 |
| 1ajx | AH1 | A | 500 |
| 1akc | PPE | A | 411 |
| 1al2 | MYR | 4 | 1 |
| 1al2 | SPH | 1 | 0 |
| 1al7 | HST | A | 361 |
| 1al8 | DHP | A | 361 |
| 1aln | CTD | A | 295 |
| 1alw | ISA | A | 11 |
| 1am6 | HAE | A | 555 |
| 1ami | MIC | A | 755 |
| 1amz | MLT | A | 702 |
| 1ank | ANP | B | 216 |
| 1ao0 | ADP | A | 468 |
| 1aob | DDU | A | 301 |
| 1aoe | GW3 | A | 194 |
| 1aog | MAE | A | 500 |
| 1aph | DCE | B | 200 |
| 1apv | DMF | I | 587 |
| 1aqf | PEQ | A | 532 |
| 1aqi | SAH | A | 500 |
| 1aqj | SFG | A | 500 |
| 1aql | TCH | A | 601 |
| 1ar6 | MYR | 4 | 1 |
| 1ar6 | SPH | 1 | 0 |
| 1ar7 | MYR | 4 | 1 |
| 1ar7 | SPH | 1 | 0 |
| 1ar8 | MYR | 4 | 1 |
| 1ar8 | SPH | 1 | 0 |
| 1ar9 | MYR | 4 | 1 |
| 1ar9 | SPH | 1 | 0 |
| 1as0 | GSP | A | 355 |
| 1asj | MYR | 4 | 1 |
| 1asj | SPH | 1 | 0 |
| 1atn | NDG | D | 261 |
| 1auk | NDG | A | 601 |
| 1aux | SAP | A | 800 |
| 1ax0 | A2G | A | 401 |
| 1axw | MTX | A | 732 |
| 1ayp | INB | A | 201 |
| 1az8 | IN4 | A | 1 |
| 1b0d | TSU | A | 130 |
| 1b0u | ATP | A | 301 |
| 1b2l | CYH | A | 256 |
| 1b2l | NDC | A | 255 |
| 1b3d | S27 | B | 401 |
| 1b3o | SAE | A | 600 |
| 1b42 | M1A | A | 600 |
| 1b48 | HAG | A | 223 |
| 1b4d | CRA | A | 998 |
| 1b4n | GUA | A | 622 |
| 1b4p | GPS | A | 218 |
| 1b4u | DHB | B | 503 |
| 1b5e | DCM | A | 300 |
| 1b5q | MD2 | A | 590 |
| 1b66 | BIO | A | 700 |
| 1b74 | DGN | A | 538 |
| 1b7e | TPT | A | 480 |
| 1b8a | ATP | A | 500 |
| 1b8n | IMG | A | 600 |
| 1b8u | OAA | A | 350 |
| 1b8y | IN7 | A | 502 |
| 1b99 | FUP | A | 160 |
| 1b9s | FDI | A | 468 |
| 1b9v | RA2 | A | 468 |
| 1ba3 | MBR | A | 990 |
| 1bb5 | CTO | A | 800 |
| 1bb6 | UMG | A | 130 |
| 1bb7 | GUM | A | 130 |
| 1bbp | BLV | A | 500 |
| 1bcc | PEE | E | 198 |
| 1bcp | ATP | E | 111 |
| 1bd0 | IN5 | A | 389 |
| 1bd4 | URA | A | 999 |
| 1bdo | BTN | A | 157 |
| 1bdu | DUR | A | 301 |
| 1be4 | PCG | A | 160 |
| 1bep | CCH | A | 296 |
| 1bev | MYR | 1 | 900 |
| 1bfb | IDS | A | 303 |
| 1bfb | SGN | A | 302 |
| 1bg0 | DAR | A | 403 |
| 1bg9 | AF1 | A | 803 |
| 1bgg | GCO | B | 500 |
| 1bh5 | GTX | A | 200 |
| 1bh9 | PMB | B | 300 |
| 1bhg | BMA | A | 653 |
| 1bhg | MAN | A | 655 |
| 1biw | S80 | B | 401 |
| 1bj9 | DDH | A | 296 |
| 1bjg | TMF | A | 267 |
| 1bju | GP6 | A | 910 |
| 1bjv | GP8 | A | 900 |
| 1bk0 | ACV | A | 351 |
| 1bkf | FK5 | A | 108 |
| 1bky | 1MC | A | 600 |
| 1bl4 | AP1 | A | 108 |
| 1bls | IPP | A | 362 |
| 1bnw | TPD | A | 555 |
| 1bo4 | SPD | A | 200 |
| 1bo5 | FBP | O | 502 |
| 1bpe | DTP | A | 338 |
| 1bph | DCE | B | 200 |
| 1bq4 | BHC | A | 302 |
| 1br5 | NEO | A | 500 |
| 1brw | URA | B | 5001 |
| 1bsj | MLN | A | 171 |
| 1btu | 2BL | A | 7 |
| 1bug | URS | A | 1002 |
| 1bux | PPS | A | 160 |
| 1bvd | BLA | A | 154 |
| 1bvr | THT | A | 1302 |
| 1bw9 | PPY | A | 361 |
| 1bwb | 146 | B | 641 |
| 1bwc | AJ3 | A | 4058 |
| 1bxg | HCI | A | 361 |
| 1bxo | MAN | A | 328 |
| 1bxr | ANP | A | 1084 |
| 1by5 | OES | A | 715 |
| 1byd | DOM | A | 496 |
| 1bye | ATA | A | 1001 |
| 1byk | T6P | A | 317 |
| 1byq | ADP | A | 2001 |
| 1bzc | TPI | A | 902 |
| 1bzl | GCG | A | 603 |
| 1c0l | FLA | A | 1364 |
| 1c0p | FAD | A | 1363 |
| 1c0t | BM1 | A | 999 |
| 1c0u | BM5 | A | 999 |
| 1c12 | TRZ | A | 215 |
| 1c1b | GCA | A | 999 |
| 1c1c | 612 | A | 999 |
| 1c1h | MMP | A | 800 |
| 1c1x | HFA | A | 361 |
| 1c22 | MF3 | A | 300 |
| 1c23 | MPH | A | 300 |
| 1c24 | MPJ | A | 300 |
| 1c2k | ABI | A | 246 |
| 1c2t | NHS | A | 222 |
| 1c39 | P3M | A | 1200 |
| 1c3e | NHR | A | 220 |
| 1c3i | TR1 | B | 1 |
| 1c3q | TZE | A | 301 |
| 1c3x | 8IG | A | 306 |
| 1c4g | VG1 | A | 563 |
| 1c4k | GTP | A | 999 |
| 1c5q | ESI | A | 246 |
| 1c5s | ESX | A | 246 |
| 1c5t | ESP | A | 246 |
| 1c70 | L75 | B | 423 |
| 1c72 | EPY | A | 220 |
| 1c7s | CBS | A | 1001 |
| 1c7z | G3H | A | 501 |
| 1c80 | GTP | A | 356 |
| 1c81 | FDQ | A | 441 |
| 1c83 | OAI | A | 301 |
| 1c84 | 761 | A | 301 |
| 1c85 | OBA | A | 301 |
| 1c87 | OPA | A | 301 |
| 1c88 | OTA | A | 301 |
| 1c8k | CPB | A | 940 |
| 1c8v | HE1 | A | 270 |
| 1c9c | PP3 | A | 413 |
| 1c9d | HF1 | A | 270 |
| 1c9e | MP1 | A | 800 |
| 1c9k | 5GP | A | 604 |
| 1cb8 | MAN | A | 2000 |
| 1cbk | ROI | A | 601 |
| 1cbq | RE9 | A | 200 |
| 1cbx | BZS | A | 500 |
| 1ccw | CNC | A | 800 |
| 1ccw | TAR | B | 900 |
| 1ce8 | IMP | A | 5012 |
| 1ceb | AMH | A | 90 |
| 1cel | IBZ | A | 436 |
| 1cf8 | HAZ | H | 800 |
| 1cfv | E3G | H | 200 |
| 1cgz | STL | A | 390 |
| 1ch8 | GPX | A | 432 |
| 1chw | HXC | A | 390 |
| 1cin | MTS | A | 264 |
| 1ciz | DPS | A | 307 |
| 1cjp | MUG | A | 240 |
| 1cjt | DAD | A | 102 |
| 1ck6 | BMA | A | 350 |
| 1ckm | GTP | A | 899 |
| 1ckm | GTP | B | 999 |
| 1cko | GP3 | A | 999 |
| 1cl2 | PPG | A | 500 |
| 1cmc | SAM | A | 105 |
| 1cmp | DMI | A | 295 |
| 1cnf | ADP | A | 272 |
| 1cnx | EG2 | A | 555 |
| 1cny | EG3 | A | 555 |
| 1cov | MYR | 4 | 1 |
| 1cov | PLM | 1 | 282 |
| 1coy | AND | A | 508 |
| 1coz | CTP | A | 130 |
| 1cph | DCE | B | 200 |
| 1cpo | XYS | A | 748 |
| 1cq1 | BGC | A | 455 |
| 1cq7 | PY5 | A | 413 |
| 1cq8 | PY6 | A | 413 |
| 1cqi | ADP | B | 801 |
| 1cqp | 803 | A | 311 |
| 1cqx | DGG | A | 406 |
| 1cqx | FAD | A | 405 |
| 1cr1 | TTP | A | 600 |
| 1cr2 | DTP | A | 600 |
| 1cr4 | TYD | A | 600 |
| 1cr6 | CPU | A | 1100 |
| 1cra | TRI | A | 494 |
| 1cru | HDN | A | 3002 |
| 1cru | PQQ | A | 1905 |
| 1cs4 | 101 | B | 1082 |
| 1csh | OAA | A | 702 |
| 1css | FCX | A | 700 |
| 1cte | PYS | A | 255 |
| 1cul | 103 | B | 1082 |
| 1cvu | ACD | A | 701 |
| 1cw2 | HSP | A | 270 |
| 1cw4 | AKG | A | 417 |
| 1cwv | CIT | A | 994 |
| 1cxf | ACX | A | 688 |
| 1cxf | ACX | A | 689 |
| 1cxl | G4D | A | 692 |
| 1cxl | G4D | A | 694 |
| 1cxv | CBP | A | 10 |
| 1cy1 | TMP | A | 600 |
| 1cz9 | CIT | A | 399 |
| 1cza | ADP | N | 922 |
| 1cza | G6P | N | 919 |
| 1czc | GUA | A | 414 |
| 1cze | SIN | A | 414 |
| 1czq | DTR | D | 10 |
| 1czs | PHG | A | 296 |
| 1d0c | HEM | A | 500 |
| 1d0c | INE | A | 760 |
| 1d0h | A2G | A | 601 |
| 1d0k | DGL | A | 404 |
| 1d0l | BLG | A | 401 |
| 1d0m | NDG | A | 406 |
| 1d0s | DMD | A | 999 |
| 1d1c | NMQ | A | 999 |
| 1d1q | 4NP | A | 401 |
| 1d1w | ATQ | A | 805 |
| 1d1x | 4BT | A | 830 |
| 1d1y | 3BT | A | 820 |
| 1d2a | ADE | B | 401 |
| 1d2h | SAH | A | 1301 |
| 1d2n | ANP | A | 1 |
| 1d2s | DHT | A | 301 |
| 1d3b | CIT | B | 702 |
| 1d3g | DDQ | A | 700 |
| 1d3g | ORO | A | 399 |
| 1d3h | A26 | A | 397 |
| 1d4d | SIN | A | 700 |
| 1d4i | BEG | A | 501 |
| 1d4m | MYR | 4 | 1 |
| 1d4m | W71 | 1 | 501 |
| 1d5j | MM3 | A | 401 |
| 1d6f | B3P | A | 391 |
| 1d6n | PPO | A | 300 |
| 1d6z | HY1 | A | 2001 |
| 1d6z | PEA | A | 2003 |
| 1d7b | HEM | A | 401 |
| 1d7i | DSS | A | 301 |
| 1d7j | BUQ | A | 311 |
| 1d7l | RFL | A | 395 |
| 1d7r | 5PA | A | 500 |
| 1d7s | DCS | A | 500 |
| 1d7u | LCS | A | 500 |
| 1d7v | NMA | A | 500 |
| 1d7x | SPC | A | 401 |
| 1d8c | GLV | A | 2000 |
| 1d8c | SOR | A | 4000 |
| 1d8f | SPI | B | 901 |
| 1d8m | BBH | B | 901 |
| 1d9u | NDG | B | 1404 |
| 1dae | IKT | A | 225 |
| 1dag | ACP | A | 226 |
| 1dag | DSD | A | 225 |
| 1dah | DNN | A | 225 |
| 1dak | DPU | A | 801 |
| 1dam | DTB | A | 803 |
| 1dao | FAB | A | 348 |
| 1db4 | 8IN | A | 200 |
| 1db5 | 6IN | A | 200 |
| 1dbg | MAN | A | 507 |
| 1dbj | AE2 | H | 229 |
| 1dbk | ANO | H | 229 |
| 1dbm | SIH | H | 229 |
| 1dbn | SIA | A | 822 |
| 1dbo | GC4 | A | 516 |
| 1dcp | HBI | A | 105 |
| 1dcy | I3N | A1201 |  |
| 1dd6 | MCI | A | 550 |
| 1ddo | DTR | E | 350 |
| 1ddo | ITR | A | 349 |
| 1ddt | APU | A | 950 |
| 1ddx | PGX | A | 701 |
| 1de5 | RNT | A1460 |  |
| 1de6 | RNS | A1462 |  |
| 1dek | DGP | A | 301 |
| 1df7 | MTX | A | 501 |
| 1dfg | NDT | A | 0 |
| 1dfh | TDB | A | 0 |
| 1dfo | FFO | A1002 |  |
| 1dg7 | WRB | A | 200 |
| 1dgf | NDP | A4000 |  |
| 1dgp | FOH | A | 0 |
| 1di8 | DTQ | A | 500 |
| 1di9 | MSQ | A | 500 |
| 1dia | L24 | A3001 |  |
| 1did | DIG | A | 400 |
| 1dig | L37 | A3001 |  |
| 1dil | AXP | A | 384 |
| 1diq | PCR | A | 798 |
| 1dj9 | KAM | A | 400 |
| 1djx | I3P | A | 1 |
| 1djy | I2P | A | 1 |
| 1dkf | OLA | A | 700 |
| 1dkt | V7O | A | 100 |
| 1dku | ABM | A1003 |  |
| 1dli | UDX | A | 404 |
| 1dlj | UGA | A | 404 |
| 1dlk | PHQ | E | 1 |
| 1dm2 | HMD | A | 400 |
| 1dm6 | PH3 | A1780 |  |
| 1dm8 | TZC | A1790 |  |
| 1dmg | CIT | A | 510 |
| 1dmh | MCT | A | 401 |
| 1dmi | BHS | A1610 |  |
| 1dmj | AP4 | A1650 |  |
| 1dmk | AP6 | A1660 |  |
| 1dmk | ITU | A1800 |  |
| 1dmt | RDF | A | 750 |
| 1doa | GER | A | 952 |
| 1dod | DOB | A | 396 |
| 1dph | DCE | B | 200 |
| 1dq9 | HMG | A | 101 |
| 1dqa | MAH | A | 201 |
| 1dqe | BOM | A | 300 |
| 1dqp | IMG | A | 300 |
| 1dqs | CRB | A | 401 |
| 1dr1 | HBI | A | 198 |
| 1dr2 | TAP | A | 191 |
| 1dr6 | MBO | A | 201 |
| 1drv | A3D | A | 301 |
| 1dry | AAG | A | 331 |
| 1ds1 | AKG | A | 330 |
| 1ds1 | PGO | A | 331 |
| 1dsy | PSF | A | 401 |
| 1dtl | BEP | A | 204 |
| 1dtm | 4MZ | A | 155 |
| 1dtn | APG | A | 399 |
| 1dtq | FPT | A | 999 |
| 1dtt | FTC | A | 999 |
| 1dtu | ADH | A | 693 |
| 1duo | 1MZ | A | 155 |
| 1duv | PSQ | G | 401 |
| 1dv2 | ATP | A1000 |  |
| 1dvp | CIT | A | 300 |
| 1dvs | STL | A | 125 |
| 1dvu | DBF | B | 125 |
| 1dvx | DIF | B | 125 |
| 1dvy | BPD | A | 125 |
| 1dvz | OFL | A | 125 |
| 1dx4 | 760 | A1580 |  |
| 1dx4 | BMA | A1579 |  |
| 1dxm | RED | A | 163 |
| 1dxr | MST | L | 502 |
| 1dy3 | 87Y | A | 201 |
| 1dy3 | ATP | A | 200 |
| 1dy4 | SNP | A | 437 |
| 1dyj | DDF | A | 161 |
| 1dzm | BZM | A | 600 |
| 1dzp | BZQ | A | 600 |
| 1dzt | ATY | B1000 |  |
| 1e06 | IPB | A | 600 |
| 1e0o | IDS | E | 10 |
| 1e0o | SGN | E | 9 |
| 1e12 | MPG | B | 701 |
| 1e1f | PSG | A | 513 |
| 1e1v | CMG | A | 401 |
| 1e1x | NW1 | A | 401 |
| 1e22 | ACP | A | 512 |
| 1e2i | APS | A | 500 |
| 1e2m | HPT | A | 500 |
| 1e2p | CCV | A | 500 |
| 1e2q | ATP | A | 302 |
| 1e2r | DHE | A | 602 |
| 1e36 | TPY | B | 280 |
| 1e3i | CXF | A | 378 |
| 1e3r | AND | A | 801 |
| 1e3v | DXC | A | 801 |
| 1e42 | DTD | A | 950 |
| 1e4i | NFG | A3000 |  |
| 1e55 | DHR | B | 514 |
| 1e5d | FMN | A | 403 |
| 1e5k | CIT | A | 302 |
| 1e5q | NDP | A | 500 |
| 1e5q | SHR | A | 501 |
| 1e66 | HUX | A | 803 |
| 1e6q | NTZ | M | 999 |
| 1e6s | GOX | M | 999 |
| 1e6u | UVW | A1318 |  |
| 1e6x | LGC | M | 999 |
| 1e73 | G2F | M | 999 |
| 1e79 | ATP | A | 600 |
| 1e7a | PFL | A4001 |  |
| 1e7b | HLT | A4001 |  |
| 1e7e | DKA | A1001 |  |
| 1e7h | PLM | A1001 |  |
| 1e7i | STE | A1001 |  |
| 1e7p | MLA | T | 7 |
| 1e7v | LY2 | A3095 |  |
| 1e7w | MTX | A | 301 |
| 1e7y | BG6 | A | 501 |
| 1e8c | UAG | A1498 |  |
| 1e8g | FCR | A | 601 |
| 1e8h | ADP | A | 600 |
| 1e8v | NDG | A1572 |  |
| 1e8w | QUE | A2095 |  |
| 1e8x | ATP | A3000 |  |
| 1e90 | MYC | A2095 |  |
| 1e92 | HBI | A1290 |  |
| 1e9a | Z5A | A | 301 |
| 1e9b | ATM | A | 301 |
| 1eah | MYR | 4 | 1 |
| 1eaz | CIT | A3001 |  |
| 1eb9 | HBA | A1259 |  |
| 1ebu | HSE | D1301 |  |
| 1ebu | NDA | D1300 |  |
| 1ebv | SCL | A | 700 |
| 1ebw | BEI | A | 501 |
| 1ebz | BEC | B | 501 |
| 1ec0 | BED | A | 501 |
| 1ec1 | BEE | A | 501 |
| 1ec2 | BEJ | B | 501 |
| 1ec3 | MS3 | A | 501 |
| 1ec8 | GLR | A | 499 |
| 1ec9 | XYH | A | 499 |
| 1ecb | 5GP | A | 505 |
| 1ecm | TSA | A | 500 |
| 1ecq | DXG | A | 499 |
| 1ecv | 878 | A | 301 |
| 1ed4 | IPU | A1830 |  |
| 1ed5 | NRG | A1705 |  |
| 1ed6 | ILO | A1765 |  |
| 1ee2 | CHD | A1150 |  |
| 1eef | I06 | G | 108 |
| 1eet | BFU | A2000 |  |
| 1eex | PGO | A | 602 |
| 1ef9 | 2CP | A | 270 |
| 1efd | GCR | N | 503 |
| 1efk | MAK | A | 603 |
| 1efy | BZC | A | 201 |
| 1eg2 | MTA | A | 401 |
| 1egh | PGA | A | 201 |
| 1egy | 9AP | A | 800 |
| 1eh4 | IC1 | A | 300 |
| 1eh5 | PLM | A | 430 |
| 1ei9 | NDG | A | 400 |
| 1ein | PLC | A | 601 |
| 1eix | BMQ | A1301 |  |
| 1ej0 | SAM | A | 301 |
| 1ej1 | M7G | A1000 |  |
| 1ejb | INJ | A | 200 |
| 1eji | THF | A | 600 |
| 1ejj | 3PG | A | 601 |
| 1ek8 | DEM | A | 901 |
| 1ekj | CIT | D3101 |  |
| 1el3 | I84 | A | 320 |
| 1el5 | DMG | A | 801 |
| 1el7 | MTD | A | 801 |
| 1el8 | MSF | A | 801 |
| 1el9 | MTG | A | 801 |
| 1elv | NES | A2003 |  |
| 1em6 | CP4 | A | 862 |
| 1enu | APZ | A | 400 |
| 1ep3 | FAD | B | 502 |
| 1ep4 | S11 | A | 999 |
| 1eq2 | ADQ | A2500 |  |
| 1eqc | CTS | A | 401 |
| 1eqg | IBP | A | 701 |
| 1eqm | ADP | A | 171 |
| 1equ | EQI | A | 329 |
| 1esb | BBL | A | 256 |
| 1esq | TZP | A | 320 |
| 1esz | CPO | A | 500 |
| 1ev1 | MYR | 4 | 1 |
| 1ev1 | PLM | 1 | 0 |
| 1eve | E20 | A2001 |  |
| 1evi | 2PC | A | 352 |
| 1evl | TSB | A2002 |  |
| 1ew8 | PAE | A | 556 |
| 1ew9 | MMQ | A | 559 |
| 1ewy | FAD | A | 304 |
| 1ex0 | PGO | A1340 |  |
| 1ex0 | PGO | A1341 |  |
| 1ex0 | PGO | B1343 |  |
| 1ex2 | SUC | A | 200 |
| 1ex8 | A4P | A | 171 |
| 1exa | 394 | A | 450 |
| 1exc | DUT | A | 201 |
| 1exx | 961 | A | 450 |
| 1ey3 | DAK | A | 500 |
| 1eye | PMM | A | 301 |
| 1eyn | 2AN | A | 550 |
| 1eys | BGL | L | 701 |
| 1eys | BGL | L | 704 |
| 1eys | BGL | M | 702 |
| 1eys | PEF | H | 708 |
| 1eyz | ANP | A | 400 |
| 1ez2 | DII | A1403 |  |
| 1ez9 | GLO | A | 380 |
| 1f06 | 2NP | B | 950 |
| 1f07 | MPO | A | 331 |
| 1f0l | APU | A | 601 |
| 1f0r | 815 | A | 401 |
| 1f0u | RPR | A | 501 |
| 1f0x | FAD | A | 600 |
| 1f12 | 3HC | A | 351 |
| 1f1h | ADP | A1471 |  |
| 1f1v | DHY | A | 999 |
| 1f3e | DPZ | A | 400 |
| 1f3f | D4D | B | 163 |
| 1f3f | D4T | A | 160 |
| 1f4c | TP2 | A | 801 |
| 1f4e | TPR | A | 301 |
| 1f4f | TP3 | A | 701 |
| 1f4g | TP4 | A | 601 |
| 1f57 | DCY | A | 308 |
| 1f5l | AMR | A | 301 |
| 1f7p | UDP | A | 701 |
| 1f8c | 4AM | A | 4 |
| 1f8d | 9AM | A | 0 |
| 1f8i | GLV | A | 461 |
| 1f8r | CIT | A | 526 |
| 1f8y | 5MD | A | 158 |
| 1f91 | DKA | A | 901 |
| 1f92 | UKP | A | 300 |
| 1f9b | 3ID | A | 694 |
| 1f9o | SSG | A2096 |  |
| 1f9p | ESA | A | 101 |
| 1f9v | ADP | A | 998 |
| 1fbf | AHM | A | 336 |
| 1fbl | HTA | A | 900 |
| 1fbo | GLO | A2101 |  |
| 1fbz | CC1 | A | 150 |
| 1fc4 | AKB | A1200 |  |
| 1fcp | FCI | A | 750 |
| 1fcx | 184 | A | 450 |
| 1fcy | 564 | A | 450 |
| 1fcy | LMU | A | 451 |
| 1fcz | 156 | A | 450 |
| 1fd0 | 254 | A | 450 |
| 1fdj | G3P | B6003 |  |
| 1fdk | GLE | A | 150 |
| 1fdq | HXA | A | 133 |
| 1fe0 | SUC | A | 205 |
| 1fen | AZE | A | 184 |
| 1ffx | GTP | A | 500 |
| 1fg3 | HSA | A | 501 |
| 1fgh | ATH | A | 755 |
| 1fgi | SU1 | A1001 |  |
| 1fh9 | LOX | A | 602 |
| 1fhd | XIM | A | 602 |
| 1fhw | I5P | A1001 |  |
| 1fi1 | RIF | A1022 |  |
| 1fig | TSA | H | 224 |
| 1fit | FRU | A | 500 |
| 1fiw | FUL | A | 300 |
| 1fk0 | DKA | A | 201 |
| 1fk2 | MYR | A | 201 |
| 1fk3 | PAM | A | 201 |
| 1fk4 | STE | A | 201 |
| 1fk5 | OLA | A | 201 |
| 1fk6 | LNL | A1201 |  |
| 1fk7 | RCL | A | 201 |
| 1fk9 | EFZ | A | 999 |
| 1fkw | PUR | A | 353 |
| 1flc | NDG | A1500 |  |
| 1flc | NDG | B1301 |  |
| 1flr | FLU | L | 600 |
| 1fm4 | DXC | A1001 |  |
| 1fm7 | DFV | A | 501 |
| 1fm8 | DDC | A | 501 |
| 1fmc | CHO | A | 257 |
| 1fmw | ATP | A | 999 |
| 1fnd | A2P | A | 316 |
| 1fo3 | KIF | A | 704 |
| 1fo4 | SAL | A3005 |  |
| 1foa | UD1 | A | 449 |
| 1foi | 14W | A | 810 |
| 1foj | 7I2 | B1770 |  |
| 1fp1 | HCC | D2000 |  |
| 1fp2 | HMO | A2000 |  |
| 1fp2 | SAH | A1699 |  |
| 1fpd | AHG | A | 338 |
| 1fpx | SAM | A1699 |  |
| 1fpy | PPQ | A5900 |  |
| 1fq9 | IDS | A | 303 |
| 1fq9 | SGN | A | 304 |
| 1fq9 | UAP | A | 301 |
| 1fqo | FPC | A1267 |  |
| 1fro | GSB | A | 200 |
| 1frw | GTP | A | 198 |
| 1fs5 | 16G | A1267 |  |
| 1fsg | 9DG | A | 304 |
| 1fsw | CTB | A | 964 |
| 1fsy | 105 | A | 964 |
| 1ft4 | 703 | A | 962 |
| 1ftl | DNQ | A | 365 |
| 1ftm | AMQ | A | 428 |
| 1ftq | GL2 | A | 998 |
| 1ftw | GL5 | A | 998 |
| 1fty | GL7 | A | 998 |
| 1fu4 | GL9 | A | 998 |
| 1fu7 | CR1 | A | 998 |
| 1fui | FOC | A | 594 |
| 1fuo | CIT | A | 469 |
| 1fup | PMA | A | 474 |
| 1fuq | SIF | A1000 |  |
| 1fur | MLT | A | 468 |
| 1fuy | FIP | A | 269 |
| 1fv0 | 9AR | A | 401 |
| 1fv9 | 172 | A | 246 |
| 1fvt | 106 | A | 299 |
| 1fvv | 107 | A | 501 |
| 1fwt | E4P | A1269 |  |
| 1fwu | SGA | A2001 |  |
| 1fxo | TMP | A8500 |  |
| 1fxq | A5P | A1269 |  |
| 1fxu | GU7 | A | 290 |
| 1fyf | SSA | A1001 |  |
| 1fzd | NDG | B | 11 |
| 1fzk | FUL | A | 602 |
| 1fzz | 681 | A | 395 |
| 1g0i | INS | A | 594 |
| 1g0n | PHH | A | 402 |
| 1g0r | G1P | A2500 |  |
| 1g0r | THM | A2530 |  |
| 1g12 | MAN | A | 900 |
| 1g1l | CIT | A3800 |  |
| 1g1l | DAU | A3500 |  |
| 1g1t | SIA | A | 601 |
| 1g1y | BCD | A | 601 |
| 1g27 | BB1 | A1001 |  |
| 1g2k | NM1 | B | 501 |
| 1g2l | T87 | A | 1 |
| 1g2n | EPH | A4000 |  |
| 1g32 | R11 | B | 1 |
| 1g35 | AHF | B | 501 |
| 1g3l | TRH | A | 500 |
| 1g3m | PCQ | A | 712 |
| 1g42 | CP2 | A | 701 |
| 1g45 | FSB | A | 555 |
| 1g48 | F6B | A | 555 |
| 1g49 | 111 | B | 901 |
| 1g4h | 1BO | A | 501 |
| 1g4j | FFB | A | 555 |
| 1g4k | HQQ | A | 306 |
| 1g4o | BSB | A | 555 |
| 1g4p | FQP | A2001 |  |
| 1g4t | FTP | A2001 |  |
| 1g51 | AMO | A | 831 |
| 1g52 | F2B | A | 555 |
| 1g55 | SAH | A | 392 |
| 1g5s | I17 | A | 400 |
| 1g5t | ATP | A | 999 |
| 1g60 | SAM | A | 500 |
| 1g67 | ICP | A2001 |  |
| 1g67 | TZP | A2005 |  |
| 1g6c | IFP | A2001 |  |
| 1g6h | ADP | A | 300 |
| 1g7c | 5GP | A | 507 |
| 1g7f | INZ | A | 500 |
| 1g7g | INX | A | 600 |
| 1g86 | NEQ | A | 153 |
| 1g8x | ADP | A1100 |  |
| 1g93 | UPG | A | 500 |
| 1g94 | DAF | A | 598 |
| 1g98 | PA5 | A | 558 |
| 1g9j | SGC | A | 631 |
| 1g9r | UPF | A | 401 |
| 1g9v | RQ3 | A | 801 |
| 1ga8 | DEL | A | 500 |
| 1gaf | NPE | H | 218 |
| 1gai | MAN | A | 474 |
| 1gc5 | ADP | A | 470 |
| 1gc6 | I3P | A1229 |  |
| 1gcz | YZ9 | A | 900 |
| 1gd0 | CIT | A | 551 |
| 1gdk | ISQ | A | 500 |
| 1gg5 | E09 | A | 703 |
| 1gg6 | APL | B | 302 |
| 1gg8 | GLG | A | 998 |
| 1ghq | NDG | C | 701 |
| 1ghz | 120 | A | 246 |
| 1gi0 | BMZ | A | 246 |
| 1gi4 | 122 | A | 246 |
| 1gi5 | 123 | A | 246 |
| 1gi6 | 124 | A | 246 |
| 1gii | 1PU | A | 501 |
| 1gij | 2PU | A | 501 |
| 1gj5 | 130 | H | 410 |
| 1gj6 | 132 | A | 246 |
| 1gj7 | CIT | B | 1 |
| 1gj8 | 133 | B | 251 |
| 1gj9 | 134 | B | 251 |
| 1gjd | 136 | B | 251 |
| 1gjv | SAP | A1383 |  |
| 1gka | D12 | A1183 |  |
| 1gkd | BUM | A1449 |  |
| 1gkd | STN | A1448 |  |
| 1gkz | ADP | A | 601 |
| 1gl9 | ANP | B2055 |  |
| 1glc | G3H | G | 503 |
| 1glj | ATS | O | 601 |
| 1gll | ACP | O | 601 |
| 1glp | GTS | A | 210 |
| 1glq | GTB | A | 210 |
| 1gm9 | SOX | B1569 |  |
| 1gmn | SGN | A1209 |  |
| 1gmo | IDS | B1209 |  |
| 1gmo | SGN | B1210 |  |
| 1gmy | AEM | A | 500 |
| 1gmy | APD | A | 501 |
| 1gmy | DFA | A | 502 |
| 1gn8 | ATP | A | 700 |
| 1gnj | ACD | A1001 |  |
| 1gnx | SUC | A | 600 |
| 1gp6 | DH2 | A | 385 |
| 1gp6 | QUE | A | 380 |
| 1gpe | BMA | A | 606 |
| 1gpe | MAN | A | 607 |
| 1gpj | CIT | A1407 |  |
| 1gpk | HUP | A1540 |  |
| 1gpm | CIT | A | 529 |
| 1gpy | G6P | A | 998 |
| 1gqc | CMK | A1244 |  |
| 1gqg | DCD | A1351 |  |
| 1gqh | KOJ | A1351 |  |
| 1gql | BDP | A1706 |  |
| 1gqr | SAF | A1998 |  |
| 1gqy | ACP | A1476 |  |
| 1gra | GSH | A | 481 |
| 1gra | NDP | A | 480 |
| 1grb | NDP | A | 483 |
| 1grh | FAD | A | 479 |
| 1gs5 | ANP | A1260 |  |
| 1gs5 | NLG | A1259 |  |
| 1gt1 | 3OL | B1159 |  |
| 1gt1 | ANC | A1161 |  |
| 1gt3 | DHM | A1160 |  |
| 1gt4 | UNA | A1160 |  |
| 1gt6 | OLA | A1270 |  |
| 1gt8 | UAA | A1035 |  |
| 1gtb | PZQ | A | 901 |
| 1gte | FAD | A1031 |  |
| 1gte | IUR | A1034 |  |
| 1gth | IDH | B1034 |  |
| 1gth | URA | D1034 |  |
| 1gtv | TMP | B | 302 |
| 1gtv | TYD | A | 302 |
| 1gtz | DHK | A1162 |  |
| 1gu1 | FA1 | A | 201 |
| 1guh | GSB | A | 223 |
| 1guq | GUD | A | 352 |
| 1gvc | NTA | A1252 |  |
| 1gvf | PGH | A | 287 |
| 1gvg | PCX | A | 331 |
| 1gvh | HEM | A1398 |  |
| 1gvo | DNF | A | 500 |
| 1gvq | CYH | A | 500 |
| 1gvr | TNL | A | 500 |
| 1gw1 | MAF | A1427 |  |
| 1gw1 | MBF | A1428 |  |
| 1gwe | HEM | A | 504 |
| 1gwm | BGC | A1156 |  |
| 1gwq | ZTW | A | 600 |
| 1gx5 | GTP | A1531 |  |
| 1gx6 | UTP | A1001 |  |
| 1gxa | PLM | A1163 |  |
| 1gxs | DKA | A | 701 |
| 1gym | MYG | A | 702 |
| 1gyy | FHC | A | 500 |
| 1gz4 | ATP | A | 601 |
| 1gz4 | TTN | A | 603 |
| 1gz5 | G6P | A | 901 |
| 1gz8 | MBP | A1300 |  |
| 1gzf | ADP | D1247 |  |
| 1gzm | PEF | A1341 |  |
| 1gzq | D12 | A1284 |  |
| 1gzr | C15 | B1067 |  |
| 1h00 | FAP | A1300 |  |
| 1h00 | FCP | A1400 |  |
| 1h01 | FAL | A | 301 |
| 1h01 | FBL | A | 302 |
| 1h08 | BWP | A | 300 |
| 1h08 | BYP | A | 299 |
| 1h0a | I3P | A1164 |  |
| 1h0c | AOA | A1392 |  |
| 1h0j | SDS | A1061 |  |
| 1h0s | FA6 | A | 200 |
| 1h0v | UN4 | A1299 |  |
| 1h0w | 207 | A1299 |  |
| 1h11 | FCT | A | 500 |
| 1h12 | XYS | A | 502 |
| 1h16 | DTL | A9009 |  |
| 1h16 | PYR | A1001 |  |
| 1h17 | OXM | A1001 |  |
| 1h1d | BIA | A | 335 |
| 1h1i | QUE | A1358 |  |
| 1h1l | CIT | A1479 |  |
| 1h1m | KMP | A1360 |  |
| 1h1o | HEM | A1184 |  |
| 1h1q | 2A6 | A1298 |  |
| 1h1r | 6CP | A1298 |  |
| 1h1s | 4SP | A1298 |  |
| 1h2b | OCA | A1360 |  |
| 1h2j | DCB | A1304 |  |
| 1h2t | 7MG | Z1152 |  |
| 1h2y | ZPR | A | 791 |
| 1h35 | R01 | A | 800 |
| 1h36 | R88 | A | 800 |
| 1h39 | R03 | A | 800 |
| 1h3a | R04 | A | 800 |
| 1h3c | R79 | A | 800 |
| 1h3m | N2P | A1231 |  |
| 1h3n | LMS | A1816 |  |
| 1h41 | GCV | A1713 |  |
| 1h47 | GPP | A1157 |  |
| 1h49 | HBO | A1502 |  |
| 1h5u | CHI | A | 920 |
| 1h5v | MA3 | A1309 |  |
| 1h5v | SGC | A1307 |  |
| 1h5v | SSG | A1306 |  |
| 1h62 | ANB | A | 501 |
| 1h6c | SIN | A | 600 |
| 1h6d | NDP | A | 500 |
| 1h72 | ANP | C1301 |  |
| 1h72 | HSE | C1300 |  |
| 1h74 | ADP | A | 400 |
| 1h74 | SAP | B | 400 |
| 1h78 | DCP | A1588 |  |
| 1h79 | TTP | A1586 |  |
| 1h7a | DTP | A1590 |  |
| 1h7l | TYD | A1258 |  |
| 1h7n | SHF | A1341 |  |
| 1h7t | SIA | B1243 |  |
| 1h7u | SAP | A1366 |  |
| 1h7x | NDP | D1032 |  |
| 1h7x | URF | A1033 |  |
| 1h83 | DIA | A | 590 |
| 1h8l | GEM | A | 801 |
| 1h8l | NDG | A | 921 |
| 1h8t | MYR | D3500 |  |
| 1h9g | MYR | A1229 |  |
| 1h9u | LG2 | A | 1 |
| 1h9z | WRR | A3001 |  |
| 1ha2 | WRS | A3001 |  |
| 1ha3 | MAU | A | 408 |
| 1hak | K21 | A | 901 |
| 1hbk | MYR | A1090 |  |
| 1hcx | DDQ | B1319 |  |
| 1hcx | TPT | A1319 |  |
| 1hcy | NDG | A | 658 |
| 1hdq | INF | A1308 |  |
| 1hdu | ING | A1309 |  |
| 1hdx | CXL | A | 378 |
| 1he2 | BLA | A1207 |  |
| 1he3 | MBV | A1207 |  |
| 1he4 | FMN | A1207 |  |
| 1hee | LHY | A1309 |  |
| 1hfc | PLH | A | 280 |
| 1hg0 | SIN | A | 350 |
| 1hg1 | DAS | A | 350 |
| 1hgg | SIA | A | 354 |
| 1hiy | 3AN | A1156 |  |
| 1hj1 | AOE | A1436 |  |
| 1hj1 | PMB | A1437 |  |
| 1hj9 | ANL | A | 246 |
| 1hjg | KIV | A1308 |  |
| 1hk8 | DGT | A1587 |  |
| 1hkk | AMI | A1388 |  |
| 1hkn | N2M | C4138 |  |
| 1hlf | GL4 | A | 998 |
| 1hlk | 113 | A2002 |  |
| 1hm2 | IDR | A | 710 |
| 1hm2 | IDR | A | 711 |
| 1hm2 | MAN | A | 706 |
| 1hm9 | UD1 | A1500 |  |
| 1hms | OLA | A | 133 |
| 1hmt | STE | A | 133 |
| 1hmw | GCD | A | 710 |
| 1hmw | NG6 | A | 712 |
| 1hni | AAA | A | 559 |
| 1hnj | MLC | A | 350 |
| 1hnn | SKF | A3001 |  |
| 1hon | GNH | A | 432 |
| 1hp1 | ATP | A | 606 |
| 1hpu | A12 | A1602 |  |
| 1hqc | ADE | A | 501 |
| 1hqd | INK | A | 612 |
| 1hqj | SIN | A | 1 |
| 1hqs | CIT | A | 425 |
| 1hqs | PGO | A | 901 |
| 1hqu | HBY | A | 561 |
| 1hri | S57 | 1 | 290 |
| 1hrs | PP9 | A | 200 |
| 1hrv | SDZ | 1 | 682 |
| 1hs6 | BES | A | 901 |
| 1hsl | HIS | A | 239 |
| 1hsr | BHO | A | 800 |
| 1ht5 | FL2 | A1701 |  |
| 1ht8 | 34C | A1701 |  |
| 1hte | G23 | A | 200 |
| 1hto | CIT | A7476 |  |
| 1htw | ADP | A | 560 |
| 1hu9 | 4HM | A | 861 |
| 1hv5 | RXP | A6001 |  |
| 1hv6 | MAV | A | 402 |
| 1hv6 | MAW | A | 401 |
| 1hvv | TAR | C1352 |  |
| 1hw5 | CMP | A | 211 |
| 1hw8 | 114 | A | 2 |
| 1hw8 | ADP | B | 101 |
| 1hw8 | ADP | D | 102 |
| 1hw9 | SIM | A | 4 |
| 1hwi | 115 | A | 2 |
| 1hwj | 116 | A | 2 |
| 1hwk | 117 | A | 2 |
| 1hwl | FBI | A | 2 |
| 1hwr | 216 | B | 216 |
| 1hwy | AKG | A | 506 |
| 1hwz | NDP | A | 551 |
| 1hx0 | AC1 | A1990 |  |
| 1hxp | UDP | B | 352 |
| 1hxs | PLM | 12000 |  |
| 1hy7 | MBS | B | 901 |
| 1hyo | HBU | A1012 |  |
| 1hyt | BZS | A | 807 |
| 1hyv | TTA | A | 304 |
| 1hzj | UD1 | A | 401 |
| 1hzy | PEL | A | 428 |
| 1i05 | LTL | A | 408 |
| 1i0l | 7HP | A | 800 |
| 1i0v | 2GP | A | 106 |
| 1i1d | 16G | A | 905 |
| 1i1l | 2ML | A | 414 |
| 1i24 | UPG | A | 402 |
| 1i29 | LPG | A | 460 |
| 1i2b | USQ | A2402 |  |
| 1i2d | ADX | A | 575 |
| 1i2w | OUT | B2301 |  |
| 1i2z | 654 | A | 502 |
| 1i30 | 826 | A | 302 |
| 1i32 | NMD | A | 361 |
| 1i33 | TND | A | 361 |
| 1i3a | NCO | A | 300 |
| 1i3h | MAN | A | 238 |
| 1i3u | RR1 | A | 401 |
| 1i41 | HEN | A | 500 |
| 1i43 | PMC | A | 600 |
| 1i48 | CCO | A | 600 |
| 1i58 | ACP | A | 998 |
| 1i58 | ADP | B | 999 |
| 1i5d | 128 | A | 999 |
| 1i5g | TS5 | A | 401 |
| 1i5o | PAL | B | 998 |
| 1i5r | HYC | A | 328 |
| 1i6i | ACP | A | 500 |
| 1i6k | TYM | A | 350 |
| 1i76 | BSI | A | 1 |
| 1i7e | IBS | A2001 |  |
| 1i7g | CPQ | A | 470 |
| 1i80 | 9HX | A | 301 |
| 1i80 | IMR | A | 351 |
| 1i82 | BGC | A | 189 |
| 1i83 | NTU | A | 840 |
| 1i8n | ROP | A | 801 |
| 1i8z | INL | A | 555 |
| 1i91 | INQ | A | 555 |
| 1i9l | INV | A | 555 |
| 1i9m | INW | A | 555 |
| 1i9n | IOA | A | 555 |
| 1i9o | IOC | A | 555 |
| 1i9p | IOE | A | 555 |
| 1i9q | IOF | A | 555 |
| 1i9z | 2IP | A | 302 |
| 1ia1 | TQ3 | A | 194 |
| 1ia2 | TQ4 | A | 194 |
| 1ia3 | TQ5 | A | 194 |
| 1ia4 | TQ6 | A | 194 |
| 1ia9 | ANP | A2002 |  |
| 1iah | ADP | A2002 |  |
| 1iay | AVG | A | 501 |
| 1ibv | PYR | B | 82 |
| 1ic1 | NDG | B | 313 |
| 1icm | MYR | A | 133 |
| 1icq | OPD | A | 503 |
| 1id0 | ANP | A | 487 |
| 1idt | CB1 | A1219 |  |
| 1ie8 | KH1 | A | 500 |
| 1iei | ZES | A | 351 |
| 1ieq | BGC | A | 619 |
| 1iev | INS | A | 617 |
| 1iew | G2F | A | 620 |
| 1iex | TCB | A | 617 |
| 1if2 | 129 | A | 600 |
| 1if4 | FBS | A | 555 |
| 1if5 | FBT | A | 555 |
| 1if6 | FBU | A | 555 |
| 1ig0 | VIB | A | 702 |
| 1igb | IPO | A | 520 |
| 1igx | EPA | A | 700 |
| 1igz | BGC | A | 750 |
| 1ih7 | GMP | A1000 |  |
| 1ih8 | APC | A3001 |  |
| 1ihi | IU5 | A | 326 |
| 1ihu | TAS | A | 701 |
| 1ihx | SND | A | 335 |
| 1ihx | SND | C | 337 |
| 1ihy | APR | A | 335 |
| 1iig | 3PP | B | 600 |
| 1iin | UPG | A1501 |  |
| 1ij8 | NDG | A | 401 |
| 1ijr | CC0 | A | 150 |
| 1ik4 | PGH | A | 153 |
| 1ikg | REX | A | 400 |
| 1iki | REY | A | 400 |
| 1ikx | PNU | A2000 |  |
| 1iky | MSD | A2000 |  |
| 1il3 | 7DG | A | 301 |
| 1il4 | 9DG | A | 301 |
| 1il5 | DDP | A | 301 |
| 1im8 | SAI | A | 302 |
| 1imx | CPQ | A | 101 |
| 1in4 | ADP | A | 700 |
| 1inc | ICL | A | 260 |
| 1ing | ST5 | A | 471 |
| 1iop | HE6 | A | 154 |
| 1iow | ADP | A | 310 |
| 1ipb | GTA | A1000 |  |
| 1iql | XMJ | A | 401 |
| 1iqm | XMK | A | 401 |
| 1iqu | TDR | A | 500 |
| 1isg | SAP | A1002 |  |
| 1ish | ENP | A1001 |  |
| 1iss | MCG | A1001 |  |
| 1it6 | CYU | A | 501 |
| 1itu | CIL | A | 451 |
| 1iut | PAB | A | 396 |
| 1ivc | ST2 | A | 471 |
| 1ivd | FUL | A | 477 |
| 1ivd | ST1 | A | 471 |
| 1ive | ST3 | A | 471 |
| 1iw0 | HEM | A | 901 |
| 1iw0 | SUC | B | 904 |
| 1iwe | IMP | A | 460 |
| 1iwh | PEM | A | 501 |
| 1ix1 | MHA | A | 401 |
| 1ixn | G3P | A1001 |  |
| 1iy7 | CXA | A | 500 |
| 1iyb | 5GP | A1295 |  |
| 1iyd | GUA | A | 414 |
| 1iyk | MIM | A | 502 |
| 1iyl | R64 | A | 452 |
| 1iz2 | SUM | A | 800 |
| 1izc | PYR | A2001 |  |
| 1izh | Q50 | B1001 |  |
| 1izl | HEM | 0 | 138 |
| 1izo | PAM | A | 601 |
| 1j01 | XIL | A | 601 |
| 1j09 | ATP | A | 501 |
| 1j0d | 5PA | A1401 |  |
| 1j17 | ZEN | T | 1 |
| 1j1a | BHP | A1301 |  |
| 1j1n | LGU | A | 603 |
| 1j1s | FMP | A | 701 |
| 1j20 | AS1 | A | 520 |
| 1j2y | DQA | A | 219 |
| 1j2z | SOG | A | 409 |
| 1j39 | UPG | A | 400 |
| 1j3k | WRA | A | 609 |
| 1j3n | CIT | A | 500 |
| 1j4h | SUB | A | 201 |
| 1j4i | TST | A | 201 |
| 1j4r | 001 | A | 108 |
| 1j51 | TCZ | A1450 |  |
| 1j77 | HEM | A | 300 |
| 1j78 | OLA | A | 459 |
| 1j79 | ORO | A | 410 |
| 1j7k | ATP | A2060 |  |
| 1j99 | AND | A | 401 |
| 1j9c | FUL | L | 360 |
| 1j9z | FAD | B | 850 |
| 1ja1 | FMN | A1751 |  |
| 1ja9 | PYQ | A | 406 |
| 1jak | IFG | A | 601 |
| 1jay | F42 | A | 214 |
| 1jb0 | LHG | A5001 |  |
| 1jb0 | LHG | A5003 |  |
| 1jb0 | LHG | B5004 |  |
| 1jbv | ACP | A | 999 |
| 1jbw | ACQ | A | 999 |
| 1jbw | TMF | A | 996 |
| 1jcn | CPR | A | 631 |
| 1jct | GKR | A | 499 |
| 1jcx | PAI | A1300 |  |
| 1jcy | R5P | A1269 |  |
| 1jd0 | AZM | A1400 |  |
| 1jdj | CFP | A | 367 |
| 1jdp | NDG | A | 501 |
| 1jdz | FMB | A | 270 |
| 1je1 | GMP | A1260 |  |
| 1jeo | CIT | A3000 |  |
| 1jep | DFL | A | 501 |
| 1jfb | HEM | A | 501 |
| 1jfh | MA1 | A | 994 |
| 1jfh | MA2 | A | 991 |
| 1jfh | MA3 | A | 992 |
| 1jg0 | DDT | A | 304 |
| 1jg1 | SAH | A | 500 |
| 1jg4 | SAM | A | 500 |
| 1jg7 | UDP | A | 400 |
| 1jgi | SUC | A2064 |  |
| 1jgt | CMA | A | 803 |
| 1jgu | HBC | H3001 |  |
| 1jh1 | JST | A1000 |  |
| 1jh7 | UVC | A | 201 |
| 1jh8 | ADE | A | 999 |
| 1jha | AAM | A | 990 |
| 1jhm | 5MB | A | 990 |
| 1jho | RMB | A | 990 |
| 1jhp | 5OB | A | 990 |
| 1jhq | PMO | A | 990 |
| 1jhr | P2P | A | 990 |
| 1jhu | PCR | A | 990 |
| 1jib | MTT | A | 601 |
| 1jim | ICU | A | 251 |
| 1jip | KTN | A | 801 |
| 1jiz | CGS | A | 998 |
| 1jj0 | SUC | A2380 |  |
| 1jj9 | BBT | A1000 |  |
| 1jje | BYS | A | 250 |
| 1jjt | BDS | A | 250 |
| 1jk7 | OKA | A | 501 |
| 1jki | DG6 | A | 630 |
| 1jkx | 138 | A1221 |  |
| 1jla | TNK | A | 999 |
| 1jlr | GTP | A | 303 |
| 1jlx | A2G | A | 305 |
| 1jn2 | SFP | P1001 |  |
| 1jnh | ECO | B | 351 |
| 1jnq | EGT | A | 871 |
| 1jnr | FAD | A1000 |  |
| 1joc | ITP | A | 101 |
| 1jom | FFO | A | 161 |
| 1jpz | 140 | A1470 |  |
| 1jqn | DCO | A | 901 |
| 1jr8 | FAD | A | 335 |
| 1js8 | MAN | A | 893 |
| 1js8 | NDG | A | 890 |
| 1jsv | U55 | A | 400 |
| 1jsz | NDM | A | 600 |
| 1jt6 | DEQ | A | 201 |
| 1jtq | LY3 | A | 501 |
| 1jtu | LYB | A | 501 |
| 1jut | LYD | A | 303 |
| 1juy | H5P | A | 433 |
| 1jv2 | NDG | B3560 |  |
| 1jv2 | NDG | B3655 |  |
| 1jv3 | UD2 | A | 901 |
| 1jv4 | TZL | A | 170 |
| 1jvi | HCS | A | 402 |
| 1jw0 | GUA | B | 999 |
| 1jwa | ATP | B | 250 |
| 1jwt | BLI | A | 300 |
| 1jwy | BGC | A | 803 |
| 1jxi | HMH | A2001 |  |
| 1jxn | MFU | A | 501 |
| 1jyv | 145 | A2001 |  |
| 1jyv | 145 | A2002 |  |
| 1jyw | 147 | A2001 |  |
| 1jyx | IPT | A2002 |  |
| 1jyy | 2FL | A2001 |  |
| 1jz2 | 2FG | A2001 |  |
| 1jz2 | 2FG | B2001 |  |
| 1jz3 | 2DG | A2001 |  |
| 1jz4 | 2DG | B2001 |  |
| 1jz5 | 149 | A2001 |  |
| 1jz6 | GTZ | A2001 |  |
| 1jze | DRU | A | 903 |
| 1jze | LRU | A | 904 |
| 1jzf | IME | A | 902 |
| 1jzg | IMF | A | 902 |
| 1jzi | REP | A | 903 |
| 1jzj | DOS | A | 903 |
| 1jzj | LOS | A | 904 |
| 1jzq | ILA | A1301 |  |
| 1jzs | MRC | A1301 |  |
| 1k06 | BZD | A | 920 |
| 1k0i | PHB | A | 396 |
| 1k0j | NDP | A | 398 |
| 1k0u | DEA | A1433 |  |
| 1k1i | FD1 | A | 999 |
| 1k1j | FD2 | A | 999 |
| 1k1m | FD4 | A | 999 |
| 1k1n | CCR | A | 999 |
| 1k1y | MAL | B | 660 |
| 1k2o | RFA | A | 900 |
| 1k2u | TFM | A1820 |  |
| 1k3a | ACP | A | 300 |
| 1k3c | ADP | A | 541 |
| 1k3u | IAD | A | 801 |
| 1k3y | GTX | A5100 |  |
| 1k4h | APQ | A | 900 |
| 1k4w | STE | A1001 |  |
| 1k4y | 4PN | A2001 |  |
| 1k5m | SPH | A1971 |  |
| 1k6e | 1BP | A2002 |  |
| 1k6e | PGO | A2001 |  |
| 1k6v | XN2 | B | 252 |
| 1k70 | HPY | A | 501 |
| 1k72 | CBI | A | 777 |
| 1k7f | IAV | A | 401 |
| 1k7h | MAE | A | 487 |
| 1k7w | AS1 | A1004 |  |
| 1k8y | 13P | A | 701 |
| 1k8z | IAG | A | 501 |
| 1k90 | 3AT | A | 902 |
| 1k9s | FM1 | D9907 |  |
| 1k9s | FM2 | A9901 |  |
| 1ka1 | A3P | A | 601 |
| 1kah | HIS | A | 502 |
| 1kak | FNP | A | 301 |
| 1kb0 | TFB | A1810 |  |
| 1kb9 | PEF | C | 510 |
| 1kb9 | PEF | C | 513 |
| 1kbc | HLE | A | 1 |
| 1kbc | RIN | A | 2 |
| 1kbi | HEM | A | 760 |
| 1kbo | 340 | A | 703 |
| 1kbq | 936 | A | 703 |
| 1kc3 | TRH | A | 601 |
| 1kc7 | PPR | A1000 |  |
| 1kcc | GTR | A1001 |  |
| 1kdg | 6FA | A6801 |  |
| 1kdg | EMT | A6907 |  |
| 1kds | NPB | A | 364 |
| 1kdt | DOC | A5407 |  |
| 1ke0 | CVB | A | 364 |
| 1ke5 | LS1 | A | 299 |
| 1ke6 | LS2 | A | 299 |
| 1ke7 | LS3 | A | 299 |
| 1ke8 | LS4 | A | 299 |
| 1ke9 | LS5 | A | 299 |
| 1kej | DAD | A | 601 |
| 1kek | HTL | A2236 |  |
| 1kel | AAH | H | 219 |
| 1kev | NDP | A | 352 |
| 1kf6 | HQO | C | 700 |
| 1kfd | CTP | A | 1 |
| 1kfg | GS1 | A | 618 |
| 1kgi | T4A | C | 128 |
| 1kgj | FL8 | B | 328 |
| 1kgq | NPI | A | 301 |
| 1kgq | SCO | A | 302 |
| 1kgt | PML | A | 301 |
| 1kh3 | ANP | A | 510 |
| 1khf | PEP | A | 703 |
| 1khr | VIR | A | 401 |
| 1ki0 | BCN | A | 335 |
| 1ki3 | PE2 | A | 1 |
| 1ki4 | BTD | A | 1 |
| 1ki7 | ID2 | A | 1 |
| 1ki8 | BVD | A | 1 |
| 1kie | AD3 | A | 801 |
| 1kiu | MMA | B | 500 |
| 1kj8 | ATP | A | 1 |
| 1kji | ACP | A | 1 |
| 1kjo | PHQ | A | 317 |
| 1kjq | ADP | A | 1 |
| 1kjq | MPO | A | 397 |
| 1kjr | BEK | A | 500 |
| 1kl2 | FON | A | 505 |
| 1klk | PMD | A | 653 |
| 1klm | SPP | A | 999 |
| 1km6 | OMP | A2001 |  |
| 1kme | BGC | B | 501 |
| 1kmv | NDP | A | 202 |
| 1kn2 | PNE | L | 551 |
| 1knf | MBD | A | 301 |
| 1kno | PNP | B | 551 |
| 1knu | YPA | A1001 |  |
| 1kny | APC | A | 556 |
| 1ko5 | ATP | A | 302 |
| 1ko8 | 6PG | A | 502 |
| 1kof | ACP | A | 500 |
| 1koj | PAN | A | 902 |
| 1kok | HIF | A | 296 |
| 1kor | ANP | A | 510 |
| 1kor | SIN | A | 530 |
| 1kou | DHC | A | 201 |
| 1kp2 | ATP | A | 459 |
| 1kp8 | ATP | A | 1 |
| 1kpe | ADW | B | 127 |
| 1kpg | SAH | A1900 |  |
| 1kph | 10A | A1901 |  |
| 1kpm | VIT | A | 401 |
| 1kq0 | MED | A | 601 |
| 1kqf | HEM | C | 809 |
| 1kqf | HEM | C | 810 |
| 1kqg | HQO | C | 811 |
| 1kr6 | DGL | A1317 |  |
| 1krh | FAD | A | 501 |
| 1kro | DTH | A1317 |  |
| 1kru | IPT | A | 209 |
| 1krv | 147 | A | 206 |
| 1ks7 | DAS | A1317 |  |
| 1ksk | URA | A | 232 |
| 1ksw | NBS | A | 1 |
| 1ksz | PGS | A | 440 |
| 1kt8 | ILP | A | 400 |
| 1kta | KIV | B3001 |  |
| 1kti | AZC | A | 998 |
| 1ktt | C02 | B | 1 |
| 1ktw | G4S | A | 2 |
| 1kvk | ATP | A | 535 |
| 1kvl | THN | B | 372 |
| 1kw6 | BPY | B | 401 |
| 1kwf | BGC | A | 401 |
| 1kwq | SG1 | A | 900 |
| 1kwr | SG2 | A | 900 |
| 1kws | UGA | A | 404 |
| 1kwu | MMA | A | 501 |
| 1kww | MFU | A | 501 |
| 1kwx | MFB | A | 501 |
| 1kxg | CIT | A | 889 |
| 1kxm | BZI | A | 2 |
| 1ky5 | ADY | A | 433 |
| 1kya | XYD | A | 514 |
| 1kyn | KTP | A | 601 |
| 1kyw | HFL | F | 0 |
| 1kyx | CRM | A | 501 |
| 1kz8 | PFE | A | 738 |
| 1kza | MAN | 11001 |  |
| 1kzb | MAN | 11501 |  |
| 1kzc | MAN | 11001 |  |
| 1kze | MAN | 11001 |  |
| 1kzi | THG | A | 303 |
| 1kzk | JE2 | A | 701 |
| 1kzl | CRM | A | 501 |
| 1kzn | CBN | A | 1 |
| 1kzo | FAR | C | 12 |
| 1l0l | FMX | C | 611 |
| 1l1q | 9DA | A | 201 |
| 1l2q | X7O | A | 601 |
| 1l2s | STC | A1115 |  |
| 1l3i | SAH | A | 801 |
| 1l3w | NDG | A | 804 |
| 1l4f | 150 | A | 990 |
| 1l4g | MCT | A | 990 |
| 1l4h | IND | A | 990 |
| 1l4k | 34A | A | 990 |
| 1l4l | XYD | A | 990 |
| 1l4m | 2AC | A | 990 |
| 1l4n | 2AF | A | 990 |
| 1l4x | SIN | A | 0 |
| 1l5f | BZI | A | 990 |
| 1l5k | 1RB | A | 990 |
| 1l5l | 7RP | A | 990 |
| 1l5m | 7RA | A | 990 |
| 1l5o | 2MP | A | 990 |
| 1l5o | NCN | A | 991 |
| 1l5r | 700 | A | 862 |
| 1l5s | URC | A | 863 |
| 1l5v | G1P | A | 900 |
| 1l6f | PP3 | A | 390 |
| 1l7f | BCZ | A | 801 |
| 1l7v | V4O | C | 250 |
| 1l7z | MYR | B | 200 |
| 1l8a | TDP | A | 887 |
| 1l8d | CIT | A | 507 |
| 1l8g | DBD | A | 322 |
| 1l8l | APO | A | 800 |
| 1l8n | GCW | A | 701 |
| 1l8s | LPE | B | 322 |
| 1l9d | PYC | A6401 |  |
| 1l9l | MPO | A | 111 |
| 1l9n | BGL | A1015 |  |
| 1l9w | DHS | A | 301 |
| 1lbc | CYZ | A | 330 |
| 1lbf | 137 | A | 300 |
| 1lbz | FBP | A | 295 |
| 1lc8 | 33P | A | 800 |
| 1lco | PPY | A | 580 |
| 1lco | PPY | B | 580 |
| 1lcp | PLU | A | 500 |
| 1lcs | BMA | B | 704 |
| 1lcw | SHM | A | 401 |
| 1lcz | BH7 | A | 401 |
| 1ld7 | U66 | B1003 |  |
| 1ld8 | FPP | B1002 |  |
| 1ld8 | U49 | B1003 |  |
| 1lde | FPI | A | 378 |
| 1ldg | OXM | A | 402 |
| 1ldo | SNR | A | 150 |
| 1lee | R36 | A | 500 |
| 1les | FRU | A | 206 |
| 1lf2 | R37 | A | 330 |
| 1lf3 | EH5 | A | 332 |
| 1lf7 | CIT | A | 200 |
| 1lfo | BEO | A | 130 |
| 1lfw | AEP | A | 683 |
| 1lgh | DET | A | 75 |
| 1lgt | BP3 | A | 300 |
| 1lgw | 1AN | A | 401 |
| 1lgx | 5AN | A | 401 |
| 1lhd | DI2 | H | 400 |
| 1lhf | DI4 | H | 400 |
| 1lhg | DI5 | H | 400 |
| 1lhn | AON | A | 301 |
| 1lho | AOM | A | 301 |
| 1li3 | 3CH | A | 401 |
| 1li6 | 5MP | A | 400 |
| 1lic | HDS | A | 133 |
| 1lij | RPP | A | 699 |
| 1lin | TFP | A | 153 |
| 1ljn | CBS | A | 130 |
| 1ljt | HDI | A | 115 |
| 1lk7 | DER | A1001 |  |
| 1lkd | BP6 | A | 300 |
| 1lke | DOG | A | 500 |
| 1ll9 | AXL | B | 964 |
| 1llf | F23 | A | 800 |
| 1llr | FNG | D | 104 |
| 1llr | LNQ | D | 105 |
| 1ln1 | DLP | A2313 |  |
| 1ln3 | CPL | A | 300 |
| 1lnm | DTX | A | 700 |
| 1lnz | G4P | B | 600 |
| 1lo0 | BC1 | H | 501 |
| 1lo2 | OX1 | H | 501 |
| 1lo3 | AN1 | H | 501 |
| 1lo7 | 4CO | A | 170 |
| 1lo8 | 4CA | A | 170 |
| 1lo9 | BCA | A | 170 |
| 1loj | URI | B9007 |  |
| 1lol | BU2 | A5001 |  |
| 1lov | 3GP | A | 106 |
| 1lox | RS7 | A | 841 |
| 1lpa | PLC | B | 452 |
| 1lpc | CMP | A | 901 |
| 1lpg | IMA | B | 301 |
| 1lpn | DSC | A | 560 |
| 1lq8 | MAN | G | 6 |
| 1lq8 | NDG | A | 1 |
| 1lqf | BGD | A | 802 |
| 1lqp | FCN | A4002 |  |
| 1lqt | FAD | A2457 |  |
| 1lqt | ODP | A2458 |  |
| 1lqu | NDP | A1458 |  |
| 1lr5 | MAN | A | 184 |
| 1lr8 | IHS | A | 30 |
| 1lrh | NLA | A5190 |  |
| 1ls3 | TGF | B | 420 |
| 1lsl | FUL | A1432 |  |
| 1lsx | 1MZ | A | 111 |
| 1lt5 | TDG | D | 104 |
| 1lt8 | CBH | A | 601 |
| 1lt8 | CIT | A | 701 |
| 1ltm | BCN | A | 401 |
| 1ltz | HBI | A | 500 |
| 1lu1 | ADE | A | 601 |
| 1lug | MBO | A1205 |  |
| 1lug | SUA | A1002 |  |
| 1lv2 | PLM | A | 328 |
| 1lvu | 9PP | A6100 |  |
| 1lvw | TYD | A3001 |  |
| 1lw5 | PLG | B1000 |  |
| 1lwj | ACG | A | 989 |
| 1lwx | AZD | A | 156 |
| 1lx6 | ZAM | A | 502 |
| 1lxc | AYM | A | 302 |
| 1lxk | BDP | A1001 |  |
| 1lxk | BDP | A1003 |  |
| 1lzj | BHG | A | 452 |
| 1lzy | NDG | A | 131 |
| 1lzz | IHG | A | 830 |
| 1m00 | BHH | A | 831 |
| 1m0s | CIT | A | 501 |
| 1m0w | 3GC | A | 501 |
| 1m0w | ANP | A | 504 |
| 1m13 | HYF | A | 435 |
| 1m15 | ADP | A | 400 |
| 1m17 | AQ4 | A | 999 |
| 1m1b | SPV | A | 996 |
| 1m26 | A2G | A | 135 |
| 1m2k | APR | A1001 |  |
| 1m2p | HNA | A | 351 |
| 1m2x | MCO | A | 811 |
| 1m2z | DEX | A | 301 |
| 1m32 | POA | A4601 |  |
| 1m3u | KPL | A | 265 |
| 1m3y | NDG | A | 438 |
| 1m40 | CB4 | A | 300 |
| 1m48 | FRG | A | 301 |
| 1m4a | MPE | A | 201 |
| 1m4b | NMP | A | 201 |
| 1m4d | TOY | A | 500 |
| 1m4i | PAP | A | 602 |
| 1m4n | AAD | A | 502 |
| 1m51 | TSX | A1001 |  |
| 1m52 | P17 | A | 119 |
| 1m56 | PEH | A2009 |  |
| 1m56 | PEH | A2012 |  |
| 1m56 | PEH | C2008 |  |
| 1m56 | PEH | C2010 |  |
| 1m56 | PEH | C2013 |  |
| 1m5w | DXP | A1001 |  |
| 1m66 | BCP | A | 401 |
| 1m66 | PLM | A | 402 |
| 1m67 | BOA | A | 402 |
| 1m6b | NDG | B | 626 |
| 1m6d | MYP | A1283 |  |
| 1m6e | SAL | X2000 |  |
| 1m6p | M6P | A | 200 |
| 1m6w | 12H | A4378 |  |
| 1m6y | SAH | A | 401 |
| 1m79 | MQ1 | A | 194 |
| 1m7a | MQU | A | 194 |
| 1m7g | ADP | B1304 |  |
| 1m7g | ADX | A1302 |  |
| 1m7g | AV2 | A1301 |  |
| 1m7p | G3H | A5401 |  |
| 1m7q | DQO | A | 501 |
| 1m7v | THG | A1999 |  |
| 1m7y | PPG | A | 600 |
| 1m8p | PPS | A | 574 |
| 1m98 | SUC | A | 401 |
| 1m9b | IBG | A | 220 |
| 1m9k | 7NI | A | 906 |
| 1m9m | 6NI | A | 906 |
| 1m9q | 5NI | A | 907 |
| 1mau | CIT | A1003 |  |
| 1mau | LTN | A | 390 |
| 1mb4 | NDP | A | 502 |
| 1mc0 | PCG | A | 160 |
| 1mc1 | PCX | A | 703 |
| 1mc5 | AHE | A | 404 |
| 1mcd | DPN | P | 7 |
| 1mcd | HIS | P | 6 |
| 1mcz | RMN | A | 534 |
| 1md2 | 233 | D2201 |  |
| 1mdb | DBH | A | 601 |
| 1mdl | RMN | A | 398 |
| 1mdp | MAL | 1 | 371 |
| 1me3 | P10 | A | 300 |
| 1me4 | T10 | A | 300 |
| 1me8 | RVP | A | 602 |
| 1mer | DMQ | A | 450 |
| 1mez | 2SA | A1455 |  |
| 1mfa | ABE | H | 503 |
| 1mfi | FHC | A | 115 |
| 1mfp | IDN | A | 302 |
| 1mfu | HMC | A | 599 |
| 1mg0 | DFB | A | 378 |
| 1mgo | PFB | B | 600 |
| 1mgo | PFB | B | 601 |
| 1mh5 | HAL | B | 601 |
| 1miw | ATP | A5501 |  |
| 1miy | CTP | A5501 |  |
| 1mj3 | HXC | A | 951 |
| 1mj4 | HEM | A | 502 |
| 1mjt | SUC | A | 601 |
| 1mk0 | CIT | A | 300 |
| 1mkv | GEL | A | 150 |
| 1ml2 | ZEM | A | 296 |
| 1ml3 | CYX | B1166 |  |
| 1ml4 | PAL | A | 385 |
| 1ml7 | HEV | A | 185 |
| 1ml7 | PYZ | A | 186 |
| 1mld | CIT | A | 375 |
| 1mly | ACZ | A | 731 |
| 1mmk | TIH | A | 428 |
| 1mmp | RSS | A | 269 |
| 1mmq | RRS | A | 269 |
| 1mmu | BGC | A | 348 |
| 1mmv | 3AR | A1785 |  |
| 1mmw | VIO | A1780 |  |
| 1mmy | G6D | A | 348 |
| 1mn0 | XYS | A | 348 |
| 1mn9 | RTP | A | 160 |
| 1mo9 | FAD | A1013 |  |
| 1mo9 | KPC | A1015 |  |
| 1moo | 4MZ | A | 264 |
| 1moq | GLP | A | 609 |
| 1mor | G6P | A | 609 |
| 1mqd | SHI | A3001 |  |
| 1mqi | FWD | A | 401 |
| 1mqj | HWD | A | 301 |
| 1mql | NDG | A | 334 |
| 1mql | NDG | B | 223 |
| 1mqo | CIT | A | 300 |
| 1mr2 | A12 | A | 301 |
| 1mr3 | G3D | F | 800 |
| 1mrl | DOL | A | 300 |
| 1mrs | 5HU | A | 217 |
| 1mrz | CIT | A | 295 |
| 1msk | SAM | A1301 |  |
| 1muq | TDG | B | 302 |
| 1muu | SUC | C1009 |  |
| 1mv9 | HXA | A | 200 |
| 1mvl | FMN | A1001 |  |
| 1mvn | PCO | A1001 |  |
| 1mvt | DTM | A | 187 |
| 1mwh | GTG | A1425 |  |
| 1mx5 | HTQ | A | 111 |
| 1mx9 | NLX | A | 1 |
| 1mxb | ADP | A | 385 |
| 1mxc | ABP | A | 385 |
| 1mxh | DHF | A1278 |  |
| 1my3 | BWD | A | 800 |
| 1my4 | IWD | A | 601 |
| 1mze | TAR | A | 500 |
| 1mzm | PLM | A | 201 |
| 1mzs | 669 | A | 350 |
| 1n08 | ADP | A | 301 |
| 1n0s | FLU | A | 500 |
| 1n0x | CXS | H1201 |  |
| 1n13 | PYR | B | 53 |
| 1n1d | C2G | A | 130 |
| 1n1g | BCP | A | 402 |
| 1n1m | A3M | A | 954 |
| 1n1p | FAD | A | 510 |
| 1n1t | DAN | A | 700 |
| 1n20 | 3AG | A | 900 |
| 1n22 | 7A8 | A | 801 |
| 1n23 | 2BN | A | 801 |
| 1n26 | NDG | A | 636 |
| 1n2e | APC | A | 801 |
| 1n2i | PAJ | A1001 |  |
| 1n2x | SAM | A | 401 |
| 1n2z | CNC | A | 400 |
| 1n3q | BDF | B | 254 |
| 1n46 | PFA | A | 462 |
| 1n4f | ASR | A | 140 |
| 1n4g | PYZ | A | 600 |
| 1n4k | I3P | A1000 |  |
| 1n4u | FAE | A | 510 |
| 1n52 | GTG | B2137 |  |
| 1n5j | TTP | A | 219 |
| 1n5s | ADL | B1001 |  |
| 1n5t | OAL | B1001 |  |
| 1n5u | HEM | A | 605 |
| 1n5u | MYR | A1002 |  |
| 1n62 | FAD | C3932 |  |
| 1n62 | MCN | B3920 |  |
| 1n69 | PEH | A | 300 |
| 1n6a | SAM | A | 402 |
| 1n6b | DMZ | A | 501 |
| 1n6d | D10 | G2101 |  |
| 1n6d | D10 | J2101 |  |
| 1n7i | LY1 | A3002 |  |
| 1n7j | IDI | A3003 |  |
| 1n7m | MMP | L | 600 |
| 1n7q | GTR | A1005 |  |
| 1n8k | PZO | A | 378 |
| 1n8q | DHB | A | 859 |
| 1n8v | BDD | A | 501 |
| 1n92 | PYZ | A | 378 |
| 1n95 | HFP | B | 501 |
| 1n9b | MA4 | A | 300 |
| 1na3 | IPT | A | 201 |
| 1naa | ABL | A | 901 |
| 1naq | MBO | A | 987 |
| 1nas | ASE | A | 801 |
| 1nav | IH5 | A | 600 |
| 1nbe | MLT | A | 311 |
| 1nbp | MHC | A | 201 |
| 1nc1 | MTH | A | 233 |
| 1nco | CHR | B | 114 |
| 1ncr | MYR | D4000 |  |
| 1nd0 | DP4 | A | 401 |
| 1nd2 | MYR | A6001 |  |
| 1nd2 | MYR | D4000 |  |
| 1nd5 | 2BF | A6000 |  |
| 1ndv | FR0 | A1001 |  |
| 1ndw | FR2 | A1001 |  |
| 1ndy | FR3 | A1001 |  |
| 1ne4 | RP1 | A | 401 |
| 1nek | OAA | A | 589 |
| 1nen | DNT | C | 306 |
| 1ney | 13P | A5001 |  |
| 1nf5 | BGC | B | 403 |
| 1nf8 | ISC | A | 220 |
| 1nfp | FMN | A | 229 |
| 1nfp | MYR | A | 424 |
| 1nfs | DED | A | 301 |
| 1nfu | RRP | A | 301 |
| 1nfv | 3PY | D1310 |  |
| 1nfw | RRR | A | 301 |
| 1nfy | RTR | A | 301 |
| 1ngp | NPA | H | 302 |
| 1ngs | E4P | A | 900 |
| 1nh8 | HIS | A | 289 |
| 1nhc | BMA | B | 405 |
| 1nhk | CMP | L | 146 |
| 1nhu | 153 | A5001 |  |
| 1nhv | 154 | A5001 |  |
| 1nhx | FTB | A | 704 |
| 1nhz | 486 | A | 800 |
| 1ni1 | 2C5 | B | 10 |
| 1niv | MDM | A | 110 |
| 1nj1 | 5CA | A | 512 |
| 1nj5 | P5A | A | 510 |
| 1nj6 | A5A | A | 508 |
| 1njj | GET | A | 601 |
| 1njs | KEU | A | 510 |
| 1nkz | BCL | A | 301 |
| 1nl2 | LPS | A | 451 |
| 1nl9 | 989 | A | 322 |
| 1nlm | UD1 | A | 401 |
| 1nlu | IVA | B | 1 |
| 1nmb | MAN | N | 474 |
| 1nme | 159 | B9002 |  |
| 1nml | CIT | A | 501 |
| 1nms | 161 | A1278 |  |
| 1nmy | FDM | A | 301 |
| 1nmz | NYM | A | 301 |
| 1nn3 | ADP | A | 302 |
| 1nn5 | 2DT | A | 301 |
| 1nn5 | ANP | A | 303 |
| 1nnf | EDT | A | 400 |
| 1nni | FMN | 1 | 200 |
| 1no3 | 4NC | A | 859 |
| 1no6 | 794 | A | 322 |
| 1no9 | 4ND | H | 250 |
| 1noj | NTZ | A | 998 |
| 1npa | 3NH | A | 100 |
| 1nqo | G3H | A5337 |  |
| 1nqt | ADP | A | 1 |
| 1nqu | RDL | A1201 |  |
| 1nqv | LMZ | A1201 |  |
| 1nqx | RLP | A1201 |  |
| 1nr6 | DIF | A | 501 |
| 1nt4 | G1P | A2000 |  |
| 1nth | BGX | A | 501 |
| 1nu1 | QNO | C | 383 |
| 1nu2 | I3P | A | 200 |
| 1nu3 | VPR | A4001 |  |
| 1nu4 | MLA | A | 402 |
| 1nup | NMN | A | 401 |
| 1nuq | DND | A | 401 |
| 1nut | APC | A | 401 |
| 1nuw | F6P | A2336 |  |
| 1nv8 | SAM | A | 300 |
| 1nv9 | SAH | A | 300 |
| 1nva | ADP | A1400 |  |
| 1nvq | UCN | A | 400 |
| 1nvs | UCM | A | 400 |
| 1nw6 | SFG | A | 401 |
| 1nw7 | SAH | A | 401 |
| 1nwl | 964 | A | 501 |
| 1nww | HPN | A | 900 |
| 1nwz | HC4 | A | 169 |
| 1nx8 | AKG | A | 280 |
| 1nxc | MAN | A | 8 |
| 1nxj | GLV | A | 741 |
| 1ny5 | ADP | A | 604 |
| 1nyt | DTV | A1405 |  |
| 1nyw | DAU | A9001 |  |
| 1nyx | DRF | A | 101 |
| 1nzc | TDX | A9002 |  |
| 1nzy | BCA | A | 272 |
| 1o01 | CRD | B4512 |  |
| 1o08 | G16 | A | 501 |
| 1o0f | A3P | A1301 |  |
| 1o0h | ADP | A1125 |  |
| 1o0n | U3P | A1101 |  |
| 1o0o | A2P | A | 998 |
| 1o0s | TTN | A | 810 |
| 1o1r | GRG | B2001 |  |
| 1o23 | GUD | B | 809 |
| 1o26 | FAD | A | 615 |
| 1o26 | UMP | A | 603 |
| 1o2h | CR3 | A | 246 |
| 1o2i | 655 | A | 246 |
| 1o2k | 656 | A | 246 |
| 1o2n | 762 | A | 246 |
| 1o2o | 950 | A | 246 |
| 1o2p | 972 | A | 246 |
| 1o2q | 991 | A | 246 |
| 1o2r | CR9 | A | 246 |
| 1o2s | CR4 | A | 246 |
| 1o2t | 783 | A | 246 |
| 1o2w | 847 | A | 246 |
| 1o2z | 312 | A | 246 |
| 1o30 | 693 | A | 246 |
| 1o33 | 801 | A | 246 |
| 1o35 | 802 | A | 246 |
| 1o38 | 653 | A | 246 |
| 1o3d | 780 | A | 246 |
| 1o3f | 696 | A | 246 |
| 1o3i | 907 | A | 246 |
| 1o3j | 334 | A | 246 |
| 1o3m | 785 | A | 246 |
| 1o42 | 843 | A | 300 |
| 1o46 | 903 | A | 300 |
| 1o47 | 822 | A | 300 |
| 1o48 | 853 | A | 300 |
| 1o49 | 493 | A | 300 |
| 1o4b | 876 | A | 300 |
| 1o4f | 790 | A | 300 |
| 1o4g | I59 | A | 300 |
| 1o4h | 772 | A | 300 |
| 1o4j | IS2 | A | 300 |
| 1o4l | CIT | A | 300 |
| 1o4m | MLA | A | 300 |
| 1o4n | OXD | A | 109 |
| 1o4o | HPS | A | 300 |
| 1o4p | 791 | A | 300 |
| 1o4q | 256 | A | 300 |
| 1o4r | 787 | A | 300 |
| 1o51 | ADP | A | 200 |
| 1o5r | FR9 | A1001 |  |
| 1o5w | MLG | A | 709 |
| 1o5x | 2PG | B4400 |  |
| 1o5x | 3PY | A3300 |  |
| 1o68 | KIV | A | 275 |
| 1o6b | ADP | A | 173 |
| 1o6g | SIN | A | 724 |
| 1o6q | R17 | A | 800 |
| 1o6r | R19 | A | 800 |
| 1o6u | PLM | A1398 |  |
| 1o79 | R23 | A | 800 |
| 1o7d | BMA | C | 3 |
| 1o7d | MAN | C | 167 |
| 1o7d | NDG | C | 1 |
| 1o7n | IND | A1452 |  |
| 1o7p | NDH | A1450 |  |
| 1o7q | UDP | A1374 |  |
| 1o84 | D10 | A1074 |  |
| 1o84 | MAL | A1073 |  |
| 1o8b | ABF | A1269 |  |
| 1o94 | ADP | A1731 |  |
| 1o97 | FAD | D1319 |  |
| 1o98 | 2PG | A | 801 |
| 1o9l | EMT | A9007 |  |
| 1o9o | MLM | A | 501 |
| 1o9p | MLA | A | 501 |
| 1o9t | ATP | B1397 |  |
| 1oaa | OAA | A | 1 |
| 1oad | MQD | A | 395 |
| 1oau | DNF | H | 501 |
| 1obb | MAL | A | 900 |
| 1obc | 2AD | A1816 |  |
| 1obd | ATP | A | 307 |
| 1obh | LMS | A1819 |  |
| 1obo | FMN | A1170 |  |
| 1oc2 | TDX | A1349 |  |
| 1oc7 | DMF | A | 503 |
| 1oc7 | MA3 | A | 605 |
| 1ocb | FLG | B | 460 |
| 1ocn | IFM | A | 452 |
| 1ocq | IFM | A | 500 |
| 1ocy | CIT | A1528 |  |
| 1od2 | ADE | B3190 |  |
| 1od3 | BGC | A1151 |  |
| 1od6 | PNS | A1161 |  |
| 1odc | A8B | A1538 |  |
| 1odm | ASV | A1332 |  |
| 1odu | FUL | A1448 |  |
| 1odz | MAN | A1425 |  |
| 1oec | AA2 | A | 789 |
| 1of1 | SCT | A | 400 |
| 1of8 | G3P | A1371 |  |
| 1of8 | PEP | A1370 |  |
| 1ofd | AKG | A3510 |  |
| 1ofe | ONL | B2511 |  |
| 1ofl | NGK | A | 526 |
| 1ofo | PGA | A | 442 |
| 1ofs | SUC | A1184 |  |
| 1ofz | FUL | A1313 |  |
| 1og5 | HEC | A | 501 |
| 1og5 | SWF | A | 502 |
| 1ogd | RIP | A1134 |  |
| 1oge | RP5 | A1133 |  |
| 1ogk | DUD | B1280 |  |
| 1ogo | BGC | X1575 |  |
| 1ogp | MTQ | A1394 |  |
| 1ogs | NDG | A1997 |  |
| 1ogu | ST8 | A1298 |  |
| 1oh0 | EQU | A1128 |  |
| 1ohp | ESR | A1126 |  |
| 1oi6 | TMP | A1204 |  |
| 1oi9 | N20 | A1298 |  |
| 1oif | IFM | A1447 |  |
| 1oik | AKG | A1301 |  |
| 1oik | C26 | A1300 |  |
| 1oiq | HDU | A1299 |  |
| 1oir | HDY | A1298 |  |
| 1oit | HDT | A1299 |  |
| 1oiu | N76 | A1298 |  |
| 1oiy | N41 | A1298 |  |
| 1oj1 | CG2 | A1106 |  |
| 1oj4 | ANP | A1285 |  |
| 1oj7 | NZQ | A1388 |  |
| 1oj9 | 1PB | A1503 |  |
| 1oja | ISN | A | 601 |
| 1ojj | BGC | A1400 |  |
| 1ojn | NG6 | A | 901 |
| 1ojr | 2HA | A1277 |  |
| 1okc | CXT | A | 401 |
| 1okc | LDM | A | 903 |
| 1okm | SAB | A | 555 |
| 1okn | STB | A | 555 |
| 1olt | SAM | A | 501 |
| 1om1 | IQA | A | 338 |
| 1omz | UD2 | A | 405 |
| 1on3 | DXX | A5001 |  |
| 1on3 | MCA | A4001 |  |
| 1on6 | UD1 | A | 401 |
| 1on8 | BDP | A | 509 |
| 1on8 | UDP | A | 351 |
| 1onp | FOM | A1001 |  |
| 1onz | 968 | A | 322 |
| 1oo6 | SN2 | A | 219 |
| 1ooh | 1BO | A | 600 |
| 1oon | BEL | A | 362 |
| 1oop | MYR | D | 70 |
| 1ooq | DTC | B | 222 |
| 1opj | MYR | A | 1 |
| 1opk | P16 | A | 2 |
| 1opm | IYG | A | 801 |
| 1oqm | UD2 | B | 404 |
| 1ork | ATC | A | 222 |
| 1os1 | PYR | A | 542 |
| 1os5 | NH1 | A | 901 |
| 1os6 | DXC | A | 75 |
| 1osf | KOS | A | 280 |
| 1osn | BVP | A | 500 |
| 1ot7 | IU5 | B1002 |  |
| 1oth | PAO | A | 355 |
| 1otj | TAU | A | 952 |
| 1oty | 6MP | A | 247 |
| 1ou6 | 168 | A5001 |  |
| 1ouk | 084 | A | 501 |
| 1oum | TAL | A | 245 |
| 1ouw | MLT | C | 502 |
| 1ov5 | 2LP | A | 403 |
| 1ov6 | DBM | A | 245 |
| 1ov7 | LYL | A | 407 |
| 1ove | 358 | A | 401 |
| 1ovg | MDR | A | 245 |
| 1ovh | 2CM | A | 404 |
| 1ovj | FLM | A | 408 |
| 1ovk | NYL | A | 405 |
| 1ovp | BDF | A | 115 |
| 1ow4 | 2AN | A1004 |  |
| 1owd | 497 | A1001 |  |
| 1owe | 675 | A1001 |  |
| 1owh | 239 | A1001 |  |
| 1owi | 426 | A | 245 |
| 1owj | 155 | A | 245 |
| 1owk | 303 | A | 245 |
| 1owz | 4FA | A | 409 |
| 1oxc | FUL | A | 115 |
| 1oxl | IDA | A | 501 |
| 1oxo | IK2 | A | 411 |
| 1oyo | 3ID | A | 694 |
| 1oyt | FSN | H | 501 |
| 1oz0 | MS1 | A1002 |  |
| 1oz1 | FPH | A | 500 |
| 1ozg | HE3 | A | 700 |
| 1ozh | HE3 | A1406 |  |
| 1ozn | NDG | A | 401 |
| 1p0b | PQ0 | A | 900 |
| 1p0i | BUA | A | 606 |
| 1p0p | BCH | A | 604 |
| 1p1o | QUS | A | 301 |
| 1p1r | NMH | A | 378 |
| 1p28 | HBR | A1002 |  |
| 1p28 | HBS | A1001 |  |
| 1p2a | 5BN | A | 301 |
| 1p2y | NCT | A | 440 |
| 1p3d | ANP | A | 603 |
| 1p3y | FAD | 1 | 259 |
| 1p42 | MYR | A | 601 |
| 1p44 | GEQ | A | 350 |
| 1p4f | DRG | A | 295 |
| 1p4g | CGF | A | 998 |
| 1p4h | CR6 | A | 998 |
| 1p4j | CBF | A | 998 |
| 1p4m | FMN | A | 401 |
| 1p5d | G1P | X | 658 |
| 1p5g | G6P | X | 658 |
| 1p60 | DCZ | A | 402 |
| 1p62 | GEO | B | 302 |
| 1p6b | EBP | A | 7 |
| 1p6d | 3PC | A | 601 |
| 1p6e | PC5 | A | 601 |
| 1p6i | DP3 | A | 793 |
| 1p6o | HPY | A | 411 |
| 1p6w | GTM | A5003 |  |
| 1p6w | SGC | A5001 |  |
| 1p6w | SGC | A6001 |  |
| 1p6x | THM | A | 701 |
| 1p77 | ATR | A | 300 |
| 1p7l | ANP | A | 384 |
| 1p7p | MPH | A | 553 |
| 1p7t | PYR | A | 810 |
| 1p84 | DBT | C | 705 |
| 1p8j | DKA | J | 801 |
| 1p8j | DKA | K | 801 |
| 1p91 | SAM | A1401 |  |
| 1p9b | HDA | A1602 |  |
| 1p9b | IMO | A1601 |  |
| 1pax | DHQ | A | 200 |
| 1pbf | BHA | A | 396 |
| 1pbq | DK1 | A1001 |  |
| 1pca | CIT | A | 600 |
| 1pcj | M1P | X | 600 |
| 1pck | PEZ | A1268 |  |
| 1pcm | M6P | X | 600 |
| 1pcw | H4P | A1268 |  |
| 1pdz | PGA | A | 439 |
| 1pe1 | 2PG | A1268 |  |
| 1pe7 | 4BR | A | 322 |
| 1pea | ACM | A | 386 |
| 1peo | DCP | A | 716 |
| 1pf8 | SU9 | A | 500 |
| 1pfk | ADP | A | 324 |
| 1pfu | MPJ | A | 553 |
| 1pfv | 2FM | A | 553 |
| 1pfw | MF3 | A | 553 |
| 1pfy | MSP | A | 553 |
| 1pge | ISF | A | 800 |
| 1pgf | IMM | A | 800 |
| 1pgn | NBP | A | 499 |
| 1pgp | 6PG | A | 502 |
| 1ph0 | 418 | A | 322 |
| 1phb | PFZ | A | 422 |
| 1phg | MYT | A | 422 |
| 1phh | DHB | A | 396 |
| 1phq | FPE | A | 301 |
| 1piv | MYR | 4 | 1 |
| 1piv | W71 | 1 | 303 |
| 1pj2 | MLT | A | 701 |
| 1pj3 | PYR | A | 603 |
| 1pj6 | FOL | A2887 |  |
| 1pjx | DXE | A | 441 |
| 1pk8 | ATP | A | 800 |
| 1pk9 | 2FA | A | 306 |
| 1pke | 2FD | A | 446 |
| 1pkf | EPD | A | 450 |
| 1pkk | DCP | B | 293 |
| 1pkx | XMP | A1901 |  |
| 1pl1 | ABY | A | 654 |
| 1pl6 | 572 | A | 405 |
| 1pmn | 984 | A | 501 |
| 1pmq | 880 | A | 501 |
| 1pmu | 9HP | A | 501 |
| 1pmv | 537 | A | 501 |
| 1pn0 | FAD | A6011 |  |
| 1pn4 | HDC | A1277 |  |
| 1pnf | NDG | A | 431 |
| 1pnq | NDP | A | 500 |
| 1po1 | J80 | 1 | 0 |
| 1po1 | MYR | 4 | 1 |
| 1po2 | J77 | 1 | 0 |
| 1po2 | MYR | 4 | 1 |
| 1po8 | SHV | A | 122 |
| 1poc | GEL | A | 420 |
| 1pot | SPD | A | 350 |
| 1pov | MYR | 0 | 1 |
| 1pov | SPH | 1 | 0 |
| 1poy | SPD | 1 | 400 |
| 1pp9 | BHG | A4002 |  |
| 1pp9 | BHG | C2008 |  |
| 1pp9 | BHG | F3011 |  |
| 1pp9 | BHG | F4001 |  |
| 1pp9 | PEE | A4003 |  |
| 1pp9 | PEE | C2007 |  |
| 1pp9 | PEE | D2006 |  |
| 1pp9 | PEE | G2005 |  |
| 1ppa | ANL | A | 134 |
| 1ppg | MAN | E | 418 |
| 1ppj | BHG | C2010 |  |
| 1ppj | BHG | C4002 |  |
| 1ppj | BHG | D4003 |  |
| 1ppj | BHG | R4007 |  |
| 1ppj | PEE | Q3006 |  |
| 1pq6 | 965 | A1500 |  |
| 1pq9 | 44B | A1501 |  |
| 1pqh | MLA | A | 390 |
| 1pqp | HSE | A | 372 |
| 1pr4 | MTP | A1245 |  |
| 1pr6 | XYA | A1245 |  |
| 1pro | A88 | A | 301 |
| 1ps3 | KIF | A1501 |  |
| 1ps6 | 4TP | A | 332 |
| 1ps9 | MDE | A | 704 |
| 1pt2 | SUC | A | 501 |
| 1pt9 | TAP | A | 300 |
| 1pth | SAL | A | 710 |
| 1ptj | SND | A | 500 |
| 1pu7 | 39A | A | 219 |
| 1pu8 | EA1 | A | 219 |
| 1pv7 | TDG | A | 500 |
| 1pvc | MYR | 4 | 1 |
| 1pvc | SPH | 11000 |  |
| 1pvg | ANP | A | 901 |
| 1pvn | MZP | A | 971 |
| 1pvs | 7HP | A | 800 |
| 1pw1 | HEL | A | 400 |
| 1pw5 | NDG | A | 254 |
| 1pw6 | FRB | A | 201 |
| 1pw7 | RAB | A | 645 |
| 1pwl | BFI | A | 320 |
| 1pwp | NSC | A9002 |  |
| 1pwp | NSC | B9003 |  |
| 1pwq | SD2 | A9002 |  |
| 1pwu | GM6 | A1001 |  |
| 1pwy | AC2 | E | 290 |
| 1pwz | RSO | A | 256 |
| 1px0 | RPN | A1001 |  |
| 1px4 | IPT | A2001 |  |
| 1pxd | SFP | A | 134 |
| 1pxh | SNA | A | 401 |
| 1pxi | CK1 | A | 500 |
| 1pxk | CK3 | A | 500 |
| 1pxl | CK4 | A | 500 |
| 1pxm | CK5 | A | 500 |
| 1pxn | CK6 | A | 500 |
| 1pxo | CK7 | A | 500 |
| 1pxx | DIF | A | 701 |
| 1py0 | YMA | A | 195 |
| 1py2 | FRH | A | 201 |
| 1py5 | PY1 | A | 700 |
| 1pyd | TDP | A | 557 |
| 1pye | PM1 | A | 700 |
| 1pyh | BCL | 1 | 38 |
| 1pyh | BCL | A | 301 |
| 1pz4 | PLM | A | 200 |
| 1pzg | A3D | A | 601 |
| 1pzi | 1DM | D | 104 |
| 1pzj | 15B | D | 104 |
| 1pzj | J15 | E | 105 |
| 1pzk | J12 | D | 105 |
| 1pzl | MYR | A | 500 |
| 1pzo | CBT | A | 300 |
| 1pzp | FTA | A | 300 |
| 1q0h | CIT | A | 402 |
| 1q0q | DXP | A | 701 |
| 1q0r | AKT | A | 600 |
| 1q0s | SAH | A | 300 |
| 1q0z | AKA | A | 600 |
| 1q16 | HEM | C | 806 |
| 1q16 | HEM | C | 807 |
| 1q19 | SSC | A | 506 |
| 1q1a | OAD | A1001 |  |
| 1q1g | MTI | A | 301 |
| 1q1m | 234 | A | 322 |
| 1q20 | PLO | A | 401 |
| 1q23 | FUA | A | 702 |
| 1q2e | SGC | A | 902 |
| 1q2o | DP1 | A | 790 |
| 1q33 | BGC | A | 400 |
| 1q36 | SKP | A | 600 |
| 1q3e | PCG | A | 401 |
| 1q3g | UDA | A1450 |  |
| 1q3q | ANP | A1528 |  |
| 1q3s | ADP | A1528 |  |
| 1q3w | ATU | A | 502 |
| 1q41 | IXM | A | 451 |
| 1q44 | MLA | A | 901 |
| 1q4g | BFL | A | 701 |
| 1q4g | NDG | A | 672 |
| 1q4l | 679 | A | 451 |
| 1q4n | TAM | X | 900 |
| 1q4s | PHB | A | 272 |
| 1q4t | 4CO | A | 370 |
| 1q4u | 4CA | A | 370 |
| 1q4x | G24 | A | 462 |
| 1q5d | EPB | A | 450 |
| 1q5k | TMU | A | 901 |
| 1q65 | BHB | A | 900 |
| 1q66 | KMB | A | 900 |
| 1q6k | TCO | A | 0 |
| 1q6m | P27 | A | 301 |
| 1q6n | P90 | A | 801 |
| 1q6o | LG6 | A7301 |  |
| 1q6q | LXP | A7301 |  |
| 1q6r | LX1 | A7301 |  |
| 1q79 | 3AT | A1000 |  |
| 1q7a | OPB | A | 124 |
| 1q7g | NHO | A | 361 |
| 1q84 | TZ4 | A | 951 |
| 1q8a | HCS | A | 701 |
| 1q8j | C2F | A | 801 |
| 1q8m | GSH | A | 999 |
| 1q8t | Y27 | A | 930 |
| 1q8u | H52 | A | 961 |
| 1q91 | DPB | A | 1 |
| 1qan | SAH | A | 501 |
| 1qaq | SFG | A | 245 |
| 1qb6 | 623 | A | 300 |
| 1qb7 | ADE | A | 300 |
| 1qb7 | CIT | A | 325 |
| 1qb9 | 806 | A | 300 |
| 1qbo | 711 | A | 300 |
| 1qbu | 846 | B | 300 |
| 1qbv | PPX | H | 907 |
| 1qcf | PP1 | A | 532 |
| 1qcj | APT | B | 902 |
| 1qd0 | RR6 | A1003 |  |
| 1qd1 | FON | A | 330 |
| 1qdd | NDG | A | 601 |
| 1qf0 | TI2 | A | 317 |
| 1qf1 | TI1 | A | 317 |
| 1qf2 | TI3 | A | 317 |
| 1qf4 | RPD | A | 435 |
| 1qf5 | RPL | A | 435 |
| 1qfg | DDQ | A1100 |  |
| 1qfg | FTT | A1009 |  |
| 1qfg | KDO | A1002 |  |
| 1qfg | MYR | A1014 |  |
| 1qfm | SGL | A | 782 |
| 1qfm | SGM | A | 783 |
| 1qfm | SGM | A | 784 |
| 1qfo | SIA | A | 201 |
| 1qfz | NDP | A | 310 |
| 1qgf | TPX | A | 280 |
| 1qgj | GSH | A1794 |  |
| 1qgl | EJT | A | 238 |
| 1qgl | SIN | B | 241 |
| 1qgq | UDP | A | 311 |
| 1qhc | PUA | A | 901 |
| 1qhg | ATP | A | 726 |
| 1qhi | BPG | A | 1 |
| 1qho | MAL | A1201 |  |
| 1qho | MAL | A1202 |  |
| 1qhu | HEM | A | 500 |
| 1qhx | ATP | A | 501 |
| 1qi1 | G3P | B | 505 |
| 1qi3 | MTT | A | 460 |
| 1qin | GIP | A | 300 |
| 1qip | GNB | A1002 |  |
| 1qj3 | KAP | A1430 |  |
| 1qje | IP1 | A1333 |  |
| 1qjf | ACS | A1333 |  |
| 1qji | PKF | A1202 |  |
| 1qjq | PFC | A1022 |  |
| 1qju | W01 | 17001 |  |
| 1qjw | MAN | A | 503 |
| 1qjx | W02 | 17002 |  |
| 1qjy | W03 | 17003 |  |
| 1qk0 | IOB | A | 901 |
| 1qk0 | XYS | B | 902 |
| 1qk3 | 5GP | A | 300 |
| 1qk4 | IMP | A | 300 |
| 1qk5 | XMP | A | 300 |
| 1qki | GOA | A | 900 |
| 1qki | GOA | C | 900 |
| 1qkm | GEN | A | 600 |
| 1qkn | RAL | A | 600 |
| 1qkq | BMA | A | 858 |
| 1qks | DHE | A | 602 |
| 1qll | TDA | A | 122 |
| 1qm4 | AMB | C | 397 |
| 1qm5 | SGC | A | 997 |
| 1qmg | APX | A | 600 |
| 1qmg | DMV | A | 620 |
| 1qmh | CIT | A | 401 |
| 1qmq | DRB | A | 419 |
| 1qmq | LRB | A | 418 |
| 1qnq | TPT | A | 401 |
| 1qnr | MAB | A | 401 |
| 1qo0 | BMD | A | 400 |
| 1qon | I40 | A | 997 |
| 1qop | IPL | A | 300 |
| 1qp8 | NDP | A | 310 |
| 1qpb | PYM | A | 602 |
| 1qpb | PYM | B | 602 |
| 1qpe | PP2 | A1904 |  |
| 1qpf | B7G | A | 109 |
| 1qpl | 587 | A | 108 |
| 1qpn | NCN | A2901 |  |
| 1qpq | NTM | A2901 |  |
| 1qpr | PHT | A | 1 |
| 1qpr | PPC | A | 288 |
| 1qqp | IDS | 1 | 703 |
| 1qqp | SGN | 1 | 704 |
| 1qqs | DKA | A | 181 |
| 1qrd | CBD | A | 275 |
| 1qrd | DQN | A | 276 |
| 1qs4 | 100 | A1004 |  |
| 1qsg | TCL | A1302 |  |
| 1qtn | DTD | A | 506 |
| 1qtn | DTD | B | 505 |
| 1qv0 | CZH | A | 201 |
| 1qv6 | 24B | A | 378 |
| 1qvj | RP5 | A | 554 |
| 1qvn | FRI | A | 201 |
| 1qw8 | KHO | A | 950 |
| 1qw9 | KHP | A1750 |  |
| 1qwj | NCC | A2002 |  |
| 1qx1 | FMF | A2003 |  |
| 1qxw | M1C | A3001 |  |
| 1qxy | M2C | A3001 |  |
| 1qxz | M3C | A2001 |  |
| 1qy2 | IPZ | A | 300 |
| 1qy4 | 6PG | A | 300 |
| 1qy5 | NEC | A | 338 |
| 1qy8 | RDI | A | 338 |
| 1qyx | ASD | A | 500 |
| 1qz5 | KAB | A | 500 |
| 1qz6 | JAS | A | 500 |
| 1qz6 | SUC | A | 600 |
| 1qzy | TDE | A | 500 |
| 1r08 | W42 | 1 | 900 |
| 1r09 | JEN | 11000 |  |
| 1r0c | NCD | A2001 |  |
| 1r0e | DFN | A | 702 |
| 1r0p | KSA | A | 0 |
| 1r16 | PYF | A | 400 |
| 1r1h | BIR | A2001 |  |
| 1r1i | TI1 | A2001 |  |
| 1r1j | OIR | A2001 |  |
| 1r1o | SDC | A1000 |  |
| 1r20 | HWG | D2000 |  |
| 1r30 | DTB | A | 502 |
| 1r31 | MEV | A1003 |  |
| 1r31 | MEV | B1004 |  |
| 1r33 | LKA | A1162 |  |
| 1r35 | I58 | A | 800 |
| 1r3n | BIB | A2502 |  |
| 1r3s | 1CP | A | 901 |
| 1r3t | CP3 | A | 867 |
| 1r4s | MUA | A | 800 |
| 1r4u | OXC | A | 999 |
| 1r55 | 097 | A | 518 |
| 1r58 | AO5 | A | 501 |
| 1r5g | AO1 | A | 501 |
| 1r5h | AO2 | A | 501 |
| 1r5y | DQU | A | 900 |
| 1r66 | TYD | A | 901 |
| 1r6a | RVP | A | 300 |
| 1r6d | DAU | A | 401 |
| 1r6g | 442 | A | 500 |
| 1r6n | 434 | A1111 |  |
| 1r6w | 164 | A | 735 |
| 1r78 | FMD | A | 299 |
| 1r7u | DLG | A | 452 |
| 1r81 | UD2 | A | 475 |
| 1r82 | AOG | A | 452 |
| 1r89 | CTP | A | 501 |
| 1r89 | CTP | A | 502 |
| 1r8b | ATP | A | 501 |
| 1r8b | ATP | A | 502 |
| 1r8c | UTP | A | 501 |
| 1r8c | UTP | A | 502 |
| 1r9e | PGO | A1802 |  |
| 1r9l | BET | A1001 |  |
| 1ra0 | FPY | A | 501 |
| 1ra8 | ATR | A | 164 |
| 1rb0 | HH2 | A | 181 |
| 1rc2 | BGL | B | 600 |
| 1rca | CGP | A | 150 |
| 1rcd | BET | A | 200 |
| 1rcv | BV1 | D | 104 |
| 1rd4 | L08 | A | 328 |
| 1rd5 | MLA | A | 302 |
| 1rd9 | BV2 | D | 104 |
| 1rdf | ESA | A | 500 |
| 1rdi | MFU | 1 | 1 |
| 1rdj | MFB | 1 | 1 |
| 1rdl | MMA | 1 | 1 |
| 1rdm | MMA | 1 | 1 |
| 1rdn | NDG | 1 | 1 |
| 1rdp | BV3 | D | 104 |
| 1rdq | ADP | E | 599 |
| 1rdq | ATP | E | 600 |
| 1re0 | CIT | A | 703 |
| 1re8 | BD2 | A | 351 |
| 1re9 | DSO | A | 601 |
| 1rej | B1L | A | 351 |
| 1rek | B8L | A | 351 |
| 1rem | BMA | A | 131 |
| 1reo | NDG | A | 490 |
| 1req | DCA | A | 801 |
| 1rf2 | BV4 | D | 104 |
| 1rf7 | DHF | A | 161 |
| 1rf9 | DBR | A | 900 |
| 1rft | ACP | A | 401 |
| 1rfu | ADP | A | 402 |
| 1rgb | ELD | B | 134 |
| 1rge | 2GP | A | 98 |
| 1rhc | F42 | A | 351 |
| 1ri1 | GTG | A | 300 |
| 1ri4 | SAM | A | 299 |
| 1rid | IDS | A | 245 |
| 1rid | SGN | A | 246 |
| 1rjd | SAM | A | 801 |
| 1rkd | ADP | A | 310 |
| 1rkd | RIB | A | 311 |
| 1rkg | VD1 | A | 500 |
| 1rkp | IBM | A | 201 |
| 1rkw | PNT | A | 225 |
| 1rl4 | BL5 | A | 501 |
| 1rl8 | RIT | A9001 |  |
| 1rl9 | IOM | A | 902 |
| 1rm0 | D6P | A1520 |  |
| 1rmy | DCZ | A | 807 |
| 1rmz | NGH | A | 269 |
| 1rnc | 5GP | A | 161 |
| 1ro9 | 8BR | A | 531 |
| 1rob | C2P | A | 126 |
| 1ros | DEO | A | 1 |
| 1rp0 | AHZ | A | 600 |
| 1rp0 | AHZ | B | 601 |
| 1rp9 | DAF | A3000 |  |
| 1rpa | TAR | A | 343 |
| 1rpg | CPA | A | 126 |
| 1rpq | CIT | C | 403 |
| 1rpw | DID | B | 193 |
| 1rq2 | CIT | A | 380 |
| 1rq5 | CTT | A | 820 |
| 1rqd | GC7 | A | 371 |
| 1rqe | OAA | A | 529 |
| 1rqh | PYR | A | 602 |
| 1rqj | IPR | A | 900 |
| 1rql | VSO | B | 600 |
| 1rqp | SAM | A | 500 |
| 1rqx | MLP | A | 501 |
| 1rr2 | 2KT | A | 530 |
| 1rri | A45 | A1001 |  |
| 1rrm | APR | A | 389 |
| 1rrm | PGO | A | 390 |
| 1rrv | BGC | C | 8 |
| 1rrw | 9MG | A1001 |  |
| 1rry | 204 | A1001 |  |
| 1rs2 | 209 | A1001 |  |
| 1rs4 | 45P | A1001 |  |
| 1rsd | PSB | A1001 |  |
| 1rsg | FAD | A1801 |  |
| 1rsi | 977 | A1001 |  |
| 1rsn | SGP | A | 97 |
| 1rt1 | MKC | A | 999 |
| 1rt5 | UC2 | A | 999 |
| 1rt6 | UC3 | A | 999 |
| 1rt7 | UC4 | A | 999 |
| 1rth | U05 | A | 999 |
| 1rti | HEF | A | 999 |
| 1ruc | W35 | 1 | 290 |
| 1rue | W35 | 1 | 290 |
| 1rug | W35 | 1 | 290 |
| 1rus | 3PG | A | 500 |
| 1ruv | UVC | A | 125 |
| 1rv0 | DAN | L | 701 |
| 1rv0 | NDG | H | 642 |
| 1rv1 | IMZ | A | 110 |
| 1rv6 | B3P | W | 301 |
| 1rvt | BGC | J | 805 |
| 1rvv | INI | 2 | 200 |
| 1rw8 | 580 | A | 501 |
| 1rwh | GCT | A | 811 |
| 1rwq | 5AP | A | 900 |
| 1rx0 | 2MC | C | 400 |
| 1rxc | URF | B2011 |  |
| 1rxg | AKG | A | 313 |
| 1rxi | LCP | A | 152 |
| 1rxs | DUR | B2012 |  |
| 1rxs | V7O | A5011 |  |
| 1rxu | THM | A2012 |  |
| 1ry0 | PG2 | A2325 |  |
| 1ryi | GOA | A9110 |  |
| 1rzh | BCL | L | 852 |
| 1rzl | CXS | A | 201 |
| 1rzu | ADP | A1531 |  |
| 1rzy | 5AS | A | 301 |
| 1s0i | SLT | A | 923 |
| 1s0j | MUS | A | 700 |
| 1s0x | C3S | A1001 |  |
| 1s0z | EB1 | A | 510 |
| 1s13 | 2FH | A | 300 |
| 1s17 | GNR | A | 501 |
| 1s19 | MC9 | A | 500 |
| 1s1d | GP2 | A4001 |  |
| 1s1f | MLA | A | 432 |
| 1s1j | IQZ | A | 301 |
| 1s1s | WAC | B | 301 |
| 1s1t | UC1 | A | 999 |
| 1s22 | ULA | A | 500 |
| 1s26 | APC | A1139 |  |
| 1s2c | FLF | A2001 |  |
| 1s2d | ADE | A1245 |  |
| 1s2i | BP1 | A1245 |  |
| 1s2n | PMS | A1284 |  |
| 1s38 | MAQ | A | 900 |
| 1s39 | AQO | A | 900 |
| 1s3b | RMA | A | 601 |
| 1s3f | SNI | A | 745 |
| 1s3v | TQD | A | 187 |
| 1s3w | TQT | A | 188 |
| 1s4p | MMA | A | 901 |
| 1s59 | DGT | C1102 |  |
| 1s5o | 152 | A | 617 |
| 1s5z | SON | A1160 |  |
| 1s63 | 778 | B3012 |  |
| 1s6p | IET | A | 701 |
| 1s6q | TPB | A | 701 |
| 1s6v | HEM | B | 104 |
| 1s7f | MLA | A | 999 |
| 1s83 | 4HA | A | 5 |
| 1s84 | DYB | A | 11 |
| 1s8c | BLA | A | 400 |
| 1s9a | TAM | A | 305 |
| 1s9d | AFB | A | 403 |
| 1s9e | ADB | A | 701 |
| 1s9g | ABZ | A | 701 |
| 1s9j | BBM | A1001 |  |
| 1s9q | CHD | A | 459 |
| 1sa0 | CN2 | B | 700 |
| 1sa0 | GTP | A | 600 |
| 1sa1 | POD | B | 700 |
| 1say | PYR | A | 501 |
| 1saz | ACP | A | 399 |
| 1sb8 | UD2 | A | 343 |
| 1sbr | VIB | A | 501 |
| 1sc9 | CNH | A | 300 |
| 1sd3 | SYM | A | 998 |
| 1sdd | NDG | B2189 |  |
| 1sdw | IYT | A | 701 |
| 1se3 | SIA | A | 400 |
| 1seg | PPI | A | 168 |
| 1sez | OMN | A | 601 |
| 1sff | IK2 | A | 450 |
| 1sg0 | FAD | A | 434 |
| 1sg0 | STL | A | 501 |
| 1sgj | OAA | A | 285 |
| 1sgj | OAA | B | 285 |
| 1shl | FXN | A | 401 |
| 1sir | NBC | A | 400 |
| 1sj0 | E4D | A | 600 |
| 1sj1 | NCO | A | 71 |
| 1sjd | NPG | A1200 |  |
| 1sjd | NPG | B1163 |  |
| 1sjw | NGV | A | 333 |
| 1sk6 | CMP | A | 289 |
| 1skb | NDG | A | 507 |
| 1skj | UR2 | A | 113 |
| 1sln | INH | A | 256 |
| 1smk | CIT | C1313 |  |
| 1smy | G4P | N9100 |  |
| 1snn | 5RP | A | 401 |
| 1so2 | 666 | A | 461 |
| 1so4 | TX4 | A1301 |  |
| 1sq5 | PAU | A6001 |  |
| 1sqa | UI1 | A1001 |  |
| 1sqb | AZO | C | 383 |
| 1sql | GUN | A1001 |  |
| 1sqn | NDR | A1001 |  |
| 1sqo | UI2 | A1001 |  |
| 1sqp | MYX | C | 383 |
| 1sqp | PEE | E | 197 |
| 1sqt | UI3 | A1001 |  |
| 1sqv | UHD | C | 383 |
| 1sr9 | KIV | A | 701 |
| 1srf | MTB | B | 300 |
| 1srg | MHB | A | 300 |
| 1sri | DMB | A | 300 |
| 1st0 | GTG | A | 651 |
| 1st4 | GTA | A | 451 |
| 1st8 | NDG | A | 650 |
| 1su2 | ATP | A | 357 |
| 1sui | FRE | A | 306 |
| 1suo | CPZ | A | 600 |
| 1suq | 185 | A | 600 |
| 1sus | SPF | A | 401 |
| 1sux | BTS | B | 401 |
| 1sv3 | ANN | A | 347 |
| 1sv5 | 65B | A | 600 |
| 1sv9 | DIF | A | 701 |
| 1svt | ADP | A | 600 |
| 1sww | POA | A | 600 |
| 1sx3 | ATP | A | 1 |
| 1sx4 | ADP | A | 600 |
| 1sx6 | OLA | A | 302 |
| 1sxg | 171 | A3256 |  |
| 1sy7 | HEM | A1883 |  |
| 1syh | CPW | A | 501 |
| 1sz7 | PLM | A | 221 |
| 1szc | CNA | A1001 |  |
| 1szg | FNS | A5570 |  |
| 1szn | MAN | A1606 |  |
| 1t02 | LVA | A | 429 |
| 1t0a | FPP | A1831 |  |
| 1t0f | MLA | A1001 |  |
| 1t0i | FMN | A | 200 |
| 1t13 | INI | A | 203 |
| 1t1s | CBQ | A2001 |  |
| 1t24 | OXQ | A | 417 |
| 1t2b | CNL | A | 500 |
| 1t3i | 2OS | A | 602 |
| 1t41 | ID5 | A | 320 |
| 1t47 | NTD | A | 551 |
| 1t48 | BB3 | A | 299 |
| 1t49 | 892 | A | 301 |
| 1t4j | FRJ | A | 299 |
| 1t4p | 2BH | A1000 |  |
| 1t4r | AHI | A1000 |  |
| 1t4t | DIR | A1000 |  |
| 1t4v | 14A | H1411 |  |
| 1t5a | FBP | A | 532 |
| 1t5b | FMN | A | 202 |
| 1t5e | 3GR | C | 361 |
| 1t5p | 1FH | A | 300 |
| 1t5s | ACP | A1001 |  |
| 1t64 | TSN | A | 386 |
| 1t7k | BH0 | B1000 |  |
| 1t7l | MRY | A1353 |  |
| 1t7r | DHT | A | 300 |
| 1t83 | NDG | A | 448 |
| 1t8s | FMP | A | 501 |
| 1t8t | CIT | B1399 |  |
| 1t8u | IDS | B | 3 |
| 1t8u | SGN | B | 2 |
| 1t8u | UAP | B | 1 |
| 1t8x | A5P | A1269 |  |
| 1t8x | PEP | A1268 |  |
| 1t93 | FLV | A | 431 |
| 1t96 | PEP | A1268 |  |
| 1t9a | 1TB | A | 695 |
| 1t9a | YF4 | B1700 |  |
| 1t9b | NSP | A | 704 |
| 1t9b | P22 | A1702 |  |
| 1t9b | P25 | B | 698 |
| 1t9c | P23 | B | 700 |
| 1t9r | CIT | A | 101 |
| 1t9s | 5GP | A | 859 |
| 1t9w | NFN | A6001 |  |
| 1ta0 | CIT | A | 274 |
| 1ta6 | 177 | A | 401 |
| 1ta8 | NMN | A | 401 |
| 1tal | TAM | A | 1 |
| 1taq | BGL | A | 833 |
| 1tb7 | B3P | A | 601 |
| 1tbb | ROL | A | 501 |
| 1tc0 | ATP | A | 301 |
| 1tc1 | FMB | A | 900 |
| 1tco | FK5 | C | 509 |
| 1tcs | NDP | A | 280 |
| 1tcv | NDS | A | 951 |
| 1td7 | NFL | A2001 |  |
| 1tdi | GSH | A | 223 |
| 1tdk | LVG | A | 491 |
| 1te2 | PGA | A | 711 |
| 1ted | MYR | A3045 |  |
| 1tet | CIT | L | 212 |
| 1tf0 | CIT | A2001 |  |
| 1tfz | 869 | A | 551 |
| 1tg2 | H2B | A | 426 |
| 1tgv | 5UD | A3001 |  |
| 1tgy | URA | A1255 |  |
| 1th6 | OIN | A | 401 |
| 1thc | FL9 | A | 130 |
| 1thg | NDG | A | 996 |
| 1thz | 326 | A4001 |  |
| 1ti1 | D12 | A | 190 |
| 1tid | ATP | A | 200 |
| 1tip | F6P | A | 401 |
| 1tiw | TFB | A2002 |  |
| 1tj5 | SUC | A | 245 |
| 1tjj | LPE | A2483 |  |
| 1tjj | PFS | A2482 |  |
| 1tjy | PAV | A | 400 |
| 1tk3 | NDG | A | 770 |
| 1tk3 | NDG | B | 779 |
| 1tka | N3T | A | 681 |
| 1tkb | N1T | A | 681 |
| 1tkc | M6T | A | 681 |
| 1tkf | DTR | A | 913 |
| 1tkg | SSA | A | 500 |
| 1tkh | DPN | A | 911 |
| 1tkj | MED | A | 913 |
| 1tkt | H12 | A | 999 |
| 1tkx | GWB | A | 999 |
| 1tky | A3S | A | 500 |
| 1tkz | H16 | A | 999 |
| 1tl1 | H18 | A | 999 |
| 1tl2 | NDG | A | 237 |
| 1tl3 | H20 | A | 999 |
| 1tlc | DGP | A | 265 |
| 1tll | FMN | A1451 |  |
| 1tlw | THM | A | 798 |
| 1tlz | URI | A | 800 |
| 1tmm | APC | B | 371 |
| 1tmm | HHR | B | 381 |
| 1tn6 | FII | B | 1 |
| 1tng | AMC | A | 900 |
| 1tnh | FBA | A | 900 |
| 1tnl | TPA | A | 900 |
| 1to2 | CIT | E | 452 |
| 1toi | HCI | A | 410 |
| 1tok | MAE | A | 410 |
| 1tom | MIN | H | 1 |
| 1tou | B1V | A | 501 |
| 1tow | CRZ | A | 501 |
| 1tpb | PGH | 1 | 250 |
| 1tpc | PGH | 1 | 250 |
| 1tph | PGH | 1 | 250 |
| 1tqh | 4PA | A | 701 |
| 1tqm | ANP | A | 283 |
| 1tqu | GHA | A1300 |  |
| 1tsi | 4PB | A | 600 |
| 1tsm | MR2 | A | 318 |
| 1tsn | C2F | A | 266 |
| 1tsn | UFP | A | 265 |
| 1tt6 | DES | A | 129 |
| 1tt8 | PHB | A | 400 |
| 1ttm | 667 | A | 264 |
| 1tu5 | NDG | A | 804 |
| 1tu5 | NDG | B | 800 |
| 1tu6 | FSP | A | 551 |
| 1tu9 | PPI | A | 302 |
| 1tuf | AZ1 | A | 502 |
| 1tug | CTP | B1156 |  |
| 1tuk | PGM | A | 103 |
| 1tuv | VK3 | A4558 |  |
| 1tv2 | BG5 | A | 459 |
| 1tv3 | BG4 | A | 459 |
| 1tv4 | BG3 | A | 511 |
| 1tv6 | CP9 | A | 561 |
| 1tve | 178 | A1360 |  |
| 1tvo | FRZ | A1001 |  |
| 1tvp | CBI | B | 699 |
| 1tw3 | ERT | A | 600 |
| 1tw5 | CBS | A | 100 |
| 1twa | ATP | A3011 |  |
| 1twf | UTP | B3571 |  |
| 1twg | CTP | B3008 |  |
| 1twr | VER | A | 300 |
| 1tx2 | 680 | A | 502 |
| 1tx8 | AM4 | A | 302 |
| 1txc | 2AN | A | 715 |
| 1txi | TX5 | A | 500 |
| 1ty8 | ADP | A | 285 |
| 1tyo | ENP | A1001 |  |
| 1typ | GSH | A | 495 |
| 1typ | SPD | A | 496 |
| 1tys | DHF | A | 566 |
| 1tys | TMP | A | 565 |
| 1tyu | MAN | A | 6 |
| 1tyu | TYV | A | 3 |
| 1tyx | ABE | A | 3 |
| 1tz2 | 1AC | A | 501 |
| 1tzc | PA5 | A | 600 |
| 1tzj | A3B | A | 501 |
| 1tzk | 2KT | A | 501 |
| 1tzm | C2N | A | 501 |
| 1tzx | CIT | A | 600 |
| 1u0f | G6P | A | 900 |
| 1u0g | E4P | A | 901 |
| 1u11 | CIT | A1001 |  |
| 1u19 | HTG | A1507 |  |
| 1u19 | PLM | A1322 |  |
| 1u1d | 181 | A3400 |  |
| 1u1f | 183 | A3300 |  |
| 1u1g | BBB | A3300 |  |
| 1u1w | 3HA | A | 700 |
| 1u1x | HHA | A | 401 |
| 1u21 | P2C | A | 211 |
| 1u22 | THG | A | 773 |
| 1u25 | IHS | A2165 |  |
| 1u29 | I3P | A | 101 |
| 1u2g | APR | A | 500 |
| 1u2g | APR | B | 600 |
| 1u2g | NDP | C | 400 |
| 1u2r | APR | A1699 |  |
| 1u2z | SAH | A | 801 |
| 1u30 | GOX | A | 500 |
| 1u3d | ANP | A | 511 |
| 1u3d | NDS | A | 512 |
| 1u3g | THF | A | 501 |
| 1u3q | 272 | A | 501 |
| 1u3r | 338 | A | 201 |
| 1u3s | 797 | A | 201 |
| 1u3t | CCB | A1378 |  |
| 1u3u | BNF | A1378 |  |
| 1u3v | HPL | A1378 |  |
| 1u3w | FXY | A1378 |  |
| 1u43 | SUD | A | 669 |
| 1u4g | HPI | A | 800 |
| 1u4j | MAN | B1008 |  |
| 1u4l | H1S | A | 301 |
| 1u4m | H3S | A | 301 |
| 1u4o | NDD | A | 401 |
| 1u4s | BIH | A | 336 |
| 1u5a | BIK | A1001 |  |
| 1u5u | HEM | A | 999 |
| 1u6q | 745 | A | 245 |
| 1u6s | DCC | A | 960 |
| 1u71 | MXA | A | 187 |
| 1u7t | TDT | A | 501 |
| 1u7w | CTP | A | 500 |
| 1u88 | GTY | A | 220 |
| 1u8c | NDG | A2585 |  |
| 1u8c | NDG | B3320 |  |
| 1u8c | NDG | B3655 |  |
| 1u8v | FAD | A | 492 |
| 1u8x | G6P | X | 902 |
| 1u9e | 397 | A | 201 |
| 1u9n | CNS | A1001 |  |
| 1u9o | CNS | B | 217 |
| 1u9q | 186 | X | 300 |
| 1u9z | R5P | A5301 |  |
| 1ua3 | MLR | A1990 |  |
| 1ua4 | BGC | A2457 |  |
| 1uak | SAM | A | 301 |
| 1ual | SAH | A | 301 |
| 1uaq | DUC | A | 300 |
| 1ub5 | SPB | B | 701 |
| 1ubx | FPP | A | 401 |
| 1uc2 | SUC | A1001 |  |
| 1uc4 | CNC | B2601 |  |
| 1uc9 | ADP | A | 300 |
| 1ucc | U3P | A | 300 |
| 1ucd | URA | A | 501 |
| 1udb | UFG | A | 341 |
| 1udc | UFM | A | 341 |
| 1udw | CTP | A | 301 |
| 1uei | UTP | A | 301 |
| 1uej | CIT | A | 302 |
| 1uej | CTN | A | 301 |
| 1uf8 | ING | A | 999 |
| 1uh3 | ACI | A | 901 |
| 1uh3 | GLD | A | 902 |
| 1uhh | CZP | A1001 |  |
| 1uhj | CZB | A1001 |  |
| 1uhk | CZN | A1001 |  |
| 1uhl | MEI | A1001 |  |
| 1ui0 | URA | A | 211 |
| 1uio | HPR | A | 353 |
| 1uj2 | ADP | A2302 |  |
| 1uj5 | 5RP | A | 302 |
| 1uj6 | A5P | A | 228 |
| 1ujp | CIT | A1001 |  |
| 1uk0 | FRM | A | 501 |
| 1uk1 | FRQ | A | 501 |
| 1uk6 | PPI | A1300 |  |
| 1uk7 | BUA | A1300 |  |
| 1uk9 | IVA | A1300 |  |
| 1uke | UP5 | A | 195 |
| 1ult | CIT | A1001 |  |
| 1um4 | SH4 | H1001 |  |
| 1umc | 4MV | D1403 |  |
| 1umd | TDP | A1402 |  |
| 1umg | 2FP | A | 400 |
| 1umi | NDG | A | 298 |
| 1uml | FR4 | A1001 |  |
| 1ump | SQA | A1630 |  |
| 1un9 | ANP | A1551 |  |
| 1uo9 | SIN | A | 313 |
| 1uog | P1C | A1311 |  |
| 1uou | CMU | A1481 |  |
| 1uoz | SSG | A | 502 |
| 1upf | URF | A | 999 |
| 1upj | U01 | A | 201 |
| 1upv | 444 | A | 462 |
| 1uqt | U2F | A | 900 |
| 1uqu | UPG | A1457 |  |
| 1uqz | GCV | A1377 |  |
| 1ur1 | XYS | A1376 |  |
| 1ur4 | B2G | A1399 |  |
| 1ur9 | PHJ | A2503 |  |
| 1urs | MLR | A1396 |  |
| 1us0 | CIT | A | 400 |
| 1us0 | LDT | A | 320 |
| 1us0 | NDP | A | 318 |
| 1usn | IN9 | A | 300 |
| 1usy | HIS | D1276 |  |
| 1ut6 | A8N | A1538 |  |
| 1utn | ABN | A | 246 |
| 1uto | PEA | A1246 |  |
| 1utt | CP8 | A1266 |  |
| 1utz | PF3 | A1265 |  |
| 1uu7 | BI2 | A1364 |  |
| 1uu9 | BI3 | A1370 |  |
| 1uum | AFI | A | 400 |
| 1uuy | PPI | A1166 |  |
| 1uv6 | CCE | D1206 |  |
| 1uvr | BI8 | A1372 |  |
| 1uvs | I11 | H | 11 |
| 1uvt | I48 | H | 1 |
| 1uw1 | ADP | A1074 |  |
| 1uw6 | NCT | A1208 |  |
| 1uwk | URO | A1559 |  |
| 1uwz | THU | A | 138 |
| 1uxa | SIA | A1366 |  |
| 1uxg | FMR | A1311 |  |
| 1uxr | F6P | A1503 |  |
| 1uxt | G1P | A1502 |  |
| 1uxy | FAD | A | 401 |
| 1uy6 | PU3 | A1224 |  |
| 1uy7 | PU4 | A1224 |  |
| 1uy8 | PU5 | A1224 |  |
| 1uy9 | PU6 | A1224 |  |
| 1uyc | PU7 | A1224 |  |
| 1uyd | PU8 | A1224 |  |
| 1uye | PU9 | A1224 |  |
| 1uyf | PU1 | A1224 |  |
| 1uyg | PU2 | A1224 |  |
| 1uyh | PU0 | A1224 |  |
| 1uyi | PUZ | A1224 |  |
| 1uyk | PUX | A1224 |  |
| 1uyr | D1L | A3219 |  |
| 1uys | H1L | A3000 |  |
| 1uz8 | MAG | A1213 |  |
| 1uz9 | CRS | A1022 |  |
| 1uze | EAL | A3002 |  |
| 1uzf | MCO | A | 702 |
| 1uzi | V4O | A1001 |  |
| 1uzr | CIT | A1294 |  |
| 1uzu | INR | A1000 |  |
| 1v0f | SIA | C1686 |  |
| 1v0h | SHA | X | 253 |
| 1v0j | FAD | A1393 |  |
| 1v0o | INR | A2638 |  |
| 1v0p | PVB | A1287 |  |
| 1v0y | HI5 | A1515 |  |
| 1v0z | BMA | A1486 |  |
| 1v0z | MAN | A1483 |  |
| 1v1a | KDG | A1302 |  |
| 1v1j | FA3 | A | 201 |
| 1v1k | 3FP | A | 299 |
| 1v25 | ANP | A | 666 |
| 1v26 | MYR | A1001 |  |
| 1v2a | GTS | B2001 |  |
| 1v2e | KMT | A | 520 |
| 1v2g | OCA | A | 201 |
| 1v2n | BBA | T | 1 |
| 1v2o | ANH | T | 800 |
| 1v2x | SAM | A | 400 |
| 1v34 | UTP | A3000 |  |
| 1v39 | M7G | A | 401 |
| 1v3q | 2DI | E | 290 |
| 1v3v | 5OP | A1360 |  |
| 1v40 | O16 | B3401 |  |
| 1v41 | AZG | E | 290 |
| 1v45 | 3DG | E | 290 |
| 1v48 | HA1 | A | 290 |
| 1v54 | CHD | B4085 |  |
| 1v54 | CHD | C3271 |  |
| 1v54 | CHD | C3525 |  |
| 1v54 | CHD | J3060 |  |
| 1v5e | FAD | A1601 |  |
| 1v5g | HTL | A1602 |  |
| 1v5y | 4HC | A1455 |  |
| 1v5z | 2HC | A | 815 |
| 1v6u | XYS | A1461 |  |
| 1v79 | FR7 | A1001 |  |
| 1v7a | FRC | A1001 |  |
| 1v7r | CIT | A1200 |  |
| 1v7w | NDG | A1004 |  |
| 1v7z | CRN | A | 401 |
| 1v84 | NDG | A | 506 |
| 1v84 | UDP | A | 503 |
| 1v8t | R5P | A | 601 |
| 1v97 | FYX | A3006 |  |
| 1v9y | HEM | A1140 |  |
| 1va5 | SOG | A1001 |  |
| 1va6 | ADP | A | 521 |
| 1vah | NPO | A | 701 |
| 1vba | J78 | 1 | 500 |
| 1vba | MYR | 4 | 1 |
| 1vbb | J80 | 1 | 500 |
| 1vbb | MYR | 4 | 1 |
| 1vbc | J77 | 1 | 500 |
| 1vbc | MYR | 4 | 1 |
| 1vbd | J78 | 1 | 500 |
| 1vbd | MYR | 4 | 1 |
| 1vbe | J78 | 1 | 500 |
| 1vbe | MYR | 4 | 1 |
| 1vbj | CIT | A4001 |  |
| 1vc8 | 5FA | A | 501 |
| 1vcr | CHL | A | 261 |
| 1vdv | YSH | A5101 |  |
| 1ve7 | 4NP | A | 701 |
| 1vea | HBN | B1001 |  |
| 1vf1 | GSH | A2457 |  |
| 1vf2 | GTX | A2457 |  |
| 1vfs | DCS | A | 401 |
| 1vgn | OPS | A1161 |  |
| 1vi0 | DCC | A | 205 |
| 1vif | FOL | A | 1 |
| 1vj5 | CIU | A | 781 |
| 1vj7 | GPX | B1999 |  |
| 1vjd | ATP | A | 417 |
| 1vjy | 460 | A | 999 |
| 1vkf | CIT | A | 501 |
| 1vkg | CRI | A | 402 |
| 1vkp | MPO | A1000 |  |
| 1vl1 | CIT | A | 300 |
| 1vlb | PCD | A | 921 |
| 1vma | CIT | B | 295 |
| 1vmk | GUN | A | 300 |
| 1vp8 | FMN | A | 500 |
| 1vpe | ANP | A | 400 |
| 1vps | SIA | A | 1 |
| 1vr0 | 3SL | A | 300 |
| 1vrh | SD8 | 1 | 290 |
| 1vrq | FON | A1003 |  |
| 1vrt | NVP | A | 999 |
| 1vru | AAP | A | 999 |
| 1vso | AT1 | A | 258 |
| 1vyp | TNF | X | 500 |
| 1vyq | DUX | A1160 |  |
| 1vyr | FMN | A1401 |  |
| 1vyw | 292 | A2300 |  |
| 1vyz | N5B | A | 300 |
| 1vz5 | SIN | A1301 |  |
| 1vzq | SHY | H1256 |  |
| 1w07 | FAD | A1660 |  |
| 1w0c | TAQ | A | 301 |
| 1w0p | SIA | A1784 |  |
| 1w19 | D1D | E1166 |  |
| 1w19 | DTV | C1161 |  |
| 1w19 | T1P | A1163 |  |
| 1w19 | T2P | B1163 |  |
| 1w19 | T4P | D1163 |  |
| 1w19 | T5P | C1162 |  |
| 1w1a | NDG | 11261 |  |
| 1w1i | MAN | A | 833 |
| 1w1p | GIO | A1518 |  |
| 1w1q | ZIP | A1536 |  |
| 1w1t | CHQ | A1513 |  |
| 1w1x | BMA | A1480 |  |
| 1w1x | MAN | B2481 |  |
| 1w1x | SIA | B2477 |  |
| 1w1y | TYP | A1507 |  |
| 1w1y | TYP | A1509 |  |
| 1w20 | BMA | A1484 |  |
| 1w20 | MAN | B2484 |  |
| 1w20 | SIA | B2477 |  |
| 1w21 | BMA | A1484 |  |
| 1w21 | MAN | B2484 |  |
| 1w21 | SIA | B2477 |  |
| 1w22 | NHB | A1378 |  |
| 1w29 | D1D | E1165 |  |
| 1w29 | TS0 | D1162 |  |
| 1w29 | TS1 | A1162 |  |
| 1w29 | TS1 | B1162 |  |
| 1w2c | ANP | A1462 |  |
| 1w2c | I3P | A1463 |  |
| 1w2d | ADP | A1462 |  |
| 1w2t | CIT | A1433 |  |
| 1w2t | SUC | A1434 |  |
| 1w2y | DUN | A1230 |  |
| 1w3f | NLC | A1320 |  |
| 1w3g | NLC | A1319 |  |
| 1w3l | BGC | A1307 |  |
| 1w3t | 3GR | A1297 |  |
| 1w3y | PVC | A1891 |  |
| 1w48 | APC | A | 700 |
| 1w4l | GL8 | A1540 |  |
| 1w4o | UA3 | A1125 |  |
| 1w4p | UM3 | A1125 |  |
| 1w4q | UMF | A1125 |  |
| 1w4r | TTP | A | 300 |
| 1w52 | DDQ | X | 501 |
| 1w5b | GTP | A | 500 |
| 1w5c | HEM | Z5051 |  |
| 1w5f | G2P | A | 500 |
| 1w61 | PYC | A | 700 |
| 1w6f | ISZ | A1276 |  |
| 1w6j | C14 | A1735 |  |
| 1w6j | R71 | A1733 |  |
| 1w6p | NDG | A4000 |  |
| 1w6r | GNT | A1536 |  |
| 1w6s | PQQ | A1596 |  |
| 1w6u | HXC | A1330 |  |
| 1w78 | ADP | A1425 |  |
| 1w7h | 3IP | A1355 |  |
| 1w7m | DPN | A | 914 |
| 1w7q | D3P | A | 10 |
| 1w7r | D3P | A | 10 |
| 1w82 | L10 | A1356 |  |
| 1w83 | L11 | A1355 |  |
| 1w84 | L12 | A1356 |  |
| 1w8c | N69 | A1301 |  |
| 1w8h | NDG | A | 335 |
| 1w8l | 1P3 | A1166 |  |
| 1w8m | E1P | A1166 |  |
| 1w8o | CIT | A1649 |  |
| 1w8s | FBP | A | 270 |
| 1w8u | BMA | A1143 |  |
| 1w96 | S1A | A1567 |  |
| 1w9b | CGT | M | 999 |
| 1w9t | XYS | A1146 |  |
| 1wa5 | GTP | A1177 |  |
| 1waj | 5GP | A | 904 |
| 1wax | LO1 | A1300 |  |
| 1way | L02 | B1248 |  |
| 1wb1 | DXC | B1393 |  |
| 1wb1 | DXC | C1477 |  |
| 1wb1 | DXC | C1478 |  |
| 1wb1 | DXC | C1480 |  |
| 1wb5 | SYR | A2086 |  |
| 1wb6 | VXX | A2086 |  |
| 1wbe | DKA | A1001 |  |
| 1wbg | L03 | B1248 |  |
| 1wbj | G3P | A1268 |  |
| 1wbk | 568 | B1100 |  |
| 1wbl | AMG | A | 400 |
| 1wbm | BLL | B1100 |  |
| 1wbn | L09 | A1355 |  |
| 1wbo | 2CH | A1355 |  |
| 1wbt | WBT | A1355 |  |
| 1wbu | WBU | A1125 |  |
| 1wc1 | TAT | A1500 |  |
| 1wc3 | APC | A1500 |  |
| 1wc7 | PP3 | A1214 |  |
| 1wc9 | MYR | A1172 |  |
| 1wcc | CIG | A1299 |  |
| 1wdi | CIT | A1322 |  |
| 1we2 | DHK | A | 181 |
| 1we3 | ADP | A1551 |  |
| 1wei | ADE | A1428 |  |
| 1wg8 | SAM | A3142 |  |
| 1wht | BZS | A | 430 |
| 1wk9 | TSB | A | 999 |
| 1wkh | PPE | A | 513 |
| 1wkl | ATP | B | 856 |
| 1wma | AB3 | A | 307 |
| 1wma | NDP | A | 309 |
| 1wmr | NDG | B1000 |  |
| 1wmz | A2G | B | 502 |
| 1wn3 | HXC | C2003 |  |
| 1wn6 | BST | A1003 |  |
| 1wnb | BTL | B5001 |  |
| 1wnl | ADP | A1301 |  |
| 1wnl | ADP | B1302 |  |
| 1woa | G2H | A | 300 |
| 1wog | 16D | A1401 |  |
| 1wok | CNQ | A | 1 |
| 1wom | PGO | A | 601 |
| 1woo | THG | A2887 |  |
| 1wop | FFO | A2887 |  |
| 1wop | FFO | A2888 |  |
| 1woq | BGC | A | 290 |
| 1wor | RED | A | 900 |
| 1wpg | ADP | A1002 |  |
| 1wpl | HBI | A1003 |  |
| 1wpq | 13P | A2002 |  |
| 1wpv | HIS | A1001 |  |
| 1wqv | PSM | H1001 |  |
| 1wrr | UNC | A | 999 |
| 1ws1 | BB2 | A | 400 |
| 1ws2 | URN | A | 900 |
| 1ws3 | URA | A | 900 |
| 1ws4 | AMG | E | 503 |
| 1ws5 | MMA | A | 501 |
| 1wss | 3CB | H2001 |  |
| 1wsv | THH | A3001 |  |
| 1wtc | ACP | A | 370 |
| 1wu1 | D91 | A | 700 |
| 1wua | AP8 | A1400 |  |
| 1wun | P5B | H2001 |  |
| 1wut | BN2 | A | 930 |
| 1wuu | ANP | A | 395 |
| 1wuw | TSU | A | 101 |
| 1wuy | BN3 | A | 930 |
| 1wv0 | BN4 | A | 930 |
| 1wv1 | BN5 | A | 930 |
| 1wv7 | 5PI | H2001 |  |
| 1wva | S2C | A1317 |  |
| 1wvg | APR | A | 400 |
| 1wvg | CXY | A | 401 |
| 1wvj | IBC | A1001 |  |
| 1wvx | BD4 | A1001 |  |
| 1ww4 | SIA | A | 202 |
| 1ww5 | SGA | A | 200 |
| 1wwj | MLT | A6001 |  |
| 1wxz | FRL | A | 401 |
| 1wy7 | SAH | A1001 |  |
| 1wyg | SAL | A4005 |  |
| 1wyv | AOA | B1476 |  |
| 1wz1 | DNS | H | 200 |
| 1wzd | YOK | A | 901 |
| 1wzf | YOL | A | 901 |
| 1wzg | YOM | A | 901 |
| 1wzu | MLT | A | 301 |
| 1x06 | FPS | A | 901 |
| 1x09 | IPE | A | 901 |
| 1x0p | FAD | A9150 |  |
| 1x1i | 46M | A | 780 |
| 1x1j | 46D | A2001 |  |
| 1x1v | MMA | A | 801 |
| 1x1z | BMP | A | 301 |
| 1x29 | PMG | A | 413 |
| 1x2b | STX | A | 401 |
| 1x2e | ATX | A | 401 |
| 1x2h | LPA | A | 338 |
| 1x31 | DMG | B1005 |  |
| 1x38 | IDD | A1001 |  |
| 1x39 | IDE | A1001 |  |
| 1x3k | HEM | A1001 |  |
| 1x3m | ADP | A | 403 |
| 1x3n | ANP | A | 404 |
| 1x3z | SUC | A1001 |  |
| 1x6n | AO3 | A | 564 |
| 1x70 | 715 | A | 801 |
| 1x71 | DB1 | A | 201 |
| 1x76 | 697 | A | 201 |
| 1x78 | 244 | A | 201 |
| 1x7b | 041 | A | 201 |
| 1x7e | 244 | A | 201 |
| 1x7r | GEN | A | 201 |
| 1x82 | PA5 | A | 301 |
| 1x89 | CM1 | A | 200 |
| 1x8b | 824 | A | 901 |
| 1x8d | RNS | A1105 |  |
| 1x8j | AE2 | A | 400 |
| 1x8k | ANR | A1102 |  |
| 1x8l | OXR | A1102 |  |
| 1x8p | HEM | A | 185 |
| 1x8u | CM2 | A | 200 |
| 1x8v | ESL | A | 471 |
| 1x9h | F6R | A | 701 |
| 1x9i | G6Q | A | 600 |
| 1x9j | CIT | D | 381 |
| 1x9p | C15 | A | 998 |
| 1x9q | FLU | A1341 |  |
| 1x9t | C15 | A1014 |  |
| 1xa5 | KAR | A | 153 |
| 1xan | HXP | A1024 |  |
| 1xap | TTB | A | 1 |
| 1xbb | STI | A | 1 |
| 1xbo | IX1 | A | 322 |
| 1xbt | TTP | A1195 |  |
| 1xbu | IOY | A | 913 |
| 1xby | 5RP | A | 501 |
| 1xc7 | GL6 | A | 998 |
| 1xcj | NMG | A | 237 |
| 1xco | UVW | A | 401 |
| 1xcw | 3SA | A | 501 |
| 1xcx | IAB | A | 501 |
| 1xd1 | 6SA | A | 501 |
| 1xda | MYR | B | 39 |
| 1xdd | AAY | A | 401 |
| 1xdn | ATP | A | 501 |
| 1xdp | ATP | A | 701 |
| 1xds | DRA | A5600 |  |
| 1xdu | SFG | A5635 |  |
| 1xe7 | GUN | A | 210 |
| 1xe8 | ADE | A | 500 |
| 1xe8 | CIT | A1102 |  |
| 1xef | ATP | A | 800 |
| 1xet | MCA | D4001 |  |
| 1xey | GUA | A | 506 |
| 1xf0 | ASD | A | 600 |
| 1xf1 | CIT | A1101 |  |
| 1xfv | 3AT | A | 903 |
| 1xfw | CMP | A | 901 |
| 1xg3 | SIN | A2106 |  |
| 1xgi | NST | B | 416 |
| 1xgj | HTC | A | 777 |
| 1xh0 | AAO | A | 500 |
| 1xhl | TNE | A1279 |  |
| 1xi2 | CB1 | A | 501 |
| 1xih | SOR | A | 389 |
| 1xii | XUL | A | 389 |
| 1xio | PEE | A | 302 |
| 1xje | TTP | A1005 |  |
| 1xjf | DTP | A1002 |  |
| 1xjg | DTP | A1004 |  |
| 1xjg | UDP | A1001 |  |
| 1xji | C14 | A | 403 |
| 1xji | D10 | A | 405 |
| 1xji | D12 | A | 401 |
| 1xjj | DGT | A1001 |  |
| 1xjj | DGT | A1003 |  |
| 1xjk | ADP | A1001 |  |
| 1xjm | TTP | A1001 |  |
| 1xjn | DTP | A1005 |  |
| 1xjn | DTP | C1007 |  |
| 1xjq | ADP | A | 900 |
| 1xjt | CIT | A | 192 |
| 1xk9 | P34 | A1001 |  |
| 1xkk | FMM | A | 91 |
| 1xkx | IMK | A | 910 |
| 1xl0 | OX2 | A | 998 |
| 1xl1 | TH1 | A | 998 |
| 1xl6 | SPM | B3001 |  |
| 1xl8 | OCB | B | 613 |
| 1xlf | GCO | A | 400 |
| 1xlr | VNL | A | 181 |
| 1xlt | SUC | A | 600 |
| 1xlt | SUC | D | 600 |
| 1xlz | FIL | A1003 |  |
| 1xm6 | 5RM | A1003 |  |
| 1xmm | G7M | B1502 |  |
| 1xmm | M7G | A | 401 |
| 1xmn | IDS | B | 3 |
| 1xng | ATP | A | 303 |
| 1xng | DND | A | 301 |
| 1xnj | ADX | A2900 |  |
| 1xnn | HYQ | A | 101 |
| 1xnx | ATE | A | 500 |
| 1xny | 191 | A5601 |  |
| 1xny | BTN | A9600 |  |
| 1xnz | FCD | A | 268 |
| 1xo2 | FSE | B | 309 |
| 1xog | ABW | A1000 |  |
| 1xoi | 288 | A | 862 |
| 1xoi | NBG | B1861 |  |
| 1xoi | PLP | B1860 |  |
| 1xom | CIO | A | 603 |
| 1xor | ZAR | A | 701 |
| 1xow | R18 | A1001 |  |
| 1xoz | CIA | A | 501 |
| 1xp8 | SAP | A | 400 |
| 1xpm | HMG | A1401 |  |
| 1xpq | FAD | A | 803 |
| 1xpq | SPM | A | 924 |
| 1xpy | NLQ | C3376 |  |
| 1xpz | 4TZ | A | 270 |
| 1xq0 | 4TR | A | 270 |
| 1xqc | AEJ | A1001 |  |
| 1xqd | DND | A | 502 |
| 1xql | PMH | A | 503 |
| 1xql | PMP | A | 501 |
| 1xqp | 8HG | A | 257 |
| 1xqx | PCS | A | 300 |
| 1xr2 | C2F | A1200 |  |
| 1xr3 | ISZ | A | 263 |
| 1xs1 | DUT | A1194 |  |
| 1xs7 | MMI | D | 386 |
| 1xt3 | CIT | A | 101 |
| 1xt3 | IDS | A | 61 |
| 1xt4 | GUN | A | 901 |
| 1xtp | SAI | A | 401 |
| 1xtu | CTP | A1260 |  |
| 1xuz | MMN | A3001 |  |
| 1xuz | PEP | A2001 |  |
| 1xv5 | UDP | A1402 |  |
| 1xv9 | CI2 | D1001 |  |
| 1xva | SAM | A | 293 |
| 1xvb | BHL | A1200 |  |
| 1xve | 3BB | A1172 |  |
| 1xvv | CCQ | A | 501 |
| 1xw4 | ADP | X | 998 |
| 1xws | BI1 | A1001 |  |
| 1xx6 | ADP | A | 301 |
| 1xxr | MAN | B | 725 |
| 1xxs | STE | A | 201 |
| 1xyc | 3MF | A | 950 |
| 1xym | GLO | A | 950 |
| 1xz1 | HLT | A2001 |  |
| 1xz3 | ICF | A | 201 |
| 1xz8 | 3GP | A | 300 |
| 1xzb | MAC | A | 801 |
| 1xzc | PMB | A | 801 |
| 1xzq | FON | A | 451 |
| 1xzq | FON | B | 118 |
| 1y0b | G4P | A | 888 |
| 1y0g | 8PP | A | 320 |
| 1y0p | FAD | A1805 |  |
| 1y0p | HEM | A | 801 |
| 1y0p | MEZ | A1806 |  |
| 1y1a | GSH | B | 601 |
| 1y1d | FHI | A2000 |  |
| 1y1m | AC5 | A1001 |  |
| 1y1p | NMN | A1001 |  |
| 1y1r | ANU | B | 707 |
| 1y1s | URA | A8001 |  |
| 1y20 | 1AC | A1001 |  |
| 1y2b | DEE | A | 502 |
| 1y2c | 3DE | A1003 |  |
| 1y2d | 4DE | A | 601 |
| 1y2e | 5DE | A1003 |  |
| 1y2f | WAI | A | 300 |
| 1y2h | 6DE | A | 101 |
| 1y2k | 7DE | A | 602 |
| 1y38 | G3P | A | 201 |
| 1y4l | SVR | B | 301 |
| 1y52 | BTN | Y | 802 |
| 1y55 | BTN | Y | 802 |
| 1y56 | ATP | A | 801 |
| 1y56 | CXS | A | 803 |
| 1y57 | MPZ | A | 600 |
| 1y5r | C0R | A | 11 |
| 1y5v | NE8 | A | 500 |
| 1y5w | NEZ | A | 500 |
| 1y5x | E89 | A | 500 |
| 1y60 | H4M | B | 997 |
| 1y6a | AAZ | A | 201 |
| 1y6b | AAX | A | 201 |
| 1y6q | TDI | A | 233 |
| 1y7i | SAL | A | 501 |
| 1y7p | RIP | A4182 |  |
| 1y7t | NDP | A1334 |  |
| 1y7v | INS | A | 507 |
| 1y80 | B1M | A | 301 |
| 1y8e | SVR | A | 501 |
| 1y8j | STS | A | 900 |
| 1y8o | RED | B | 373 |
| 1y8p | ATP | A | 504 |
| 1y8r | ATP | B | 802 |
| 1y8y | CT7 | A | 401 |
| 1y91 | CT9 | A | 401 |
| 1y93 | HAE | A | 301 |
| 1y9d | FAD | A2603 |  |
| 1y9q | MED | A | 201 |
| 1y9r | 1CA | A2001 |  |
| 1y9t | HHG | A | 150 |
| 1ya8 | MVB | A2011 |  |
| 1yae | DOQ | F1603 |  |
| 1yas | HIS | A | 300 |
| 1yb1 | AE2 | A | 602 |
| 1yba | AKG | A | 413 |
| 1ybg | TAV | A | 550 |
| 1ybu | APC | A | 601 |
| 1ybv | BEA | A | 285 |
| 1yc1 | 4BC | A | 401 |
| 1yc4 | 43P | A | 301 |
| 1yc5 | NCA | A2001 |  |
| 1yci | NDF | A | 400 |
| 1ydr | IQP | E | 351 |
| 1yds | IQS | E | 351 |
| 1ydt | IQB | E | 351 |
| 1yec | PNB | H | 551 |
| 1yef | PNC | L | 551 |
| 1yei | PGG | L | 551 |
| 1yej | PNF | L | 551 |
| 1yek | NPO | H | 551 |
| 1yfk | CIT | A | 500 |
| 1yfw | 4AA | A | 401 |
| 1yfy | 3HA | A | 401 |
| 1ygj | RMC | A1001 |  |
| 1ygy | TAR | A | 600 |
| 1ygy | TAR | B1600 |  |
| 1yh8 | PAM | A | 801 |
| 1yhc | PAM | A | 802 |
| 1yhj | R6C | A | 313 |
| 1yhl | DMA | A1301 |  |
| 1yi1 | 1TB | A | 695 |
| 1yi3 | LY2 | A | 306 |
| 1yia | HRP | B1154 |  |
| 1yik | MM1 | A | 500 |
| 1yil | MM2 | A | 500 |
| 1yim | CM4 | A | 600 |
| 1ykd | CMP | A | 501 |
| 1yki | CIT | C4220 |  |
| 1yki | NFZ | A1219 |  |
| 1ykr | 628 | A | 299 |
| 1yky | 1BO | X | 208 |
| 1ylj | SUC | A1050 |  |
| 1ymf | ADP | A | 998 |
| 1ymt | DR9 | A | 500 |
| 1ynd | SFA | A | 401 |
| 1ynh | SUO | A1001 |  |
| 1yni | SUG | A1001 |  |
| 1ynk | SC5 | H | 501 |
| 1ynl | NES | L | 501 |
| 1ynp | SUC | A | 298 |
| 1ynq | NDP | B | 301 |
| 1ynq | SUC | A | 298 |
| 1yns | HPO | A1261 |  |
| 1yoe | RIB | A2001 |  |
| 1yom | P01 | A | 1 |
| 1yon | APX | A | 501 |
| 1yoo | IVA | A | 414 |
| 1you | PFD | A | 998 |
| 1yow | P0E | A | 201 |
| 1yp0 | PEF | A | 201 |
| 1yp2 | PMB | A3226 |  |
| 1yp3 | ATP | A | 757 |
| 1yp4 | ADP | A | 557 |
| 1yp4 | ADQ | B | 506 |
| 1ypk | CCR | H5555 |  |
| 1ypl | RA8 | H5555 |  |
| 1ypm | RA4 | H5555 |  |
| 1yq3 | PEE | D | 104 |
| 1yq4 | PEE | C | 145 |
| 1yqc | GLV | A1001 |  |
| 1yqj | 6NP | A | 362 |
| 1yqt | ADP | A | 593 |
| 1yqu | GUN | A | 400 |
| 1yqy | 915 | A | 782 |
| 1yqz | FAD | A | 439 |
| 1yrs | L47 | A | 604 |
| 1yrx | D9G | A | 302 |
| 1ys4 | MLA | A | 903 |
| 1ys4 | MLA | A | 904 |
| 1yt9 | OIS | A1000 |  |
| 1yte | PCP | A | 401 |
| 1ytm | ATP | A | 541 |
| 1ytm | ATP | B1541 |  |
| 1ytm | OXD | A | 543 |
| 1ytm | OXD | B1543 |  |
| 1ytz | DR6 | C | 205 |
| 1ytz | DR6 | I | 183 |
| 1ytz | DR6 | T | 103 |
| 1yuc | EPH | A1001 |  |
| 1yuk | NDG | A | 402 |
| 1yuk | NDG | B | 463 |
| 1yum | CIT | A | 301 |
| 1yum | NCN | A | 302 |
| 1yv3 | ADP | A1000 |  |
| 1yv3 | BIT | A | 800 |
| 1yve | HIO | I | 603 |
| 1yve | NDP | I | 600 |
| 1yvf | PH7 | A | 800 |
| 1yvj | DTV | A2962 |  |
| 1yvm | TMG | A | 501 |
| 1yvx | IPC | A | 580 |
| 1yvz | JPC | A | 578 |
| 1yw2 | PGJ | A2001 |  |
| 1yw7 | A41 | A | 482 |
| 1yw8 | A75 | A | 104 |
| 1yw9 | A84 | A | 482 |
| 1yxi | F6P | A | 339 |
| 1yxm | ADE | B4002 |  |
| 1yy4 | 4NA | A | 201 |
| 1yy5 | FAD | A | 803 |
| 1yy7 | CIT | B | 501 |
| 1yye | 196 | A | 531 |
| 1yyr | SAZ | A | 709 |
| 1yyu | RAZ | A | 710 |
| 1yyz | F6P | A | 339 |
| 1yz0 | F6P | A1843 |  |
| 1yz3 | SKA | A3001 |  |
| 1yzx | GSF | A | 301 |
| 1z03 | OCH | A4600 |  |
| 1z0j | GTP | A | 500 |
| 1z0n | BCD | A1200 |  |
| 1z0s | ATP | A | 737 |
| 1z11 | 8MO | A | 501 |
| 1z1e | CIT | A | 401 |
| 1z1f | CIT | A | 401 |
| 1z1h | HBB | A | 201 |
| 1z1r | HBH | A | 201 |
| 1z2l | 1AL | B | 516 |
| 1z2n | ADP | X | 871 |
| 1z2o | ADP | X | 871 |
| 1z2o | I4P | X2699 |  |
| 1z2p | ACP | X1476 |  |
| 1z2p | I3S | X | 500 |
| 1z34 | 2FD | A | 300 |
| 1z35 | 2FA | A | 300 |
| 1z36 | FMC | A | 300 |
| 1z39 | 2ND | A | 300 |
| 1z3c | SA8 | A | 299 |
| 1z3n | 3NA | A | 320 |
| 1z3t | CBI | A | 500 |
| 1z3w | IDC | A | 600 |
| 1z45 | GUD | A | 704 |
| 1z4l | TMP | A1001 |  |
| 1z4o | GL1 | A1220 |  |
| 1z4p | DGP | X | 1 |
| 1z4q | D4M | A1489 |  |
| 1z53 | PP9 | A | 296 |
| 1z57 | DBQ | A | 1 |
| 1z5b | ADP | A1001 |  |
| 1z5u | CMP | A | 502 |
| 1z5v | GSP | A2466 |  |
| 1z62 | IAA | A | 990 |
| 1z68 | NDG | A2271 |  |
| 1z6d | IMP | A | 919 |
| 1z6l | DRE | A | 804 |
| 1z6l | FAD | A | 803 |
| 1z6p | 194 | A | 843 |
| 1z6q | 195 | A | 843 |
| 1z6t | ADP | A | 900 |
| 1z6t | ADP | B | 901 |
| 1z70 | CXS | X3004 |  |
| 1z71 | L17 | A1001 |  |
| 1z7e | ATP | A1001 |  |
| 1z7e | UGA | A1101 |  |
| 1z7s | MYR | 4 | 1 |
| 1z7x | CIT | X | 900 |
| 1z82 | G3H | A | 600 |
| 1z82 | G3P | B | 600 |
| 1z82 | NDP | A | 500 |
| 1z83 | AP5 | A | 631 |
| 1z8a | 62P | A | 600 |
| 1z8d | ADE | A | 902 |
| 1z8n | 1IQ | A | 694 |
| 1z8n | 1IQ | A | 695 |
| 1z8o | DEB | A | 420 |
| 1z8q | DEB | A | 420 |
| 1z8q | HEM | A | 410 |
| 1z95 | 198 | A | 501 |
| 1z9g | RRT | E1006 |  |
| 1z9j | BCL | A | 850 |
| 1z9j | BCL | B | 852 |
| 1z9n | HEM | A1001 |  |
| 1z9t | CIT | A | 244 |
| 1z9y | FUN | A | 500 |
| 1za2 | CTP | B | 155 |
| 1zaf | 789 | A | 201 |
| 1zaj | M2P | A3001 |  |
| 1zao | ATP | A | 286 |
| 1zar | ADP | A | 401 |
| 1zb6 | DIN | A | 401 |
| 1zb6 | GST | A | 502 |
| 1zc2 | CIT | B | 502 |
| 1zcw | GPP | A | 401 |
| 1zd2 | NC3 | P | 700 |
| 1zd3 | NC4 | A | 790 |
| 1zd4 | NC6 | A | 700 |
| 1zd5 | NC7 | A | 700 |
| 1zdp | TIO | E1006 |  |
| 1zds | ACM | A2500 |  |
| 1zdt | PEF | A1001 |  |
| 1zdw | FLV | A | 401 |
| 1zea | CIT | L1001 |  |
| 1zea | DGN | A | 5 |
| 1zed | PNP | A | 901 |
| 1zeh | CRS | A | 22 |
| 1zeh | CRS | D | 33 |
| 1zfk | NR2 | A1400 |  |
| 1zfq | ZEC | A | 300 |
| 1zg8 | L98 | A | 401 |
| 1zgb | A1E | A1001 |  |
| 1zgc | A2E | A1002 |  |
| 1zgf | TRU | A | 300 |
| 1zgi | 382 | A1001 |  |
| 1zgs | XMM | A | 450 |
| 1zgv | 501 | A1001 |  |
| 1zh9 | MPX | A | 500 |
| 1zha | PEP | A1268 |  |
| 1zha | R5P | A1269 |  |
| 1zhm | GSH | A1002 |  |
| 1zhp | GSH | A1002 |  |
| 1zhs | BMA | A | 203 |
| 1zht | HCR | A1001 |  |
| 1zhw | HC2 | A1001 |  |
| 1zhx | HC3 | A1001 |  |
| 1zhz | ERG | A1001 |  |
| 1zi5 | DR3 | A | 2 |
| 1zin | AP5 | A | 218 |
| 1ziw | NDG | A | 1 |
| 1ziw | NDG | A | 7 |
| 1zj1 | NLC | A | 1 |
| 1zj3 | DR2 | A | 1 |
| 1zji | 2PG | A1268 |  |
| 1zji | R5P | A1269 |  |
| 1zjp | DR4 | A | 1 |
| 1zjy | SS2 | A1260 |  |
| 1zk4 | AC0 | A1260 |  |
| 1zkk | SAH | A2800 |  |
| 1zkl | IBM | A | 503 |
| 1zky | 689 | A | 700 |
| 1zl2 | ANU | B7016 |  |
| 1zm2 | APR | E | 843 |
| 1zm7 | TTP | A | 300 |
| 1zmc | FAD | A | 480 |
| 1zmd | FAD | A | 480 |
| 1zmt | RNO | A1002 |  |
| 1zmx | THM | A1400 |  |
| 1zo8 | SNO | A5002 |  |
| 1zoe | K25 | A | 501 |
| 1zog | K37 | A | 501 |
| 1zoh | K44 | A | 501 |
| 1zos | MTM | A | 301 |
| 1zoy | EPH | D1306 |  |
| 1zoy | EPH | D1307 |  |
| 1zp0 | TTF | C | 308 |
| 1zp0 | TTF | D | 309 |
| 1zp5 | 2NI | A1000 |  |
| 1zp9 | ATP | A | 260 |
| 1zpb | 995 | A | 301 |
| 1zpd | CIT | A | 610 |
| 1zpl | MAG | A | 700 |
| 1zpu | NDG | A2018 |  |
| 1zq5 | E04 | A | 400 |
| 1zq9 | SAM | A4000 |  |
| 1zrk | 367 | A | 900 |
| 1zs0 | EIN | A | 994 |
| 1zsj | 709 | A | 301 |
| 1zsk | 421 | A | 301 |
| 1ztf | XYA | A | 300 |
| 1zth | ADP | A | 901 |
| 1zu0 | CBS | A | 901 |
| 1zua | TOL | X1320 |  |
| 1zuc | T98 | A | 201 |
| 1zvx | FIN | A | 994 |
| 1zw9 | H64 | A1001 |  |
| 1zwh | RDE | A1001 |  |
| 1zxc | IH6 | A | 478 |
| 1zxi | MCN | E3923 |  |
| 1zxm | ANP | A | 901 |
| 1zxm | ANP | B | 902 |
| 1zxn | ADP | A | 901 |
| 1zxv | MFM | A9002 |  |
| 1zy5 | ANP | A | 303 |
| 1zy8 | FAD | A4750 |  |
| 1zy8 | FAD | O4750 |  |
| 1zyd | ATP | A | 303 |
| 1zyj | BI5 | A | 362 |
| 1zys | 199 | A | 400 |
| 1zyt | MTN | A | 165 |
| 1zyu | ACP | A | 180 |
| 1zz1 | SHH | A2452 |  |
| 1zz2 | B11 | A | 362 |
| 1zzl | TZY | A | 999 |
| 1zzr | DP9 | A | 799 |
| 1zzr | HEM | A | 750 |
| 1zzs | DP9 | A | 799 |
| 1zzu | DP1 | A | 790 |
| 1zzu | HEM | A | 750 |
| 20gs | CBD | A | 211 |
| 256b | HEM | A | 109 |
| 2a06 | HEM | C | 501 |
| 2a06 | PEE | B4017 |  |
| 2a06 | PEE | C2007 |  |
| 2a06 | PEE | D2006 |  |
| 2a06 | PEE | Q3006 |  |
| 2a0c | CK9 | X | 500 |
| 2a14 | SAH | A4001 |  |
| 2a15 | NCA | A1001 |  |
| 2a19 | ANP | B1640 |  |
| 2a1l | PCW | A | 501 |
| 2a1t | FAD | R | 599 |
| 2a26 | CXS | B | 302 |
| 2a2c | ADP | A | 463 |
| 2a2d | A2G | A | 459 |
| 2a2g | LEO | A | 201 |
| 2a2z | DCZ | A | 304 |
| 2a2z | UDP | A | 301 |
| 2a30 | DCZ | A | 302 |
| 2a31 | PG3 | A | 501 |
| 2a3b | CFF | A1435 |  |
| 2a3i | C0R | A | 301 |
| 2a3r | LDP | A | 297 |
| 2a3x | CPK | C | 402 |
| 2a3y | CPJ | A | 610 |
| 2a4x | BLM | A | 700 |
| 2a4z | BYM | A | 101 |
| 2a5b | 8HG | A | 255 |
| 2a5h | PLP | A | 419 |
| 2a5u | QYT | A | 101 |
| 2a5y | ATP | C | 551 |
| 2a69 | RPT | C8001 |  |
| 2a69 | RPT | M8002 |  |
| 2a6c | CIT | B | 72 |
| 2a6h | STD | D8001 |  |
| 2a74 | NDG | A | 644 |
| 2a7p | 3IL | A | 410 |
| 2a7q | CFB | A | 328 |
| 2a81 | BCN | A | 501 |
| 2a84 | ATP | A2001 |  |
| 2a8h | 4NH | A | 158 |
| 2a8s | GTP | A | 250 |
| 2a8u | DR5 | A | 2 |
| 2a8x | FAD | A | 480 |
| 2a8y | MTA | A4003 |  |
| 2a98 | I3P | A | 1 |
| 2a9n | ORE | H | 149 |
| 2a9w | 2BR | B1819 |  |
| 2aa0 | MTP | A1001 |  |
| 2aa2 | AS4 | A | 201 |
| 2aa7 | 1CA | A | 201 |
| 2aac | FCB | A | 1 |
| 2aaq | FAD | A | 499 |
| 2aaw | DTL | C | 224 |
| 2ab2 | SNL | A | 502 |
| 2ab6 | GSM | A1218 |  |
| 2abe | HIS | A | 300 |
| 2abi | 1CA | A1001 |  |
| 2abj | CBC | A1401 |  |
| 2abm | PEE | A | 612 |
| 2abs | ACP | A1001 |  |
| 2ack | EDR | A | 999 |
| 2acl | L05 | B | 101 |
| 2aco | VCA | B | 501 |
| 2acq | G6P | A | 317 |
| 2acw | UPG | A | 900 |
| 2acx | ANP | A | 577 |
| 2acz | AT5 | C | 131 |
| 2acz | HEB | C | 130 |
| 2acz | OAA | A | 589 |
| 2ad5 | ADP | A | 601 |
| 2ad5 | ADP | B1601 |  |
| 2ad5 | CTP | A | 602 |
| 2adm | SAM | A | 500 |
| 2aex | CIT | A | 500 |
| 2af9 | MYR | A3045 |  |
| 2afi | ADP | E1292 |  |
| 2afk | ACP | E1292 |  |
| 2afu | BGT | A | 381 |
| 2ag2 | CH5 | B2341 |  |
| 2ag4 | OLA | A3628 |  |
| 2agd | UDH | A | 399 |
| 2age | SIN | A | 1 |
| 2agk | CIT | A1002 |  |
| 2agt | CIT | A | 450 |
| 2agt | FID | A | 320 |
| 2agv | BHQ | A1004 |  |
| 2agw | TSS | D3001 |  |
| 2ah0 | TSC | H | 1 |
| 2ah9 | CTO | A | 405 |
| 2ahx | NDG | A1004 |  |
| 2ai1 | P1G | A | 293 |
| 2ai2 | P1D | A | 293 |
| 2ai3 | P2G | A | 293 |
| 2aib | ERG | A | 475 |
| 2aid | THK | A | 201 |
| 2aio | MX1 | A | 1 |
| 2aiq | NDG | A | 901 |
| 2aj8 | SC3 | A1601 |  |
| 2aj8 | SC3 | A1608 |  |
| 2ajp | ANP | A | 501 |
| 2ajx | TGN | L | 501 |
| 2ajy | ECG | L | 304 |
| 2ajz | ECG | A | 302 |
| 2akr | CIS | A1001 |  |
| 2al1 | 2PG | A | 441 |
| 2al1 | PEP | A | 440 |
| 2al4 | CX6 | C | 801 |
| 2al5 | 4MP | A | 801 |
| 2alu | NDG | A | 691 |
| 2aly | YSA | A | 999 |
| 2am2 | 2LG | A1000 |  |
| 2am3 | UPG | A | 449 |
| 2am4 | U2F | A | 449 |
| 2am5 | UDP | A | 449 |
| 2amb | 17H | A1001 |  |
| 2an0 | HEM | A | 901 |
| 2an2 | HEM | A | 901 |
| 2an3 | CTL | A3001 |  |
| 2an5 | TTL | A1401 |  |
| 2an9 | GMP | A | 602 |
| 2anl | JE2 | A3151 |  |
| 2ano | 817 | A | 400 |
| 2ans | 2AN | A | 201 |
| 2aot | 2PM | A | 400 |
| 2aot | SAH | A | 500 |
| 2aou | CQA | A | 402 |
| 2aov | 4DI | A | 301 |
| 2aov | C2M | A | 401 |
| 2apg | FAD | A | 600 |
| 2aq6 | PLP | A | 401 |
| 2aqj | FAD | A | 600 |
| 2aqx | ATP | A1462 |  |
| 2ar8 | FAD | A | 600 |
| 2aru | ATP | A1065 |  |
| 2asf | CIT | A1001 |  |
| 2asm | RGA | A | 500 |
| 2aso | SPX | A | 500 |
| 2asp | RGC | A | 500 |
| 2at1 | MAL | A | 312 |
| 2ath | 3EA | A1001 |  |
| 2ati | IHU | A | 848 |
| 2atz | DGT | A | 201 |
| 2avn | SAI | A1300 |  |
| 2awh | B7G | A1920 |  |
| 2awh | VCA | A1001 |  |
| 2awx | HIS | B | 3 |
| 2ax6 | HFT | A | 1 |
| 2ax8 | FHM | A | 1 |
| 2ax9 | BHM | A | 1 |
| 2axa | FHM | A | 1 |
| 2axi | MPO | A | 201 |
| 2axn | ADP | A | 738 |
| 2axn | EDT | A | 737 |
| 2axn | F6P | A | 736 |
| 2axr | ABL | A | 505 |
| 2ay1 | AHC | B | 414 |
| 2ay2 | CXP | B | 414 |
| 2ay3 | MPP | A | 414 |
| 2ay4 | PPT | B | 414 |
| 2ay6 | 3IB | B | 414 |
| 2ay8 | 4TB | A | 414 |
| 2ay9 | 5PV | A | 414 |
| 2ayd | SIN | A | 401 |
| 2ayp | 43A | A | 270 |
| 2ays | A2G | A | 690 |
| 2azr | 982 | A | 301 |
| 2azy | CHD | A | 237 |
| 2azz | TCH | A | 131 |
| 2b00 | GCH | A | 254 |
| 2b04 | CHO | A | 200 |
| 2b0c | G1P | A | 496 |
| 2b0m | 201 | A | 401 |
| 2b15 | DNF | A | 130 |
| 2b1r | CBI | A | 245 |
| 2b1v | 458 | A | 201 |
| 2b1z | 17M | A | 201 |
| 2b36 | 5PP | A | 290 |
| 2b3b | BGC | C2607 |  |
| 2b3d | FAD | A1205 |  |
| 2b4b | B33 | B | 173 |
| 2b4r | AES | O | 601 |
| 2b4t | AES | P | 602 |
| 2b4z | HEM | A | 500 |
| 2b51 | UTP | A | 503 |
| 2b52 | D42 | A | 299 |
| 2b53 | D23 | A | 299 |
| 2b54 | D05 | A | 300 |
| 2b55 | D31 | A | 299 |
| 2b56 | UTP | A | 501 |
| 2b5j | 3AC | A | 562 |
| 2b5u | CIT | A | 602 |
| 2b60 | RIT | B | 100 |
| 2b65 | MAL | A3001 |  |
| 2b69 | UDP | A | 900 |
| 2b77 | 3CA | A | 240 |
| 2b7a | IZA | A2001 |  |
| 2b7n | NTM | A1001 |  |
| 2b7y | NDG | A | 803 |
| 2b8h | BMA | A | 505 |
| 2b8h | MAN | A | 506 |
| 2b8j | SPM | A | 653 |
| 2b8t | THM | A4970 |  |
| 2b8w | 5GP | A | 593 |
| 2b96 | ANN | A | 501 |
| 2b9a | FBC | A | 301 |
| 2b9f | ADP | A | 500 |
| 2b9h | ADP | A | 500 |
| 2b9i | ADP | A | 500 |
| 2b9j | ADP | A | 500 |
| 2b9w | FAD | A | 500 |
| 2b9x | FAD | A | 500 |
| 2b9y | FAD | A1500 |  |
| 2ba9 | FAD | A | 500 |
| 2bab | FAD | A1500 |  |
| 2bab | ODD | A1001 |  |
| 2bac | FAD | A | 500 |
| 2bac | ODT | A1001 |  |
| 2bal | PQA | A | 401 |
| 2ban | 357 | A | 562 |
| 2bb7 | QMS | A | 305 |
| 2bb9 | AKC | A | 401 |
| 2bbb | HH1 | A | 301 |
| 2bbf | 344 | A | 900 |
| 2bbo | ATP | A | 1 |
| 2bbs | ATP | A | 1 |
| 2bbt | ATP | A | 1 |
| 2bcg | GER | G2000 |  |
| 2bd0 | BIO | D | 900 |
| 2bdm | TMI | A | 501 |
| 2bdz | E64 | A | 501 |
| 2be2 | R22 | A4002 |  |
| 2be2 | SUC | A2000 |  |
| 2be2 | SUC | B2001 |  |
| 2be5 | TGT | D9001 |  |
| 2bed | 736 | B2001 |  |
| 2bel | CBO | A1270 |  |
| 2beo | ACM | B1239 |  |
| 2ber | SLB | A1648 |  |
| 2bes | RES | A | 200 |
| 2bet | DEZ | A | 200 |
| 2bex | MAK | B1461 |  |
| 2bf4 | FAD | A | 750 |
| 2bf6 | SIA | A1692 |  |
| 2bfa | CB3 | A1290 |  |
| 2bfd | TDP | A | 601 |
| 2bfq | APR | A1193 |  |
| 2bfy | H1N | A1357 |  |
| 2bgd | T1D | A1298 |  |
| 2bge | T2D | A1298 |  |
| 2bgi | HTG | A1274 |  |
| 2bgm | MAX | A6001 |  |
| 2bgs | NDP | A1321 |  |
| 2bhh | RYU | A1299 |  |
| 2bhi | SFT | A1061 |  |
| 2bhj | FC1 | A1499 |  |
| 2bhl | BG6 | A1510 |  |
| 2bhw | CHL | A | 609 |
| 2bhw | DGD | A | 802 |
| 2bhw | LHG | A | 801 |
| 2bhw | XAT | A | 504 |
| 2bhz | MAL | A1603 |  |
| 2bhz | MAL | A1606 |  |
| 2bhz | MAL | A1607 |  |
| 2bi7 | FAD | A1385 |  |
| 2bif | ANP | A | 500 |
| 2biw | 3ON | A1491 |  |
| 2bj0 | CXS | A1204 |  |
| 2bjf | DXC | A | 330 |
| 2bjf | TAU | A | 331 |
| 2bju | IH4 | A1330 |  |
| 2bk3 | FOH | A1503 |  |
| 2bk4 | RAS | A | 601 |
| 2bk9 | CXS | A1155 |  |
| 2bk9 | HEM | A1154 |  |
| 2bkl | ZAH | A1533 |  |
| 2bkm | HEM | A1132 |  |
| 2bku | GTP | A | 220 |
| 2bkv | PGA | A1242 |  |
| 2bkx | F6R | A1243 |  |
| 2bl2 | LHG | A1158 |  |
| 2bl2 | UMQ | A1162 |  |
| 2bl9 | CP6 | A1240 |  |
| 2blc | CP7 | A1240 |  |
| 2ble | 5GP | A1344 |  |
| 2bln | FON | A1305 |  |
| 2bm2 | PM2 | A3211 |  |
| 2bmb | PMM | A1865 |  |
| 2bmw | FAD | A1304 |  |
| 2bn4 | FAD | A | 750 |
| 2bnf | UTP | A1242 |  |
| 2bnn | FCN | A1199 |  |
| 2bog | BGC | X | 903 |
| 2bog | SGC | X | 902 |
| 2boi | MFU | A | 400 |
| 2bow | P4P | A | 162 |
| 2boy | BHO | A1256 |  |
| 2boz | D10 | H1252 |  |
| 2bpm | 529 | A1299 |  |
| 2bpo | FAD | A | 750 |
| 2bpy | 3IN | B | 902 |
| 2bq7 | IID | A1001 |  |
| 2bqw | IIE | B1246 |  |
| 2bqz | SAH | A1354 |  |
| 2br1 | PFP | A1277 |  |
| 2br4 | SAM | A | 301 |
| 2br5 | SAH | D | 301 |
| 2br6 | HSL | A1255 |  |
| 2bra | FAD | A1485 |  |
| 2brb | PFQ | A1277 |  |
| 2brc | CT5 | A1215 |  |
| 2bre | KJ2 | A1214 |  |
| 2brg | DFY | A1270 |  |
| 2brh | DFW | A1272 |  |
| 2brk | CMF | A1534 |  |
| 2brm | DFZ | A1275 |  |
| 2brn | DF1 | A1277 |  |
| 2bro | DF2 | A1277 |  |
| 2brs | SGN | A5702 |  |
| 2brv | MLA | X1892 |  |
| 2brv | MLA | X1894 |  |
| 2bry | FAD | A1493 |  |
| 2bs2 | FMR | A1657 |  |
| 2bs2 | HEM | F1255 |  |
| 2bs3 | CIT | A1657 |  |
| 2bs4 | DMW | E1244 |  |
| 2bs5 | BGC | A | 801 |
| 2bs7 | CBS | 11178 |  |
| 2bsl | DHB | B1313 |  |
| 2bsm | BSM | A1224 |  |
| 2bt9 | MFU | A1091 |  |
| 2bt9 | MFU | B1090 |  |
| 2btd | ADP | A1211 |  |
| 2bto | GTP | A1433 |  |
| 2btr | U73 | A | 300 |
| 2bts | U32 | A1299 |  |
| 2bu2 | ATP | A1386 |  |
| 2bu2 | TF1 | A1387 |  |
| 2bu3 | 3GC | A1240 |  |
| 2bu5 | TF1 | A1386 |  |
| 2bu6 | TF2 | A1386 |  |
| 2bu7 | TF3 | A1386 |  |
| 2bua | 007 | A1767 |  |
| 2bub | FPB | A1771 |  |
| 2buc | 008 | A1767 |  |
| 2bue | RIO | A1201 |  |
| 2bui | OCA | A | 905 |
| 2bur | PHB | B | 999 |
| 2buu | 4NC | B1542 |  |
| 2buv | DHB | B1542 |  |
| 2bv4 | MMA | A | 400 |
| 2bvc | ADP | A | 501 |
| 2bvj | HEM | A1408 |  |
| 2bvl | UDP | A1545 |  |
| 2bvr | 4CP | H1246 |  |
| 2bvw | CTT | A | 600 |
| 2bvx | 5CB | H1246 |  |
| 2bw7 | APC | A2200 |  |
| 2bw7 | ECS | A2203 |  |
| 2bwn | SIN | A1398 |  |
| 2bwo | SCA | A | 500 |
| 2bx8 | AZQ | A2002 |  |
| 2bxa | C1F | A2001 |  |
| 2bxf | DZP | A2001 |  |
| 2bxg | IBP | A2001 |  |
| 2bxh | IOS | A1001 |  |
| 2bxh | IOS | A1002 |  |
| 2bxi | AZQ | A2002 |  |
| 2bxk | AZQ | A2002 |  |
| 2bxk | MYR | A1001 |  |
| 2bxl | DIU | A3001 |  |
| 2bxl | MYR | A1001 |  |
| 2bxm | MYR | A1001 |  |
| 2bxn | IDB | A2001 |  |
| 2bxn | MYR | A1001 |  |
| 2bxo | MYR | A1001 |  |
| 2bxo | OPB | A2001 |  |
| 2bxp | MYR | A1001 |  |
| 2bxp | P1Z | A3001 |  |
| 2bxq | MYR | A1001 |  |
| 2bxr | MLG | A | 601 |
| 2bxs | MLG | A | 601 |
| 2bxt | C2D | H1246 |  |
| 2bxu | C1D | H1246 |  |
| 2by4 | ACP | A1996 |  |
| 2byb | DPK | A | 601 |
| 2byh | 2D7 | A1226 |  |
| 2byi | 2DD | A1226 |  |
| 2byo | HXA | A1215 |  |
| 2byo | LNL | A1216 |  |
| 2byo | MLT | A1219 |  |
| 2byo | OAA | A1218 |  |
| 2bz0 | G2P | A1176 |  |
| 2bz1 | TAU | A1179 |  |
| 2bz6 | 346 | H1258 |  |
| 2bzg | B3P | A1247 |  |
| 2bzz | AP5 | A2135 |  |
| 2c0k | HEM | A1152 |  |
| 2c12 | SPM | B1433 |  |
| 2c1b | CQP | A1351 |  |
| 2c1l | TAR | A1365 |  |
| 2c1x | B3P | A1458 |  |
| 2c1z | KMP | A1456 |  |
| 2c1z | U2F | A1457 |  |
| 2c25 | SIA | A | 600 |
| 2c2b | SAM | A | 500 |
| 2c2n | AE4 | A1346 |  |
| 2c2n | DXE | A1347 |  |
| 2c2z | DTD | B1480 |  |
| 2c2z | PHQ | C | 1 |
| 2c31 | ADP | A1555 |  |
| 2c37 | RP5 | N | 405 |
| 2c39 | ADP | B | 404 |
| 2c39 | ADP | H | 404 |
| 2c3i | IYZ | B1306 |  |
| 2c3k | ABO | A1271 |  |
| 2c3l | IDZ | A1274 |  |
| 2c42 | PYR | A3237 |  |
| 2c47 | 5ID | A1300 |  |
| 2c49 | ANP | A1304 |  |
| 2c4c | FAD | A | 600 |
| 2c4i | BTN | A1274 |  |
| 2c4k | TAM | B1353 |  |
| 2c4t | SA8 | A1299 |  |
| 2c4v | CIT | A1159 |  |
| 2c53 | DUR | A1245 |  |
| 2c56 | SUC | A1245 |  |
| 2c5b | 5F1 | A | 500 |
| 2c5n | CK8 | A1297 |  |
| 2c5o | CK2 | A1297 |  |
| 2c5u | APC | A1375 |  |
| 2c5y | MTW | A1298 |  |
| 2c66 | RM2 | A1503 |  |
| 2c68 | CT6 | A1297 |  |
| 2c69 | CT8 | A1299 |  |
| 2c6c | 24I | A1767 |  |
| 2c6d | ANP | A1391 |  |
| 2c6h | HEM | A1407 |  |
| 2c6i | DT1 | A1299 |  |
| 2c6k | DT2 | A1299 |  |
| 2c6l | DT4 | A1299 |  |
| 2c6m | DT5 | A1297 |  |
| 2c6q | IMP | A1337 |  |
| 2c6q | NDP | A1338 |  |
| 2c75 | RSA | A | 601 |
| 2c7g | FAD | A | 501 |
| 2c7g | ODP | A | 502 |
| 2c7v | MTX | A1272 |  |
| 2c7x | HEM | A1407 |  |
| 2c88 | ACP | A1996 |  |
| 2c8a | NCA | A1252 |  |
| 2c8k | ACP | A1996 |  |
| 2c8m | LPA | A | 900 |
| 2c8y | C3M | B1252 |  |
| 2c93 | C4M | B1251 |  |
| 2c97 | JCL | A | 701 |
| 2c97 | JCL | E | 701 |
| 2c9e | DGD | A1332 |  |
| 2c9o | ADP | A1450 |  |
| 2c9z | QUE | A1458 |  |
| 2ca0 | HEM | A1407 |  |
| 2cad | CIT | B1150 |  |
| 2cb8 | MYA | A1088 |  |
| 2cbo | TH2 | A | 120 |
| 2cbr | A80 | A | 201 |
| 2cbt | TH2 | A1120 |  |
| 2cbu | CTS | A1447 |  |
| 2cbv | CGB | A1447 |  |
| 2cbz | ATP | A1873 |  |
| 2ccc | LFN | A1067 |  |
| 2ccg | TMP | A1205 |  |
| 2ccj | TMP | A1209 |  |
| 2ccr | B4G | A1398 |  |
| 2ccs | 4BH | A1224 |  |
| 2cct | 2E1 | A1224 |  |
| 2ccu | 2D9 | A1224 |  |
| 2ccv | A2G | A1101 |  |
| 2cd8 | HEM | A | 419 |
| 2cdb | BGC | A1368 |  |
| 2cdb | BGC | B1368 |  |
| 2cdc | XYS | A1368 |  |
| 2cdc | XYS | B1370 |  |
| 2cdq | SAM | A1500 |  |
| 2cdq | TAR | A | 700 |
| 2cdu | ADP | A | 501 |
| 2ce2 | XY2 | X | 190 |
| 2cex | DAN | B1307 |  |
| 2cf8 | SIN | I | 1 |
| 2cfc | KPC | A1252 |  |
| 2cft | PLP | A1296 |  |
| 2cfu | 1DB | A1655 |  |
| 2cfz | 1DO | A1655 |  |
| 2cgf | P2N | A1214 |  |
| 2cgo | FMR | A | 351 |
| 2cgu | 3A3 | A1272 |  |
| 2cgv | 3B3 | A1273 |  |
| 2cgz | TNR | A1102 |  |
| 2ch2 | KY1 | B1391 |  |
| 2ch5 | NDG | D | 500 |
| 2ch9 | FUL | A1152 |  |
| 2cha | TSU | C | 51 |
| 2chh | BMA | A1118 |  |
| 2chh | MAN | A1117 |  |
| 2chn | NGT | A1718 |  |
| 2cht | TSA | A | 203 |
| 2chw | 039 | A2093 |  |
| 2chz | 093 | A2093 |  |
| 2ci0 | 1CM | A1450 |  |
| 2ci1 | CIT | A1282 |  |
| 2ci5 | HCS | A1281 |  |
| 2cib | CM6 | A1450 |  |
| 2cip | ZZ1 | A1285 |  |
| 2cir | BG6 | A | 501 |
| 2cit | G2F | A1283 |  |
| 2ciw | HEM | A1300 |  |
| 2ciw | MAN | A1306 |  |
| 2cjf | RP4 | A1151 |  |
| 2cji | GSK | A1246 |  |
| 2cjp | VPR | B1322 |  |
| 2cju | PHX | H1114 |  |
| 2ck3 | ADP | D | 600 |
| 2ck3 | ANP | A | 600 |
| 2cl4 | HEM | X | 251 |
| 2cl5 | BIE | A1218 |  |
| 2cl6 | XY2 | X | 332 |
| 2cl7 | GTP | X1167 |  |
| 2cl8 | BGC | A1247 |  |
| 2clc | GTP | X1167 |  |
| 2cle | F6F | A1268 |  |
| 2clf | F6F | B1396 |  |
| 2clk | G3H | A1268 |  |
| 2cll | F9F | A1268 |  |
| 2clo | F19 | A1268 |  |
| 2clx | F18 | A1299 |  |
| 2cm4 | RCL | A1170 |  |
| 2cm7 | IZD | A1299 |  |
| 2cm8 | F16 | A1299 |  |
| 2cmc | DFM | A1281 |  |
| 2cme | D10 | B1099 |  |
| 2cml | BMA | A1709 |  |
| 2cml | MAN | B2484 |  |
| 2cml | ZMR | B2477 |  |
| 2cmm | POR | A | 155 |
| 2cmn | HEM | A1271 |  |
| 2cmw | OLP | A1340 |  |
| 2cn0 | F25 | H1246 |  |
| 2cn3 | BGC | A1764 |  |
| 2cn3 | XYS | A1765 |  |
| 2cn3 | XYS | A1766 |  |
| 2cn5 | ADP | A1509 |  |
| 2cn8 | DBQ | A1506 |  |
| 2cnq | ADP | A1307 |  |
| 2cnq | SIN | A1312 |  |
| 2cnw | 5GP | D3001 |  |
| 2cqs | BGC | A1901 |  |
| 2csg | SIN | A | 502 |
| 2csn | CKI | A | 300 |
| 2ctc | HFA | A | 309 |
| 2cu0 | XMP | A1001 |  |
| 2cu0 | XMP | B1002 |  |
| 2cul | FAD | A | 301 |
| 2cvd | GSH | A2001 |  |
| 2cvd | HQL | A2201 |  |
| 2cvt | ANP | A | 890 |
| 2cvu | ANP | A1001 |  |
| 2cvv | ANP | A1001 |  |
| 2cvv | UDP | A1002 |  |
| 2cvw | TTP | A1001 |  |
| 2cvx | ADP | A1002 |  |
| 2cvx | DGT | A1001 |  |
| 2cvy | TTP | A1001 |  |
| 2cvz | NDP | A1302 |  |
| 2cw6 | 3HG | A | 399 |
| 2cwg | SIA | D | 4 |
| 2cwh | NDP | A2510 |  |
| 2cwh | PYC | A2520 |  |
| 2cx8 | SAH | A | 300 |
| 2cx8 | SAH | B | 400 |
| 2cxo | DER | A1601 |  |
| 2cxp | A5P | A1601 |  |
| 2cxq | S6P | A1601 |  |
| 2cxs | F6P | A1601 |  |
| 2cyd | LHG | A | 558 |
| 2cyd | UMQ | C1001 |  |
| 2cyf | MAL | A | 240 |
| 2cz1 | BUA | A1301 |  |
| 2cz2 | GSH | A1001 |  |
| 2cz5 | CIT | A | 401 |
| 2cz8 | FAD | A1201 |  |
| 2czq | CIT | A | 501 |
| 2d0f | BGC | A | 803 |
| 2d0g | BGC | A | 801 |
| 2d0g | BGC | A | 910 |
| 2d0k | FOL | A1161 |  |
| 2d0o | ADP | A1001 |  |
| 2d1e | BLA | A1130 |  |
| 2d1j | D01 | A | 700 |
| 2d1r | OLU | A2001 |  |
| 2d20 | NPO | A | 453 |
| 2d20 | XYS | A | 451 |
| 2d22 | XYS | A | 451 |
| 2d23 | XYS | A | 451 |
| 2d24 | XYS | A | 451 |
| 2d2c | BNT | B | 309 |
| 2d2g | DZZ | A | 901 |
| 2d2v | MAL | A | 245 |
| 2d32 | ANP | A1521 |  |
| 2d3a | ADP | A6001 |  |
| 2d3b | ANP | A6001 |  |
| 2d3c | ADP | A6001 |  |
| 2d3s | TNR | A | 401 |
| 2d3u | CCT | A1001 |  |
| 2d3z | FIH | A2001 |  |
| 2d41 | SNH | A1001 |  |
| 2d44 | XYS | A | 702 |
| 2d4h | 5GP | A | 593 |
| 2d4n | DUP | A | 777 |
| 2d5m | FMN | A1001 |  |
| 2d5x | L35 | A1200 |  |
| 2d5y | IVA | A | 414 |
| 2d61 | MAE | A | 414 |
| 2d63 | IVA | A | 414 |
| 2d64 | IVA | A | 414 |
| 2d7f | MMA | A | 238 |
| 2d7h | DCM | A | 106 |
| 2d7i | UDP | A | 705 |
| 2d7r | TNR | A | 707 |
| 2d7r | UDP | A | 705 |
| 2d7z | MAE | A | 414 |
| 2daa | DCS | A | 285 |
| 2db4 | LHG | A | 558 |
| 2db4 | UMQ | A1163 |  |
| 2dbl | S5H | L | 212 |
| 2dc1 | CIT | A1585 |  |
| 2dc1 | CIT | B2585 |  |
| 2dcf | ACA | A | 501 |
| 2dcn | CKP | A2001 |  |
| 2dcy | TAR | A | 504 |
| 2dd4 | FRU | B3008 |  |
| 2dd4 | TAR | C3401 |  |
| 2dd7 | CXS | B | 501 |
| 2ddf | CIT | B | 500 |
| 2ddh | HXD | A | 900 |
| 2de3 | OBP | A1366 |  |
| 2de4 | BPS | A1366 |  |
| 2de7 | 9CA | B2001 |  |
| 2deb | PLM | A2351 |  |
| 2dfd | HIS | A3301 |  |
| 2dfd | MLT | A3104 |  |
| 2dhr | ADP | A1001 |  |
| 2djh | UM3 | A | 118 |
| 2djl | FMN | A1350 |  |
| 2djl | NCO | A1351 |  |
| 2djl | SIN | A1370 |  |
| 2djl | SIN | B2370 |  |
| 2djx | FMN | A1350 |  |
| 2djx | NCO | A1351 |  |
| 2dkc | 16G | A | 701 |
| 2dld | OXM | A | 402 |
| 2dm6 | TAM | B5000 |  |
| 2dn1 | MBN | A1001 |  |
| 2doo | C4H | A | 601 |
| 2dpm | SAM | A | 300 |
| 2dpt | PUY | A | 501 |
| 2dq0 | SSA | A1001 |  |
| 2dq3 | SSA | A1001 |  |
| 2dqa | BGC | B2001 |  |
| 2dqm | BES | A | 901 |
| 2dqs | ACP | A1002 |  |
| 2dqy | CHD | A | 1 |
| 2dqz | PLM | A | 111 |
| 2dri | RIP | A | 272 |
| 2ds0 | SIA | A | 280 |
| 2ds1 | 1CD | A | 501 |
| 2dsa | HPX | A1225 |  |
| 2dsf | LXC | A3937 |  |
| 2dt8 | PLM | A | 701 |
| 2dtj | CIT | B | 501 |
| 2dto | ATP | A1301 |  |
| 2dto | BTN | A1401 |  |
| 2dtx | BMA | A | 301 |
| 2du2 | FMN | B1512 |  |
| 2du8 | FAD | B1351 |  |
| 2dur | MAN | A | 13 |
| 2duv | 371 | A | 501 |
| 2dvc | SUC | A1001 |  |
| 2dvl | FAD | A1001 |  |
| 2dvn | IMP | A1301 |  |
| 2dw0 | GM6 | A | 901 |
| 2dw1 | GM6 | A | 2 |
| 2dw2 | MAN | A | 805 |
| 2dw6 | TAR | A1001 |  |
| 2dwc | ADP | A | 501 |
| 2dwi | CBI | A | 695 |
| 2dwo | PEP | A | 701 |
| 2dwp | ACP | A | 700 |
| 2dwp | F6P | A | 720 |
| 2dwu | DGL | A | 278 |
| 2dx7 | CIT | A | 501 |
| 2dxd | ANP | A | 501 |
| 2dxr | SOR | A1001 |  |
| 2dxs | JTP | A1000 |  |
| 2dxt | ATP | A1301 |  |
| 2dxt | ATP | B1302 |  |
| 2dxt | BTN | A1401 |  |
| 2dy5 | 224 | A | 301 |
| 2dya | ADP | A | 501 |
| 2dza | PAB | B1301 |  |
| 2dzb | HH2 | A1301 |  |
| 2e0a | ANP | A | 501 |
| 2e0p | CTT | A | 800 |
| 2e14 | FRR | A | 361 |
| 2e1m | FAD | A | 801 |
| 2e1q | SAL | A2006 |  |
| 2e1t | MLC | A1001 |  |
| 2e1w | FR6 | A | 1 |
| 2e21 | ANP | A | 500 |
| 2e22 | MAN | A1001 |  |
| 2e2o | BGC | A | 400 |
| 2e2p | ADP | A | 600 |
| 2e2r | 2OH | A1401 |  |
| 2e2y | HEM | A | 154 |
| 2e33 | BMA | B1003 |  |
| 2e33 | MAN | B1004 |  |
| 2e3t | URC | A4001 |  |
| 2e3x | GM6 | A | 700 |
| 2e41 | BTX | A1301 |  |
| 2e48 | FAD | B1351 |  |
| 2e49 | FAD | B1351 |  |
| 2e4a | FAD | B1351 |  |
| 2e56 | MYR | A | 501 |
| 2e5d | NCA | A1501 |  |
| 2e5v | FAD | A1002 |  |
| 2e5y | ATP | A1001 |  |
| 2e68 | DOR | A1353 |  |
| 2e68 | DOR | B2353 |  |
| 2e68 | FMN | A1350 |  |
| 2e68 | NCO | A1352 |  |
| 2e6a | NCO | A1351 |  |
| 2e6d | FMN | A1350 |  |
| 2e6d | FMR | A1352 |  |
| 2e6d | FMR | B2352 |  |
| 2e6d | NCO | A1351 |  |
| 2e6f | FMN | A1350 |  |
| 2e6f | NCO | A1351 |  |
| 2e6f | OXC | A1352 |  |
| 2e6f | OXC | B2352 |  |
| 2e6v | BMA | A | 1 |
| 2e74 | HEM | A | 303 |
| 2e74 | UMQ | A1101 |  |
| 2e74 | UMQ | A1102 |  |
| 2e74 | UMQ | A1104 |  |
| 2e75 | HEM | C | 301 |
| 2e75 | QNO | A | 501 |
| 2e76 | HEM | C | 301 |
| 2e77 | FMN | B1512 |  |
| 2e77 | PYR | A4442 |  |
| 2e77 | PYR | B1442 |  |
| 2e7y | PGO | A | 601 |
| 2e82 | FAD | B1351 |  |
| 2e82 | IM3 | A | 352 |
| 2e82 | IM3 | B1352 |  |
| 2e89 | ATP | A | 900 |
| 2e8a | ANP | A | 601 |
| 2e8t | FPS | A1201 |  |
| 2e8v | GRG | A1501 |  |
| 2e8x | GPP | A1001 |  |
| 2e93 | B29 | A | 901 |
| 2e98 | B29 | A | 501 |
| 2e99 | B08 | A | 505 |
| 2e9a | B28 | A | 701 |
| 2e9d | B76 | A | 701 |
| 2e9l | BGC | A1401 |  |
| 2e9l | OLA | A2001 |  |
| 2e9l | PLM | A1001 |  |
| 2e9o | A58 | A1001 |  |
| 2e9p | 77A | A1001 |  |
| 2e9u | A25 | A1001 |  |
| 2ea2 | F77 | A | 482 |
| 2eaa | CIT | A1285 |  |
| 2eaa | CIT | B1284 |  |
| 2eae | FUL | A | 900 |
| 2eal | A2G | A | 151 |
| 2eas | CZA | A1005 |  |
| 2eat | CZA | A1005 |  |
| 2eau | CZA | A1005 |  |
| 2eb3 | ANP | A2001 |  |
| 2eco | 4MV | A | 414 |
| 2ed4 | FAD | A | 400 |
| 2eeo | FMR | B | 501 |
| 2eeo | PYR | B | 25 |
| 2ef6 | MDM | A1002 |  |
| 2efe | GNH | B1200 |  |
| 2efj | SAH | A | 501 |
| 2egv | SAM | A1300 |  |
| 2egw | SAH | A | 300 |
| 2ehz | MCT | A1600 |  |
| 2ei0 | BP7 | A | 400 |
| 2ei1 | D1N | A1637 |  |
| 2ei3 | BPY | A | 350 |
| 2ei6 | D92 | A | 700 |
| 2ei7 | D93 | A | 700 |
| 2eiy | 4MV | A2414 |  |
| 2ej0 | PMP | A3413 |  |
| 2ej2 | PPE | A3413 |  |
| 2ejf | BTN | C1400 |  |
| 2ejg | BTN | A1401 |  |
| 2ejt | SAM | A | 501 |
| 2eju | SAH | A | 501 |
| 2ek9 | BES | A2001 |  |
| 2ekg | FAD | A2001 |  |
| 2eo7 | CTT | A | 801 |
| 2epn | NGT | A1650 |  |
| 2eq6 | FAD | A1482 |  |
| 2eq8 | FAD | A4482 |  |
| 2eq9 | FAD | A8482 |  |
| 2erp | GM6 | A1002 |  |
| 2erz | HFS | E | 351 |
| 2esm | M77 | A | 416 |
| 2et1 | GLV | A | 203 |
| 2etk | HFS | A | 416 |
| 2etm | 7PY | A | 131 |
| 2etr | Y27 | A | 416 |
| 2eu8 | AP5 | A1218 |  |
| 2eua | TAR | A1000 |  |
| 2eud | ANP | A | 890 |
| 2eug | URA | A | 230 |
| 2eum | OCA | A | 302 |
| 2eus | ABN | A | 400 |
| 2ev1 | OLA | A1002 |  |
| 2ev2 | OLA | A1001 |  |
| 2ev3 | OLA | A1001 |  |
| 2ev4 | OLA | A1001 |  |
| 2evc | FC3 | A | 999 |
| 2evd | D10 | A | 304 |
| 2eve | MPO | A | 301 |
| 2evl | LNK | A | 304 |
| 2evm | FC2 | A | 999 |
| 2evo | CT0 | A | 503 |
| 2ewb | ZED | A2000 |  |
| 2ewd | A3D | A | 401 |
| 2ewd | PYR | A | 901 |
| 2ewn | BTX | B | 500 |
| 2ews | ANP | A1001 |  |
| 2ex4 | SAH | A1401 |  |
| 2exc | JNK | X | 600 |
| 2exj | XYS | A2005 |  |
| 2exj | XYS | C2010 |  |
| 2exm | ZIP | A | 400 |
| 2ez8 | PYR | A1615 |  |
| 2ez9 | FAD | A1612 |  |
| 2ezu | HTL | A1614 |  |
| 2f01 | BTN | A5100 |  |
| 2f01 | BTQ | A5101 |  |
| 2f06 | HIS | A | 152 |
| 2f11 | IEM | A | 382 |
| 2f17 | PYI | A | 902 |
| 2f17 | PYI | B2202 |  |
| 2f1g | GNF | A1002 |  |
| 2f2c | AP9 | B | 401 |
| 2f2g | HMH | A1300 |  |
| 2f2h | MPO | B3010 |  |
| 2f2h | XTG | A3015 |  |
| 2f2h | XTG | F3021 |  |
| 2f2l | CIT | X | 301 |
| 2f2l | MLD | X | 201 |
| 2f2t | 5IQ | A | 463 |
| 2f2u | M77 | A | 501 |
| 2f38 | 15M | A | 325 |
| 2f3x | MLC | A | 391 |
| 2f48 | FBP | A | 829 |
| 2f4b | EHA | A | 201 |
| 2f59 | INI | A | 201 |
| 2f5t | MAL | X | 343 |
| 2f5z | FAD | A | 480 |
| 2f64 | 12Q | A | 472 |
| 2f69 | SAH | A | 800 |
| 2f6m | DDQ | A | 101 |
| 2f6m | DDQ | B | 119 |
| 2f6t | 1C2 | A | 608 |
| 2f6u | CIT | A3001 |  |
| 2f6v | SK2 | A | 608 |
| 2f6w | UN3 | A | 608 |
| 2f6y | ENT | A | 608 |
| 2f7e | 2EA | E | 351 |
| 2f7i | 26C | A | 326 |
| 2f7p | 2SK | A5009 |  |
| 2f7x | 4EA | E | 351 |
| 2f7z | 6EA | E1000 |  |
| 2f89 | 210 | F9001 |  |
| 2f8a | MLA | A | 501 |
| 2f8i | 205 | A1002 |  |
| 2f8l | SAM | A | 400 |
| 2f92 | AHD | F9001 |  |
| 2f94 | BFQ | F9001 |  |
| 2f99 | AKV | A1335 |  |
| 2f9b | N1H | H | 258 |
| 2f9g | ADP | A | 500 |
| 2fa1 | BDF | B | 1 |
| 2fah | MLA | A2001 |  |
| 2fai | 459 | A | 101 |
| 2faq | ATP | A1304 |  |
| 2far | DTP | A1304 |  |
| 2fb3 | GTP | A | 404 |
| 2fbw | BHG | C | 142 |
| 2fbw | CBE | P | 202 |
| 2fbw | HEM | P | 201 |
| 2fbw | PEE | D | 109 |
| 2fbz | H2B | X | 903 |
| 2fcp | GP1 | A | 902 |
| 2fcp | GP4 | A | 901 |
| 2fd6 | NDG | U | 400 |
| 2fdj | SIN | A | 401 |
| 2fdu | D1G | A | 501 |
| 2fdv | D2G | A | 501 |
| 2fdw | D3G | A | 501 |
| 2fdy | D4G | A | 501 |
| 2ffa | ADP | A | 708 |
| 2ffb | ADP | A | 708 |
| 2ffq | GSP | A | 355 |
| 2ffu | UDP | A | 601 |
| 2fgh | ATP | A5380 |  |
| 2fgj | ATP | A | 800 |
| 2fgk | ATP | A | 800 |
| 2fgl | LXC | B | 708 |
| 2fgq | MLT | X | 700 |
| 2fh8 | BGC | A1098 |  |
| 2fhi | IB2 | A | 301 |
| 2fhl | BNI | A | 501 |
| 2fhl | BNI | B | 502 |
| 2fhn | BNI | Y | 502 |
| 2fhy | A37 | A | 502 |
| 2fie | A74 | A | 701 |
| 2fik | PLM | A | 502 |
| 2fix | 870 | A | 701 |
| 2fja | ADX | C3200 |  |
| 2fje | FAD | A1000 |  |
| 2fjp | S14 | A1001 |  |
| 2fkm | G16 | X | 464 |
| 2fky | N2T | A | 604 |
| 2fl2 | N4T | A | 604 |
| 2fl6 | N5T | A | 604 |
| 2flb | 6NH | H1246 |  |
| 2flq | HEM | A | 901 |
| 2flr | 7NH | H | 258 |
| 2fm3 | PYR | A | 402 |
| 2fme | 3QC | A | 370 |
| 2fmy | HEM | A | 300 |
| 2fmz | DPN | A | 300 |
| 2fn1 | SAL | A | 506 |
| 2fnn | 4MZ | A | 264 |
| 2fnu | UD1 | A | 902 |
| 2fnw | REP | A | 801 |
| 2fnz | OXM | A | 402 |
| 2fon | FAD | A1000 |  |
| 2fp2 | TSA | B | 500 |
| 2fp4 | GTP | B | 403 |
| 2fp9 | TAR | A | 345 |
| 2fpb | TSS | A1001 |  |
| 2fpk | ADP | A | 401 |
| 2fpl | ANP | A | 401 |
| 2fpm | ANP | A | 401 |
| 2fpt | ILB | A | 405 |
| 2fpv | ILC | A | 405 |
| 2fpy | ILF | A | 407 |
| 2fqe | CIT | A | 701 |
| 2fqi | ILH | A | 600 |
| 2fqx | GMP | A | 400 |
| 2fr6 | CTN | A1001 |  |
| 2fr6 | URI | B1002 |  |
| 2fsg | ATP | A | 900 |
| 2fsg | ATP | B | 901 |
| 2fsh | ANP | A | 900 |
| 2fsh | ANP | B | 901 |
| 2fsi | ADP | A | 900 |
| 2fsi | ADP | B | 901 |
| 2fsn | ADP | A | 328 |
| 2fto | CB3 | X | 266 |
| 2fto | TMP | X | 265 |
| 2fu7 | PHN | A | 410 |
| 2fu9 | MP2 | A1410 |  |
| 2fue | M1P | A | 400 |
| 2fug | FMN | 7 | 500 |
| 2fut | H1S | A | 801 |
| 2fv0 | BGC | A | 401 |
| 2fv1 | NDG | A | 401 |
| 2fv5 | 541 | A | 1 |
| 2fvc | 888 | A | 901 |
| 2fvk | DUC | A | 604 |
| 2fvy | BGC | A | 310 |
| 2fw0 | CIT | A | 314 |
| 2fw0 | MLA | A | 315 |
| 2fwn | TDP | A | 530 |
| 2fwt | HEM | A | 803 |
| 2fx6 | 270 | A | 246 |
| 2fxs | RDA | A1001 |  |
| 2fxu | ATP | A | 401 |
| 2fy3 | CHT | A2000 |  |
| 2fyt | SAH | A | 549 |
| 2fyu | FDN | C | 400 |
| 2fzd | TOL | A | 317 |
| 2fze | APR | A1377 |  |
| 2g01 | 73Q | A | 901 |
| 2g01 | 73Q | B1001 |  |
| 2g0b | NLT | A | 400 |
| 2g0g | SP0 | A | 101 |
| 2g19 | 4HG | A | 801 |
| 2g1m | 4HG | A | 701 |
| 2g1n | 1IG | A | 885 |
| 2g1o | 2IG | A | 885 |
| 2g1q | N9H | A | 604 |
| 2g1r | 3IG | A | 885 |
| 2g1s | 4IG | A | 885 |
| 2g1y | 5IG | A | 885 |
| 2g24 | 7IG | A | 334 |
| 2g2h | P16 | A | 532 |
| 2g2i | ADP | A | 820 |
| 2g2r | TNS | A1005 |  |
| 2g3h | HEM | A | 154 |
| 2g44 | T3O | A | 700 |
| 2g50 | PYR | A | 600 |
| 2g58 | PHQ | B | 1 |
| 2g5f | PYR | A2001 |  |
| 2g5g | HEM | X | 605 |
| 2g5i | ADP | B | 601 |
| 2g5n | 23M | A | 301 |
| 2g5o | DRQ | A | 201 |
| 2g5u | PCQ | A | 240 |
| 2g5v | 22M | A | 301 |
| 2g6f | NCO | X | 200 |
| 2g6i | HBI | A | 760 |
| 2g6j | HEM | A | 750 |
| 2g6p | HM2 | A | 410 |
| 2g70 | HNT | A5001 |  |
| 2g71 | FTS | A3001 |  |
| 2g72 | F21 | A4001 |  |
| 2g72 | SAM | A2001 |  |
| 2g76 | MLT | A | 503 |
| 2g7z | HXA | A9136 |  |
| 2g88 | CIT | A1322 |  |
| 2g8m | CB3 | A | 702 |
| 2g8n | F83 | A2001 |  |
| 2g8o | CB3 | A | 902 |
| 2g8t | MI2 | A | 401 |
| 2g93 | MMA | A | 687 |
| 2g9r | G27 | A | 998 |
| 2g9v | IFM | A | 998 |
| 2g9x | NU5 | A | 299 |
| 2ga2 | A19 | A | 482 |
| 2ga4 | ADE | A | 701 |
| 2ga9 | SAP | D | 482 |
| 2gb4 | SAH | A | 300 |
| 2gbb | CIT | A | 191 |
| 2gbc | NDG | A1090 |  |
| 2gbi | XIH | A | 901 |
| 2gc0 | PAN | A | 901 |
| 2gc1 | S6P | A | 502 |
| 2gc2 | FPC | A | 501 |
| 2gc3 | M6P | A | 501 |
| 2gc8 | 885 | A | 579 |
| 2gc9 | CIT | A | 181 |
| 2gd8 | PO1 | A | 264 |
| 2gdo | 12C | A | 298 |
| 2gfk | VII | A1410 |  |
| 2gfs | PQB | A | 361 |
| 2gfx | PMN | A1001 |  |
| 2ggh | NLQ | C1376 |  |
| 2ggn | HEM | X | 251 |
| 2ggq | TTP | A | 500 |
| 2ggu | MLR | A3505 |  |
| 2gh5 | FAD | A | 479 |
| 2gh6 | CF3 | A9452 |  |
| 2ghc | HEM | X | 251 |
| 2ghd | HEM | X | 251 |
| 2ghe | HEM | X | 251 |
| 2gil | GTP | A1200 |  |
| 2giq | NN2 | A1001 |  |
| 2gir | NN3 | A1001 |  |
| 2giu | FBR | A | 600 |
| 2gj3 | FAD | A | 500 |
| 2gj4 | 2TH | A | 949 |
| 2gjl | FMN | A1904 |  |
| 2gjx | NDG | B | 8 |
| 2gk6 | ADP | A1001 |  |
| 2gks | ADP | A | 900 |
| 2gks | ADP | A | 902 |
| 2gl6 | ADP | A1009 |  |
| 2glp | BDE | A3001 |  |
| 2glu | SAM | A | 301 |
| 2gm9 | 3TH | A | 1 |
| 2gmh | BHG | A | 616 |
| 2gmh | FAD | A | 611 |
| 2gmj | FAD | A | 611 |
| 2gmx | 877 | A | 901 |
| 2gmx | 877 | B1001 |  |
| 2go3 | PLM | A | 701 |
| 2go4 | TUX | A | 901 |
| 2goy | ADX | B | 310 |
| 2goy | ADX | H | 316 |
| 2gp5 | AKG | A | 501 |
| 2gpp | 1BA | A | 459 |
| 2gq3 | MLT | A | 900 |
| 2gq9 | HBA | A2760 |  |
| 2gqt | FAD | A | 601 |
| 2gqw | FAD | A1449 |  |
| 2gr0 | ADP | A1001 |  |
| 2gr2 | APR | A1500 |  |
| 2grj | ADP | A | 200 |
| 2gs7 | ANP | A | 301 |
| 2gsk | CNC | A | 701 |
| 2gss | EAA | A | 0 |
| 2gtk | 208 | A1001 |  |
| 2gu5 | NLP | A3808 |  |
| 2gu8 | 796 | A1001 |  |
| 2gud | BMA | A | 123 |
| 2gud | MAN | A | 122 |
| 2guf | MPG | A | 701 |
| 2gup | SUC | A | 290 |
| 2gvd | 128 | A | 584 |
| 2gvj | DGB | A | 502 |
| 2gvy | NDG | B | 500 |
| 2gx5 | PCG | C | 514 |
| 2gy5 | NDG | A1158 |  |
| 2gyu | P4G | A | 603 |
| 2gz2 | A2P | A | 502 |
| 2gz7 | D3F | A | 307 |
| 2gz8 | F3F | A | 307 |
| 2h00 | SAH | A | 300 |
| 2h02 | 2UN | A | 402 |
| 2h03 | 3UN | A | 401 |
| 2h04 | 4UN | A | 401 |
| 2h0f | AZA | A | 900 |
| 2h0j | URN | A | 122 |
| 2h0t | MA4 | A | 400 |
| 2h0y | ESA | A | 600 |
| 2h0y | MA4 | A | 400 |
| 2h10 | MA4 | A | 400 |
| 2h1f | ADP | A | 400 |
| 2h2e | SA8 | A | 800 |
| 2h39 | ADQ | A | 506 |
| 2h4g | 694 | A | 300 |
| 2h4k | 509 | A | 300 |
| 2h4l | R1P | X | 600 |
| 2h4t | D12 | A1701 |  |
| 2h57 | GTP | A | 201 |
| 2h58 | ADP | A | 901 |
| 2h5z | CTO | B | 200 |
| 2h6f | FAR | P2010 |  |
| 2h6f | SUC | B3010 |  |
| 2h6g | GER | P2010 |  |
| 2h6g | SUC | B3010 |  |
| 2h6i | GER | P2010 |  |
| 2h6o | MAN | A1171 |  |
| 2h7c | SIA | D | 482 |
| 2h7i | 566 | A | 401 |
| 2h7j | H7J | A | 300 |
| 2h7l | 665 | A | 401 |
| 2h7m | 641 | A | 501 |
| 2h7n | 744 | A | 501 |
| 2h7p | 468 | A | 501 |
| 2h88 | BHG | C | 141 |
| 2h88 | BHG | P | 205 |
| 2h88 | HEM | C | 142 |
| 2h8g | ADE | A | 701 |
| 2h8g | ADE | B | 702 |
| 2h8p | B3H | D | 201 |
| 2h96 | 893 | A | 901 |
| 2h9t | SVR | H | 301 |
| 2h9v | Y27 | A | 500 |
| 2ha3 | CHT | A | 951 |
| 2ha4 | ACH | A | 545 |
| 2ha8 | SAH | A | 401 |
| 2hai | PFI | A | 601 |
| 2hau | CIT | A9202 |  |
| 2hau | CIT | B9206 |  |
| 2hav | CIT | A9207 |  |
| 2hb9 | L13 | A | 500 |
| 2hbt | UN9 | A | 1 |
| 2hbu | UN9 | A | 1 |
| 2hd6 | BOS | A | 266 |
| 2hd9 | CIT | A1001 |  |
| 2hds | 4MB | A1601 |  |
| 2hei | D1D | A | 202 |
| 2hei | D1D | B | 302 |
| 2hen | ADP | A | 400 |
| 2hf9 | GSP | A | 300 |
| 2hfk | E4H | A | 801 |
| 2hfn | FMN | A | 201 |
| 2hfu | MEV | A | 400 |
| 2hg0 | NDG | A | 409 |
| 2hgs | ADP | A | 500 |
| 2hgs | GSH | A | 503 |
| 2hh9 | VIB | A | 702 |
| 2hha | 3TP | A1000 |  |
| 2hhj | 3PG | A3200 |  |
| 2hi4 | BHF | A | 800 |
| 2hi9 | CIT | A1001 |  |
| 2hix | ATP | A | 602 |
| 2hj0 | CIT | A | 601 |
| 2hjp | PPR | A | 291 |
| 2hjr | APR | A3001 |  |
| 2hjr | CIT | A2001 |  |
| 2hka | C3S | B | 400 |
| 2hkm | PEA | D1402 |  |
| 2hko | FAD | A2762 |  |
| 2hkr | ZHZ | H1501 |  |
| 2hkx | HEM | A | 300 |
| 2hl0 | A3S | A | 500 |
| 2hl1 | A3S | A | 500 |
| 2hl2 | SSA | A | 500 |
| 2hl6 | CXS | A | 280 |
| 2hld | ANP | A | 600 |
| 2hld | ANP | D | 600 |
| 2hma | SAM | A | 375 |
| 2hmk | PEY | A | 701 |
| 2hmm | AN3 | A | 801 |
| 2hmp | SPD | A5400 |  |
| 2hmu | ATP | A | 601 |
| 2hmv | ADP | A | 601 |
| 2hmy | SAM | B | 328 |
| 2hn9 | TDR | A2001 |  |
| 2hnd | NVP | A | 999 |
| 2hnx | PLM | A | 135 |
| 2hny | NVP | A | 999 |
| 2hnz | PC0 | A | 999 |
| 2hoc | 1CN | A | 265 |
| 2hod | NDG | E | 470 |
| 2hog | 710 | A | 324 |
| 2hoz | PMP | A5000 |  |
| 2hps | CTZ | A | 187 |
| 2hpv | FMN | A1200 |  |
| 2hq2 | HEM | A | 402 |
| 2hqm | FAD | A1001 |  |
| 2hqm | GSH | A3001 |  |
| 2hrd | TDR | A | 901 |
| 2hrl | BGC | A | 205 |
| 2hrq | SUC | A | 11 |
| 2hru | ADP | A2005 |  |
| 2htw | DAN | A1850 |  |
| 2hu6 | 37A | A | 400 |
| 2hub | PPI | A5001 |  |
| 2huf | 1BO | A | 602 |
| 2hui | GLV | A | 601 |
| 2huo | INS | A | 303 |
| 2hv7 | ADP | B | 324 |
| 2hvc | LGD | A2226 |  |
| 2hvw | DCP | A1201 |  |
| 2hvw | DDN | A1301 |  |
| 2hvx | DRX | A | 500 |
| 2hw1 | FRU | A | 601 |
| 2hwb | W91 | 1 | 700 |
| 2hwc | W54 | 1 | 500 |
| 2hwd | W91 | 1 | 700 |
| 2hwe | W54 | 1 | 500 |
| 2hwf | JEN | 1 | 700 |
| 2hwh | RNA | A | 960 |
| 2hwi | VRX | A | 600 |
| 2hwp | DJK | A1345 |  |
| 2hwu | URI | A | 201 |
| 2hxc | ABN | D1369 |  |
| 2hxl | 422 | A | 324 |
| 2hxm | 302 | A | 401 |
| 2hxq | 373 | A | 324 |
| 2hxs | G3D | A | 401 |
| 2hxv | NDP | A | 400 |
| 2hyu | SGN | A | 804 |
| 2hyv | IDS | A | 803 |
| 2hyv | SGN | A | 802 |
| 2hyy | STI | A | 600 |
| 2hz0 | GIN | A | 600 |
| 2hzl | PYR | A | 601 |
| 2hzn | KIN | A | 600 |
| 2i0g | I0G | A | 1 |
| 2i0j | I0G | A | 1 |
| 2i0k | FAD | A | 700 |
| 2i0s | HY1 | D | 500 |
| 2i0t | PEL | D | 500 |
| 2i0v | 6C3 | A1000 |  |
| 2i0y | 5CN | A1000 |  |
| 2i0z | FAD | A | 501 |
| 2i10 | NPO | B | 201 |
| 2i14 | PCP | A | 392 |
| 2i1m | 5CN | A1000 |  |
| 2i1q | ANP | A | 401 |
| 2i1r | VXR | A | 600 |
| 2i29 | CIT | A | 274 |
| 2i2z | MYR | A1001 |  |
| 2i2z | SAL | A1100 |  |
| 2i30 | MYR | A1300 |  |
| 2i30 | SAL | A1100 |  |
| 2i37 | NDG | B | 705 |
| 2i40 | BLZ | A | 301 |
| 2i4n | 5CA | A | 439 |
| 2i4o | ATP | A | 442 |
| 2i55 | G16 | B | 249 |
| 2i5j | K05 | A4001 |  |
| 2i5j | SUC | A3000 |  |
| 2i5x | UA5 | A | 701 |
| 2i67 | APR | A | 301 |
| 2i6a | 5I5 | A | 500 |
| 2i6b | 89I | A | 500 |
| 2i6p | 4NP | A | 401 |
| 2i74 | MAN | A | 2 |
| 2i76 | NDP | A | 301 |
| 2i76 | NDP | B | 301 |
| 2i78 | KIQ | B | 901 |
| 2i7c | AAT | A | 801 |
| 2i7d | DUR | A | 300 |
| 2i7f | CIT | A | 503 |
| 2i7s | REP | A | 801 |
| 2i80 | G1L | A | 400 |
| 2i80 | G1L | B | 400 |
| 2i99 | NDP | A | 502 |
| 2i9u | GUN | A | 503 |
| 2ia8 | HEM | A | 296 |
| 2iaj | ATP | A | 601 |
| 2iba | AZA | A | 651 |
| 2ibm | ADP | A | 781 |
| 2ic3 | HBY | A | 562 |
| 2ick | DMA | A | 301 |
| 2ics | ADE | A1114 |  |
| 2icv | HEM | A | 296 |
| 2icx | UTP | A | 901 |
| 2icy | UPG | A | 901 |
| 2id4 | MLA | A | 908 |
| 2id4 | NDG | A | 904 |
| 2idk | C2F | A1410 |  |
| 2idv | M7G | A1000 |  |
| 2ieg | FRY | A | 901 |
| 2iei | FRX | A | 904 |
| 2igc | MTN | A | 200 |
| 2igq | SAH | A | 101 |
| 2igt | SAM | A1001 |  |
| 2ihq | LG7 | A | 90 |
| 2ihu | TAR | A | 603 |
| 2ihu | TAR | D | 603 |
| 2iit | 872 | A9001 |  |
| 2iiv | 565 | A9001 |  |
| 2ij7 | TPF | A2472 |  |
| 2ijn | 221 | A9366 |  |
| 2ikg | BTO | A | 317 |
| 2ilt | NN1 | A | 902 |
| 2ilu | NDP | A | 482 |
| 2ily | ATP | A1001 |  |
| 2im0 | CTP | A1001 |  |
| 2imd | 2C2 | A | 305 |
| 2imd | TOH | A | 304 |
| 2imf | CXS | A | 312 |
| 2imf | GSH | A | 301 |
| 2imf | TOM | A | 305 |
| 2img | MLT | A | 501 |
| 2imi | GSH | A | 224 |
| 2imk | GTX | A | 302 |
| 2iml | FMN | A | 200 |
| 2imq | HEM | X | 300 |
| 2in6 | 839 | A | 901 |
| 2inv | FRU | A | 1 |
| 2io6 | 330 | A | 901 |
| 2io7 | ANP | A | 964 |
| 2io8 | ADP | A5001 |  |
| 2iod | MYC | A1341 |  |
| 2iok | IOK | A | 600 |
| 2iop | ADP | A1001 |  |
| 2ior | ADP | A1000 |  |
| 2ipj | FFA | A | 325 |
| 2ipo | MAE | C | 411 |
| 2ipx | MTA | A4001 |  |
| 2iqd | LPA | A | 351 |
| 2irw | NN4 | A | 911 |
| 2irx | GTP | A | 305 |
| 2iry | DGT | A1102 |  |
| 2is5 | CIT | C | 502 |
| 2isa | HEM | A | 486 |
| 2itn | ANP | A2021 |  |
| 2itv | ANP | A2020 |  |
| 2iu8 | PLM | A1349 |  |
| 2iu8 | UD1 | B1348 |  |
| 2iut | SAP | A1723 |  |
| 2iuu | ADP | A1723 |  |
| 2iv7 | UDP | A1372 |  |
| 2ivd | ACJ | A1465 |  |
| 2ivn | ANP | A1327 |  |
| 2ivp | ATP | A1327 |  |
| 2ivu | ZD6 | A3015 |  |
| 2ivv | PP1 | A3014 |  |
| 2iw1 | U2F | A | 900 |
| 2iw3 | ADP | A1973 |  |
| 2iwe | 2IH | A1001 |  |
| 2iwh | ANP | A2973 |  |
| 2iws | NP4 | A1215 |  |
| 2iwu | NP5 | A1215 |  |
| 2iwv | TAM | B1289 |  |
| 2iww | BGC | A1281 |  |
| 2iwx | M1S | A1215 |  |
| 2ix5 | FAD | A1433 |  |
| 2ix6 | FAD | A | 800 |
| 2ixb | A2G | A1446 |  |
| 2ixe | ATP | A | 1 |
| 2ixf | ATP | A | 1 |
| 2ixg | ATP | A | 1 |
| 2ixl | TRH | A1198 |  |
| 2iyf | ERY | A1400 |  |
| 2iyp | 5RP | A1471 |  |
| 2iyp | A2P | B1470 |  |
| 2iyv | ADP | A | 201 |
| 2iyw | ATP | A | 201 |
| 2iyy | S3P | A | 201 |
| 2iz0 | ATR | B1470 |  |
| 2iz1 | ATR | B1470 |  |
| 2iz1 | RES | A1471 |  |
| 2izk | GLL | A | 300 |
| 2izr | PGO | A1342 |  |
| 2izu | P01 | A1335 |  |
| 2izx | DTD | A1044 |  |
| 2j09 | FMN | A1422 |  |
| 2j0b | UDP | A1322 |  |
| 2j0d | ERY | A1498 |  |
| 2j0f | TDR | A1480 |  |
| 2j0g | MAN | F | 402 |
| 2j0h | ACH | A1290 |  |
| 2j0l | ANP | A1689 |  |
| 2j0y | BGC | F1289 |  |
| 2j1e | NDG | A1768 |  |
| 2j1p | PGO | B1303 |  |
| 2j1s | FUL | A1153 |  |
| 2j1u | A2G | A1152 |  |
| 2j2m | HEM | A | 501 |
| 2j2u | GSQ | A1244 |  |
| 2j34 | GS6 | A1245 |  |
| 2j38 | GS5 | A1245 |  |
| 2j3f | A2G | B1289 |  |
| 2j3j | HC4 | A1347 |  |
| 2j3l | P5A | A1576 |  |
| 2j3m | ATP | A | 701 |
| 2j3q | TFL | A1538 |  |
| 2j4g | NB1 | A1590 |  |
| 2j4j | ACP | A | 228 |
| 2j4q | TYD | A | 194 |
| 2j4r | G4P | A1307 |  |
| 2j4z | 626 | A1389 |  |
| 2j5f | DJK | A3021 |  |
| 2j60 | ABE | A1277 |  |
| 2j65 | UDP | A | 402 |
| 2j79 | GTL | A1446 |  |
| 2j7c | IDE | A1446 |  |
| 2j7m | NDG | A1769 |  |
| 2j7w | GTP | A1886 |  |
| 2j7y | E3O | A1454 |  |
| 2j8g | DGL | A1349 |  |
| 2j8s | LMU | A3049 |  |
| 2j90 | IZA | A | 300 |
| 2j90 | IZA | B | 300 |
| 2j94 | G15 | A1245 |  |
| 2j9g | ANP | A1449 |  |
| 2j9h | GTX | A | 300 |
| 2j9l | ATP | B1754 |  |
| 2ja3 | ADP | A1752 |  |
| 2jae | FAD | A1490 |  |
| 2jaf | PLM | A | 700 |
| 2jah | NDP | A1248 |  |
| 2jam | J60 | A1305 |  |
| 2jap | NDP | A1248 |  |
| 2jaq | DCP | A1202 |  |
| 2jar | UMP | A1200 |  |
| 2jas | DTP | A1201 |  |
| 2jat | DCM | A1205 |  |
| 2jau | ATM | A1230 |  |
| 2jav | 5Z5 | A1280 |  |
| 2jaw | BVP | A1228 |  |
| 2jb1 | FAD | A1489 |  |
| 2jb2 | FAD | A1489 |  |
| 2jb3 | FAD | A1489 |  |
| 2jbs | FMN | A1423 |  |
| 2jbt | FMN | A1423 |  |
| 2jc0 | 699 | A1563 |  |
| 2jc1 | 698 | A | 600 |
| 2jc5 | BCN | A1261 |  |
| 2jc6 | QPP | A1314 |  |
| 2jc6 | QPP | C1314 |  |
| 2jcb | ADP | A1190 |  |
| 2jcr | BDP | A1176 |  |
| 2jcs | TTP | A1210 |  |
| 2jdd | 3PG | A1148 |  |
| 2jdo | I5S | A1480 |  |
| 2jds | L20 | A1351 |  |
| 2je7 | XMM | A1242 |  |
| 2je8 | B3P | A1865 |  |
| 2je8 | B3P | B1869 |  |
| 2jen | XYS | A1271 |  |
| 2jf3 | UD1 | A1263 |  |
| 2jfa | RAL | A | 600 |
| 2jfa | RAL | B | 600 |
| 2jff | LK2 | A1440 |  |
| 2jfg | ADP | A1441 |  |
| 2jfh | LK1 | A1441 |  |
| 2jh0 | 701 | D1247 |  |
| 2jh5 | 895 | D1249 |  |
| 2jh6 | 894 | D1249 |  |
| 2jh7 | SIA | A1240 |  |
| 2jh8 | GUN | A1646 |  |
| 2jh8 | M7G | A1645 |  |
| 2jh9 | GTP | A1645 |  |
| 2jh9 | GUN | A1646 |  |
| 2jha | GP3 | A1645 |  |
| 2jha | GUN | A1646 |  |
| 2jhc | GUN | A1645 |  |
| 2jhd | SIA | A1240 |  |
| 2jhi | A2G | F1299 |  |
| 2jhl | SLB | F1299 |  |
| 2jhn | MBO | A1296 |  |
| 2jhp | GUN | A1647 |  |
| 2jhp | SAH | A1645 |  |
| 2ji7 | B3P | A1570 |  |
| 2ji7 | B3P | A1571 |  |
| 2ji7 | B3P | A1572 |  |
| 2jib | ADP | A1568 |  |
| 2jid | GVB | A1771 |  |
| 2jie | G2F | A1449 |  |
| 2jif | FAD | A1433 |  |
| 2jih | 097 | A1001 |  |
| 2jiy | D12 | M1314 |  |
| 2jiz | ADP | D1476 |  |
| 2jiz | ANP | A1511 |  |
| 2jiz | ANP | F1475 |  |
| 2jiz | STL | G1273 |  |
| 2jj1 | ADP | D1476 |  |
| 2jj1 | ANP | A1511 |  |
| 2jj1 | ANP | F1475 |  |
| 2jj2 | ADP | D1476 |  |
| 2jj2 | ANP | A1511 |  |
| 2jj2 | ANP | F1475 |  |
| 2jj2 | QUE | G1273 |  |
| 2jj3 | JJ3 | A1499 |  |
| 2jjg | L18 | A | 601 |
| 2jjn | HEM | A | 412 |
| 2jjq | SAH | A1406 |  |
| 2jjr | TAM | A1257 |  |
| 2jk8 | ATP | A | 701 |
| 2jkc | FAD | A1518 |  |
| 2jkj | TH8 | B1142 |  |
| 2jkk | BI9 | A1686 |  |
| 2jkp | CTS | A1727 |  |
| 2jky | 5GP | A | 300 |
| 2jl4 | GSH | A1213 |  |
| 2jle | I15 | A1546 |  |
| 2jlr | ANP | A1620 |  |
| 2ki5 | AC2 | A | 1 |
| 2kin | ADP | A | 400 |
| 2lig | PHN | A | 390 |
| 2mjp | ANP | A | 500 |
| 2mpr | BGC | A | 428 |
| 2myd | NPN | A | 155 |
| 2mye | ENC | A | 155 |
| 2nlr | BGC | A | 601 |
| 2nlr | G2F | A | 604 |
| 2nmo | BGC | A | 501 |
| 2nng | ZYX | A | 301 |
| 2nno | M28 | A | 301 |
| 2nns | M25 | A | 301 |
| 2nnv | M29 | A | 301 |
| 2no0 | ADP | A | 301 |
| 2no0 | GEO | A | 302 |
| 2no1 | ADP | A | 301 |
| 2no1 | DCZ | A | 302 |
| 2no3 | 859 | A | 901 |
| 2no6 | ADP | A | 301 |
| 2no7 | ADP | A | 301 |
| 2no9 | ADP | A | 301 |
| 2noa | ADP | A | 301 |
| 2nom | DUT | A | 501 |
| 2np5 | NDS | A | 601 |
| 2np8 | CC3 | A | 394 |
| 2npi | ATP | A | 600 |
| 2npn | SAM | A4633 |  |
| 2nq6 | HM4 | A | 410 |
| 2nq7 | HM5 | A | 410 |
| 2nqd | NDG | B | 221 |
| 2nr9 | PA6 | A | 201 |
| 2nrl | HEM | A | 148 |
| 2nru | T12 | A | 600 |
| 2nsd | 4PI | A | 400 |
| 2nta | 521 | A | 301 |
| 2ntk | IMP | A | 205 |
| 2nun | ADP | A | 600 |
| 2nuo | BGC | A | 301 |
| 2nuv | 2TN | A2001 |  |
| 2nv7 | 555 | A | 501 |
| 2nv9 | PLP | A2001 |  |
| 2nvc | ITA | A | 600 |
| 2nvd | ITB | A | 600 |
| 2nvu | ATP | B | 103 |
| 2nw4 | 8NH | A | 1 |
| 2nw6 | POT | A | 612 |
| 2nw8 | HEM | A | 401 |
| 2nwl | PLM | A | 801 |
| 2nwr | PEP | A1268 |  |
| 2nws | A5P | A1269 |  |
| 2nws | PEP | A1268 |  |
| 2nx1 | PEP | A1268 |  |
| 2nx1 | RP5 | A1269 |  |
| 2nxe | SAM | A | 302 |
| 2nxg | A5P | A1269 |  |
| 2nxg | PEP | A1268 |  |
| 2nxh | PEP | A1268 |  |
| 2ny1 | SUC | D4000 |  |
| 2nyr | SVR | B | 401 |
| 2nz5 | 226 | A | 431 |
| 2nza | HEM | B | 430 |
| 2nzl | GLV | A | 601 |
| 2nzt | BG6 | A1002 |  |
| 2nzt | BG6 | A1004 |  |
| 2nzu | BG6 | G1092 |  |
| 2nzv | FBP | G2128 |  |
| 2o07 | MTA | A | 501 |
| 2o07 | SPD | A | 601 |
| 2o08 | DGI | B | 500 |
| 2o0e | PEP | A | 502 |
| 2o0h | ATP | A | 400 |
| 2o0j | ADP | A | 400 |
| 2o12 | FMN | A | 593 |
| 2o1u | ANP | A | 755 |
| 2o1u | ANP | B | 755 |
| 2o1v | ADP | B | 302 |
| 2o1x | TDP | A1001 |  |
| 2o28 | 16G | A | 501 |
| 2o2c | G6Q | A5001 |  |
| 2o2d | CIT | A5001 |  |
| 2o2h | DCE | A | 303 |
| 2o2r | NDP | A | 903 |
| 2o2u | 738 | A | 403 |
| 2o3k | HPY | B | 410 |
| 2o3p | QUE | A | 501 |
| 2o4l | TPV | A | 403 |
| 2o4n | TPV | A | 300 |
| 2o4p | TPV | A | 300 |
| 2o4s | AB1 | A | 400 |
| 2o5n | NDG | B | 404 |
| 2o5z | ANO | H | 201 |
| 2o63 | MYC | A | 501 |
| 2o65 | MYF | A | 501 |
| 2o74 | GUN | A1001 |  |
| 2o7b | HC4 | A | 701 |
| 2o7d | DHC | A | 701 |
| 2o7i | CBI | A1893 |  |
| 2o7n | 2O7 | A | 1 |
| 2o7r | 4PA | A | 369 |
| 2o7s | DHK | A4733 |  |
| 2o8j | SAH | A1505 |  |
| 2o99 | GOA | A | 902 |
| 2o9a | PYR | B | 906 |
| 2o9d | HSG | A | 232 |
| 2o9d | HSH | A | 233 |
| 2o9i | 444 | B | 1 |
| 2o9j | CZA | A1001 |  |
| 2o9r | TCB | A | 500 |
| 2o9t | BGC | A | 500 |
| 2oa0 | ADP | A1002 |  |
| 2oa0 | CZA | A1001 |  |
| 2oa1 | FAD | A2004 |  |
| 2oag | DLI | B4000 |  |
| 2oal | FAD | B | 601 |
| 2oax | SNL | A1001 |  |
| 2obc | RP5 | A | 401 |
| 2obd | PCW | A | 487 |
| 2obf | F83 | B2002 |  |
| 2obf | SAH | B3002 |  |
| 2obj | VRV | A | 400 |
| 2obm | ADP | A | 600 |
| 2obs | A2G | A | 3 |
| 2obx | INI | A | 201 |
| 2ocp | DTP | A | 301 |
| 2odv | PGO | A1001 |  |
| 2oec | ANU | B | 2 |
| 2oem | 1AE | A | 901 |
| 2of0 | CMZ | A | 390 |
| 2of2 | 547 | A | 201 |
| 2of4 | 979 | A | 101 |
| 2of8 | BNI | B | 501 |
| 2ofb | BNI | B | 501 |
| 2off | OFF | A | 930 |
| 2ofp | PAF | A | 602 |
| 2ofv | 242 | A | 503 |
| 2ofx | PPS | A1100 |  |
| 2ogx | ATP | A | 281 |
| 2oh0 | 2PY | E | 351 |
| 2oh5 | ATP | A | 253 |
| 2oh5 | CTP | A | 254 |
| 2oh5 | GTP | A | 252 |
| 2oh6 | ATP | A | 253 |
| 2oh6 | CTP | A | 254 |
| 2oh7 | ATP | A | 253 |
| 2oh7 | CTP | A | 254 |
| 2oh8 | HEM | A | 154 |
| 2oh9 | HEM | A | 154 |
| 2oha | HEM | A | 154 |
| 2ohb | HEM | A | 154 |
| 2ohf | ACP | A9001 |  |
| 2ohh | FMN | A | 701 |
| 2ohi | FMN | A | 701 |
| 2ohj | FMN | A | 701 |
| 2ohm | 8AP | A | 389 |
| 2ohr | 8IP | A | 504 |
| 2ohs | 9IP | A | 389 |
| 2oht | IP6 | A | 601 |
| 2ohu | IP7 | A | 390 |
| 2ohv | NHL | A | 400 |
| 2oi0 | 283 | A | 1 |
| 2oi6 | GP1 | A5000 |  |
| 2oi7 | DCA | A2000 |  |
| 2oid | ANP | A | 1 |
| 2oif | PGO | A1210 |  |
| 2oiq | STI | A1001 |  |
| 2oiz | TSR | D1702 |  |
| 2oj9 | BMI | A | 301 |
| 2ojf | 4PY | E1000 |  |
| 2ojg | 19A | A | 360 |
| 2oji | 33A | A | 360 |
| 2ojj | 82A | A | 360 |
| 2ojw | ADP | A | 501 |
| 2ok1 | 33A | A | 1 |
| 2okg | G3H | B | 501 |
| 2okj | ABU | A1001 |  |
| 2okl | CIT | A | 501 |
| 2olh | CBI | A | 840 |
| 2oli | IOP | A1001 |  |
| 2olj | ADP | A | 401 |
| 2olr | ATP | A | 541 |
| 2omg | CRS | A | 502 |
| 2onc | SY1 | A | 800 |
| 2onc | SY1 | A | 801 |
| 2onj | ANP | A | 701 |
| 2onm | ADP | A | 501 |
| 2ony | TMJ | A2001 |  |
| 2onz | SAH | B3002 |  |
| 2onz | TMJ | B2002 |  |
| 2oo0 | N2P | A | 700 |
| 2oo0 | N2P | B | 700 |
| 2oo0 | PLP | A | 600 |
| 2oo7 | AP5 | A1218 |  |
| 2ooh | OX3 | B | 200 |
| 2ooq | B3P | A | 301 |
| 2oow | OX4 | A | 200 |
| 2ooy | ATP | E | 401 |
| 2ooz | OX5 | A | 200 |
| 2op9 | WR1 | A | 601 |
| 2opa | FHC | A | 500 |
| 2oph | 277 | A1001 |  |
| 2opq | HBQ | A | 999 |
| 2opr | HBQ | A | 999 |
| 2ops | HBQ | A | 999 |
| 2opx | DXC | A1001 |  |
| 2oq2 | A3P | A | 999 |
| 2oqi | GGO | B | 901 |
| 2oqv | MA9 | A | 901 |
| 2or4 | QUS | A1801 |  |
| 2ori | AP5 | A | 418 |
| 2oro | 228 | A | 601 |
| 2orq | MSR | A | 601 |
| 2orr | 333 | A | 601 |
| 2orr | HEM | A | 600 |
| 2ors | 391 | A | 601 |
| 2ors | HEM | A | 600 |
| 2ort | 342 | A | 601 |
| 2os1 | BB2 | A | 400 |
| 2os9 | INS | A | 631 |
| 2osb | AP5 | A1218 |  |
| 2osc | MUH | A | 50 |
| 2osx | SIA | A | 600 |
| 2ot1 | N3P | A3001 |  |
| 2ot4 | CIT | B2010 |  |
| 2otf | 2TN | A | 201 |
| 2otv | NCA | A1301 |  |
| 2ou7 | ANP | A | 500 |
| 2ouq | 5GP | A | 401 |
| 2our | CMP | A | 778 |
| 2ov4 | ANL | A | 950 |
| 2ov5 | BCN | A | 501 |
| 2ovu | MAN | A | 241 |
| 2ovu | MMA | A | 242 |
| 2ovv | PFH | A | 999 |
| 2ovx | 4MR | A | 501 |
| 2ovy | PFJ | A | 999 |
| 2ow1 | 7MR | A | 501 |
| 2ow2 | 8MR | A | 501 |
| 2ow4 | MMA | A | 242 |
| 2ow9 | SP6 | A | 501 |
| 2owb | 626 | A | 500 |
| 2owz | CIT | A | 604 |
| 2owz | F6P | A | 603 |
| 2ox3 | F6P | A | 603 |
| 2ox3 | PEP | A | 604 |
| 2oxc | ADP | A | 300 |
| 2oye | IM8 | P | 700 |
| 2oyf | IAC | A1001 |  |
| 2oyl | IDC | B | 600 |
| 2oyr | SAH | A | 301 |
| 2oys | FMN | A | 301 |
| 2oyu | IMS | P | 700 |
| 2oyy | HEM | A | 201 |
| 2oz0 | FMN | B | 570 |
| 2oz0 | PYR | B | 571 |
| 2ozo | ANP | A | 615 |
| 2ozr | GG1 | A2001 |  |
| 2ozy | HEC | A | 201 |
| 2p02 | SAM | A | 2 |
| 2p05 | ADP | A | 100 |
| 2p09 | ATP | A | 500 |
| 2p0a | ANP | A | 707 |
| 2p0b | HEC | A | 201 |
| 2p0c | ANP | A | 1 |
| 2p0d | I3P | A | 800 |
| 2p0h | I3S | A | 101 |
| 2p18 | SPD | A | 304 |
| 2p1d | 5GP | A | 912 |
| 2p1o | NLA | B | 801 |
| 2p1p | IAC | B | 601 |
| 2p1q | IAC | B | 901 |
| 2p2i | 608 | A | 501 |
| 2p33 | J07 | A | 507 |
| 2p35 | SAH | A | 301 |
| 2p39 | SCR | A | 1 |
| 2p3s | AP5 | A | 318 |
| 2p41 | CIT | A1013 |  |
| 2p54 | 735 | A | 469 |
| 2p55 | MRA | A9000 |  |
| 2p6f | GN8 | A | 701 |
| 2p6g | 3LP | C | 703 |
| 2p72 | UPG | B | 215 |
| 2p73 | UDP | A | 411 |
| 2p7k | CIT | A | 134 |
| 2p85 | IND | A | 501 |
| 2p8c | SUG | A | 501 |
| 2p8i | CIT | C | 118 |
| 2p8s | 417 | A1001 |  |
| 2p93 | ME1 | A | 1 |
| 2p95 | ME5 | A | 1 |
| 2p98 | YE7 | A | 266 |
| 2p9a | YE6 | A | 267 |
| 2p9e | CIT | A | 514 |
| 2p9e | CIT | A | 518 |
| 2p9e | CIT | B | 516 |
| 2p9h | IPT | A | 998 |
| 2p9i | ADP | B | 402 |
| 2p9k | ATP | B | 602 |
| 2p9n | ADP | B | 395 |
| 2p9p | ADP | B | 395 |
| 2p9s | ATP | B | 502 |
| 2p9u | ANP | B | 902 |
| 2pa4 | GUD | A | 326 |
| 2pa7 | TYD | A | 141 |
| 2paf | NPF | A | 902 |
| 2pan | TDP | A | 801 |
| 2pan | TDP | C | 801 |
| 2par | TMP | A | 301 |
| 2pav | ATP | A | 602 |
| 2pax | 4AN | A | 1 |
| 2pb1 | NFG | A | 402 |
| 2pbd | ATP | A1001 |  |
| 2pbj | HEM | A | 476 |
| 2pbw | DOQ | A | 401 |
| 2pcp | 1PC | B | 227 |
| 2pd4 | DCN | A2414 |  |
| 2pd5 | ZST | A | 600 |
| 2pd9 | FID | A | 600 |
| 2pdb | ZST | A | 600 |
| 2pdc | 393 | A | 600 |
| 2pdf | ZST | A | 600 |
| 2pdg | 47D | A | 600 |
| 2pdh | 47D | A | 600 |
| 2pdi | ZST | A | 600 |
| 2pdj | 393 | A | 600 |
| 2pdl | TOL | A | 600 |
| 2pdm | ZST | A | 600 |
| 2pdn | 47D | A | 600 |
| 2pdp | 393 | A | 600 |
| 2pdq | 47D | A | 600 |
| 2pdu | 393 | A | 600 |
| 2pdw | FID | A | 600 |
| 2pdx | ZST | A | 600 |
| 2pdy | FID | A | 600 |
| 2pe0 | 39Z | A | 501 |
| 2pe1 | 517 | A | 501 |
| 2pf8 | FID | A | 321 |
| 2pfc | PLM | A | 184 |
| 2pg0 | FAD | A | 401 |
| 2pg2 | K01 | A | 604 |
| 2pg4 | CIT | B | 97 |
| 2pga | ANU | B7016 |  |
| 2pgn | FAD | A | 612 |
| 2pgo | FAD | B | 613 |
| 2pgt | GPR | A | 211 |
| 2ph8 | 35A | A | 388 |
| 2ph9 | GNT | A | 301 |
| 2phb | 230 | A9001 |  |
| 2phh | APR | A | 395 |
| 2pia | FMN | A | 322 |
| 2pid | YSA | A | 384 |
| 2pin | 4HY | A | 500 |
| 2pin | LEG | A | 501 |
| 2pip | K10 | L | 936 |
| 2pit | 4HY | A | 933 |
| 2pix | FLF | A2386 |  |
| 2piy | 528 | A | 401 |
| 2piz | 606 | A | 401 |
| 2pj1 | 578 | A | 401 |
| 2pj2 | 864 | A | 401 |
| 2pj6 | 059 | A | 401 |
| 2pj7 | 235 | A | 401 |
| 2pjl | 047 | A | 600 |
| 2pjt | 347 | A | 401 |
| 2pkk | 2FA | A | 501 |
| 2pks | G44 | C | 101 |
| 2plv | MYR | 4 | 1 |
| 2plv | SPH | 1 | 0 |
| 2pmi | SAP | A3001 |  |
| 2pmk | 128 | A | 301 |
| 2pml | ANP | X | 381 |
| 2pmo | HMD | X | 400 |
| 2pnn | ATP | A | 374 |
| 2pnu | ENM | A | 302 |
| 2pnz | 5GP | A | 279 |
| 2pnz | UDP | A | 250 |
| 2po0 | ADP | A | 300 |
| 2poc | BG6 | A | 713 |
| 2poc | UD1 | A5002 |  |
| 2pog | WST | A | 201 |
| 2pog | WST | B | 301 |
| 2pok | BGC | A | 458 |
| 2pou | I7A | A1000 |  |
| 2pqf | CIT | A | 801 |
| 2pqj | ADE | A | 501 |
| 2pql | TSS | A | 145 |
| 2pr1 | SUC | A | 201 |
| 2pr5 | FMN | A | 500 |
| 2pri | D6G | A | 930 |
| 2prz | OMP | A | 450 |
| 2ps1 | ORO | A | 500 |
| 2psv | MUV | A | 200 |
| 2ptm | CMP | A | 401 |
| 2ptq | FMR | A1200 |  |
| 2ptr | 2SA | B1100 |  |
| 2ptz | PAH | A | 600 |
| 2pu2 | DK2 | A | 702 |
| 2pu4 | OX6 | A | 901 |
| 2pu4 | OX7 | B | 902 |
| 2puj | HPZ | A | 289 |
| 2pul | ACP | A | 999 |
| 2put | F6R | A5003 |  |
| 2put | UD1 | A5002 |  |
| 2puv | UD1 | A5002 |  |
| 2puw | BG6 | A | 713 |
| 2pva | DTD | A1001 |  |
| 2pvh | P19 | A | 501 |
| 2pvj | P44 | A | 501 |
| 2pvk | P45 | A | 501 |
| 2pvl | P55 | A | 501 |
| 2pvm | P29 | A | 501 |
| 2pvn | P63 | A | 501 |
| 2pw0 | TRC | A2001 |  |
| 2pw3 | CMP | A | 1 |
| 2pwg | CTS | A8000 |  |
| 2pws | IBP | A3960 |  |
| 2pwu | GUN | A | 600 |
| 2pwy | SAH | A | 501 |
| 2px1 | RIP | A | 694 |
| 2pxx | SAH | A | 301 |
| 2pxz | OAA | X | 701 |
| 2pyh | MAV | D | 601 |
| 2pze | ATP | A | 1 |
| 2pzf | ATP | A | 1 |
| 2pzg | ATP | A | 1 |
| 2pzi | AXX | A | 753 |
| 2pzl | UDP | A | 402 |
| 2pzm | UDP | A | 602 |
| 2pzn | 393 | A | 320 |
| 2pzy | B18 | A | 1 |
| 2q0c | CTP | A | 501 |
| 2q0d | ATP | A | 501 |
| 2q0e | GTP | A | 501 |
| 2q0k | FAD | A | 400 |
| 2q0l | FAD | A | 400 |
| 2q0r | ATP | A | 425 |
| 2q0u | ATP | A | 425 |
| 2q11 | XX4 | A | 501 |
| 2q14 | ADP | A | 501 |
| 2q1a | 2KT | X | 295 |
| 2q1h | AS4 | A | 301 |
| 2q1t | UDP | A | 400 |
| 2q1u | UDP | A | 701 |
| 2q28 | ADP | A4003 |  |
| 2q2c | HIS | A | 504 |
| 2q2h | CIT | A | 601 |
| 2q2r | ADP | A2001 |  |
| 2q2r | BGC | A1001 |  |
| 2q3e | UPG | A | 501 |
| 2q3i | DGN | D | 7 |
| 2q3y | 1CA | A | 247 |
| 2q58 | ZOL | A | 1 |
| 2q5a | 16P | A | 300 |
| 2q5o | PPY | A5001 |  |
| 2q5q | KPV | A5001 |  |
| 2q6h | CXX | A | 801 |
| 2q6m | P34 | A1001 |  |
| 2q6o | SAM | A | 500 |
| 2q6s | PLB | B5001 |  |
| 2q6v | UDP | A1081 |  |
| 2q70 | DC8 | A | 50 |
| 2q70 | DC8 | B | 51 |
| 2q72 | IXX | A | 801 |
| 2q78 | MLC | A | 200 |
| 2q7a | HEM | A | 182 |
| 2q7d | ANP | A | 917 |
| 2q7e | ANP | A | 900 |
| 2q7g | ATP | A | 900 |
| 2q7t | TMP | B2001 |  |
| 2q7u | TMP | A2001 |  |
| 2q7v | FAD | A | 348 |
| 2q7w | PMP | A | 700 |
| 2q83 | CIT | B | 3 |
| 2q85 | 973 | A | 401 |
| 2q8c | AKG | A | 503 |
| 2q8d | SIN | A | 503 |
| 2q8d | SIN | B | 504 |
| 2q8g | AZX | A | 438 |
| 2q8j | M2P | A | 707 |
| 2q8m | BG6 | A | 340 |
| 2q8m | FBP | A | 344 |
| 2q8p | HEM | A | 300 |
| 2q92 | B23 | A | 400 |
| 2q93 | B21 | A | 400 |
| 2q94 | A04 | A | 400 |
| 2q95 | A05 | A | 400 |
| 2q96 | A18 | A | 400 |
| 2q97 | ATP | A1001 |  |
| 2q9e | MTN | A | 844 |
| 2q9f | C3S | A | 600 |
| 2q9f | HEM | A | 602 |
| 2qa3 | PMP | A | 700 |
| 2qa6 | KN2 | A | 1 |
| 2qa8 | GEN | A | 600 |
| 2qa8 | GEN | B | 600 |
| 2qab | EI1 | A | 1 |
| 2qad | MLA | B | 186 |
| 2qad | MLA | F | 186 |
| 2qae | FAD | A | 480 |
| 2qag | GTP | B | 428 |
| 2qaj | AP5 | A | 618 |
| 2qb2 | PMP | A | 700 |
| 2qb3 | PMP | A | 700 |
| 2qb4 | DSM | A | 801 |
| 2qb5 | ADP | A | 557 |
| 2qb5 | ADP | B | 558 |
| 2qb8 | ATP | A | 501 |
| 2qbr | 910 | A | 800 |
| 2qbs | 024 | A | 800 |
| 2qc1 | MAN | B | 304 |
| 2qc1 | MAN | B | 308 |
| 2qc6 | G12 | A | 1 |
| 2qc8 | ADP | A | 501 |
| 2qca | DGP | A | 200 |
| 2qch | 5IU | A | 1 |
| 2qch | UMP | A | 2 |
| 2qcl | OMP | A | 1 |
| 2qcs | TAM | A | 406 |
| 2qcu | FAD | A | 600 |
| 2qcu | TAM | A | 805 |
| 2qd1 | PP9 | A | 701 |
| 2qd3 | CHD | B | 504 |
| 2qd3 | HEM | B | 925 |
| 2qd4 | CHD | B | 926 |
| 2qd5 | CHD | B1103 |  |
| 2qd5 | PP9 | B1105 |  |
| 2qdt | I38 | A | 501 |
| 2qe2 | 452 | A | 579 |
| 2qe4 | JJ3 | A | 601 |
| 2qe5 | 617 | A | 579 |
| 2qe9 | CIT | A | 201 |
| 2qeo | LNR | A | 200 |
| 2qes | ADE | A | 501 |
| 2qeu | CIT | A | 146 |
| 2qey | GTP | A2696 |  |
| 2qf2 | OAA | A | 701 |
| 2qf2 | PYR | A4083 |  |
| 2qf6 | A56 | A | 256 |
| 2qf7 | SAP | A1162 |  |
| 2qfo | A51 | A | 999 |
| 2qfp | NDG | B | 438 |
| 2qfp | NDG | B | 439 |
| 2qfr | NDG | A | 453 |
| 2qg0 | A94 | A2001 |  |
| 2qg2 | A91 | A | 300 |
| 2qg6 | NMN | A | 301 |
| 2qgc | MR4 | A | 128 |
| 2qgd | MR5 | A | 200 |
| 2qge | MR6 | A | 201 |
| 2qgi | UDP | B | 800 |
| 2qgq | CXS | A | 501 |
| 2qgt | EED | A | 701 |
| 2qgu | PEF | A | 301 |
| 2qgw | EES | A | 1 |
| 2qh6 | ODE | A | 700 |
| 2qhc | AB1 | B9001 |  |
| 2qhf | NCA | A | 493 |
| 2qhm | 7CS | A | 500 |
| 2qhn | 582 | A | 500 |
| 2qhs | OCA | A3913 |  |
| 2qhw | PZZ | A | 134 |
| 2qik | CIT | A | 301 |
| 2qio | TCL | A | 601 |
| 2qis | RIS | A | 901 |
| 2qjf | ADX | A | 100 |
| 2qjo | APR | A | 501 |
| 2qjr | PZF | A | 900 |
| 2qjy | BGL | B1041 |  |
| 2qk8 | MTX | A | 200 |
| 2qkm | ATP | B | 267 |
| 2qkr | IXM | A | 401 |
| 2ql5 | CIT | D | 850 |
| 2ql6 | ADP | A | 301 |
| 2ql7 | CIT | D | 850 |
| 2ql9 | CIT | D | 850 |
| 2qlb | CIT | D | 850 |
| 2qlj | CIT | A | 850 |
| 2qls | HEM | A | 142 |
| 2qlu | ADE | A | 488 |
| 2qm9 | TDZ | A | 201 |
| 2qmb | HEM | A | 150 |
| 2qmb | HEM | B | 150 |
| 2qmc | GTB | B | 1 |
| 2qmy | AD1 | A | 501 |
| 2qmz | LDP | A | 501 |
| 2qn7 | IMP | A | 930 |
| 2qnx | MDX | A | 861 |
| 2qnx | UDT | A | 860 |
| 2qo0 | D1T | A | 761 |
| 2qo1 | VZZ | A | 760 |
| 2qo4 | CHD | A | 130 |
| 2qo7 | ANP | A1000 |  |
| 2qo8 | 3CC | A | 800 |
| 2qo9 | ANP | A | 948 |
| 2qoa | MAJ | A | 800 |
| 2qoc | ANP | A | 949 |
| 2qoe | 448 | A1282 |  |
| 2qoh | P3Y | A | 1 |
| 2qoq | ANP | A | 948 |
| 2qor | 5GP | A | 197 |
| 2qp8 | TAR | A | 2 |
| 2qpa | ADP | A | 800 |
| 2qpd | HEM | A | 800 |
| 2qpe | HEM | A | 800 |
| 2qpk | SHA | A | 616 |
| 2qpp | HEM | A | 300 |
| 2qpq | CIT | A | 302 |
| 2qpt | ANP | A | 600 |
| 2qq0 | ADP | B | 701 |
| 2qq0 | ANP | A | 601 |
| 2qq0 | THM | A | 501 |
| 2qq0 | TMP | B | 601 |
| 2qqw | SUC | A | 800 |
| 2qr1 | ADP | E1002 |  |
| 2qr2 | VK3 | B | 235 |
| 2qr9 | HZ3 | A | 1 |
| 2qrc | ADP | E1002 |  |
| 2qrd | ADP | E1004 |  |
| 2qrd | ATP | E1002 |  |
| 2qrh | M08 | A | 998 |
| 2qrn | DCM | A | 403 |
| 2qrp | S06 | A | 998 |
| 2qrq | S13 | A | 998 |
| 2qrv | SAH | A | 1 |
| 2qry | TPS | A | 400 |
| 2qse | 1HP | A | 1 |
| 2qsz | NMN | A | 201 |
| 2qt0 | ANP | A1102 |  |
| 2qt9 | 524 | A1522 |  |
| 2qtb | 474 | A1521 |  |
| 2qtg | MTH | A | 268 |
| 2qtl | FAD | A | 700 |
| 2qts | MAL | B | 1 |
| 2qtt | ADE | A | 268 |
| 2qtu | 3AS | A | 1 |
| 2qtz | FAD | A | 700 |
| 2qu0 | HEM | A | 142 |
| 2qu0 | HEM | B | 147 |
| 2qu2 | 251 | A | 1 |
| 2qu3 | 462 | A | 1 |
| 2qu5 | 276 | A | 501 |
| 2qv6 | GTP | A | 300 |
| 2qv7 | ADP | A | 500 |
| 2qvd | BER | A1811 |  |
| 2qvn | GMP | A | 500 |
| 2qvr | CIT | A | 335 |
| 2qwe | GNA | A | 800 |
| 2qwf | G20 | A | 800 |
| 2qwg | G28 | A | 800 |
| 2qwx | ML1 | A | 233 |
| 2qx8 | ML2 | A | 235 |
| 2qxf | TMP | A3000 |  |
| 2qxs | RAL | A | 600 |
| 2qxx | TTP | A | 201 |
| 2qyk | NPV | A | 3 |
| 2qyn | NPV | A | 1 |
| 2qyo | SAH | A | 601 |
| 2qyu | B3P | A | 1 |
| 2qz4 | ADP | A | 700 |
| 2qzo | KN1 | A | 1 |
| 2qzo | KN1 | B | 1 |
| 2qzr | S79 | A | 500 |
| 2qzs | ADP | A | 487 |
| 2qzy | PEP | A1102 |  |
| 2r01 | FMN | A | 212 |
| 2r04 | W71 | 1 | 900 |
| 2r06 | W35 | 1 | 900 |
| 2r07 | W33 | 1 | 900 |
| 2r0g | 7CK | A1355 |  |
| 2r0h | CTO | A | 165 |
| 2r0p | K2C | A1185 |  |
| 2r1h | HEM | B | 148 |
| 2r1n | EPL | A | 701 |
| 2r1p | DPJ | A | 1 |
| 2r1y | KDO | B | 212 |
| 2r24 | LDT | A | 317 |
| 2r2b | KDO | B | 215 |
| 2r2i | MYR | A | 1 |
| 2r2j | SIN | A | 380 |
| 2r2m | I50 | B5000 |  |
| 2r2v | CIT | C | 37 |
| 2r2w | 4PG | U | 300 |
| 2r3c | DGN | C | 11 |
| 2r3c | DGN | D | 11 |
| 2r3c | DTR | C | 10 |
| 2r3d | ACM | A | 303 |
| 2r3f | SC8 | A | 501 |
| 2r3g | SC9 | A | 501 |
| 2r3h | SCE | A | 501 |
| 2r3i | SCF | A | 501 |
| 2r3j | SCJ | A | 501 |
| 2r3k | SCQ | A | 501 |
| 2r3l | SCW | A | 501 |
| 2r3m | SCX | A | 501 |
| 2r3n | SCZ | A | 501 |
| 2r3o | 2SC | A | 501 |
| 2r3p | 3SC | A | 501 |
| 2r3q | 5SC | A | 501 |
| 2r3r | 6SC | A | 501 |
| 2r42 | FPS | A | 400 |
| 2r45 | 2PG | A | 700 |
| 2r45 | FAD | A | 600 |
| 2r46 | FAD | A | 600 |
| 2r46 | PEP | A | 700 |
| 2r4e | 13P | A1955 |  |
| 2r4e | FAD | A | 600 |
| 2r4i | CIT | A | 123 |
| 2r4j | 13P | A1968 |  |
| 2r4j | BCN | A1969 |  |
| 2r4j | FAD | A | 600 |
| 2r4j | TAM | B | 812 |
| 2r5b | DTR | H | 11 |
| 2r5d | DTR | H | 11 |
| 2r5n | R5P | A | 670 |
| 2r5n | RP5 | B | 671 |
| 2r5v | HHH | A4114 |  |
| 2r64 | 740 | A | 500 |
| 2r66 | F6P | A | 501 |
| 2r6f | ADP | A1000 |  |
| 2r6j | NDP | A | 401 |
| 2r6s | BCN | A | 505 |
| 2r6v | NCA | A | 174 |
| 2r71 | INS | A | 787 |
| 2r7a | HEM | A | 600 |
| 2r7b | 253 | A | 701 |
| 2r7k | ACP | A | 400 |
| 2r7l | ATP | A | 400 |
| 2r7p | ANP | A | 400 |
| 2r80 | HEM | A | 150 |
| 2r80 | HEM | B | 150 |
| 2r87 | ADP | A | 400 |
| 2r8q | IBM | A | 3 |
| 2r9j | NCA | A | 692 |
| 2r9s | 255 | A | 502 |
| 2r9v | ATP | A | 506 |
| 2r9w | 23C | A | 365 |
| 2r9x | WH6 | A | 365 |
| 2r9z | FAD | A | 500 |
| 2rab | FAD | A | 500 |
| 2rao | HEM | A | 142 |
| 2rao | HEM | B | 147 |
| 2rbe | ZMG | A | 601 |
| 2rbs | 269 | A1001 |  |
| 2rc5 | FAD | A | 415 |
| 2rc6 | FAD | A | 415 |
| 2rc9 | 1AC | A1001 |  |
| 2rcw | AAI | A | 400 |
| 2rd6 | 78P | A | 900 |
| 2rdb | MPO | B4401 |  |
| 2rdg | NDG | A | 603 |
| 2rdg | SIA | A | 601 |
| 2rdk | MAN | A | 110 |
| 2rdn | 1PL | A | 280 |
| 2rdq | AKG | A | 269 |
| 2rdt | 2RD | A | 365 |
| 2rep | ADP | A | 800 |
| 2rf2 | MRX | A | 561 |
| 2rfb | HEM | A | 410 |
| 2rfc | HEM | D | 410 |
| 2rfh | 23N | A1309 |  |
| 2rfi | SAH | A | 101 |
| 2rfm | BU2 | A | 195 |
| 2rfs | AM8 | A | 1 |
| 2rft | SIA | A3021 |  |
| 2rfu | SIA | A | 801 |
| 2rg0 | CTT | B | 431 |
| 2rg5 | 279 | A | 501 |
| 2rgo | FAD | A1001 |  |
| 2rgz | HEM | A | 300 |
| 2rh1 | MAL | A | 401 |
| 2rh9 | IGP | A | 269 |
| 2rhc | EMO | A | 401 |
| 2rho | GSP | A | 501 |
| 2rhr | EMO | A | 302 |
| 2rht | C1E | A | 287 |
| 2rhw | C0E | A | 288 |
| 2ri1 | GLP | A | 234 |
| 2ri4 | HEM | A | 142 |
| 2ri4 | HEM | B | 147 |
| 2ri9 | MMA | A1901 |  |
| 2rib | GMH | A | 356 |
| 2rij | CIT | A | 389 |
| 2rin | ACH | A | 1 |
| 2rio | ADP | A1101 |  |
| 2rip | 34Q | A | 800 |
| 2rj3 | URA | A8001 |  |
| 2rj4 | AD7 | A | 356 |
| 2rj7 | DA8 | A | 452 |
| 2rjh | DCS | A | 362 |
| 2rjr | 295 | A1001 |  |
| 2rkd | 3PP | A | 703 |
| 2rkf | AB1 | A | 501 |
| 2rkg | AB1 | B | 501 |
| 2rku | TAR | A | 503 |
| 2rkv | MPO | A | 453 |
| 2rkz | SIN | B | 1 |
| 2rl9 | MAN | A3002 |  |
| 2rlc | CHD | A | 332 |
| 2rm2 | W43 | 1 | 900 |
| 2roy | P28 | A | 128 |
| 2rr1 | W8R | 1 | 900 |
| 2rs3 | W59 | 1 | 900 |
| 2rs5 | W56 | 1 | 900 |
| 2rtm | IMI | A | 300 |
| 2sar | 3GP | A | 98 |
| 2sfp | PPI | A | 400 |
| 2skc | FPO | A | 997 |
| 2tct | CTC | A | 222 |
| 2tdd | THF | A | 568 |
| 2tpl | HPP | B | 600 |
| 2tps | TPS | A2001 |  |
| 2udp | UPP | A | 341 |
| 2usn | IN8 | A | 300 |
| 2uue | MTZ | A1297 |  |
| 2uui | PLM | A1156 |  |
| 2uuj | 896 | B1250 |  |
| 2uum | BLA | B1082 |  |
| 2uum | BLA | X1082 |  |
| 2uuo | LK3 | A1441 |  |
| 2uup | LK4 | A1440 |  |
| 2uuq | HEM | A1405 |  |
| 2uus | SCR | A1138 |  |
| 2uuu | FAD | A1587 |  |
| 2uuu | FAD | D1586 |  |
| 2uv8 | FMN | G3051 |  |
| 2uva | FMN | G3079 |  |
| 2uvc | FMN | G3079 |  |
| 2uvn | ECN | A1409 |  |
| 2uvn | HEM | A1408 |  |
| 2uvo | NDG | A1173 |  |
| 2uvo | NDG | B1177 |  |
| 2uvy | GVI | A1351 |  |
| 2uvz | GVJ | A1351 |  |
| 2uw0 | GVK | A1351 |  |
| 2uw4 | L15 | A1351 |  |
| 2uw5 | GVN | A1351 |  |
| 2uw6 | GVO | A1351 |  |
| 2uw7 | GVP | A1351 |  |
| 2uw8 | GVQ | A1351 |  |
| 2uwd | 2GG | A1228 |  |
| 2uwl | 895 | A1245 |  |
| 2uwn | SCR | A1507 |  |
| 2uwo | 701 | A1245 |  |
| 2uwp | 894 | A1245 |  |
| 2ux8 | G1P | B1290 |  |
| 2ux9 | FMN | A1069 |  |
| 2uxh | QUE | A1211 |  |
| 2uxi | G50 | A1211 |  |
| 2uxw | FAD | A | 700 |
| 2uy4 | AZM | A1311 |  |
| 2uyi | K02 | A | 604 |
| 2uyn | 2KT | A1129 |  |
| 2uyp | PPI | B1129 |  |
| 2uyq | SAM | A1311 |  |
| 2uyt | ADP | A1482 |  |
| 2uz9 | XAN | A1453 |  |
| 2uzb | C75 | A1297 |  |
| 2uzd | C85 | A1297 |  |
| 2uze | C95 | A1297 |  |
| 2uzh | IPE | B1160 |  |
| 2uzl | C94 | A1297 |  |
| 2uzn | C96 | A1299 |  |
| 2uzo | C62 | A1297 |  |
| 2uzp | PLP | A1294 |  |
| 2uzt | SS3 | A1351 |  |
| 2uzw | SS4 | E1351 |  |
| 2v03 | CIT | A1297 |  |
| 2v0c | LMS | A1814 |  |
| 2v0d | C53 | A1299 |  |
| 2v0i | UD1 | A1455 |  |
| 2v0k | UDP | A1454 |  |
| 2v0l | URI | A1454 |  |
| 2v0u | FMN | A1547 |  |
| 2v1a | FMN | A1547 |  |
| 2v1d | FAD | A1837 |  |
| 2v1x | ADP | B1593 |  |
| 2v23 | HEM | A1295 |  |
| 2v2a | 13P | A1277 |  |
| 2v2e | HEM | A1295 |  |
| 2v2e | ISZ | A1296 |  |
| 2v34 | CTN | A1269 |  |
| 2v38 | IDR | A1310 |  |
| 2v3a | FAD | A1393 |  |
| 2v3b | FAD | A1385 |  |
| 2v3e | NND | A1502 |  |
| 2v3k | SAM | A1254 |  |
| 2v3v | LCP | A | 814 |
| 2v3v | LCP | A | 815 |
| 2v42 | NDS | A1317 |  |
| 2v4l | ABJ | A2093 |  |
| 2v4m | F6R | A | 800 |
| 2v51 | ATP | B1372 |  |
| 2v52 | ATP | B1377 |  |
| 2v54 | TYD | A1205 |  |
| 2v55 | ANP | A1480 |  |
| 2v58 | LZJ | A1448 |  |
| 2v59 | LZK | A1446 |  |
| 2v5a | LZL | A1447 |  |
| 2v5e | SCR | A1350 |  |
| 2v5k | OXM | A | 302 |
| 2v5w | MCM | I | 6 |
| 2v5z | FAD | A1502 |  |
| 2v5z | SAG | A1503 |  |
| 2v60 | C17 | A1503 |  |
| 2v61 | C18 | A1503 |  |
| 2v6o | FAD | A1594 |  |
| 2v6t | H2B | A1104 |  |
| 2v73 | SIA | A1190 |  |
| 2v77 | PAY | A | 400 |
| 2v7k | DTR | A1361 |  |
| 2v7k | HEM | A1360 |  |
| 2v7p | OXM | A | 501 |
| 2v7q | ADP | D1478 |  |
| 2v7q | ATP | A1511 |  |
| 2v7t | SAH | A1299 |  |
| 2v7w | 5FD | A1299 |  |
| 2v8e | SCR | A1507 |  |
| 2v8h | BCN | A | 601 |
| 2v8p | ADP | A1270 |  |
| 2v95 | HCY | A1375 |  |
| 2v96 | CFQ | A1536 |  |
| 2v9l | PGO | A1281 |  |
| 2v9m | CIT | A1278 |  |
| 2va5 | C8C | A1389 |  |
| 2va6 | H24 | A1504 |  |
| 2va7 | C27 | A1505 |  |
| 2vag | V25 | A1482 |  |
| 2vas | ADP | A | 998 |
| 2vb6 | ADP | A | 998 |
| 2vba | P4T | A1405 |  |
| 2vbv | FMN | A1140 |  |
| 2vbx | HIS | A1151 |  |
| 2vcf | ISZ | X1252 |  |
| 2vcg | S17 | A1378 |  |
| 2vch | UDP | A1477 |  |
| 2vcj | 2EQ | A1224 |  |
| 2vck | BLA | A1234 |  |
| 2vcn | HEM | A1251 |  |
| 2vcn | ISZ | A1254 |  |
| 2vco | MAN | A1161 |  |
| 2vcp | ATP | A1376 |  |
| 2vcq | D25 | A1201 |  |
| 2vcs | HEM | A1251 |  |
| 2vcs | ISZ | A1252 |  |
| 2vct | ASD | A1223 |  |
| 2vcv | ASD | A1224 |  |
| 2vcw | ZZA | B1201 |  |
| 2vcx | D26 | B1201 |  |
| 2vcz | VC3 | B1200 |  |
| 2vd0 | D27 | B1200 |  |
| 2vd1 | D28 | B1200 |  |
| 2vd4 | P21 | A1454 |  |
| 2vd5 | BI8 | A1417 |  |
| 2vd9 | IN5 | A1394 |  |
| 2vdb | DKA | A1585 |  |
| 2vdb | DKA | A1589 |  |
| 2vdb | NPS | A1591 |  |
| 2vdg | 1BO | A1325 |  |
| 2vdg | NDP | A1321 |  |
| 2vdj | HSE | A1298 |  |
| 2vdw | SAH | A1846 |  |
| 2vek | ASF | B1253 |  |
| 2vek | CIT | A1251 |  |
| 2ves | GVR | A1296 |  |
| 2vet | UMP | A1266 |  |
| 2vez | G6P | A1192 |  |
| 2vf7 | ADP | A1842 |  |
| 2vf8 | ADP | A1843 |  |
| 2vfa | 5GP | A1224 |  |
| 2vft | SOR | A1419 |  |
| 2vfz | UPF | A1360 |  |
| 2vg0 | GPP | A1257 |  |
| 2vg1 | FPP | B1262 |  |
| 2vg5 | NNC | A1551 |  |
| 2vg6 | NNB | A1551 |  |
| 2vg7 | NNI | A1551 |  |
| 2vgb | FBP | A | 580 |
| 2vgb | PGA | A | 581 |
| 2vgf | FBP | A | 580 |
| 2vgg | FBP | A | 580 |
| 2vgg | FBP | B | 580 |
| 2vgg | PGA | B | 581 |
| 2vgi | FBP | A1574 |  |
| 2vgi | PGA | B1575 |  |
| 2vgo | AD5 | A1357 |  |
| 2vgr | BLA | A1234 |  |
| 2vhj | ADP | A1301 |  |
| 2vhl | GLP | A1395 |  |
| 2vhx | PYR | A1373 |  |
| 2vhx | PYR | B1373 |  |
| 2vi2 | TAR | A1207 |  |
| 2vig | FAD | A | 600 |
| 2vin | 505 | A1247 |  |
| 2vip | L1R | A1247 |  |
| 2viq | D55 | A1246 |  |
| 2viv | VG2 | A1247 |  |
| 2viw | D56 | A1246 |  |
| 2vj0 | DTD | A1940 |  |
| 2vj2 | MLT | B1336 |  |
| 2vk1 | PYR | A | 602 |
| 2vk1 | PYR | A | 603 |
| 2vk6 | DAN | A2692 |  |
| 2vk8 | 2OP | A1566 |  |
| 2vkh | UPG | A1544 |  |
| 2vkl | MLT | A1090 |  |
| 2vku | DBE | A1446 |  |
| 2vkz | FMN | G3051 |  |
| 2vl8 | CTS | A1544 |  |
| 2vle | DZN | A1501 |  |
| 2vln | MLA | B1135 |  |
| 2vlp | MLA | B1135 |  |
| 2vlq | MLA | B1135 |  |
| 2vmd | MBG | A | 501 |
| 2vmg | MBG | A1179 |  |
| 2vn0 | TDZ | A | 501 |
| 2vn1 | FK5 | A | 501 |
| 2vn8 | CIT | A1398 |  |
| 2vn9 | GVD | A1314 |  |
| 2vng | A2G | A1210 |  |
| 2vnx | HEM | X | 251 |
| 2vnz | HEM | X | 251 |
| 2vo0 | M03 | A1354 |  |
| 2vo2 | HEM | X1250 |  |
| 2vo3 | M04 | A1352 |  |
| 2vo4 | GTB | A1220 |  |
| 2vo6 | M05 | A1352 |  |
| 2voh | CIT | A1151 |  |
| 2voj | 2OP | A | 501 |
| 2vor | ACP | A1490 |  |
| 2vp2 | DGT | A1210 |  |
| 2vp4 | DCP | A1210 |  |
| 2vpa | PYR | A1197 |  |
| 2vpp | GEO | A1210 |  |
| 2vpq | ANP | A1449 |  |
| 2vq5 | HBA | A1203 |  |
| 2vq5 | LDP | B1197 |  |
| 2vqm | HA3 | A1410 |  |
| 2vqq | TFG | A1410 |  |
| 2vr0 | HQO | C1005 |  |
| 2vra | IDS | B1254 |  |
| 2vrj | NCW | A1446 |  |
| 2vrl | MBN | A | 601 |
| 2vro | NDP | A1523 |  |
| 2vsd | NDG | A1100 |  |
| 2vsn | UDP | A | 900 |
| 2vsr | 9HO | A1477 |  |
| 2vst | 243 | A1477 |  |
| 2vt3 | ATP | A | 302 |
| 2vt4 | D10 | A | 406 |
| 2vt4 | P32 | A | 400 |
| 2vt4 | SOG | A | 402 |
| 2vt5 | ROK | A1337 |  |
| 2vtd | LKM | A1441 |  |
| 2vte | LK4 | A1440 |  |
| 2vtf | B3P | A1618 |  |
| 2vth | LZ2 | A1300 |  |
| 2vti | LZ3 | A1299 |  |
| 2vtj | LZ4 | A1300 |  |
| 2vtl | LZ5 | A1299 |  |
| 2vtn | LZ7 | A1299 |  |
| 2vto | LZ8 | A1299 |  |
| 2vtp | LZ9 | A1299 |  |
| 2vtr | LZB | A1299 |  |
| 2vts | LZC | A1299 |  |
| 2vtt | LZD | A1299 |  |
| 2vu1 | OPI | A1393 |  |
| 2vu2 | PN5 | A1393 |  |
| 2vu3 | LZE | A1299 |  |
| 2vue | BLA | A2001 |  |
| 2vuf | FUA | A2001 |  |
| 2vuf | FUA | B2001 |  |
| 2vug | ANP | A | 401 |
| 2vuk | P83 | B1291 |  |
| 2vuv | CIT | A | 500 |
| 2vuw | 5ID | A1800 |  |
| 2vuz | BMA | A1131 |  |
| 2vv1 | 4HD | A1476 |  |
| 2vv1 | 4HD | B1474 |  |
| 2vv2 | 5HE | A1476 |  |
| 2vv4 | 6OB | B1475 |  |
| 2vv6 | HEM | A1258 |  |
| 2vv7 | HEM | A1258 |  |
| 2vv8 | HEM | A1258 |  |
| 2vv9 | IM9 | A1300 |  |
| 2vvl | FAD | A | 600 |
| 2vvl | FAD | G | 600 |
| 2vvm | FAD | A | 600 |
| 2vvp | 5RP | A | 300 |
| 2vvt | I24 | A1269 |  |
| 2vvu | H22 | A1244 |  |
| 2vw1 | DAN | A1000 |  |
| 2vwg | LGC | A | 600 |
| 2vwi | ANP | A1294 |  |
| 2vwn | H25 | A1245 |  |
| 2vwo | LZG | A1245 |  |
| 2vwu | 7X1 | A1888 |  |
| 2vwv | 7X3 | A1888 |  |
| 2vww | 7X2 | A1888 |  |
| 2vwx | 7X4 | A1889 |  |
| 2vwy | 7X5 | A1889 |  |
| 2vwz | 7X6 | A1889 |  |
| 2vx0 | 7X7 | A1889 |  |
| 2vx1 | 7X8 | A1889 |  |
| 2vx3 | D15 | A | 600 |
| 2vxk | 16G | A1191 |  |
| 2vxn | PGA | A1252 |  |
| 2vxn | PGH | A1251 |  |
| 2vxo | XMP | A1694 |  |
| 2vxo | XMP | B1694 |  |
| 2vxv | CXS | H1215 |  |
| 2vxy | CIT | A | 401 |
| 2vyp | ATP | A1375 |  |
| 2vyq | FAD | A | 304 |
| 2vyx | FMN | A1070 |  |
| 2vz2 | C15 | A1504 |  |
| 2vz2 | MFG | A1503 |  |
| 2vz6 | PGO | A1301 |  |
| 2vz7 | HEM | A1407 |  |
| 2vzm | HEM | A1407 |  |
| 2vzw | HEM | A1206 |  |
| 2vzz | SCA | A | 601 |
| 2w00 | ATP | A1886 |  |
| 2w02 | ATP | A1588 |  |
| 2w03 | CIT | B1590 |  |
| 2w05 | FRT | A1299 |  |
| 2w06 | FRV | A1300 |  |
| 2w09 | CM9 | A1451 |  |
| 2w0a | CII | A1450 |  |
| 2w0b | CMW | A1450 |  |
| 2w0d | CGS | A1273 |  |
| 2w0s | BVP | A1207 |  |
| 2w11 | 2OP | A1202 |  |
| 2w15 | WR2 | A1204 |  |
| 2w17 | I19 | A1300 |  |
| 2w1a | TSO | C1091 |  |
| 2w1a | TSO | D1091 |  |
| 2w1b | DXC | A2034 |  |
| 2w1d | L0D | A1391 |  |
| 2w1h | L0H | A1299 |  |
| 2w1i | L0I | A2133 |  |
| 2w1u | A2G | C1949 |  |
| 2w26 | RIV | A1001 |  |
| 2w27 | 5GP | A | 501 |
| 2w39 | BGC | A1300 |  |
| 2w39 | LGC | A1301 |  |
| 2w3b | FOL | A | 401 |
| 2w3d | HEM | A | 500 |
| 2w3f | HEM | B | 502 |
| 2w3g | HEM | A | 500 |
| 2w3h | HEM | B | 502 |
| 2w3r | HPA | B1780 |  |
| 2w3s | FAD | A1465 |  |
| 2w3s | XAN | B1780 |  |
| 2w3v | NDP | A1168 |  |
| 2w3w | NDP | A1169 |  |
| 2w3y | PLM | A1278 |  |
| 2w4i | VGA | B1256 |  |
| 2w4o | DKI | A1338 |  |
| 2w54 | HHR | B1780 |  |
| 2w5a | ADP | A1280 |  |
| 2w5b | SAP | A1280 |  |
| 2w5g | ATP | A1125 |  |
| 2w5i | ATP | A1125 |  |
| 2w5k | NDP | A1125 |  |
| 2w5y | SAH | A4971 |  |
| 2w5z | SAH | A4971 |  |
| 2w62 | BGC | A1490 |  |
| 2w63 | BGC | A1484 |  |
| 2w6m | OA1 | A1447 |  |
| 2w6n | OA2 | A1446 |  |
| 2w6o | OA3 | A1446 |  |
| 2w6p | OA4 | A1447 |  |
| 2w6q | OA5 | A1447 |  |
| 2w6v | HEM | B1147 |  |
| 2w6w | HEM | A1154 |  |
| 2w6x | HEM | A1154 |  |
| 2w6y | HEM | A1154 |  |
| 2w6z | L21 | A1447 |  |
| 2w70 | L22 | A1447 |  |
| 2w71 | L23 | A1448 |  |
| 2w72 | HEM | A1142 |  |
| 2w72 | HEM | B1147 |  |
| 2w74 | ATP | B1887 |  |
| 2w7y | A2G | A1428 |  |
| 2w83 | GTP | A1173 |  |
| 2w8f | BS1 | A1206 |  |
| 2w8h | MTN | A1335 |  |
| 2w8q | SIN | A3001 |  |
| 2w8r | ADP | A | 702 |
| 2w8y | 486 | A1000 |  |
| 2w8y | NDR | B1000 |  |
| 2w98 | P1Z | A1351 |  |
| 2w9g | NDP | A1160 |  |
| 2w9i | MBT | A1540 |  |
| 2w9l | SIA | C1543 |  |
| 2w9s | NDP | A1159 |  |
| 2wab | BGC | A1335 |  |
| 2wah | MAN | A1450 |  |
| 2waj | SNB | A1401 |  |
| 2wal | MLA | A1160 |  |
| 2wao | BGC | A1335 |  |
| 2wax | CXS | A1463 |  |
| 2wax | CXS | D1227 |  |
| 2wba | NDP | A1496 |  |
| 2wbg | LGS | A1446 |  |
| 2wbi | FAD | A | 600 |
| 2wbk | M2F | A1869 |  |
| 2wbp | SIN | A1360 |  |
| 2wbv | SIA | A1545 |  |
| 2wbw | SIA | A1366 |  |
| 2wc3 | AM3 | A1446 |  |
| 2wc3 | AM3 | B1446 |  |
| 2wc4 | AMF | A1446 |  |
| 2wca | NP6 | A | 900 |
| 2wco | GC4 | A1769 |  |
| 2wcq | CIT | A1191 |  |
| 2wd1 | ZZY | A2347 |  |
| 2wd9 | IBP | A1570 |  |
| 2wdq | CBE | C1130 |  |
| 2wdx | BMA | E | 708 |
| 2wdx | GHP | E | 701 |
| 2wdx | GHP | E | 705 |
| 2we5 | ADP | A1311 |  |
| 2wei | VGG | A | 600 |
| 2wek | DIF | A1373 |  |
| 2wel | K88 | A | 600 |
| 2wes | FAD | B | 650 |
| 2wev | CK7 | A1297 |  |
| 2wew | MYR | A1188 |  |
| 2wey | EV1 | A1771 |  |
| 2wf8 | BG6 | A1224 |  |
| 2wf8 | G1P | A1223 |  |
| 2wf9 | G6P | A1223 |  |
| 2wg9 | OCA | A | 301 |
| 2wgh | DTP | A1745 |  |
| 2wgj | VGH | A2346 |  |
| 2wgy | HEM | A | 450 |
| 2wgz | 147 | A1204 |  |
| 2wgz | NPO | B1205 |  |
| 2wh5 | STE | A | 801 |
| 2wh8 | HEM | A | 450 |
| 2wh8 | II2 | A | 460 |
| 2whd | FAD | A | 400 |
| 2whf | HEM | A | 450 |
| 2whf | II4 | A1405 |  |
| 2who | VGI | A1534 |  |
| 2whw | HEM | A1407 |  |
| 2whx | ADP | A1619 |  |
| 2wi1 | ZZ2 | A1225 |  |
| 2wi4 | ZZ4 | A1224 |  |
| 2wi5 | ZZ5 | A1225 |  |
| 2wi6 | ZZ6 | A1224 |  |
| 2wi7 | 2KL | A1224 |  |
| 2wi9 | HEM | A1408 |  |
| 2win | NDG | A1646 |  |
| 2wip | P49 | A1299 |  |
| 2wit | BET | A1001 |  |
| 2wiv | HEM | A1553 |  |
| 2wiy | HEM | A1560 |  |
| 2wjn | MPG | L1277 |  |
| 2wk1 | SAH | A | 301 |
| 2wk2 | NGT | A1565 |  |
| 2wk6 | IUR | A1482 |  |
| 2wk9 | PLG | B | 600 |
| 2wka | P89 | A | 605 |
| 2wkj | PYR | D1297 |  |
| 2wkp | FMN | A1725 |  |
| 2wkp | GTP | A1724 |  |
| 2wkq | FMN | A1723 |  |
| 2wkq | GTP | A1722 |  |
| 2wkr | FMN | A1723 |  |
| 2wkr | GTP | A1722 |  |
| 2wkw | W22 | A | 577 |
| 2wl9 | MBD | B1301 |  |
| 2wlj | SPM | A1303 |  |
| 2wly | NGT | A1572 |  |
| 2wlz | NGT | A1569 |  |
| 2wm0 | NGT | A1564 |  |
| 2wm1 | 13P | A1334 |  |
| 2wm3 | NFL | A1300 |  |
| 2wmd | ZZ0 | A1299 |  |
| 2wmi | A2G | A2006 |  |
| 2wmi | A2G | B2006 |  |
| 2wmk | A2G | A2010 |  |
| 2wmq | ZYQ | A1270 |  |
| 2wmr | ZYR | A1271 |  |
| 2wms | ZYS | A1271 |  |
| 2wmt | ZYT | A1271 |  |
| 2wmu | ZYU | A1271 |  |
| 2wmw | ZYW | A1270 |  |
| 2wmx | ZY6 | A1270 |  |
| 2wn6 | NDP | A1421 |  |
| 2wnb | A2G | A1346 |  |
| 2wne | BGC | A | 402 |
| 2wnf | A2G | A1345 |  |
| 2wnu | IDU | F1218 |  |
| 2wnu | SGN | F1219 |  |
| 2wnz | 2OP | D1296 |  |
| 2wor | 2AN | A1098 |  |
| 2wow | GCG | A1001 |  |
| 2wpd | ADP | D | 600 |
| 2wpd | ATP | A | 600 |
| 2wq9 | OLA | A1179 |  |
| 2wqe | ADP | A1389 |  |
| 2wqn | ADP | A1301 |  |
| 2wqy | OAA | A1002 |  |
| 2wqy | PEE | D | 109 |
| 2wr1 | SIA | C1497 |  |
| 2wr6 | ODT | A1176 |  |
| 2wr8 | SAH | A1257 |  |
| 2wrs | CIT | A1264 |  |
| 2wse | LMU | X7011 |  |
| 2wsi | FAD | A2762 |  |
| 2wss | ADP | D | 600 |
| 2wss | ANP | A | 600 |
| 2wss | ANP | F | 600 |
| 2wsw | CM5 | A1505 |  |
| 2wt1 | BGC | A1691 |  |
| 2wtk | ANP | B | 432 |
| 2wtk | ANP | C | 2 |
| 2wtx | UDP | A1460 |  |
| 2wtz | UAG | A1498 |  |
| 2wtz | UAG | C1498 |  |
| 2wu1 | 16G | B1567 |  |
| 2wu6 | DKI | A1484 |  |
| 2wug | HPK | A1290 |  |
| 2wuu | NHM | A1422 |  |
| 2wuz | HEM | A1450 |  |
| 2wuz | TPF | A1460 |  |
| 2wv2 | HEM | A1450 |  |
| 2wv2 | TPF | A | 1 |
| 2wve | CIT | A1148 |  |
| 2wvu | 147 | A2001 |  |
| 2wvz | KIF | A | 801 |
| 2ww2 | SWA | B | 900 |
| 2ww4 | ADP | A1284 |  |
| 2ww5 | CHT | A | 701 |
| 2wwc | CHT | A | 708 |
| 2wwf | ADP | A | 212 |
| 2wwf | TMP | A | 211 |
| 2wwg | ADP | A | 212 |
| 2wwg | ATM | A | 211 |
| 2wwh | T5A | A | 211 |
| 2wwi | ADP | A | 212 |
| 2wwi | ATM | A | 211 |
| 2wwu | BGC | A1492 |  |
| 2wx2 | HEM | B1450 |  |
| 2wx2 | TPF | B1460 |  |
| 2wxf | 039 | A1500 |  |
| 2wy4 | HEM | A | 150 |
| 2wyi | SWA | A1907 |  |
| 2wyw | TCL | A1261 |  |
| 2wzb | 3PG | A1420 |  |
| 2wzb | ADP | A1419 |  |
| 2wzf | BGC | A1526 |  |
| 2wzf | UDP | A1525 |  |
| 2wzg | UPG | A1525 |  |
| 2wzv | FMN | A | 801 |
| 2wzw | FMN | A | 801 |
| 2wzw | NDP | A | 901 |
| 2x07 | HEM | A1295 |  |
| 2x08 | HEM | A1254 |  |
| 2x0e | TYD | A | 414 |
| 2x0f | TRH | A | 416 |
| 2x0j | ENA | A1000 |  |
| 2x0l | FAD | A | 900 |
| 2x0q | ATP | A1607 |  |
| 2x1d | D1D | D1359 |  |
| 2x1l | CXS | A | 602 |
| 2x1t | RES | B1251 |  |
| 2x20 | CHL | M | 601 |
| 2x21 | BCL | M | 601 |
| 2x21 | DGD | M | 615 |
| 2x2g | 3PG | B1251 |  |
| 2x3j | ATP | A1589 |  |
| 2x41 | BGC | A1726 |  |
| 2x4a | CXS | A1686 |  |
| 2x58 | ADP | A | 750 |
| 2x5d | PLP | D1400 |  |
| 2x5f | PLP | A1429 |  |
| 2x5l | HEM | A1434 |  |
| 2x60 | GTP | A | 601 |
| 2x61 | SIA | A1261 |  |
| 2x65 | M1P | A | 600 |
| 2x6j | 093 | A1949 |  |
| 2x6t | ADP | A1320 |  |
| 2x6t | BMA | A1321 |  |
| 2x6w | NDG | A | 724 |
| 2x7f | 824 | A | 500 |
| 2x7i | CIT | A1307 |  |
| 2x7w | BCN | A1290 |  |
| 2x7x | FRU | A1335 |  |
| 2x8o | OIN | A1314 |  |
| 2x8p | CHT | A1317 |  |
| 2x8p | OIN | A1313 |  |
| 2x91 | LPR | A1615 |  |
| 2x9g | LYA | A1270 |  |
| 2x9g | LYA | B1270 |  |
| 2x9i | BLA | A1234 |  |
| 2x9j | BLA | A1234 |  |
| 2x9n | D1D | B1271 |  |
| 2x9n | DTD | A1271 |  |
| 2x9o | BLA | A1234 |  |
| 2x9p | HEM | A1398 |  |
| 2x9v | TMQ | A1270 |  |
| 2xa2 | UPG | A1415 |  |
| 2xa5 | SLB | A1310 |  |
| 2xad | GHP | F | 701 |
| 2xaf | FAD | A | 900 |
| 2xag | FAD | A | 900 |
| 2xah | FAD | A | 900 |
| 2xaj | FAD | A | 900 |
| 2xal | ADP | A | 600 |
| 2xam | ADP | A | 600 |
| 2xan | ANP | A | 600 |
| 2xaq | FAD | A | 900 |
| 2xas | FAD | A | 900 |
| 2xat | DCA | A | 302 |
| 2xau | ADP | A1750 |  |
| 2xbk | HEM | A1398 |  |
| 2xbl | M7P | A1198 |  |
| 2xbn | PMP | A1420 |  |
| 2xbp | ATP | A1114 |  |
| 2xc3 | HEM | A1433 |  |
| 2xce | DUP | B1131 |  |
| 2xcg | C15 | A | 603 |
| 2xcm | ADP | A1211 |  |
| 2xcu | CIT | A1355 |  |
| 2xcu | CIT | B1354 |  |
| 2xcw | ATP | A1489 |  |
| 2xcw | IMP | A1490 |  |
| 2xdr | NDP | A5502 |  |
| 2xdw | TAM | A1711 |  |
| 2xe4 | PGO | A1743 |  |
| 2xe5 | D10 | B1344 |  |
| 2xe5 | OES | B1352 |  |
| 2xe8 | ACP | A1418 |  |
| 2xeu | SUC | A1067 |  |
| 2xfd | CBI | A1113 |  |
| 2xfn | C15 | A1502 |  |
| 2xfp | C15 | A1503 |  |
| 2xfp | ISN | A | 601 |
| 2xfq | C15 | A1503 |  |
| 2xfq | RAS | A | 601 |
| 2xfy | ACX | A | 600 |
| 2xg5 | PGO | B1174 |  |
| 2xga | MTN | A1019 |  |
| 2xh0 | PEP | A1440 |  |
| 2xh4 | 2PG | A1440 |  |
| 2xhk | AKG | A | 519 |
| 2xi6 | HEM | A | 288 |
| 2xif | HEM | A1251 |  |
| 2xig | CIT | B1149 |  |
| 2xih | HEM | A1251 |  |
| 2xik | J60 | A1294 |  |
| 2xil | HEM | A1301 |  |
| 2xiq | MLC | A5001 |  |
| 2xiu | MTN | A1055 |  |
| 2xj5 | HEM | A1295 |  |
| 2xj6 | HEM | A1001 |  |
| 2xj8 | HEM | A1295 |  |
| 2xj9 | SAP | A1272 |  |
| 2xja | ADP | A1533 |  |
| 2xjb | DTP | A1490 |  |
| 2xjc | 5GP | A1489 |  |
| 2xjp | BMA | A1278 |  |
| 2xjr | MAN | A1278 |  |
| 2xjs | MAN | A1277 |  |
| 2xjt | MAN | A1279 |  |
| 2xju | MAN | A1278 |  |
| 2xjv | BGC | A1277 |  |
| 2xk2 | ADP | A1226 |  |
| 2xki | HEM | A1110 |  |
| 2xko | AKG | A1223 |  |
| 2xkp | AKG | A1223 |  |
| 2xmf | DIA | A1055 |  |
| 2xn5 | FUN | A1356 |  |
| 2xn8 | HEM | A1434 |  |
| 2xoc | ADE | B1664 |  |
| 2xoc | ADP | A1665 |  |
| 2xod | FMN | A1119 |  |
| 2xok | ANP | A | 600 |
| 2xok | ANP | D | 600 |
| 2xoy | ADE | B1666 |  |
| 2xp2 | VGH | A9000 |  |
| 2xp3 | B21 | A1165 |  |
| 2xp4 | G14 | A1165 |  |
| 2xqr | FRU | A1000 |  |
| 2xqr | FRU | E1000 |  |
| 2xr5 | MAN | A1384 |  |
| 2xr6 | MAN | A1385 |  |
| 2xr7 | MLC | A1001 |  |
| 2xrf | URA | A | 401 |
| 2xrq | SIA | D1108 |  |
| 2xry | FAD | A1463 |  |
| 2xsg | B3P | A1773 |  |
| 2xsi | MYR | A1001 |  |
| 2xsp | XYS | A1444 |  |
| 2xsq | IMP | A1183 |  |
| 2xsz | ATP | A | 501 |
| 2xsz | ATP | D | 501 |
| 2xu2 | CIT | A1249 |  |
| 2xua | SHF | H1265 |  |
| 2xud | DME | A1544 |  |
| 2xui | TZ5 | A1545 |  |
| 2xva | SFG | A1198 |  |
| 2xvj | IND | A1450 |  |
| 2xvm | SAH | A1198 |  |
| 2xvv | MYR | A1001 |  |
| 2xvw | MYR | A1001 |  |
| 2xvx | HEM | A1270 |  |
| 2xw7 | NDP | A1173 |  |
| 2xwd | LGS | A1510 |  |
| 2xwe | AMF | A1510 |  |
| 2xwl | CTP | A | 301 |
| 2xxj | OXM | B1310 |  |
| 2xyq | SAH | A1293 |  |
| 2xz5 | ACH | A1210 |  |
| 2xz7 | PEP | A6080 |  |
| 2xz9 | PYR | A6450 |  |
| 2y05 | RAL | A | 801 |
| 2y0c | UGA | A | 501 |
| 2y1w | SFG | A1001 |  |
| 2y24 | GCV | A1004 |  |
| 2y27 | ATP | A1440 |  |
| 2y33 | UN9 | A | 901 |
| 2y34 | UN9 | A | 601 |
| 2y37 | HEM | A1501 |  |
| 2y3f | BTN | A1136 |  |
| 2y3q | HEM | A | 200 |
| 2y42 | BCN | A | 501 |
| 2y4e | PP9 | A1389 |  |
| 2y4i | ATP | B1932 |  |
| 2y5a | HEM | A1001 |  |
| 2y69 | CHD | G1085 |  |
| 2y69 | CHD | P1265 |  |
| 2y6a | HEM | A | 251 |
| 2y6b | HEM | A | 251 |
| 2y6r | CTC | A1385 |  |
| 2y6r | CTC | B1385 |  |
| 2y7j | B49 | A1294 |  |
| 2y7p | SAL | A1000 |  |
| 2y85 | 137 | A1247 |  |
| 2y8h | HEM | B | 502 |
| 2ya7 | ZMR | C1776 |  |
| 2ya8 | G39 | A1777 |  |
| 2ya9 | D1D | A1302 |  |
| 2yay | DUP | A1265 |  |
| 2yaz | UMP | A1265 |  |
| 2yb0 | DUR | A1266 |  |
| 2ycg | HEM | A | 295 |
| 2ych | ATP | A | 502 |
| 2yck | THL | X7216 |  |
| 2ycx | P32 | A1355 |  |
| 2ycx | SOG | B1360 |  |
| 2ycy | P32 | A | 400 |
| 2ycy | SOG | A1360 |  |
| 2ycz | SOG | B1360 |  |
| 2yd8 | SCR | A1228 |  |
| 2yd9 | B3P | A1325 |  |
| 2ydn | BCN | A | 400 |
| 2ydv | NEC | A | 400 |
| 2ydv | SOG | A | 501 |
| 2ydx | STL | A | 501 |
| 2ydx | STL | B | 502 |
| 2ye3 | VXX | A1224 |  |
| 2yed | ADE | A1225 |  |
| 2yef | ANP | A1228 |  |
| 2yfx | VGH | A9000 |  |
| 2yg3 | FAD | A | 600 |
| 2yg4 | 4HA | A | 601 |
| 2yg4 | FAD | A | 600 |
| 2yg5 | FAD | A | 600 |
| 2yg6 | FAD | A | 600 |
| 2yg7 | FAD | A | 600 |
| 2ygg | TAM | B1156 |  |
| 2ygp | SPD | A1216 |  |
| 2ygq | SCR | A1279 |  |
| 2yih | BGC | A1540 |  |
| 2yih | XYS | A1542 |  |
| 2yiz | FMN | A1070 |  |
| 2yj0 | FMN | A | 300 |
| 2yje | ATP | A | 377 |
| 2yjf | ATP | A1377 |  |
| 2yjq | BGC | A1519 |  |
| 2yl4 | ACP | A1719 |  |
| 2yl8 | MAN | A1627 |  |
| 2yl9 | MAN | A2042 |  |
| 2yla | MAN | A2045 |  |
| 2yqu | FAD | A1601 |  |
| 2yr4 | FAD | A | 801 |
| 2yr5 | FAD | A | 801 |
| 2yr6 | FAD | A | 801 |
| 2yri | PMP | B | 501 |
| 2yrs | HEM | B | 148 |
| 2yu1 | AKG | A | 701 |
| 2yve | MBT | A1001 |  |
| 2yw2 | ATP | A | 646 |
| 2ywc | XMP | A | 701 |
| 2ywg | GTP | A | 701 |
| 2ywp | A42 | A | 1 |
| 2yww | ATP | A | 402 |
| 2yx1 | SFG | A | 401 |
| 2yx6 | ADP | C1001 |  |
| 2yxx | PLP | A | 401 |
| 2yy1 | NLC | A | 161 |
| 2yy8 | MTA | A | 400 |
| 2yy8 | SAM | B | 500 |
| 2yye | APC | A | 501 |
| 2yyi | FAD | A | 500 |
| 2yz3 | M5P | A | 401 |
| 2yzc | 1AL | A | 303 |
| 2yzj | DUD | A6194 |  |
| 2yzn | ANP | A | 401 |
| 2yzq | SAM | A6075 |  |
| 2z08 | ATP | A1001 |  |
| 2z09 | ACP | A2001 |  |
| 2z0a | SIN | A | 75 |
| 2z0k | A5A | A | 600 |
| 2z0x | 5CA | A | 200 |
| 2z0y | SAM | A | 300 |
| 2z0y | SAM | B | 400 |
| 2z1a | THM | A6510 |  |
| 2z1q | FAD | A | 700 |
| 2z1s | CTT | A | 500 |
| 2z1u | ATP | A | 342 |
| 2z1w | BDI | A | 600 |
| 2z1z | MLT | A | 433 |
| 2z1z | PLP | A | 500 |
| 2z20 | PLP | A | 701 |
| 2z26 | DOR | A1410 |  |
| 2z26 | NCD | B1420 |  |
| 2z2c | UDP | A | 950 |
| 2z2p | DOL | A2002 |  |
| 2z2w | 770 | A | 901 |
| 2z36 | HEM | A | 450 |
| 2z3k | XYA | A | 301 |
| 2z3o | TAR | A | 501 |
| 2z3t | HEM | A | 500 |
| 2z3u | CRR | A | 501 |
| 2z3u | HEM | A | 500 |
| 2z3v | PGO | A1000 |  |
| 2z49 | AMG | B1405 |  |
| 2z4x | 252 | A1101 |  |
| 2z4z | SC0 | A1201 |  |
| 2z50 | 028 | A1201 |  |
| 2z52 | H23 | A1001 |  |
| 2z52 | H23 | B1002 |  |
| 2z54 | AB1 | A | 200 |
| 2z5x | DCX | A | 1 |
| 2z5x | HRM | A | 700 |
| 2z5y | DCX | A | 1 |
| 2z5y | HRM | A | 700 |
| 2z60 | P3Y | A | 1 |
| 2z62 | FUL | A | 702 |
| 2z6c | FMN | A | 500 |
| 2z6d | FMN | A | 500 |
| 2z6f | HEM | A3747 |  |
| 2z6i | FMN | A | 401 |
| 2z6j | TUI | A | 501 |
| 2z6r | SAH | A1300 |  |
| 2z6v | PLM | A | 700 |
| 2z6w | CIT | A3001 |  |
| 2z71 | PNV | A | 903 |
| 2z77 | HE7 | A | 200 |
| 2z7g | EH9 | A | 356 |
| 2z7i | 742 | A1302 |  |
| 2z7k | BGU | A | 400 |
| 2z7l | S91 | A1001 |  |
| 2z86 | UDP | B | 1 |
| 2z86 | UGA | A | 683 |
| 2z87 | UD2 | A | 683 |
| 2z8a | HEM | A1147 |  |
| 2z8f | BGC | A | 4 |
| 2z8g | BGC | A2003 |  |
| 2z8l | SIA | A | 601 |
| 2z92 | ENE | A | 1 |
| 2z9c | DTC | A | 202 |
| 2za5 | 2FF | A | 4 |
| 2zaa | MAW | A | 404 |
| 2zac | LGU | A | 401 |
| 2zan | ATP | A | 446 |
| 2zao | ADP | A | 445 |
| 2zas | 1OH | A | 460 |
| 2zaz | GK1 | A | 362 |
| 2zb0 | GK3 | A | 361 |
| 2zb1 | GK4 | A | 361 |
| 2zb2 | A46 | A | 850 |
| 2zb4 | 5OP | A | 600 |
| 2zb7 | NCA | A | 901 |
| 2zbh | BVL | A | 134 |
| 2zbl | BMA | A | 501 |
| 2zbr | SFG | A | 300 |
| 2zbw | FAD | A1002 |  |
| 2zbx | HEM | A | 413 |
| 2zby | HEM | A | 413 |
| 2zbz | HEM | A | 413 |
| 2zc9 | 22U | H1501 |  |
| 2zcd | ANP | A5403 |  |
| 2zce | ANP | A | 501 |
| 2zcp | FPS | A | 657 |
| 2zcq | B65 | A | 451 |
| 2zcs | B70 | A | 640 |
| 2zd1 | T27 | A | 557 |
| 2zd8 | MER | A | 401 |
| 2zda | 32U | H1000 |  |
| 2zdg | ADP | A | 401 |
| 2zdh | ADP | A | 701 |
| 2zdk | 50U | A | 501 |
| 2zdl | 45U | A | 501 |
| 2zdm | 46U | A | 501 |
| 2zdn | 49U | A | 501 |
| 2zdq | ATP | A1501 |  |
| 2zdv | 37U | H | 501 |
| 2zdx | P4A | A | 500 |
| 2ze2 | T27 | A | 556 |
| 2ze3 | AKG | A | 276 |
| 2ze6 | DST | A | 400 |
| 2ze7 | DST | A | 400 |
| 2zeb | 11M | A | 244 |
| 2zec | 11N | A | 1 |
| 2zet | GTP | A | 203 |
| 2zey | BMA | A | 147 |
| 2zey | MAN | B | 147 |
| 2zf0 | 51U | H | 501 |
| 2zf4 | PPY | A | 192 |
| 2zfb | HEM | A | 150 |
| 2zfb | HEM | B | 150 |
| 2zfe | L1P | A | 280 |
| 2zff | 53U | H2001 |  |
| 2zfp | 19U | H | 801 |
| 2zfq | 45U | H | 501 |
| 2zfr | 46U | H | 501 |
| 2zfs | 12U | A | 501 |
| 2zft | 10U | A | 501 |
| 2zfu | SAH | A | 900 |
| 2zg0 | 50U | H | 701 |
| 2zgb | 21U | H1801 |  |
| 2zgx | 29U | H1601 |  |
| 2zhd | 12U | A | 501 |
| 2zhe | 13U | H | 501 |
| 2zhf | 49U | H | 501 |
| 2zhq | 27U | H | 501 |
| 2zhw | 12U | H | 501 |
| 2zi2 | 24U | H | 801 |
| 2zi3 | ADP | A | 301 |
| 2zi4 | ADP | A | 301 |
| 2zi5 | UDP | A1301 |  |
| 2zi6 | UDP | A1301 |  |
| 2zi7 | UDP | A | 301 |
| 2zi8 | SDT | A | 702 |
| 2zi9 | ADP | A | 301 |
| 2zia | UDP | A | 301 |
| 2zin | ANP | A | 501 |
| 2ziq | 26U | H | 701 |
| 2ziz | AD3 | A | 500 |
| 2zj0 | 2FA | A | 500 |
| 2zj3 | G6P | A | 1 |
| 2zj5 | ADP | A | 801 |
| 2zja | ACP | A | 901 |
| 2zjf | BSU | A | 360 |
| 2zjh | F1H | A | 449 |
| 2zji | F1I | A | 449 |
| 2zjj | F1J | A | 449 |
| 2zjk | F1K | A | 449 |
| 2zjl | F1L | A | 449 |
| 2zjw | REF | A | 336 |
| 2zk2 | GSH | A | 1 |
| 2zk6 | C08 | A | 1 |
| 2zkc | BPZ | A | 999 |
| 2zkj | ADP | A | 501 |
| 2zm1 | KSF | A | 513 |
| 2zm3 | 575 | A | 1 |
| 2zm4 | KSM | A | 604 |
| 2zmb | PXB | A | 692 |
| 2zmd | 537 | A | 900 |
| 2zmf | CMP | A2001 |  |
| 2znk | 31U | H3001 |  |
| 2znp | B7G | A | 921 |
| 2znq | 401 | A | 922 |
| 2znq | B7G | A | 921 |
| 2zoe | SLB | B2000 |  |
| 2zog | BES | A1080 |  |
| 2zoq | 5ID | A | 382 |
| 2zpa | ADP | A | 800 |
| 2zpt | A3P | X | 301 |
| 2zq1 | 11U | A | 501 |
| 2zq2 | 13U | A | 501 |
| 2zqm | CIT | A | 300 |
| 2zqn | BGC | A | 301 |
| 2zr1 | NDG | B | 268 |
| 2zrl | DTP | A | 600 |
| 2zrw | IPE | A | 701 |
| 2zrz | DMA | A | 701 |
| 2zsc | BTN | A | 301 |
| 2zsf | ATP | A | 651 |
| 2zsn | HEM | A | 154 |
| 2zso | HEM | A | 154 |
| 2zsp | HEM | A | 154 |
| 2zsq | HEM | A | 154 |
| 2zsr | HEM | A | 154 |
| 2zss | HEM | A | 154 |
| 2zst | HEM | A | 154 |
| 2zsx | HEM | A | 154 |
| 2zsy | HEM | A | 154 |
| 2zsz | HEM | A | 154 |
| 2zt0 | HEM | A | 154 |
| 2zt1 | HEM | A | 154 |
| 2zt2 | HEM | A | 154 |
| 2zt3 | HEM | A | 154 |
| 2zt4 | HEM | A | 154 |
| 2zt6 | APC | A | 699 |
| 2zt7 | ATP | A1200 |  |
| 2zt9 | HEM | C | 301 |
| 2zt9 | UMQ | A | 304 |
| 2ztg | A5A | A | 801 |
| 2ztj | AKG | A | 383 |
| 2zul | SAM | A | 376 |
| 2zut | A2G | A4001 |  |
| 2zuu | NDG | A4001 |  |
| 2zuv | NDG | A2001 |  |
| 2zuw | NDG | A4001 |  |
| 2zv8 | ANP | A | 1 |
| 2zv9 | PP2 | A | 513 |
| 2zvj | KOM | A | 302 |
| 2zvp | NPO | X1201 |  |
| 2zw7 | BLM | A | 402 |
| 2zw9 | SAM | A | 801 |
| 2zwa | CIT | A | 802 |
| 2zwa | SAH | A | 801 |
| 2zwj | HEM | A | 153 |
| 2zws | PLM | A | 652 |
| 2zwv | SAH | A | 376 |
| 2zxc | 2ED | A | 701 |
| 2zxg | S23 | A | 901 |
| 2zyb | KSL | A | 511 |
| 2zyd | GLO | A3929 |  |
| 2zyi | STE | A | 500 |
| 2zym | ACX | A | 901 |
| 2zyn | BCD | A | 901 |
| 2zyt | PPS | X | 501 |
| 2zz0 | FAD | C | 900 |
| 2zz1 | BMP | A | 301 |
| 2zz7 | BMP | A | 301 |
| 2zza | FOL | A | 164 |
| 2zzb | FAD | C | 500 |
| 2zzb | TPT | B | 501 |
| 2zzd | FRU | B6002 |  |
| 2zzd | FRU | L6001 |  |
| 2zzg | A5A | A | 999 |
| 2zzj | CIT | A | 240 |
| 2zzk | CIT | A | 801 |
| 2zzl | L1P | A | 280 |
| 2zzl | SOG | A | 430 |
| 3a06 | FOM | A | 900 |
| 3a06 | FOM | B | 901 |
| 3a06 | NDP | A3001 |  |
| 3a06 | NDP | A3003 |  |
| 3a09 | MAV | A | 503 |
| 3a09 | MAW | A | 501 |
| 3a0b | DGD | C1056 |  |
| 3a0d | MMA | A | 111 |
| 3a0g | HEM | A | 201 |
| 3a0g | HEM | B | 401 |
| 3a0i | AJI | X | 501 |
| 3a0n | BDP | A | 301 |
| 3a0t | ADP | A | 801 |
| 3a0w | EMT | A | 803 |
| 3a14 | NDP | A3003 |  |
| 3a15 | HEM | A | 354 |
| 3a16 | HEM | A | 354 |
| 3a17 | HEM | A | 354 |
| 3a18 | HEM | A | 354 |
| 3a1c | ACP | A | 997 |
| 3a1d | ADP | A | 997 |
| 3a1e | ACP | A | 997 |
| 3a1j | SUC | A6001 |  |
| 3a1l | 2CC | A | 501 |
| 3a1l | HEM | A | 500 |
| 3a26 | MTA | A | 279 |
| 3a27 | SAM | A | 250 |
| 3a2g | HEM | A | 154 |
| 3a2l | SUC | A2381 |  |
| 3a2q | ACA | A | 601 |
| 3a2u | ANP | X | 342 |
| 3a2y | TS5 | A | 198 |
| 3a3b | FMN | B | 191 |
| 3a4g | HEM | A | 412 |
| 3a4h | HEM | A | 412 |
| 3a4l | ANP | A | 301 |
| 3a4t | SFG | A | 500 |
| 3a4v | PYR | A6450 |  |
| 3a4x | NDG | A | 4 |
| 3a4z | HEM | A | 412 |
| 3a50 | HEM | A | 412 |
| 3a51 | HEM | A | 412 |
| 3a56 | CIT | A | 306 |
| 3a59 | HEM | A | 150 |
| 3a59 | HEM | B | 150 |
| 3a5a | HEM | A | 153 |
| 3a5b | HEM | A | 153 |
| 3a5l | ADP | C | 377 |
| 3a5m | ATP | C | 376 |
| 3a5n | ATP | C | 376 |
| 3a5o | ATP | C | 376 |
| 3a5r | HC4 | A | 400 |
| 3a6r | FMN | A | 123 |
| 3a6t | 8OG | A | 134 |
| 3a6t | SUC | A | 130 |
| 3a6u | 8OG | A5001 |  |
| 3a70 | NPO | A | 630 |
| 3a73 | MYR | A1002 |  |
| 3a74 | LYN | A | 495 |
| 3a76 | SPD | A | 300 |
| 3a7b | NDG | A | 821 |
| 3a7i | ADE | A | 500 |
| 3a7k | L1P | A | 293 |
| 3a8i | C2F | A | 401 |
| 3a8t | ATP | A | 800 |
| 3a8u | PLP | X | 450 |
| 3a8w | ATP | A | 601 |
| 3a9b | CBI | A1002 |  |
| 3a9m | HEM | A | 153 |
| 3a9t | FOC | A | 601 |
| 3aar | ANP | A | 1 |
| 3ab1 | FAD | A | 361 |
| 3ab8 | ATP | A | 510 |
| 3aba | HEM | A1408 |  |
| 3abb | HEM | A1430 |  |
| 3abv | EPH | D1306 |  |
| 3acc | 5GP | A | 817 |
| 3acd | IMP | A | 552 |
| 3ach | BGC | A | 401 |
| 3act | BGC | A2901 |  |
| 3ad7 | MTG | B | 801 |
| 3ad8 | PYC | B | 801 |
| 3ae1 | EPH | D1306 |  |
| 3ae2 | EPH | D1306 |  |
| 3ae2 | FAD | A | 700 |
| 3ae3 | EPH | D1306 |  |
| 3ae4 | EPH | D1306 |  |
| 3ae4 | FAD | A | 700 |
| 3ae5 | FAD | A | 700 |
| 3ae8 | EPH | D1306 |  |
| 3ae9 | EPH | D1306 |  |
| 3ae9 | FAD | A | 700 |
| 3aeb | HEM | C1305 |  |
| 3aec | FAD | A | 700 |
| 3af2 | ACP | A | 313 |
| 3afk | A2G | A | 241 |
| 3afv | HEM | A | 501 |
| 3ag2 | CHD | B1085 |  |
| 3ag2 | CHD | J | 60 |
| 3agj | GTP | A | 438 |
| 3agt | CFO | A | 201 |
| 3ah1 | SLB | A2000 |  |
| 3ahd | HTL | A | 827 |
| 3aia | SAM | A | 206 |
| 3air | DNF | A | 800 |
| 3aje | ANP | A | 354 |
| 3ajg | BLA | A1131 |  |
| 3ajn | GM1 | A | 130 |
| 3ajy | BGC | C | 270 |
| 3akk | ADP | A | 500 |
| 3akl | ADP | C | 500 |
| 3akl | ANP | A | 601 |
| 3aln | ANP | A | 1 |
| 3alo | ANP | A | 406 |
| 3alp | CIT | A | 1 |
| 3aml | SIN | A | 766 |
| 3aml | SIN | A | 789 |
| 3amm | CTT | A | 901 |
| 3amn | CBI | A | 902 |
| 3amr | IHS | A | 900 |
| 3amv | BIN | A | 930 |
| 3amz | URC | A1339 |  |
| 3an1 | FAD | A3006 |  |
| 3an1 | URC | A1333 |  |
| 3ang | DCC | A1002 |  |
| 3ank | GCD | A | 400 |
| 3ank | NG6 | A | 399 |
| 3anp | DCC | A1001 |  |
| 3anr | HRM | A | 1 |
| 3ao2 | DTV | A | 213 |
| 3ao5 | SUC | A | 1 |
| 3aof | BMA | B | 401 |
| 3aou | UMQ | C1001 |  |
| 3aow | AKG | A | 430 |
| 3ap7 | SIA | A | 156 |
| 3aqv | TAK | A | 1 |
| 3aqx | BGC | A | 702 |
| 3aqz | BGC | A | 703 |
| 3ar2 | ACP | A1002 |  |
| 3ar3 | ADP | A1002 |  |
| 3ar4 | ATP | A1002 |  |
| 3ar7 | 128 | A1002 |  |
| 3arc | DGD | c | 657 |
| 3arc | DGD | c | 661 |
| 3arc | DGD | d | 755 |
| 3arc | DGD | h | 663 |
| 3arc | HEM | f | 641 |
| 3arc | HTG | b | 696 |
| 3arc | HTG | b | 698 |
| 3arc | HTG | b | 719 |
| 3arc | HTG | d | 726 |
| 3arc | HTG | V1001 |  |
| 3arc | LHG | d | 664 |
| 3arc | LHG | d | 702 |
| 3arc | LHG | d | 714 |
| 3arc | LHG | e | 772 |
| 3arj | HEM | A | 153 |
| 3ark | HEM | A | 153 |
| 3arp | DEQ | A | 606 |
| 3at5 | HEM | A | 142 |
| 3at5 | HEM | B | 147 |
| 3at6 | HEM | A | 142 |
| 3at6 | HEM | B | 147 |
| 3atm | TSS | A | 5 |
| 3atq | C14 | A | 502 |
| 3att | ATP | A | 510 |
| 3au8 | NDP | A | 501 |
| 3au8 | NDP | B | 502 |
| 3au9 | FOM | A | 701 |
| 3au9 | NDP | A | 501 |
| 3av5 | SAH | A | 1 |
| 3av6 | SAM | A | 1 |
| 3awm | PLM | A | 601 |
| 3axs | SFG | A | 501 |
| 3aya | A2G | A | 252 |
| 3ayd | A2G | A | 252 |
| 3ayi | FAD | A | 801 |
| 3ayi | HCI | A | 907 |
| 3ayj | FAD | A | 801 |
| 3ayl | FAD | A | 801 |
| 3azr | BGC | A | 401 |
| 3azs | BMA | A | 402 |
| 3azs | MAN | A | 401 |
| 3azt | BGC | A | 401 |
| 3b0r | GW9 | A | 1 |
| 3b2q | AES | B | 462 |
| 3b2q | CIT | B | 463 |
| 3b2s | MPO | A | 456 |
| 3b30 | MPO | A | 453 |
| 3b3c | PLU | A | 500 |
| 3b3k | LRG | A | 1 |
| 3b3o | HEM | A | 750 |
| 3b3o | JI3 | A | 800 |
| 3b3p | HEM | A | 750 |
| 3b3p | JI7 | A | 800 |
| 3b4f | TUO | A | 300 |
| 3b4p | 3B4 | A | 500 |
| 3b56 | DIU | A2000 |  |
| 3b5r | B5R | A | 1 |
| 3b65 | 3B6 | A | 1 |
| 3b66 | B66 | A | 1 |
| 3b67 | B67 | A | 1 |
| 3b68 | B68 | A | 1 |
| 3b6c | SDN | A | 301 |
| 3b6h | HEM | B | 600 |
| 3b6h | MXD | A | 551 |
| 3b6h | MXD | B | 551 |
| 3b6l | SDS | A2300 |  |
| 3b6r | CRN | B | 603 |
| 3b6t | QUS | A | 801 |
| 3b74 | PEE | A | 311 |
| 3b75 | FRU | A | 142 |
| 3b75 | HEM | A | 150 |
| 3b7e | ZMR | A1001 |  |
| 3b7g | ANP | A | 300 |
| 3b7o | MLT | A | 1 |
| 3b7p | SPM | A | 501 |
| 3b7r | BIR | L1001 |  |
| 3b7u | KEL | X | 707 |
| 3b89 | 5GP | A | 500 |
| 3b8c | ACP | A1001 |  |
| 3b8r | 887 | A | 201 |
| 3b8z | 294 | A | 801 |
| 3b92 | 440 | A | 501 |
| 3b96 | FAD | A | 616 |
| 3b96 | MYA | A | 1 |
| 3b98 | HEM | A | 600 |
| 3b99 | HEM | A | 600 |
| 3b99 | U51 | A | 700 |
| 3b9f | IDS | H | 2 |
| 3b9f | SGN | H | 1 |
| 3b9l | MYR | A1001 |  |
| 3b9m | MYR | A1001 |  |
| 3b9m | SAL | A1200 |  |
| 3b9o | FMN | A | 441 |
| 3b9r | ACP | A | 998 |
| 3b9x | TAM | D | 402 |
| 3ba0 | HAE | A | 477 |
| 3ba8 | 3B8 | A | 501 |
| 3ba9 | 3B9 | A | 501 |
| 3baa | 3BA | A | 501 |
| 3bab | 3BD | A | 501 |
| 3baf | ANP | A | 700 |
| 3bb5 | CIT | B | 103 |
| 3bbh | SFG | A | 206 |
| 3bbp | GTP | A | 500 |
| 3bbr | BHY | A | 268 |
| 3bbt | FMM | B | 91 |
| 3bc4 | LLG | A2501 |  |
| 3bc9 | ACI | A | 803 |
| 3bc9 | BGC | A | 809 |
| 3bc9 | G6D | A | 801 |
| 3bcj | FIS | A | 320 |
| 3bcu | THM | A | 940 |
| 3bdl | CIT | A2000 |  |
| 3bdl | CIT | A2002 |  |
| 3be0 | CNL | B | 500 |
| 3be0 | HEM | B | 450 |
| 3be4 | AP5 | A | 219 |
| 3be9 | P04 | A | 501 |
| 3beg | ANP | A | 656 |
| 3bej | MUF | A | 473 |
| 3bem | PGO | A | 402 |
| 3bem | PGO | B | 301 |
| 3ben | LEH | A | 500 |
| 3beo | UD1 | A | 372 |
| 3beo | UDP | A1081 |  |
| 3bet | CTF | A | 264 |
| 3bex | PAU | A | 248 |
| 3bf1 | ADP | A | 247 |
| 3bf5 | PGO | A | 288 |
| 3bf6 | SVR | H | 301 |
| 3bf8 | MLA | A4892 |  |
| 3bg3 | BTI | A2100 |  |
| 3bg3 | PYR | A2000 |  |
| 3bg5 | ATP | A2100 |  |
| 3bg5 | BTI | A2000 |  |
| 3bg5 | PYR | A2001 |  |
| 3bg8 | INH | A | 612 |
| 3bgp | VX1 | A | 314 |
| 3bgq | VX2 | A | 314 |
| 3bgv | SAH | C | 313 |
| 3bgx | TMP | A | 265 |
| 3bgz | VX3 | A | 314 |
| 3bhd | CIT | A | 219 |
| 3bhe | BZN | B2501 |  |
| 3bhh | 5CP | A | 600 |
| 3bhj | GSH | A | 315 |
| 3bhl | THG | A | 266 |
| 3bhm | AHE | A | 304 |
| 3bhr | THG | A | 266 |
| 3bht | MFR | A | 299 |
| 3bht | MFR | C | 299 |
| 3bhu | MHR | A | 299 |
| 3bhv | VAR | A | 299 |
| 3bhy | 7CP | A | 600 |
| 3bi2 | FAD | A | 801 |
| 3bi4 | FAD | A | 801 |
| 3bi5 | DIA | A2841 |  |
| 3bi5 | FAD | A | 801 |
| 3bib | PSF | X | 118 |
| 3bif | SIN | A | 505 |
| 3biu | 10U | H | 999 |
| 3biv | 11U | H | 999 |
| 3bj8 | SPM | C | 500 |
| 3bjc | WAN | A | 901 |
| 3bje | R1P | A | 401 |
| 3bje | URA | A | 501 |
| 3bjf | FBP | A | 532 |
| 3bjk | CIT | A | 155 |
| 3bju | ATP | A | 603 |
| 3bju | ATP | B | 603 |
| 3bjw | SVR | A | 508 |
| 3bk7 | ADP | A | 598 |
| 3bl1 | BL1 | A | 300 |
| 3bl6 | FMC | A | 229 |
| 3bl7 | DD1 | A | 901 |
| 3bl9 | DD2 | A | 901 |
| 3bla | DD3 | A | 1 |
| 3bla | DD3 | B | 2 |
| 3blb | SWA | A1048 |  |
| 3blk | HMC | A | 504 |
| 3bll | BPQ | A | 700 |
| 3blp | HMC | X | 504 |
| 3blr | CPB | A | 940 |
| 3bm4 | ADV | B | 401 |
| 3bm9 | BXZ | A | 1 |
| 3bmc | FOL | A | 270 |
| 3bmx | P4G | A | 649 |
| 3bmy | CXZ | A | 1 |
| 3bn1 | AKG | A | 374 |
| 3bnm | FAD | A | 801 |
| 3bnu | FAD | A | 801 |
| 3bnx | FPP | A | 400 |
| 3bnz | C16 | A | 318 |
| 3bog | DTH | C | 16 |
| 3bom | HEM | D | 148 |
| 3bp1 | GUN | A | 290 |
| 3bp1 | GUN | B | 290 |
| 3bpr | OLP | A | 900 |
| 3bpt | QUE | A | 501 |
| 3bpw | XMP | A1000 |  |
| 3bpz | CMP | A | 641 |
| 3bq5 | HCS | A | 802 |
| 3bqc | EMO | A | 400 |
| 3bqm | BQM | B | 1 |
| 3bqr | 4RB | A | 401 |
| 3br2 | DEQ | A | 201 |
| 3brb | ADP | A | 901 |
| 3brq | FRU | A | 701 |
| 3bsc | 2PD | A | 579 |
| 3bsf | ADE | A | 701 |
| 3bsh | BGC | A4000 |  |
| 3bsw | CIT | A | 201 |
| 3bti | BER | A | 802 |
| 3bto | SSB | A | 378 |
| 3bug | AEH | A | 394 |
| 3buh | AED | A | 394 |
| 3buj | HEM | A | 398 |
| 3buz | ATP | B | 380 |
| 3bv4 | 13P | A3371 |  |
| 3bv9 | DAR | C | 401 |
| 3bvd | HEM | A | 800 |
| 3bw2 | FMN | A | 370 |
| 3bw3 | FMN | A | 370 |
| 3bw4 | FMN | A | 370 |
| 3bwc | SAM | A | 501 |
| 3bwh | MAN | A | 249 |
| 3bwy | DNC | A | 302 |
| 3bwy | SAM | A | 301 |
| 3bx5 | 304 | A | 501 |
| 3bx9 | CIT | A | 234 |
| 3bxf | 13P | B | 401 |
| 3bxf | FBP | A | 401 |
| 3bxg | BG6 | A | 401 |
| 3bxh | F6P | A | 401 |
| 3bxo | UPP | A | 239 |
| 3bxp | PGO | A | 279 |
| 3bxs | DRS | A | 201 |
| 3bxx | QUE | A | 342 |
| 3by8 | MLT | A | 1 |
| 3by9 | SIN | A | 287 |
| 3byx | C00 | A | 318 |
| 3byz | H11 | A | 601 |
| 3bz1 | DGD | A | 370 |
| 3bz1 | DGD | C | 490 |
| 3bz1 | DGD | C | 491 |
| 3bz7 | ADP | A1000 |  |
| 3bz7 | BL4 | A | 800 |
| 3bz8 | ADP | A1000 |  |
| 3bz8 | BL6 | A | 800 |
| 3bz9 | ADP | A1000 |  |
| 3bz9 | BL7 | A | 800 |
| 3bzm | CIT | A | 432 |
| 3bzu | A21 | A | 293 |
| 3c0a | 14C | A | 318 |
| 3c0o | M6P | A | 501 |
| 3c14 | FOK | A | 101 |
| 3c15 | FOK | A | 101 |
| 3c16 | ATP | A | 1 |
| 3c16 | FOK | A | 101 |
| 3c1m | ANP | A | 472 |
| 3c23 | 3AT | A | 611 |
| 3c27 | DKK | B5000 |  |
| 3c2n | CKR | A | 400 |
| 3c2u | B3P | A | 539 |
| 3c2v | PHT | A | 301 |
| 3c2y | S60 | A | 400 |
| 3c2z | S98 | A1403 |  |
| 3c3n | FMN | D | 316 |
| 3c3y | SAH | A | 464 |
| 3c41 | ANP | J | 601 |
| 3c43 | 315 | A | 1 |
| 3c45 | 317 | A1521 |  |
| 3c49 | KU8 | A | 601 |
| 3c4c | 324 | A | 2 |
| 3c4d | 325 | A | 1 |
| 3c4e | C4E | A | 306 |
| 3c4f | C4F | A | 1 |
| 3c4h | DRL | A | 601 |
| 3c4m | MAL | A | 194 |
| 3c4n | ADP | A | 411 |
| 3c51 | ADP | B | 562 |
| 3c5a | CIT | A | 1 |
| 3c5i | CHT | A1001 |  |
| 3c5n | I3P | A | 2 |
| 3c5u | P41 | A | 361 |
| 3c68 | BMA | A2001 |  |
| 3c6e | NDG | C1396 |  |
| 3c6n | 2S8 | B1001 |  |
| 3c6o | 2S2 | B1001 |  |
| 3c6p | 2S3 | B1001 |  |
| 3c6u | M22 | A | 561 |
| 3c74 | ANU | A1516 |  |
| 3c79 | IM4 | A | 301 |
| 3c7d | PYR | B | 406 |
| 3c7o | CTT | A | 600 |
| 3c7p | MBO | A | 266 |
| 3c7p | POF | A | 265 |
| 3c84 | TH4 | A | 301 |
| 3c8d | CIT | A | 501 |
| 3c8e | GSH | A | 289 |
| 3c8w | CIT | A | 255 |
| 3c8z | 5CA | A | 418 |
| 3c9r | ATP | A | 307 |
| 3c9s | ACP | A | 900 |
| 3c9t | TPS | A | 308 |
| 3c9u | ADP | A | 307 |
| 3ca2 | AMS | A | 265 |
| 3ca3 | A2G | A | 267 |
| 3ca3 | A2G | A | 268 |
| 3ca5 | AMG | A | 267 |
| 3ca5 | AMG | A | 268 |
| 3ca6 | A2G | A | 267 |
| 3cah | FCA | A | 267 |
| 3caj | EZL | A | 265 |
| 3cak | EFS | B | 906 |
| 3cap | BGL | A | 802 |
| 3caq | NDP | A | 327 |
| 3cav | CI2 | A | 328 |
| 3cb5 | B3P | A1000 |  |
| 3cbc | DBS | A | 301 |
| 3cbc | DBS | B | 302 |
| 3cbg | 4FE | A | 502 |
| 3cbp | SFG | A1501 |  |
| 3cbs | R12 | A | 200 |
| 3cbw | CIT | B | 1 |
| 3ccb | B2Y | A | 800 |
| 3ccc | 7AC | A | 800 |
| 3ccn | LKG | A | 1 |
| 3cd8 | L5G | A | 1 |
| 3cdp | YRG | A | 1 |
| 3cds | GRR | A | 1 |
| 3ce0 | P34 | A | 601 |
| 3ce3 | 1FN | A1401 |  |
| 3ceh | AVE | A | 833 |
| 3cej | AVF | A | 833 |
| 3cen | FXA | A | 1 |
| 3cf0 | ADP | A | 900 |
| 3cf1 | ADP | A | 900 |
| 3cf2 | ADP | A | 807 |
| 3cf2 | ANP | A | 901 |
| 3cf3 | ADP | A | 900 |
| 3cf8 | QUE | A | 164 |
| 3cfb | SPB | B | 302 |
| 3cfb | SPB | L | 301 |
| 3cfd | SPB | B | 302 |
| 3cfl | 5CH | A | 693 |
| 3cfn | 2AN | A | 1 |
| 3cfn | 2AN | B | 1 |
| 3cfo | GMP | A1000 |  |
| 3cfq | DIF | B | 1 |
| 3cft | 5NS | A | 1 |
| 3cft | 5NS | B | 128 |
| 3cge | NDP | A | 803 |
| 3cgf | JNF | A | 523 |
| 3cgg | CIT | B | 195 |
| 3cgo | JNO | A | 510 |
| 3ch0 | CIT | A | 272 |
| 3ch6 | 311 | A | 601 |
| 3chb | SIA | D | 108 |
| 3cho | 4BG | A | 901 |
| 3chq | 4BQ | A | 901 |
| 3chr | 4BS | A | 613 |
| 3chw | ATP | A | 402 |
| 3ci5 | ATP | A | 401 |
| 3ci6 | P4G | A | 169 |
| 3cif | G3H | A1337 |  |
| 3cig | NDG | A4131 |  |
| 3cip | ATP | A | 401 |
| 3cis | ATP | A1101 |  |
| 3ciw | SAH | A1501 |  |
| 3cja | ANP | A | 501 |
| 3cjb | ATP | A | 377 |
| 3cjc | ATP | A | 403 |
| 3cjd | STE | A | 201 |
| 3cjf | SAV | A1167 |  |
| 3cjg | KIM | A1167 |  |
| 3cjo | K30 | A | 1 |
| 3cjq | SAH | A | 303 |
| 3cjt | SAH | A | 258 |
| 3cjt | SAM | C | 302 |
| 3ck7 | ACX | B | 688 |
| 3ck8 | BCD | A | 800 |
| 3ckb | MLR | A | 903 |
| 3ckj | CIT | A | 331 |
| 3ckk | SAM | A | 301 |
| 3ckl | STL | A | 501 |
| 3ckq | UPG | A | 332 |
| 3ckv | UDP | A | 331 |
| 3cl9 | MTX | A | 602 |
| 3clb | TMQ | A | 611 |
| 3clp | CMP | A | 1 |
| 3cm4 | MLT | A | 350 |
| 3cmc | G3H | O | 400 |
| 3cmp | DBH | B | 303 |
| 3cmv | ANP | A1400 |  |
| 3cn0 | LJ1 | A | 128 |
| 3cn0 | LJ1 | B | 128 |
| 3cn1 | LJ2 | A | 128 |
| 3cn1 | LJ2 | B | 128 |
| 3cn2 | LJ3 | A | 128 |
| 3cn2 | LJ3 | B | 128 |
| 3cn3 | LJ4 | A | 128 |
| 3cn3 | LJ4 | B | 128 |
| 3cn4 | LJ5 | A | 128 |
| 3cn8 | FAD | A | 801 |
| 3cn8 | SPD | A | 517 |
| 3cnd | FAD | A | 801 |
| 3cne | FMN | B | 201 |
| 3cnm | HHA | A | 500 |
| 3cnp | FAD | A | 517 |
| 3cns | FAD | A | 517 |
| 3cnt | FAD | A | 517 |
| 3co9 | 3MS | A | 579 |
| 3coh | 83H | A | 1 |
| 3cok | ANP | A | 301 |
| 3cp9 | C19 | A | 501 |
| 3cpb | C92 | A | 1 |
| 3cpc | C52 | A | 1 |
| 3cqe | P91 | A | 901 |
| 3cql | NDG | A | 244 |
| 3cqu | CQU | A | 999 |
| 3cqv | HEM | A | 601 |
| 3cqw | CQW | A | 999 |
| 3cqy | SIN | A | 373 |
| 3cr0 | 809 | A | 901 |
| 3cr3 | ADP | A1211 |  |
| 3cr5 | PNT | X | 94 |
| 3cr6 | LSR | A | 138 |
| 3crc | ATP | B | 265 |
| 3crt | GSH | A | 215 |
| 3cru | GSH | A | 215 |
| 3csd | EMO | A | 302 |
| 3csd | NDP | A | 301 |
| 3cse | N22 | A | 229 |
| 3csl | HEM | A | 866 |
| 3csw | CIT | A | 302 |
| 3cth | 319 | A2001 |  |
| 3ctj | 320 | A2001 |  |
| 3ctl | S6P | A | 501 |
| 3cts | CIT | A | 439 |
| 3cty | FAD | A1000 |  |
| 3cu8 | PPI | A | 247 |
| 3cue | PLM | E | 194 |
| 3cuk | FAD | D | 401 |
| 3cv6 | HXS | A | 351 |
| 3cv8 | HEM | A | 413 |
| 3cv9 | HEM | A | 413 |
| 3cwb | PEE | C2007 |  |
| 3cwe | 825 | A | 784 |
| 3cwz | GTP | A4200 |  |
| 3cx5 | HEM | C4001 |  |
| 3cx5 | HEM | C4002 |  |
| 3cx5 | SUC | O4146 |  |
| 3cx5 | UMQ | A4021 |  |
| 3cx8 | GSP | A | 755 |
| 3cx9 | MYR | A1002 |  |
| 3cxh | UMQ | A4021 |  |
| 3cxq | GLP | A | 185 |
| 3cy2 | MB9 | A | 501 |
| 3cy3 | JN5 | A | 501 |
| 3cy5 | HEM | A | 142 |
| 3cy5 | HEM | B | 147 |
| 3cyi | ATP | A | 1 |
| 3cz1 | NBB | A | 122 |
| 3cza | MLA | A | 500 |
| 3czh | BCD | A | 603 |
| 3czj | 149 | A2001 |  |
| 3czm | OXQ | A | 708 |
| 3czr | 3CZ | A | 293 |
| 3czs | MAN | A4499 |  |
| 3czv | AZM | A | 263 |
| 3czy | AD8 | A | 901 |
| 3d04 | SAK | C | 162 |
| 3d0b | SNX | A | 233 |
| 3d0g | NDG | A | 616 |
| 3d0q | MPO | A | 401 |
| 3d0z | GSH | A | 215 |
| 3d14 | AK1 | A | 1 |
| 3d15 | AK2 | A | 1 |
| 3d1a | HEM | A | 142 |
| 3d1a | HEM | B | 147 |
| 3d1c | FAD | A | 500 |
| 3d1g | 322 | A | 501 |
| 3d1r | FBP | A3499 |  |
| 3d27 | W29 | A | 265 |
| 3d28 | B34 | A | 579 |
| 3d2i | AK3 | A | 1 |
| 3d36 | ADP | A | 500 |
| 3d3e | D3E | A | 293 |
| 3d3f | NDP | A | 1 |
| 3d3p | 20A | A | 505 |
| 3d41 | ANP | A1260 |  |
| 3d41 | FCN | A4001 |  |
| 3d42 | TAR | A | 1 |
| 3d45 | 7MG | A | 651 |
| 3d45 | 7MG | B1152 |  |
| 3d47 | MLT | A | 502 |
| 3d4f | MA4 | A | 400 |
| 3d4k | MAN | A | 240 |
| 3d4l | 605 | A1521 |  |
| 3d4n | D4N | A | 293 |
| 3d4o | TAR | A | 295 |
| 3d4p | PYR | A | 319 |
| 3d4s | TIM | A | 401 |
| 3d4x | HEM | B | 148 |
| 3d50 | OEV | A1048 |  |
| 3d51 | GOX | A1048 |  |
| 3d57 | 4HY | A | 500 |
| 3d5f | L41 | A | 501 |
| 3d5w | ADP | A | 313 |
| 3d67 | GEM | A | 660 |
| 3d6d | LRG | A | 1 |
| 3d6v | TFQ | A | 400 |
| 3d7d | FBD | A | 1 |
| 3d7o | HEM | B | 148 |
| 3d7z | GK5 | A | 401 |
| 3d80 | Q22 | A | 188 |
| 3d87 | MAN | D1281 |  |
| 3d8b | ADP | A | 900 |
| 3d8v | UD1 | A | 496 |
| 3d8w | D8W | A | 301 |
| 3d8x | FAD | A1001 |  |
| 3d8y | T3S | A | 127 |
| 3d8z | TXS | A | 125 |
| 3d97 | B3P | A | 138 |
| 3d9c | ZYZ | A | 322 |
| 3d9l | BTN | Y | 0 |
| 3d9v | H52 | A | 416 |
| 3d9z | D9Z | A | 263 |
| 3da1 | FAD | A | 609 |
| 3da2 | 4MD | A | 401 |
| 3da9 | 44U | B | 1 |
| 3daj | FXG | A | 1 |
| 3dak | ANP | A | 1 |
| 3dao | CIT | B | 265 |
| 3dat | MTX | A | 201 |
| 3dau | MTX | A | 201 |
| 3daw | ATP | A | 377 |
| 3dax | HEM | B | 601 |
| 3day | APC | A | 606 |
| 3db6 | FRS | A | 1 |
| 3db8 | 1FR | A | 313 |
| 3dbc | 2FR | A | 313 |
| 3dbk | RDF | A2001 |  |
| 3dbu | D8W | A | 301 |
| 3dbx | PLM | A | 522 |
| 3dc3 | AZM | A | 263 |
| 3dcc | D9Z | A | 263 |
| 3dcj | THH | A | 401 |
| 3dcv | 55E | A | 500 |
| 3dcw | EZL | A | 301 |
| 3dd0 | EZL | A | 301 |
| 3dd1 | CFF | B | 903 |
| 3dd1 | NBG | B | 901 |
| 3dd8 | 2C7 | A | 300 |
| 3ddl | PCW | B1416 |  |
| 3ddp | RC8 | A | 299 |
| 3ddq | SGM | B | 1 |
| 3dds | CFF | A | 904 |
| 3dds | CFF | B | 903 |
| 3dds | NBG | A | 901 |
| 3dds | NBG | B | 901 |
| 3ddu | 552 | A | 901 |
| 3ddw | CFF | B | 903 |
| 3ddw | NBG | B | 901 |
| 3dei | RXB | C | 300 |
| 3dej | RXC | C | 300 |
| 3dek | RXD | A | 300 |
| 3dey | DHT | X | 732 |
| 3df9 | DF9 | A | 233 |
| 3dfk | DKA | A | 400 |
| 3dg8 | RJ6 | A | 609 |
| 3dg8 | UMP | C | 611 |
| 3dga | RJ1 | A | 609 |
| 3dga | UMP | C | 611 |
| 3dge | ADP | A | 500 |
| 3dgl | ATP | A | 81 |
| 3dgm | ADP | A | 81 |
| 3dgn | ADP | A | 81 |
| 3dgo | ATP | A | 82 |
| 3dgq | EAA | A | 211 |
| 3dgr | A12 | A | 501 |
| 3dgv | NDG | C | 309 |
| 3dgy | BGC | A | 99 |
| 3dh0 | SAM | A | 220 |
| 3dh8 | B4N | A1000 |  |
| 3dha | C6L | A | 257 |
| 3dhc | CYK | A | 253 |
| 3dhe | AND | A | 400 |
| 3dhf | NMN | A | 503 |
| 3dhp | HMC | A | 504 |
| 3dhr | HEM | A | 142 |
| 3dhr | HEM | B | 147 |
| 3dht | HEM | A | 142 |
| 3dht | HEM | B | 147 |
| 3dhy | 3DH | A | 500 |
| 3din | ADP | A | 873 |
| 3dj4 | UD1 | A4000 |  |
| 3djf | BC3 | A | 290 |
| 3djg | FAD | X | 479 |
| 3djg | NDP | X | 483 |
| 3djj | FAD | A | 479 |
| 3djj | NDP | A | 480 |
| 3djl | FAD | A | 542 |
| 3djq | UDP | A | 125 |
| 3djv | C3P | A | 125 |
| 3dk4 | FAD | A | 479 |
| 3dk4 | GSH | A | 481 |
| 3dk4 | NDP | A | 480 |
| 3dk8 | FAD | A | 479 |
| 3dk8 | GSH | A | 481 |
| 3dk9 | FAD | A | 479 |
| 3dkc | ATP | A | 1 |
| 3dko | IHZ | A1001 |  |
| 3dkp | ADP | A | 403 |
| 3dkv | AP5 | A | 220 |
| 3dl0 | AP5 | A | 218 |
| 3dl5 | DHF | A | 605 |
| 3dl5 | NDP | A | 606 |
| 3dl6 | DHF | A | 605 |
| 3dl6 | NDP | A | 606 |
| 3dlc | SAM | A | 220 |
| 3dle | GFA | A | 999 |
| 3dls | ADP | A | 1 |
| 3dlt | BUA | A | 245 |
| 3dmf | SAM | A | 388 |
| 3dmg | SAH | A | 388 |
| 3dmh | GMP | A | 385 |
| 3dmh | SAM | A | 384 |
| 3dmk | NDG | B | 787 |
| 3dn5 | 53N | A | 600 |
| 3dnd | LL2 | A | 351 |
| 3dne | LL1 | A | 351 |
| 3dng | AXA | A | 1 |
| 3dng | AXA | B | 2 |
| 3dnt | ATP | A | 500 |
| 3doe | GTP | A | 201 |
| 3dof | GTP | A | 201 |
| 3dog | NNN | A | 299 |
| 3dop | BDT | B | 980 |
| 3dou | SAM | A | 1 |
| 3doy | 2BE | A3001 |  |
| 3doz | 3BE | B | 163 |
| 3dp0 | 2BC | B | 162 |
| 3dp1 | 2RB | B | 162 |
| 3dp2 | 4BE | B | 162 |
| 3dp3 | 4BB | A | 163 |
| 3dp4 | AMQ | A | 374 |
| 3dp9 | BIG | A | 301 |
| 3dpd | 41A | A2040 |  |
| 3dqr | HEM | A | 750 |
| 3dqs | JI3 | A | 800 |
| 3dqt | HEM | B | 700 |
| 3dqt | JI7 | A | 800 |
| 3dqw | SAP | A | 534 |
| 3dr1 | C5D | A | 500 |
| 3dr3 | MLT | A | 337 |
| 3dra | B3P | A | 307 |
| 3dra | GRG | B1721 |  |
| 3drn | CIT | A | 500 |
| 3drp | R8E | A | 601 |
| 3drs | R8D | A | 601 |
| 3ds6 | A17 | A | 361 |
| 3dsb | BET | A | 1 |
| 3dsj | 243 | A | 601 |
| 3dsl | FLE | A | 504 |
| 3dst | GRG | B | 334 |
| 3dsu | FPP | B | 334 |
| 3dsv | GER | B | 334 |
| 3dt0 | 16U | H | 901 |
| 3dt1 | P40 | A | 361 |
| 3dt2 | GTP | A | 900 |
| 3dt4 | GTP | A | 900 |
| 3dt4 | GTP | C | 900 |
| 3dt7 | GTP | A | 800 |
| 3dtb | PGA | A | 900 |
| 3dtc | VIN | A6331 |  |
| 3dtu | DXC | C | 576 |
| 3dtw | A96 | A | 501 |
| 3du4 | KAP | A | 451 |
| 3du7 | CN2 | B | 700 |
| 3du7 | CN2 | D | 701 |
| 3du7 | GTP | D | 601 |
| 3du8 | 553 | A | 553 |
| 3dur | KDO | A | 303 |
| 3dur | KDO | D | 303 |
| 3dus | KDO | A | 107 |
| 3dut | HEM | B | 150 |
| 3dut | HEM | C | 150 |
| 3duu | KDO | B | 114 |
| 3duw | SAH | A | 224 |
| 3dux | 64U | H | 901 |
| 3dv0 | PYR | B | 502 |
| 3dv4 | KDO | B | 114 |
| 3dv6 | KDO | B | 114 |
| 3dvl | ATP | A | 903 |
| 3dwb | 5HD | A | 816 |
| 3dwj | HEM | A | 901 |
| 3dwl | ATP | A | 428 |
| 3dx5 | DHB | A | 289 |
| 3dxh | UDP | A | 125 |
| 3dxj | NE6 | D1529 |  |
| 3dxk | N23 | B | 395 |
| 3dxm | N24 | A | 419 |
| 3dxy | SAM | A | 1 |
| 3dxz | SAH | A | 1 |
| 3dy0 | SGN | A | 504 |
| 3dy6 | DY6 | A | 478 |
| 3dy7 | 1CX | A9002 |  |
| 3dya | PZL | A | 562 |
| 3dye | LNR | A | 600 |
| 3dyn | PCG | A | 903 |
| 3dyo | IPT | A2001 |  |
| 3dyo | IPT | A2002 |  |
| 3dyo | IPT | B2003 |  |
| 3dz6 | M8E | A | 368 |
| 3dzg | NCA | A | 302 |
| 3dzh | GTP | A | 301 |
| 3dzk | NMN | A | 301 |
| 3dzw | MAN | A | 112 |
| 3e01 | PZ2 | A | 562 |
| 3e0b | N22 | A | 208 |
| 3e0i | PEP | A3268 |  |
| 3e0n | DPN | B | 273 |
| 3e0x | OXE | A | 243 |
| 3e1j | HEM | B | 200 |
| 3e1l | HEM | A | 200 |
| 3e1m | HEM | B | 200 |
| 3e1p | HEM | A | 200 |
| 3e1q | HEM | A | 200 |
| 3e1y | ATP | A1526 |  |
| 3e22 | GTP | A | 600 |
| 3e22 | LOC | B | 700 |
| 3e23 | SAM | A | 221 |
| 3e25 | 3PG | A | 340 |
| 3e2c | HEM | A | 203 |
| 3e2n | HEM | A | 296 |
| 3e2o | HEM | A | 296 |
| 3e2q | FAD | A2001 |  |
| 3e2r | CIT | A2502 |  |
| 3e2r | TFB | A2002 |  |
| 3e3b | CCK | X | 351 |
| 3e3c | HHG | A | 150 |
| 3e3u | NVC | A | 201 |
| 3e4f | CIT | A | 266 |
| 3e4n | HEM | A | 156 |
| 3e4p | MLA | A | 701 |
| 3e4w | HEM | A | 501 |
| 3e4y | HEM | A | 501 |
| 3e55 | HEM | A | 156 |
| 3e5i | HEM | A | 154 |
| 3e5j | HEM | A1408 |  |
| 3e5k | HEM | A1408 |  |
| 3e5l | HEM | A1408 |  |
| 3e5o | HEM | A | 156 |
| 3e5p | PPI | A | 400 |
| 3e63 | 5B2 | A | 1 |
| 3e64 | 5B3 | A | 1 |
| 3e65 | XXZ | A | 906 |
| 3e68 | AT6 | A | 905 |
| 3e6j | DR2 | A | 300 |
| 3e6l | A11 | A | 906 |
| 3e6o | A55 | A | 903 |
| 3e6t | 1A2 | A | 905 |
| 3e73 | GSH | A | 502 |
| 3e7a | MDH | C | 5 |
| 3e7e | ATP | A1501 |  |
| 3e7f | 6PG | A | 843 |
| 3e7m | AT2 | A | 906 |
| 3e7o | 35F | A | 1 |
| 3e7s | AT2 | B | 906 |
| 3e7s | HEC | B | 902 |
| 3e7t | B14 | A | 906 |
| 3e7v | DZO | A | 1 |
| 3e81 | SLB | A | 171 |
| 3e85 | BSU | A | 158 |
| 3e87 | G95 | A | 1 |
| 3e8r | CIT | B | 3 |
| 3e92 | G6A | A | 361 |
| 3e9k | 3XH | A | 467 |
| 3e9s | TTT | A | 317 |
| 3ea2 | INS | A | 801 |
| 3ea4 | FAB | A | 696 |
| 3eah | 327 | A | 864 |
| 3eah | HEC | A | 861 |
| 3eai | 328 | A | 905 |
| 3eb0 | DRK | A | 384 |
| 3eb1 | LZQ | A | 322 |
| 3ebd | 329 | A | 903 |
| 3ebf | 332 | A | 903 |
| 3ebh | BES | A1085 |  |
| 3ebi | BEY | A1085 |  |
| 3ec6 | FAD | A | 139 |
| 3ecl | HEM | A | 154 |
| 3ecn | IBM | A | 3 |
| 3ecr | DPM | A | 365 |
| 3ecx | HEM | A | 154 |
| 3ecz | HEM | A | 154 |
| 3ed0 | EMO | A | 163 |
| 3ed9 | HEM | A | 154 |
| 3eda | HEM | A | 154 |
| 3edb | HEM | A | 154 |
| 3edj | BCD | A | 700 |
| 3edn | SIN | A | 308 |
| 3edo | FMN | A | 200 |
| 3edz | 550 | B | 1 |
| 3edz | CIT | A | 4 |
| 3ee2 | NZO | A | 201 |
| 3ee4 | MYR | A | 315 |
| 3eej | 53R | A | 229 |
| 3eek | 53S | A | 229 |
| 3eel | 53T | A | 229 |
| 3eem | 53V | A | 229 |
| 3eey | SAM | A | 300 |
| 3ef5 | DGT | A3800 |  |
| 3efr | BTN | A1001 |  |
| 3efs | BTN | A2001 |  |
| 3efx | A2G | D | 201 |
| 3egi | ADP | A | 3 |
| 3egk | M18 | H | 601 |
| 3egv | SAH | A | 256 |
| 3ehf | ACP | A | 500 |
| 3ehg | ATP | A | 1 |
| 3ehh | ADP | A2500 |  |
| 3ehj | ACP | A1303 |  |
| 3ehn | NLC | A | 600 |
| 3ehs | MAL | A | 126 |
| 3eht | MAL | A | 126 |
| 3ehu | MAL | A | 503 |
| 3ehx | BDL | A | 0 |
| 3ei8 | PLP | A | 433 |
| 3eib | PLP | A | 433 |
| 3eic | UDP | A | 161 |
| 3eif | MLA | A1036 |  |
| 3eiu | AES | B | 461 |
| 3ej0 | 11X | A | 176 |
| 3ej1 | 5BP | A | 299 |
| 3ej2 | 928 | A | 176 |
| 3ej8 | HEC | A1901 |  |
| 3ejb | HEM | B | 405 |
| 3ejb | HTG | A | 100 |
| 3ejb | HTG | D | 406 |
| 3ejd | HEM | B | 405 |
| 3ejd | HTG | D | 417 |
| 3eje | HEM | B | 405 |
| 3eje | HTG | B | 417 |
| 3ejk | CIT | A | 176 |
| 3ekl | 13P | A | 350 |
| 3eko | PYU | A | 901 |
| 3ekr | PY9 | A | 901 |
| 3eks | ATP | A | 800 |
| 3eku | ATP | A | 800 |
| 3ekz | G3P | A | 352 |
| 3el2 | ATP | A | 800 |
| 3eld | SFG | A | 293 |
| 3ele | PLP | A | 500 |
| 3elf | 2FP | A | 350 |
| 3elg | CIT | B | 1 |
| 3elh | DUD | A | 160 |
| 3elj | GS7 | A | 365 |
| 3elw | GP3 | A4634 |  |
| 3elz | CHD | A | 150 |
| 3em0 | CHD | A | 150 |
| 3emb | GTG | A4634 |  |
| 3emg | 685 | A | 685 |
| 3eml | STE | A | 402 |
| 3eml | ZMA | A | 401 |
| 3emm | HEM | A | 200 |
| 3emt | DGI | A | 201 |
| 3en1 | MBN | A | 457 |
| 3en4 | KS1 | A | 1 |
| 3en5 | KS4 | A | 1 |
| 3en6 | KS5 | B | 2 |
| 3en7 | ABJ | A | 1 |
| 3ena | DGI | B | 201 |
| 3ene | NPZ | A | 1 |
| 3eng | CBI | A | 214 |
| 3eoj | BCL | A | 371 |
| 3eok | HEM | A | 142 |
| 3eok | HEM | B | 147 |
| 3ep6 | PYR | A | 68 |
| 3epm | HMH | A1001 |  |
| 3epp | SFG | A | 401 |
| 3eps | ATP | A1605 |  |
| 3epy | PLM | A | 101 |
| 3epz | BGC | A | 702 |
| 3eq7 | X99 | A | 711 |
| 3eq8 | X98 | A | 711 |
| 3eqa | MAN | A | 602 |
| 3eqc | 3BM | A | 1 |
| 3eqg | 4BM | A | 1 |
| 3eql | MXP | D1527 |  |
| 3eqm | ASD | A | 601 |
| 3eqm | HEM | A | 600 |
| 3eqo | LGC | A | 757 |
| 3erd | DES | A | 600 |
| 3erg | GTS | A | 234 |
| 3es9 | FAD | B | 752 |
| 3esn | DZ1 | A | 128 |
| 3esn | DZ1 | B | 128 |
| 3eso | DZ2 | A | 128 |
| 3eso | DZ2 | B | 128 |
| 3esp | DZ3 | A | 128 |
| 3esp | DZ3 | B | 128 |
| 3et2 | 1BO | A | 3 |
| 3etl | ANP | A | 401 |
| 3etn | CMK | A | 500 |
| 3etq | CMP | A | 1 |
| 3ets | 4MU | A | 572 |
| 3ett | NPO | A | 572 |
| 3eu1 | HEM | B | 147 |
| 3eu1 | HEM | C | 142 |
| 3eub | XAN | 47319 |  |
| 3euz | DMF | A | 905 |
| 3evd | GTP | A | 901 |
| 3evf | GTA | A | 902 |
| 3evf | SAH | A | 901 |
| 3evl | LGU | A | 402 |
| 3evo | TYD | A | 160 |
| 3evo | TYD | B | 161 |
| 3ew2 | BTN | A | 400 |
| 3ew8 | B3N | A | 501 |
| 3ew9 | ANP | A | 401 |
| 3ewa | ANP | A | 401 |
| 3ewj | 642 | B | 1 |
| 3ewk | FAD | A | 326 |
| 3ews | ADP | A | 602 |
| 3ewx | BMP | A | 481 |
| 3ex4 | BMP | A | 481 |
| 3exb | HEM | A | 300 |
| 3exm | GP2 | A | 401 |
| 3exo | 5MS | A | 453 |
| 3ey4 | 352 | A | 601 |
| 3ey9 | FAD | A | 612 |
| 3ey9 | TDP | A | 611 |
| 3eya | TDP | A | 611 |
| 3eyg | MI1 | A | 1 |
| 3eyh | IZA | A2001 |  |
| 3eyk | EYK | B | 173 |
| 3eym | EYK | F | 173 |
| 3eyv | NDG | H | 225 |
| 3eyw | FMN | A2400 |  |
| 3f0b | 53R | X | 158 |
| 3f0l | BLA | A | 249 |
| 3f0m | BLA | A | 249 |
| 3f0s | 53T | X | 158 |
| 3f0u | 53R | X | 158 |
| 3f0x | 53T | X | 158 |
| 3f10 | 8HG | A | 293 |
| 3f17 | HS4 | A | 0 |
| 3f19 | HS6 | A | 0 |
| 3f1a | HS7 | A | 0 |
| 3f1q | BCE | A | 397 |
| 3f2e | CIT | A | 200 |
| 3f2n | IZZ | A | 800 |
| 3f33 | PFL | A2001 |  |
| 3f3a | C14 | A | 707 |
| 3f3m | PPS | A | 300 |
| 3f3t | 1AU | A | 1 |
| 3f3u | 1AW | A | 1 |
| 3f3z | DRK | A | 1 |
| 3f46 | DTV | A | 363 |
| 3f4x | KLT | A | 300 |
| 3f53 | SIA | A | 401 |
| 3f5a | SIA | A | 401 |
| 3f5e | SIA | A | 401 |
| 3f5f | A3P | A | 673 |
| 3f5j | CTT | A1000 |  |
| 3f5m | ATP | A1001 |  |
| 3f5o | UOC | A | 149 |
| 3f61 | ADP | A | 340 |
| 3f66 | IHX | A | 1 |
| 3f6d | GTX | A | 220 |
| 3f6r | FMN | A | 149 |
| 3f6s | FMN | A | 149 |
| 3f6x | IHH | A | 1 |
| 3f78 | ICF | A | 1 |
| 3f7c | CIT | A | 200 |
| 3f7z | 34O | A3000 |  |
| 3f88 | 3HT | A | 999 |
| 3f8d | FAD | A2001 |  |
| 3f8s | PF2 | A | 900 |
| 3f90 | FMN | A | 149 |
| 3f9m | MRK | A | 501 |
| 3f9w | SAH | A | 801 |
| 3f9x | SAH | A | 801 |
| 3f9y | SAH | A | 801 |
| 3f9z | SAH | A | 801 |
| 3faa | 55F | A | 601 |
| 3fah | PCD | A | 921 |
| 3fat | AMQ | A | 427 |
| 3fax | BGC | A | 1 |
| 3fb4 | AP5 | A | 0 |
| 3fbr | ACP | A | 500 |
| 3fbs | FAD | A | 298 |
| 3fbv | APJ | A1999 |  |
| 3fc4 | PCD | A | 921 |
| 3fc5 | HEM | A | 750 |
| 3fc8 | IFA | A3000 |  |
| 3fc8 | IFA | B2000 |  |
| 3fc9 | CTP | A | 142 |
| 3fcb | IFB | A3000 |  |
| 3fcf | FCF | A | 1 |
| 3fcr | PLP | A | 458 |
| 3fcv | DUD | B | 201 |
| 3fd6 | ADP | A | 393 |
| 3fec | MPO | A | 741 |
| 3fee | QUS | A | 741 |
| 3feg | ADP | A | 397 |
| 3ffk | ATP | B | 380 |
| 3ffp | LC1 | X | 300 |
| 3ffu | GTP | A4033 |  |
| 3fg4 | ACD | A2001 |  |
| 3fgc | FMN | A3402 |  |
| 3fgo | CZA | A1005 |  |
| 3fgu | ANP | A | 600 |
| 3fh0 | ADP | A | 201 |
| 3fh9 | HEM | A | 142 |
| 3fh9 | HEM | B | 147 |
| 3fhq | BMA | A | 622 |
| 3fhq | MAN | A | 623 |
| 3fhq | NGT | A | 625 |
| 3fhr | P4O | A | 1 |
| 3fie | DCY | C | 58 |
| 3fiz | BGC | A1970 |  |
| 3fj0 | BGC | A1970 |  |
| 3fj6 | CIH | A | 399 |
| 3fj7 | PEQ | A | 301 |
| 3fjg | 3PG | A | 301 |
| 3fjl | CJH | A | 399 |
| 3fjo | FAD | A | 750 |
| 3fjo | FMN | A | 751 |
| 3fl5 | TXQ | A | 338 |
| 3fmd | IQB | A | 1 |
| 3fmi | KAP | A | 430 |
| 3fmz | 2T1 | A | 184 |
| 3fne | 8PC | A | 400 |
| 3fnq | HPA | A | 981 |
| 3fpb | ATP | A1002 |  |
| 3fpb | CZA | A1001 |  |
| 3fpd | SAH | A | 101 |
| 3fpf | MTA | A | 301 |
| 3fpg | B3P | B | 300 |
| 3fpj | B3P | B | 302 |
| 3fpj | SAM | A | 301 |
| 3fpm | 793 | A | 1 |
| 3fps | ADP | A1002 |  |
| 3fps | CZA | A1001 |  |
| 3fpt | NDG | C | 119 |
| 3fq0 | N22 | A | 219 |
| 3fq8 | PMP | A5000 |  |
| 3fqa | PMP | A1434 |  |
| 3fqc | 55V | A | 219 |
| 3fqe | P5C | A | 1 |
| 3fqf | 55V | A | 219 |
| 3fqh | 057 | A | 1 |
| 3fql | 79Z | A | 571 |
| 3fqo | N22 | A | 219 |
| 3fqs | 585 | A | 1 |
| 3fqv | 11F | A | 219 |
| 3fqz | 11F | A | 219 |
| 3fr0 | AJB | A | 501 |
| 3fr2 | 8CA | A | 501 |
| 3fr5 | I4A | A | 132 |
| 3fr8 | NDP | A | 601 |
| 3fra | I2H | X | 300 |
| 3frd | DHF | X | 300 |
| 3frf | XCF | X | 300 |
| 3frh | SAH | A | 260 |
| 3frj | A49 | B | 2 |
| 3frq | ERY | A | 195 |
| 3fry | CIT | A2324 |  |
| 3fs4 | HEM | B | 150 |
| 3fs4 | HEM | C | 150 |
| 3fss | MLA | A | 302 |
| 3fst | FAD | A | 395 |
| 3fst | MRY | C5321 |  |
| 3fsu | C2F | A | 995 |
| 3fsy | SCA | A | 331 |
| 3fts | STL | A | 710 |
| 3ftu | RE2 | A | 710 |
| 3ftv | 11X | A | 710 |
| 3fty | 3IP | A | 710 |
| 3fu0 | 22F | A | 710 |
| 3fu3 | 92G | A | 710 |
| 3fu3 | 92G | A | 720 |
| 3fu5 | 492 | A | 710 |
| 3fu6 | 80G | A | 710 |
| 3fuc | 9DG | A | 401 |
| 3fud | 692 | A | 710 |
| 3fuh | BES | A | 720 |
| 3fui | 812 | A | 710 |
| 3fuj | 00G | A | 710 |
| 3fum | 80A | A | 710 |
| 3fun | 798 | A | 710 |
| 3fup | MI1 | A | 1 |
| 3fus | MAN | A | 35 |
| 3fuw | MTA | A | 272 |
| 3fv8 | JK3 | A | 500 |
| 3fvb | HEM | A | 162 |
| 3fvq | ATP | A | 401 |
| 3fvu | IAC | A | 423 |
| 3fw1 | FAD | A | 232 |
| 3fw1 | STI | A | 233 |
| 3fw5 | MCT | A | 180 |
| 3fwk | BGC | A | 306 |
| 3fwn | 6PG | A2002 |  |
| 3fwn | ATR | B2000 |  |
| 3fwp | ANU | B7016 |  |
| 3fws | ANP | A | 221 |
| 3fx6 | BPX | A | 311 |
| 3fxi | FTT | A1003 |  |
| 3fxi | FTT | A1004 |  |
| 3fxi | KDO | A1012 |  |
| 3fxi | MYR | A1008 |  |
| 3fxp | GLO | A | 318 |
| 3fxu | TSU | A1001 |  |
| 3fxu | TSU | A1008 |  |
| 3fxw | F10 | A | 1 |
| 3fxx | ANP | A | 992 |
| 3fy1 | AMI | A | 803 |
| 3fy8 | I2H | X | 300 |
| 3fy9 | XCF | X | 300 |
| 3fyh | ADP | A | 401 |
| 3fyj | B97 | X | 372 |
| 3fyk | B98 | X | 372 |
| 3fyv | XCF | X | 300 |
| 3fyw | I2H | X | 300 |
| 3fz1 | B98 | A | 299 |
| 3fz6 | PMP | A | 500 |
| 3fz9 | GSH | A | 1 |
| 3fzg | SAM | A | 300 |
| 3fzk | 3BK | A | 401 |
| 3fzs | B96 | A | 999 |
| 3g07 | SAM | A | 701 |
| 3g08 | PLM | A | 287 |
| 3g0d | XIH | A | 800 |
| 3g0e | B49 | A9000 |  |
| 3g0f | B49 | A9001 |  |
| 3g0i | VPR | A | 1 |
| 3g0u | MDY | A | 2 |
| 3g0w | LGB | A | 1 |
| 3g0x | MD7 | A | 2 |
| 3g0y | P9A | A | 413 |
| 3g1a | UP6 | A | 229 |
| 3g1l | RF2 | A | 237 |
| 3g1m | RF3 | A | 217 |
| 3g1o | RF1 | A | 237 |
| 3g1p | MLT | A | 500 |
| 3g1q | HEM | A | 480 |
| 3g1r | FIT | B | 327 |
| 3g26 | MLA | A4892 |  |
| 3g2q | SFG | A | 400 |
| 3g2x | TYD | A | 201 |
| 3g2y | GF4 | A | 1 |
| 3g2z | GZ2 | A | 1 |
| 3g30 | G30 | A | 1 |
| 3g31 | GF1 | A | 1 |
| 3g32 | 3G3 | A | 1 |
| 3g34 | 1CE | A | 1 |
| 3g35 | F13 | A | 1 |
| 3g36 | DTV | C | 1 |
| 3g3e | FAD | A | 352 |
| 3g3e | FAD | B | 352 |
| 3g3n | TC8 | A | 3 |
| 3g3r | ANP | A1000 |  |
| 3g3w | MTN | A | 801 |
| 3g42 | 792 | A | 1 |
| 3g49 | 3G4 | A2004 |  |
| 3g49 | 3G4 | D2007 |  |
| 3g4k | ROL | A | 901 |
| 3g4r | DCE | A | 150 |
| 3g59 | ATP | A | 305 |
| 3g5a | APC | A | 305 |
| 3g5a | FMN | A | 306 |
| 3g5e | NDP | A | 318 |
| 3g5e | Q74 | A | 320 |
| 3g5h | YTT | A | 397 |
| 3g5k | BB2 | A1001 |  |
| 3g5m | XM5 | A | 232 |
| 3g5q | FAD | A | 444 |
| 3g5r | FAD | A | 444 |
| 3g5r | THG | A | 445 |
| 3g5s | FAD | A | 444 |
| 3g5t | SAH | A | 308 |
| 3g68 | CIT | A | 401 |
| 3g6k | FAD | A | 306 |
| 3g6k | FAD | B | 306 |
| 3g6m | CFF | A | 1 |
| 3g6m | CFF | A | 427 |
| 3g81 | MMA | A | 500 |
| 3g82 | FOK | A | 101 |
| 3g83 | MAN | A | 500 |
| 3g84 | MAN | A | 500 |
| 3g89 | SAM | A | 303 |
| 3g8a | SAH | A | 303 |
| 3g8c | ADP | A1000 |  |
| 3g8c | BTN | A1004 |  |
| 3g8d | ADP | B1001 |  |
| 3g8f | PHQ | B | 134 |
| 3g8i | RO7 | A | 1 |
| 3g90 | J72 | X | 1 |
| 3g9e | RO7 | A | 1 |
| 3g9l | J67 | X | 1 |
| 3g9n | J88 | A | 359 |
| 3gah | ATP | A | 999 |
| 3gal | 1GN | A | 998 |
| 3gam | FAD | A | 232 |
| 3gam | MXX | A | 231 |
| 3gan | SVR | A | 158 |
| 3gas | HEM | A1290 |  |
| 3gbg | PAM | A5975 |  |
| 3gc2 | SIN | A | 400 |
| 3gck | BHO | A | 800 |
| 3gcz | SAM | A4633 |  |
| 3gd9 | BGC | A | 2 |
| 3gdh | PGO | C | 856 |
| 3gdh | SAH | A | 854 |
| 3gdj | HEM | A | 142 |
| 3gdj | HEM | B | 147 |
| 3gdn | MAN | B | 531 |
| 3gdp | NDG | B | 533 |
| 3ge1 | ADP | B | 499 |
| 3gee | FON | A | 475 |
| 3geh | FON | A | 461 |
| 3geq | PP2 | A | 1 |
| 3gey | P34 | A | 701 |
| 3gf2 | SAL | A | 147 |
| 3gf4 | UPG | A | 392 |
| 3gfe | P37 | A | 361 |
| 3gft | CIT | F | 301 |
| 3gfz | FMN | A | 504 |
| 3gg2 | UGA | A1000 |  |
| 3ggd | SAH | A | 248 |
| 3ggf | GVD | A | 305 |
| 3ggo | ENO | B3168 |  |
| 3ggp | HPP | B | 313 |
| 3ghf | CIT | A | 201 |
| 3ghm | BGC | A | 906 |
| 3ghn | BGC | A | 906 |
| 3ghn | NDG | A | 900 |
| 3ghq | HEM | A | 200 |
| 3ghr | CIT | A | 450 |
| 3ghr | LDT | A | 320 |
| 3ghr | NDP | A | 318 |
| 3ghs | CIT | A | 450 |
| 3ghs | LDT | A | 320 |
| 3ghs | NDP | A | 318 |
| 3ght | CIT | A | 450 |
| 3ght | LDT | A | 320 |
| 3ght | NDP | A | 318 |
| 3ghu | CIT | A | 450 |
| 3ghu | LDT | A | 320 |
| 3ghu | NDP | A | 318 |
| 3gia | D10 | A | 800 |
| 3gid | S1A | A1000 |  |
| 3gie | ACP | A1303 |  |
| 3gif | ADP | A1301 |  |
| 3gig | ACP | B1303 |  |
| 3gjr | DZE | A | 1 |
| 3gju | PLP | A | 460 |
| 3gk1 | 32A | A | 94 |
| 3gk2 | 27A | A | 94 |
| 3gk7 | SPD | A | 501 |
| 3gkj | HC3 | A | 253 |
| 3gkn | BIH | A | 527 |
| 3gkr | UDP | A | 339 |
| 3glc | R5P | A | 400 |
| 3glq | RAB | A | 602 |
| 3glz | 1BD | A | 500 |
| 3gm5 | CIT | A | 379 |
| 3gml | PLM | A | 301 |
| 3gmm | PLM | A | 301 |
| 3gmn | PLM | A | 301 |
| 3gmo | PLM | A | 301 |
| 3gmp | PLM | A | 301 |
| 3gmq | PLM | A | 300 |
| 3gmr | MAN | A | 296 |
| 3gmr | PLM | A | 299 |
| 3gmv | TAM | X | 1 |
| 3gn0 | DMO | A | 551 |
| 3gn9 | MLT | A | 1 |
| 3gnd | 5RP | A | 984 |
| 3gni | ATP | B | 1 |
| 3gni | CIT | A | 342 |
| 3gnp | SOG | A1001 |  |
| 3go4 | SAH | A | 277 |
| 3goi | LOI | A | 1 |
| 3gol | XND | A | 579 |
| 3gon | ANP | A | 500 |
| 3gou | HEM | B | 147 |
| 3gp4 | MED | B | 513 |
| 3gp6 | SDS | A | 163 |
| 3gqe | BCN | A | 167 |
| 3gqg | HEM | B | 400 |
| 3gqi | ACP | A | 775 |
| 3gqk | ATP | A | 1 |
| 3gql | GQL | A | 1 |
| 3gqo | APR | A | 167 |
| 3gqp | HEM | B | 148 |
| 3gqr | HEM | B | 148 |
| 3gqt | UFO | A1000 |  |
| 3gqy | DZG | B | 540 |
| 3gqy | FBP | A | 541 |
| 3gqz | GF7 | A | 362 |
| 3gr2 | GF4 | A | 1 |
| 3gr3 | FMN | A | 230 |
| 3gr4 | ADP | A | 538 |
| 3gr4 | DYY | A | 550 |
| 3gr4 | FBP | A | 541 |
| 3gr6 | TCL | A | 371 |
| 3gr8 | FMN | B | 341 |
| 3grr | SAH | A | 300 |
| 3gry | SAM | A | 300 |
| 3gs0 | 6BD | A | 128 |
| 3gs4 | 7BD | A | 501 |
| 3gs4 | 7BD | B | 500 |
| 3gs6 | NP6 | A | 341 |
| 3gs7 | 8BD | A | 128 |
| 3gsg | GF1 | A | 366 |
| 3gsi | FAD | A | 902 |
| 3gsi | THG | A2887 |  |
| 3gsm | VPU | A | 341 |
| 3gt8 | ANP | A | 2 |
| 3gtc | GTC | A | 362 |
| 3gu3 | SAH | A | 401 |
| 3guc | ANP | A | 402 |
| 3gui | TAM | A | 167 |
| 3guk | MBN | A | 501 |
| 3gum | PXY | A | 501 |
| 3gun | ANL | A | 168 |
| 3gvb | 3GV | A | 1 |
| 3gve | CIT | A | 502 |
| 3gvi | ADP | A | 401 |
| 3gvj | SIA | A2002 |  |
| 3gvk | SIA | A | 912 |
| 3gvk | SLB | A | 1 |
| 3gvk | SLB | A | 911 |
| 3gvl | SIA | A | 1 |
| 3gvl | SLB | A | 2 |
| 3gvo | DTD | A1060 |  |
| 3gvu | STI | A1001 |  |
| 3gw6 | TAM | A | 32 |
| 3gw9 | HEM | A | 480 |
| 3gwc | UFP | A | 260 |
| 3gwj | MAN | A | 804 |
| 3gwl | FAD | B | 335 |
| 3gwv | RFX | A | 801 |
| 3gww | SFX | A | 801 |
| 3gwx | EPA | A | 1 |
| 3gxf | IFM | B | 517 |
| 3gxl | QIG | A | 999 |
| 3gxp | TAM | A6744 |  |
| 3gxw | SIN | A1003 |  |
| 3gxw | SIN | C1004 |  |
| 3gy3 | PNT | A | 246 |
| 3gyd | CMP | A | 300 |
| 3gyi | FAD | A | 510 |
| 3gyj | FAD | A | 510 |
| 3gys | HEM | B | 148 |
| 3gyx | FAD | A1000 |  |
| 3gz7 | CIT | A | 97 |
| 3gz8 | APR | A | 302 |
| 3gz9 | B7G | A | 570 |
| 3gzm | PNS | A | 100 |
| 3h09 | MLA | A2000 |  |
| 3h09 | MLA | A2200 |  |
| 3h0b | B35 | A | 449 |
| 3h0c | PS4 | A | 1 |
| 3h0l | ADP | B | 701 |
| 3h0r | ADP | B | 479 |
| 3h0r | ATP | E | 479 |
| 3h18 | PMS | A | 311 |
| 3h1h | HEM | C | 502 |
| 3h1h | PEE | C2008 |  |
| 3h1i | HEM | C | 501 |
| 3h1i | PEE | A2008 |  |
| 3h1j | HEM | C | 502 |
| 3h1j | PEE | C2008 |  |
| 3h1j | PLC | E2009 |  |
| 3h1k | HEM | C | 501 |
| 3h1k | PEE | R3005 |  |
| 3h1l | HEM | C | 502 |
| 3h1l | PEE | A2008 |  |
| 3h1l | PEE | P3008 |  |
| 3h1q | ATP | A | 301 |
| 3h1y | F6P | A | 392 |
| 3h2b | SAH | A | 301 |
| 3h2w | BGC | A | 601 |
| 3h2y | DGI | A | 369 |
| 3h30 | RFZ | A | 336 |
| 3h39 | ATP | A | 501 |
| 3h39 | ATP | B | 502 |
| 3h3a | CTP | A | 501 |
| 3h3a | CTP | B | 502 |
| 3h3c | P1E | A | 999 |
| 3h3g | MAL | A | 194 |
| 3h41 | DGL | A | 502 |
| 3h4i | U2F | A | 547 |
| 3h4l | ANP | A | 397 |
| 3h4m | ADP | A | 439 |
| 3h4s | ADP | A | 600 |
| 3h4t | UDP | A | 604 |
| 3h4v | DVP | A | 301 |
| 3h4y | CIT | A | 156 |
| 3h52 | 486 | A | 3 |
| 3h55 | CIT | A1100 |  |
| 3h57 | HEM | A | 154 |
| 3h58 | HEM | A | 154 |
| 3h5n | ATP | A | 502 |
| 3h5q | THM | A | 434 |
| 3h65 | H4M | A | 364 |
| 3h6f | DMF | 2 | 42 |
| 3h6i | DMF | 2 | 58 |
| 3h6l | SAM | A | 1 |
| 3h6o | D8G | B | 550 |
| 3h6o | FBP | A | 541 |
| 3h6t | CYZ | A | 265 |
| 3h6u | NS3 | A | 265 |
| 3h6v | NS6 | A | 265 |
| 3h6w | NS7 | A | 265 |
| 3h6z | SUC | A | 1 |
| 3h7j | PPY | A | 246 |
| 3h7q | BCN | A | 131 |
| 3h80 | ANP | A | 223 |
| 3h83 | SUC | A | 181 |
| 3h8g | BES | A | 505 |
| 3h8t | HEM | A | 301 |
| 3h99 | CIT | A | 603 |
| 3h9j | APC | A | 359 |
| 3h9o | 9BD | A | 1 |
| 3ha5 | SFG | A | 302 |
| 3ha7 | B32 | A | 302 |
| 3hab | 677 | A | 767 |
| 3hab | NDG | B2092 |  |
| 3hac | 361 | A | 767 |
| 3hac | NDG | B2092 |  |
| 3hap | D10 | A | 250 |
| 3hap | D12 | A | 251 |
| 3haq | D12 | A | 404 |
| 3haz | FAD | A2001 |  |
| 3hb6 | HEM | A | 485 |
| 3hb9 | ADP | A2100 |  |
| 3hb9 | BTI | A2000 |  |
| 3hbb | TMQ | A | 611 |
| 3hbf | MYC | A | 901 |
| 3hbl | ADP | C2100 |  |
| 3hbl | BTI | A2000 |  |
| 3hbn | UDP | A | 301 |
| 3hbt | ATP | A1380 |  |
| 3hc9 | HEM | A | 154 |
| 3hcc | LT3 | A2001 |  |
| 3hcf | LT5 | A1001 |  |
| 3hch | CIT | A1003 |  |
| 3hcn | CHD | A | 3 |
| 3hcn | CHD | B | 1 |
| 3hcn | HEM | A | 424 |
| 3hcn | HEM | B | 926 |
| 3hco | CHD | B | 1 |
| 3hco | HEM | B | 926 |
| 3hcp | CHD | B | 1 |
| 3hd2 | APC | A | 171 |
| 3hdm | MMG | A | 500 |
| 3hdn | GMG | A | 1 |
| 3he3 | UDP | A | 400 |
| 3hee | R5P | A | 651 |
| 3hen | HEM | A | 154 |
| 3heo | HEM | A | 154 |
| 3hep | HEM | A | 154 |
| 3hez | TAM | B | 34 |
| 3hf4 | HEM | A | 142 |
| 3hf4 | HEM | B | 147 |
| 3hfa | DMF | 2 | 99 |
| 3hfr | 6JZ | A | 269 |
| 3hfw | ADP | A | 358 |
| 3hfx | 152 | A | 601 |
| 3hgg | CHD | A | 211 |
| 3hgm | ATP | B | 148 |
| 3hgs | PHB | A | 402 |
| 3hgy | TCH | A | 211 |
| 3hh2 | CIT | C | 290 |
| 3hh2 | CIT | C | 291 |
| 3hhm | KWT | A1833 |  |
| 3hhw | TAR | K | 423 |
| 3hii | PNT | A | 901 |
| 3hiy | UTP | A | 501 |
| 3hj1 | UTP | A | 501 |
| 3hj3 | MTX | A | 605 |
| 3hj3 | NDP | A | 606 |
| 3hjn | ADP | A | 301 |
| 3hjn | TYD | A | 401 |
| 3hjo | EAA | A | 211 |
| 3hjq | MBD | A | 283 |
| 3hjv | CXS | A | 324 |
| 3hjv | CXS | A | 325 |
| 3hkb | GTP | A | 600 |
| 3hkc | GTP | A | 600 |
| 3hkd | GTP | A | 600 |
| 3hke | GTP | C | 600 |
| 3hkk | GTS | A | 203 |
| 3hkk | PAM | A1151 |  |
| 3hko | ANP | A | 329 |
| 3hl3 | SUC | A | 246 |
| 3hl7 | I47 | A | 401 |
| 3hlf | SIM | A | 500 |
| 3hlg | LVA | A | 500 |
| 3hll | I45 | A | 401 |
| 3hlx | PQQ | A | 500 |
| 3hm8 | BG6 | A1002 |  |
| 3hmi | DKI | A | 1 |
| 3hmj | FMN | G3051 |  |
| 3hmm | 855 | A | 857 |
| 3hmn | ATP | A | 1 |
| 3hmy | SIA | A | 5 |
| 3hn1 | SIA | A | 5 |
| 3hn3 | NDG | A | 650 |
| 3hna | SAH | A | 101 |
| 3hne | ATP | B | 807 |
| 3hnf | DTP | B | 805 |
| 3hng | 8ST | A2001 |  |
| 3ho8 | BTI | B2000 |  |
| 3hof | DHC | C | 123 |
| 3hok | HEM | A | 300 |
| 3hp1 | ADP | A | 301 |
| 3hp5 | 52P | A | 401 |
| 3hp8 | SUC | A | 108 |
| 3hpi | SUC | A | 371 |
| 3hpq | AP5 | A | 215 |
| 3hpr | AP5 | A | 215 |
| 3hpy | MCT | A | 311 |
| 3hq9 | HEM | A | 401 |
| 3hqo | ATP | A1001 |  |
| 3hqp | ATP | A1001 |  |
| 3hqu | UN9 | A | 500 |
| 3hqw | PF4 | A | 999 |
| 3hqy | PF6 | A | 999 |
| 3hqz | PF8 | A | 999 |
| 3hr1 | PF9 | A | 999 |
| 3hr4 | FMN | A | 999 |
| 3hrd | MCN | B | 921 |
| 3hrf | ATP | A1373 |  |
| 3hrq | PLM | A4000 |  |
| 3hrw | HEM | A | 200 |
| 3hrw | HEM | B | 200 |
| 3hs4 | AZM | A | 701 |
| 3hs5 | ACD | A | 1 |
| 3hs5 | AKR | A | 2 |
| 3hs6 | AKR | A | 2 |
| 3hs6 | EPA | A | 1 |
| 3hs7 | AKR | A | 2 |
| 3hs7 | HXA | A | 1 |
| 3hsn | HAR | A | 770 |
| 3hsn | HEM | A | 750 |
| 3hso | HAR | A | 770 |
| 3hso | HEM | A | 750 |
| 3hsp | HAR | A | 770 |
| 3hsp | HEM | A | 750 |
| 3hss | MLA | A5188 |  |
| 3hst | TAR | C | 395 |
| 3huj | NDG | A1000 |  |
| 3hv7 | 1AU | A | 361 |
| 3hvc | GG5 | A | 361 |
| 3hvh | CXS | A | 2 |
| 3hvi | D1D | A | 268 |
| 3hvs | CIT | A | 1 |
| 3hw4 | TMP | A | 257 |
| 3hwk | SIN | A | 401 |
| 3hwo | ISC | A1900 |  |
| 3hwr | BCN | A | 301 |
| 3hwr | NDP | A | 300 |
| 3hww | AKG | A | 557 |
| 3hx4 | ANP | A | 605 |
| 3hxi | GTG | A | 501 |
| 3hxn | HEM | A | 142 |
| 3hxn | HEM | B | 147 |
| 3hxu | A5A | A | 442 |
| 3hxw | SSA | A | 442 |
| 3hxz | A5A | A | 442 |
| 3hy7 | 097 | A | 801 |
| 3hyg | 099 | A | 801 |
| 3hyo | ADP | A | 281 |
| 3hys | MLA | A5188 |  |
| 3hyu | HEM | A | 142 |
| 3hyu | HEM | B | 147 |
| 3hyw | DCQ | A | 500 |
| 3hz3 | SUC | A1779 |  |
| 3hz6 | ADP | A | 510 |
| 3hz6 | XUL | A | 515 |
| 3hza | DUP | A | 201 |
| 3hzf | B72 | A | 1 |
| 3hzk | KDO | B | 303 |
| 3hzm | KDO | B | 303 |
| 3hzn | SIN | A | 219 |
| 3hzo | MLA | B5188 |  |
| 3hzt | J60 | A | 540 |
| 3hzv | KDO | A | 302 |
| 3hzy | KDO | A | 219 |
| 3i02 | KDO | A | 215 |
| 3i02 | KDO | D | 215 |
| 3i0a | DBH | B | 180 |
| 3i0r | RT3 | A | 601 |
| 3i12 | ADP | B | 365 |
| 3i12 | ADP | D | 365 |
| 3i16 | PLP | B | 427 |
| 3i26 | MAN | D5162 |  |
| 3i27 | MAN | D5162 |  |
| 3i2t | NDG | A4700 |  |
| 3i33 | ADP | A | 388 |
| 3i3b | 149 | A2001 |  |
| 3i3d | IPT | A2001 |  |
| 3i3f | BUA | B | 142 |
| 3i3q | AKG | A | 401 |
| 3i4l | ANP | A | 589 |
| 3i4x | DST | A | 460 |
| 3i4z | BU2 | A | 460 |
| 3i54 | CMP | A | 301 |
| 3i5f | ADP | A1002 |  |
| 3i5o | BGC | A2241 |  |
| 3i5u | SAM | A | 401 |
| 3i68 | J4Z | A1001 |  |
| 3i6b | KDO | B | 900 |
| 3i6d | ACJ | A | 471 |
| 3i6i | NDP | A | 350 |
| 3i6n | ISZ | A | 607 |
| 3i6q | NDP | A | 360 |
| 3i6r | J5Z | A1001 |  |
| 3i73 | ADP | A | 589 |
| 3i7v | ATP | A | 135 |
| 3i8a | N22 | X | 219 |
| 3i8r | DTV | A2001 |  |
| 3i8r | DTV | B2002 |  |
| 3i8r | HEM | B | 902 |
| 3i8v | 0MO | A | 1 |
| 3i95 | BLA | A1130 |  |
| 3i9t | DTV | A2002 |  |
| 3i9t | HEM | A | 300 |
| 3i9u | HEM | A | 300 |
| 3i9v | FMN | 1 | 440 |
| 3ia3 | HEM | B | 201 |
| 3ia4 | MTX | A | 164 |
| 3ia4 | NDP | A | 163 |
| 3iak | EV1 | A | 415 |
| 3ib0 | DIF | A | 701 |
| 3ib2 | IBP | A3960 |  |
| 3ib3 | PLM | A | 563 |
| 3ib9 | BTN | A5600 |  |
| 3ibo | REP | A | 801 |
| 3ibq | ATP | A | 281 |
| 3ic0 | B77 | A | 145 |
| 3ic0 | HEM | B | 148 |
| 3ic2 | HEM | B | 148 |
| 3ic3 | BGC | B | 99 |
| 3ic9 | FAD | A | 490 |
| 3ica | TAM | B | 211 |
| 3id6 | SAM | C | 301 |
| 3idb | ANP | A | 450 |
| 3ids | ACM | A | 360 |
| 3ie7 | ATP | A | 900 |
| 3iei | SAH | A | 601 |
| 3iew | CTP | B | 901 |
| 3if9 | GOA | A | 370 |
| 3ife | SUC | A | 416 |
| 3ife | SUC | A | 417 |
| 3igo | ANP | A | 610 |
| 3igs | 16G | A | 230 |
| 3igz | 2PG | B | 565 |
| 3igz | 3PG | B | 564 |
| 3ih0 | ANP | A | 900 |
| 3ih5 | MLA | A | 215 |
| 3iht | SAM | A | 200 |
| 3ihz | FK5 | A | 501 |
| 3ii0 | TAR | A | 435 |
| 3ii1 | BGC | A | 561 |
| 3ii4 | FAD | A | 480 |
| 3iid | APR | A | 400 |
| 3iif | APR | A | 400 |
| 3iii | PLM | A | 566 |
| 3iim | DTV | A | 173 |
| 3iiz | SAM | A1501 |  |
| 3ijc | NDS | A | 500 |
| 3iji | DGL | A | 385 |
| 3ijj | ENO | A | 901 |
| 3ijl | DGL | A | 385 |
| 3ijq | DGL | A | 385 |
| 3ijy | KDO | B | 303 |
| 3ikc | KDO | A | 213 |
| 3ikq | MAN | A | 405 |
| 3ikr | MAN | A | 405 |
| 3il5 | B82 | A | 401 |
| 3il5 | B82 | B | 401 |
| 3ilr | SGN | A | 377 |
| 3ilt | TRU | B | 800 |
| 3ilz | B72 | A | 1 |
| 3im0 | BDP | A | 301 |
| 3imy | B72 | A | 462 |
| 3in1 | ADP | A | 510 |
| 3in6 | FMN | A | 148 |
| 3in9 | H1S | A | 501 |
| 3ina | IDS | A | 503 |
| 3ina | SGN | A | 502 |
| 3inm | NDP | A | 501 |
| 3inv | NDP | A | 602 |
| 3iny | 7DG | A | 293 |
| 3ioq | E64 | A | 301 |
| 3iox | PMS | A | 900 |
| 3ip0 | HHR | A | 181 |
| 3ip9 | ABU | A | 357 |
| 3ipi | MLA | A | 304 |
| 3ipk | PMS | A | 900 |
| 3ipq | 965 | A | 801 |
| 3ipx | ADP | A | 261 |
| 3ipy | MLT | A | 261 |
| 3iq0 | ATP | B | 900 |
| 3iq7 | 5ID | A | 799 |
| 3iqe | F42 | A | 285 |
| 3iqe | H4M | A | 284 |
| 3iqv | FSC | A | 232 |
| 3ird | MLT | A | 300 |
| 3irh | DGT | A | 458 |
| 3irh | DTP | B | 459 |
| 3irn | NDP | A | 701 |
| 3iro | NDP | A | 701 |
| 3is5 | ANP | A | 1 |
| 3is8 | HEM | L | 163 |
| 3ise | HEM | E | 159 |
| 3isf | HEM | B | 159 |
| 3ish | FAD | A | 348 |
| 3iso | GSH | A | 219 |
| 3ist | SIN | A | 267 |
| 3it9 | SUC | A | 355 |
| 3itc | CIT | A | 503 |
| 3itj | CIT | A | 501 |
| 3itj | FAD | A | 400 |
| 3itu | IBM | A | 999 |
| 3iu1 | MYA | A1001 |  |
| 3iu7 | FCD | A | 288 |
| 3iup | NDP | A | 401 |
| 3iup | NDP | B | 401 |
| 3iv6 | SAM | A | 301 |
| 3iv8 | F6P | A | 500 |
| 3iva | SAH | A1401 |  |
| 3ivd | URI | A | 603 |
| 3ive | CTN | A | 603 |
| 3ivm | ZPR | A1000 |  |
| 3iw0 | HEM | A | 501 |
| 3iw1 | ASD | A1223 |  |
| 3iw2 | HEM | A | 434 |
| 3iwc | PYR | A | 63 |
| 3iwo | SUC | A | 1 |
| 3ix9 | MTX | A | 200 |
| 3ix9 | NDP | A | 193 |
| 3ixq | PGO | A | 232 |
| 3ixq | PGO | A | 233 |
| 3jq3 | ADP | A | 400 |
| 3jqm | GTP | A | 158 |
| 3jqq | A2P | A | 416 |
| 3js8 | SUC | A7021 |  |
| 3jsu | NDP | B | 610 |
| 3jsu | UMP | A | 611 |
| 3jsz | UPG | A | 526 |
| 3jt1 | UDP | A | 526 |
| 3jt3 | HEM | A | 750 |
| 3jt4 | HEM | A | 750 |
| 3jt5 | HEM | A | 750 |
| 3jt6 | HEM | A | 750 |
| 3jt7 | HEM | A | 750 |
| 3jt8 | HEM | A | 750 |
| 3jt9 | HEM | A | 750 |
| 3jta | HEM | A | 750 |
| 3ju4 | SLB | A | 1 |
| 3ju6 | ANP | A6001 |  |
| 3ju8 | SIN | A | 504 |
| 3juk | UPG | A | 282 |
| 3jus | ECN | A | 602 |
| 3jux | ADP | A | 873 |
| 3jv2 | ADP | A | 873 |
| 3jvv | ACP | A | 400 |
| 3jvv | CIT | A | 357 |
| 3jwa | MPJ | A | 399 |
| 3jwh | SAH | A | 1 |
| 3jwo | FUL | A | 790 |
| 3jwr | IBM | A | 901 |
| 3jws | HEM | A | 750 |
| 3jwt | HEM | A | 750 |
| 3jwu | HEM | A | 750 |
| 3jww | HEM | B | 500 |
| 3jx0 | HEM | A | 750 |
| 3jx1 | HEM | A | 750 |
| 3jx2 | HEM | A | 750 |
| 3jx3 | HEM | A | 750 |
| 3jx4 | HEM | A | 750 |
| 3jx5 | HEM | A | 750 |
| 3jx6 | HEM | A | 750 |
| 3jxa | NDG | A | 2 |
| 3jyf | TAM | B | 343 |
| 3jz0 | APC | A | 738 |
| 3jzb | 4HY | A | 1 |
| 3jzc | 4HY | A | 1 |
| 3jzm | ATP | A | 903 |
| 3k00 | MTT | A | 405 |
| 3k09 | ATP | A | 903 |
| 3k09 | ATP | C | 903 |
| 3k0a | ATP | A | 903 |
| 3k0a | ATP | B | 903 |
| 3k0c | ATP | A | 903 |
| 3k0c | ATP | C | 903 |
| 3k0e | ATP | A | 903 |
| 3k0f | ATP | A | 903 |
| 3k0f | ATP | B | 903 |
| 3k0t | BGC | A | 127 |
| 3k13 | THH | A | 642 |
| 3k1l | CIT | A2503 |  |
| 3k1l | MAL | A5045 |  |
| 3k1w | NDG | A | 344 |
| 3k2g | DTV | A | 402 |
| 3k2h | LYA | A | 513 |
| 3k2h | LYA | A | 514 |
| 3k2v | CMK | A | 1 |
| 3k30 | ADP | A | 803 |
| 3k30 | ADP | B | 803 |
| 3k34 | SUA | A1003 |  |
| 3k3c | PLM | A | 157 |
| 3k3i | I46 | A | 2 |
| 3k3s | MLA | A | 85 |
| 3k4f | HEM | A | 300 |
| 3k4h | MAL | A | 501 |
| 3k4q | IHS | A | 500 |
| 3k4s | 0MO | A | 414 |
| 3k4y | IPE | A | 300 |
| 3k4y | IPE | B | 300 |
| 3k4z | CBI | A | 294 |
| 3k5h | ATP | B | 400 |
| 3k5i | ADP | A | 400 |
| 3k5k | SAH | A1198 |  |
| 3k5v | STI | A | 2 |
| 3k6v | CIT | A | 501 |
| 3k7m | FAD | X | 434 |
| 3k7q | FAD | X | 434 |
| 3k7r | MLT | D | 77 |
| 3k7t | FAD | A | 434 |
| 3k7y | PLP | A | 406 |
| 3k83 | 1DO | B | 580 |
| 3k87 | FAD | A | 600 |
| 3k8b | HEM | A | 150 |
| 3k8b | HEM | B | 150 |
| 3k8d | KDO | A1244 |  |
| 3k8m | 3SA | A | 693 |
| 3k8m | MAL | A | 740 |
| 3k8t | DGT | A | 890 |
| 3k91 | PS5 | A | 154 |
| 3k9w | ADE | A | 168 |
| 3k9y | CM5 | A | 516 |
| 3k9y | HEM | A | 520 |
| 3k9z | HEM | A | 155 |
| 3ka7 | FAD | A | 500 |
| 3kaa | PSF | B | 120 |
| 3kak | 3GC | A | 501 |
| 3kal | ADP | A | 500 |
| 3kaz | BU2 | A | 189 |
| 3kb1 | ADP | A | 301 |
| 3kb2 | G3D | A | 180 |
| 3kb6 | PPI | A | 398 |
| 3kb8 | 5GP | A | 266 |
| 3kb8 | SUC | A | 268 |
| 3kbn | GLO | A | 401 |
| 3kbo | NDP | A | 313 |
| 3kcc | CMP | A | 301 |
| 3kcc | CMP | A | 303 |
| 3kco | GLO | A | 401 |
| 3ke7 | BCN | A | 2 |
| 3ke8 | EIP | A | 998 |
| 3ke9 | IPE | A | 998 |
| 3kec | HAE | A | 272 |
| 3ked | MLA | A | 990 |
| 3ked | MLA | A | 991 |
| 3kef | DMA | A | 998 |
| 3keg | MRE | A | 214 |
| 3kem | IPE | A | 998 |
| 3keu | ATP | A | 407 |
| 3keu | PLP | A | 500 |
| 3kex | ANP | A | 1 |
| 3kf3 | FRU | A | 600 |
| 3kfe | ADP | A | 545 |
| 3kfx | ADP | A | 401 |
| 3kfy | NDP | A1160 |  |
| 3kgt | GEN | B3071 |  |
| 3kgu | GEN | A3071 |  |
| 3kgu | GEN | B3071 |  |
| 3kgy | NDP | A | 301 |
| 3kh5 | ADP | A | 281 |
| 3kh6 | APR | A | 800 |
| 3khm | HEM | A | 500 |
| 3khm | TPF | A | 501 |
| 3kia | 5GP | A | 336 |
| 3kiv | ACA | A | 100 |
| 3kjd | 78P | A | 1 |
| 3kjg | ADP | A | 500 |
| 3kji | ADP | A | 256 |
| 3kk6 | BMA | A | 674 |
| 3kk6 | HEM | A | 601 |
| 3kk6 | NDG | A | 682 |
| 3kkj | FAD | A | 401 |
| 3kl0 | HIS | A | 405 |
| 3kl0 | MLA | A | 402 |
| 3kl0 | MLA | A | 403 |
| 3kl0 | TAR | A | 404 |
| 3kl3 | BDP | B | 402 |
| 3kl5 | GCV | B | 405 |
| 3klb | FMN | A | 162 |
| 3klj | FAD | A | 401 |
| 3kll | MAL | A1779 |  |
| 3klo | TAR | B | 226 |
| 3km0 | AOM | C | 1 |
| 3km6 | EAA | A | 222 |
| 3km8 | 9DI | A | 353 |
| 3kmb | MAG | 1 | 223 |
| 3kmo | EAA | A | 214 |
| 3kmt | SAH | A | 120 |
| 3kmw | ATP | A | 502 |
| 3kn3 | CIT | C | 261 |
| 3kn5 | ANP | A | 400 |
| 3ko0 | TFP | A | 201 |
| 3ko1 | ADP | A | 800 |
| 3ko5 | ADP | A | 165 |
| 3ko9 | DAR | B | 165 |
| 3kob | DGL | B | 165 |
| 3kp6 | SAL | A3002 |  |
| 3kpb | SAM | A1000 |  |
| 3kpc | MTA | A3615 |  |
| 3kpc | SAM | A1000 |  |
| 3kpd | MTA | A | 1 |
| 3kpd | SAM | B1000 |  |
| 3kpv | ADE | A | 290 |
| 3kqj | UD1 | A | 450 |
| 3kr0 | 172 | A1001 |  |
| 3kr4 | BES | A1003 |  |
| 3kr4 | BES | G1003 |  |
| 3kr5 | BEY | A1003 |  |
| 3kr5 | BEY | B1003 |  |
| 3krk | ACD | A | 700 |
| 3krk | AKR | A | 622 |
| 3kro | DST | D2002 |  |
| 3ksw | HEM | A | 488 |
| 3ksx | MPO | A | 325 |
| 3ksz | 3PG | O | 337 |
| 3kt7 | AKG | A | 646 |
| 3kta | AP5 | A | 183 |
| 3ktj | CXP | H | 1 |
| 3ku1 | SAM | A | 226 |
| 3ku2 | ANP | A | 508 |
| 3ku9 | SPM | A | 700 |
| 3kuk | DUR | A | 311 |
| 3kv2 | NNH | A | 901 |
| 3kv3 | 3PG | O | 337 |
| 3kv6 | AKG | A | 702 |
| 3kve | FAD | A | 487 |
| 3kvl | DET | A | 400 |
| 3kvr | URF | A2001 |  |
| 3kvs | BLA | A | 484 |
| 3kvv | URF | A | 254 |
| 3kvy | URA | A | 312 |
| 3kwa | SPM | A | 300 |
| 3kwm | 3GR | D | 225 |
| 3kwt | B3P | A1001 |  |
| 3kx2 | ADP | A1000 |  |
| 3kyb | FMN | A | 392 |
| 3kyb | FMN | B | 394 |
| 3kyf | 5GP | A | 501 |
| 3kyg | 5GP | A | 501 |
| 3kyt | HC2 | A1001 |  |
| 3kzi | DGD | B | 533 |
| 3kzi | DGD | C | 474 |
| 3kzi | HEM | V | 164 |
| 3kzk | OLN | A | 345 |
| 3l01 | MTT | A5360 |  |
| 3l02 | SN0 | A | 345 |
| 3l0l | HC3 | A1001 |  |
| 3l0q | XUL | A | 553 |
| 3l0s | TAR | A | 225 |
| 3l1c | ADP | A | 998 |
| 3l1n | PLM | A | 161 |
| 3l1r | SPD | A | 700 |
| 3l24 | GOA | A | 521 |
| 3l2f | ADP | A | 601 |
| 3l2f | ADP | L | 612 |
| 3l2f | NMG | A | 501 |
| 3l2f | NMG | C | 503 |
| 3l2g | ADP | A | 601 |
| 3l2h | CXS | A | 164 |
| 3l2j | MAL | A | 194 |
| 3l3x | DHT | A | 1 |
| 3l3z | DHT | A | 1 |
| 3l4p | PCD | A | 931 |
| 3l4s | 3PG | O | 338 |
| 3l53 | TAR | A7110 |  |
| 3l5u | ZEC | A | 123 |
| 3l69 | BER | A1811 |  |
| 3l6g | B3P | A | 1 |
| 3l6h | BET | A | 1 |
| 3l6t | CIT | A | 300 |
| 3l70 | HEM | C | 502 |
| 3l70 | PEE | A2008 |  |
| 3l70 | PEE | C2007 |  |
| 3l70 | PEE | E2005 |  |
| 3l71 | AZO | C2001 |  |
| 3l71 | HEM | C | 502 |
| 3l71 | PEE | A2008 |  |
| 3l72 | PEE | C2008 |  |
| 3l73 | HEM | C | 502 |
| 3l73 | PEE | A2008 |  |
| 3l74 | FMX | C2001 |  |
| 3l74 | HEM | C | 501 |
| 3l74 | PEE | C2008 |  |
| 3l74 | PEE | N3008 |  |
| 3l75 | HEM | C | 501 |
| 3l75 | PEE | A2008 |  |
| 3l7k | C2G | D | 730 |
| 3l7l | C2G | D | 730 |
| 3l7m | EDT | A | 738 |
| 3l8a | PLP | A | 388 |
| 3l8k | ADP | A | 465 |
| 3l8w | XAN | A | 300 |
| 3l8x | N4D | A | 361 |
| 3l91 | OCA | B | 1 |
| 3l94 | MYR | B | 800 |
| 3l99 | OAA | B | 428 |
| 3l9w | FMN | A2400 |  |
| 3l9w | GSH | B | 1 |
| 3l9x | FMN | A2400 |  |
| 3la2 | AKG | A | 224 |
| 3la6 | ADP | A1000 |  |
| 3lb0 | CIT | A | 253 |
| 3lb1 | IOL | A | 191 |
| 3lbo | CIT | A | 700 |
| 3lbo | LDT | A | 600 |
| 3lby | SAH | A4630 |  |
| 3lc0 | HIS | A | 501 |
| 3lcb | ATP | A1760 |  |
| 3lct | ADP | A1412 |  |
| 3lcu | SAH | A6732 |  |
| 3lcv | SAM | B | 301 |
| 3ld5 | CIT | A | 700 |
| 3ld5 | LDT | A | 600 |
| 3ldg | SAH | A | 385 |
| 3ldj | SCR | B6124 |  |
| 3ldk | SUC | A | 1 |
| 3ldu | GTP | A | 383 |
| 3len | ZST | A | 600 |
| 3leo | PAM | A1151 |  |
| 3lev | ATP | H | 219 |
| 3lf5 | HEM | A | 1 |
| 3lfi | BGC | A | 1 |
| 3lfz | ADP | A | 282 |
| 3lfz | ATP | A | 281 |
| 3lgn | HEM | A | 200 |
| 3lgs | ADE | A | 269 |
| 3lgs | SAH | A | 268 |
| 3lht | BMQ | A | 229 |
| 3lhu | BMQ | A | 229 |
| 3lhv | BMQ | A | 229 |
| 3lhw | BMQ | A | 229 |
| 3lhy | BMQ | A | 229 |
| 3lhz | BMQ | A | 229 |
| 3li0 | BMQ | A | 229 |
| 3li1 | BMQ | A | 229 |
| 3lif | CIT | A | 294 |
| 3lij | ANP | A | 522 |
| 3lik | HAE | A | 302 |
| 3lil | HAE | A | 302 |
| 3lj0 | ADP | A2101 |  |
| 3lj0 | QUE | A | 1 |
| 3lj1 | DKI | A | 1 |
| 3lj2 | IZA | A | 1 |
| 3ljg | HAE | A | 301 |
| 3ljj | 10U | A | 501 |
| 3ljo | 11U | A | 501 |
| 3ljr | GGC | A | 301 |
| 3lka | HAE | A | 269 |
| 3lki | ATP | B | 900 |
| 3ll3 | ATP | A1760 |  |
| 3ll3 | DXP | A3264 |  |
| 3ll4 | 2FP | A | 301 |
| 3ll5 | ADP | A | 246 |
| 3ll5 | IPE | A | 247 |
| 3ll6 | SIN | A | 338 |
| 3lla | ACP | A1304 |  |
| 3llf | UP6 | A | 229 |
| 3lli | FAD | A | 550 |
| 3llk | FAD | A | 550 |
| 3llm | ADP | A | 565 |
| 3llt | ANP | A | 877 |
| 3lm4 | HPX | A | 406 |
| 3lm8 | VIB | A | 223 |
| 3lm9 | ADP | A3001 |  |
| 3lm9 | FRU | A1001 |  |
| 3lmg | ANP | A | 301 |
| 3lmh | ADP | A1511 |  |
| 3lmi | ATP | A1760 |  |
| 3lmx | DHB | M | 4 |
| 3lmy | CP6 | A | 562 |
| 3lmy | NDG | A | 560 |
| 3lmy | NDG | B | 561 |
| 3lmz | CIT | A | 1 |
| 3ln0 | HEM | A | 605 |
| 3ln1 | HEM | A | 605 |
| 3lnj | DAR | B | 12 |
| 3lnj | DGL | B | 8 |
| 3lnj | DGL | F | 8 |
| 3lnj | DTR | B | 3 |
| 3lnl | B3P | A | 371 |
| 3lo8 | FAD | A | 415 |
| 3loc | URA | A | 212 |
| 3log | SIN | A | 450 |
| 3lok | DJK | A1345 |  |
| 3lp0 | NVP | A | 701 |
| 3lp1 | NVP | A | 701 |
| 3lp7 | HAR | A | 906 |
| 3lp9 | SPM | D | 230 |
| 3lpn | APC | A | 287 |
| 3lq2 | TDP | A | 887 |
| 3lqd | HEM | A | 142 |
| 3lqd | HEM | B | 147 |
| 3lqf | MRY | A | 258 |
| 3lqg | 388 | A | 600 |
| 3lqq | ATP | A | 602 |
| 3lqv | ADE | A | 1 |
| 3lr7 | HEM | A | 154 |
| 3lr9 | HEM | A | 154 |
| 3lrt | ADP | A | 300 |
| 3ls3 | SPD | A | 227 |
| 3lsj | PLM | A | 221 |
| 3lst | SAH | A | 346 |
| 3lsw | 4MP | A | 801 |
| 3lsz | GSH | A | 300 |
| 3lt8 | ADP | A | 81 |
| 3lt9 | ADP | A | 81 |
| 3lta | ATP | A | 81 |
| 3ltb | ADP | A | 81 |
| 3ltc | ATP | A | 81 |
| 3ltd | ADP | A | 82 |
| 3ltp | BMP | A | 229 |
| 3lts | BMP | A | 229 |
| 3lty | BMP | A | 229 |
| 3lu1 | UD2 | A | 344 |
| 3luh | 5GP | A | 3 |
| 3lus | MCO | B1001 |  |
| 3luy | PPY | A | 501 |
| 3lv2 | SFG | A | 600 |
| 3lv5 | BMP | A | 229 |
| 3lv6 | BMP | A | 229 |
| 3lv8 | ADP | A | 213 |
| 3lv8 | TMP | A | 214 |
| 3lv8 | TYD | A | 215 |
| 3lvv | ADP | A | 694 |
| 3lvw | GSH | A | 693 |
| 3lw6 | UDP | A | 312 |
| 3lx9 | A2G | A1000 |  |
| 3lxk | MI1 | A1125 |  |
| 3lxl | IZA | A | 1 |
| 3lxn | MI1 | A | 1 |
| 3lxp | IZA | A1183 |  |
| 3lxv | 4NC | M | 1 |
| 3lxy | SUC | A | 701 |
| 3ly1 | CIT | A | 501 |
| 3ly1 | PLP | A | 500 |
| 3ly6 | ATP | A | 700 |
| 3lzw | FAD | A1000 |  |
| 3lzx | FAD | A1000 |  |
| 3m0h | RNS | A2001 |  |
| 3m0i | ZST | A | 600 |
| 3m0o | FAD | A | 400 |
| 3m1f | ATP | A | 400 |
| 3m1j | MBO | A | 301 |
| 3m1z | BMP | A | 229 |
| 3m23 | HEM | A | 296 |
| 3m25 | HEM | A | 296 |
| 3m26 | HEM | A | 296 |
| 3m27 | HEM | A | 296 |
| 3m28 | HEM | A | 296 |
| 3m29 | HEM | A | 296 |
| 3m2a | HEM | A | 296 |
| 3m2b | HEM | A | 296 |
| 3m2c | HEM | A | 296 |
| 3m2d | HEM | A | 296 |
| 3m2e | HEM | A | 296 |
| 3m2f | HEM | A | 296 |
| 3m2g | HEM | A | 296 |
| 3m2h | HEM | A | 296 |
| 3m2i | HEM | A | 296 |
| 3m2n | BCN | A | 266 |
| 3m31 | FAD | A | 634 |
| 3m38 | HEM | A | 155 |
| 3m39 | HEM | A | 155 |
| 3m3a | HEM | A | 155 |
| 3m3b | HEM | A | 155 |
| 3m3n | ATP | A | 400 |
| 3m3r | BCD | F | 663 |
| 3m49 | TDP | A | 701 |
| 3m4f | CXS | A | 206 |
| 3m4h | 388 | A | 600 |
| 3m53 | SAH | A | 800 |
| 3m54 | SAH | A | 800 |
| 3m55 | SAH | A | 1 |
| 3m56 | SAH | A | 800 |
| 3m57 | SAH | A | 1 |
| 3m58 | SAH | A | 800 |
| 3m59 | SAH | A | 1 |
| 3m5a | SAH | A | 1 |
| 3m5h | SIA | A | 1 |
| 3m5i | SIA | A | 1 |
| 3m5j | SIA | A | 331 |
| 3m5q | HEM | A | 396 |
| 3m5q | MAN | A | 364 |
| 3m6a | ADP | A | 783 |
| 3m6g | ATP | A1380 |  |
| 3m6l | R5P | A | 701 |
| 3m6r | BB2 | A | 194 |
| 3m6w | SAM | A | 465 |
| 3m7i | RP5 | A | 801 |
| 3m7j | MMA | A | 277 |
| 3m7o | NDG | B | 201 |
| 3m7s | CBI | A | 274 |
| 3m89 | GSP | A | 408 |
| 3m8p | 65B | A | 562 |
| 3m9k | D1D | B | 106 |
| 3m9v | TYD | A | 901 |
| 3m9y | CIT | B | 256 |
| 3ma8 | CIT | A | 535 |
| 3maa | TAT | B | 1 |
| 3mb5 | SAM | A | 301 |
| 3mb9 | ZST | A | 600 |
| 3mbh | PXL | A | 400 |
| 3mbi | ADP | A | 288 |
| 3mbo | MLT | A | 388 |
| 3mct | 3MC | A | 600 |
| 3md7 | 5GP | A | 303 |
| 3mdm | HEM | A | 505 |
| 3mdp | SIN | A | 142 |
| 3mdr | HEM | A | 505 |
| 3mdt | HEM | A | 505 |
| 3mdv | HEM | A | 505 |
| 3mdz | EZL | A | 264 |
| 3me3 | FBP | A | 541 |
| 3mec | 65B | A | 561 |
| 3med | 65B | A | 561 |
| 3mee | T27 | A | 561 |
| 3meg | T27 | A | 561 |
| 3mek | SAM | A | 510 |
| 3mes | DME | A | 427 |
| 3mfl | DHY | M | 539 |
| 3mg0 | BO2 | 21405 |  |
| 3mg0 | BO2 | K1402 |  |
| 3mg5 | BTN | A5100 |  |
| 3mgb | PAP | A | 286 |
| 3mgc | PME | A | 290 |
| 3mgx | HEM | A | 397 |
| 3mhy | AKG | A | 114 |
| 3mi0 | DMF | 1 | 249 |
| 3mi0 | DMF | 2 | 101 |
| 3mit | MAN | A | 801 |
| 3miu | MAN | A | 801 |
| 3miy | B49 | A | 1 |
| 3mje | NDP | A | 476 |
| 3mjh | GTP | A | 200 |
| 3mjp | HEM | A | 150 |
| 3mjr | AC2 | A | 301 |
| 3mjr | UDP | A | 261 |
| 3mjs | NDP | A | 476 |
| 3mjt | NDP | A | 476 |
| 3mju | HEM | A | 150 |
| 3mju | HEM | B | 150 |
| 3mjv | NDP | A | 476 |
| 3mk1 | NPO | A | 911 |
| 3mkb | HEM | B | 137 |
| 3mkf | MA4 | A | 600 |
| 3mkk | BGC | A | 802 |
| 3ml5 | AZM | A | 264 |
| 3mlm | MYR | A | 124 |
| 3mmv | ATP | A | 800 |
| 3mn0 | HEM | A | 155 |
| 3mn5 | ATP | A | 800 |
| 3mn6 | ATP | A | 800 |
| 3mn7 | ATP | A | 800 |
| 3mn8 | GEM | A | 601 |
| 3mn9 | ATP | A | 800 |
| 3mne | DEX | A | 784 |
| 3mng | D1D | A | 201 |
| 3mno | DEX | A | 784 |
| 3mnp | DEX | A | 784 |
| 3mnq | STL | A | 902 |
| 3mnu | MBO | A | 263 |
| 3mo0 | SAH | A | 101 |
| 3mo2 | SAH | C | 103 |
| 3mo5 | SAH | C | 103 |
| 3moe | GTP | A1100 |  |
| 3moe | SPV | A1200 |  |
| 3mof | GTP | A | 900 |
| 3mol | HEM | A | 185 |
| 3mp1 | MAL | A | 600 |
| 3mp5 | HMG | B | 401 |
| 3mp6 | MAL | A | 600 |
| 3mp8 | MAL | A | 1 |
| 3mpb | FRU | A | 229 |
| 3mpi | FAD | A | 400 |
| 3mpj | FAD | A | 400 |
| 3mpn | MTN | A | 801 |
| 3mq2 | SAH | A | 216 |
| 3mqe | HEM | A | 605 |
| 3mqg | UDP | B | 192 |
| 3mrg | CIT | A | 294 |
| 3ms3 | ANL | A | 601 |
| 3ms9 | STI | A | 1 |
| 3mss | STI | A | 1 |
| 3muf | ADP | A | 202 |
| 3muf | S3P | A | 201 |
| 3muo | ZPR | A | 702 |
| 3muz | IPT | 12002 |  |
| 3muz | IPT | 22001 |  |
| 3mv0 | 149 | 12001 |  |
| 3mv6 | DHB | M | 539 |
| 3mvc | HEM | A | 500 |
| 3mvr | CM5 | A | 601 |
| 3mvx | DTV | B | 507 |
| 3mw9 | GTP | A | 503 |
| 3mwd | CIT | A | 426 |
| 3mx2 | TTP | A | 800 |
| 3mx5 | UTP | A | 993 |
| 3mxr | MA4 | A | 400 |
| 3mxs | MA4 | A | 400 |
| 3mxu | CIT | A | 123 |
| 3my5 | RFZ | A | 300 |
| 3my5 | SGM | B | 1 |
| 3myk | ANP | X | 999 |
| 3myk | BIT | X | 800 |
| 3myu | VIB | A | 500 |
| 3mzb | DTD | A | 522 |
| 3mzs | HEM | B | 500 |
| 3n0g | DST | A | 601 |
| 3n0i | SIA | B | 1 |
| 3n0y | APC | A | 181 |
| 3n0z | 3AT | A | 181 |
| 3n10 | CMP | A | 181 |
| 3n1a | CHQ | A1514 |  |
| 3n1c | F6P | A | 400 |
| 3n1s | 5GP | A | 201 |
| 3n21 | PGO | A | 601 |
| 3n25 | PYR | A1000 |  |
| 3n2g | GTP | A | 600 |
| 3n2i | THM | A | 214 |
| 3n2k | GTP | A | 600 |
| 3n2r | HEM | A | 750 |
| 3n2v | JT5 | A | 0 |
| 3n31 | FCA | A | 247 |
| 3n33 | AES | A | 376 |
| 3n3a | FMN | C | 134 |
| 3n3h | CIT | A | 323 |
| 3n3o | ADP | B | 751 |
| 3n3r | HEM | A1500 |  |
| 3n3x | GUN | A | 247 |
| 3n3z | IBM | A | 1 |
| 3n45 | ZOL | F | 354 |
| 3n4a | PGO | A | 601 |
| 3n5d | BGC | A1970 |  |
| 3n5q | HEM | B | 500 |
| 3n5r | HEM | B | 500 |
| 3n5v | HEM | A | 750 |
| 3n5w | HEM | A | 750 |
| 3n5x | HEM | A | 750 |
| 3n5y | HEM | A | 750 |
| 3n5z | HEM | A | 750 |
| 3n60 | HEM | A | 750 |
| 3n61 | HEM | A | 750 |
| 3n62 | HEM | A | 750 |
| 3n63 | HEM | A | 750 |
| 3n64 | HEM | A | 750 |
| 3n65 | HEM | A | 750 |
| 3n66 | HEM | A | 750 |
| 3n68 | HEM | B | 500 |
| 3n69 | HEM | B | 500 |
| 3n6a | HEM | B | 500 |
| 3n6c | HEM | B | 500 |
| 3n6d | HEM | B | 500 |
| 3n6r | BTI | A | 801 |
| 3n6u | TSU | A1003 |  |
| 3n71 | SFG | A | 491 |
| 3n75 | G4P | A | 716 |
| 3n7a | FA1 | A | 147 |
| 3n83 | ADP | A | 501 |
| 3n8d | ANP | A | 400 |
| 3n8v | HEM | A | 801 |
| 3n8w | HEM | A | 801 |
| 3n8w | HEM | B | 601 |
| 3n8w | NDG | A | 672 |
| 3n8w | NDG | B1672 |  |
| 3n8x | HEM | A | 601 |
| 3n8y | DIF | A | 701 |
| 3n8y | DIF | B | 585 |
| 3n8y | NDG | A | 672 |
| 3n8y | NDG | B1672 |  |
| 3n8y | SAL | B | 900 |
| 3n8z | HEM | A | 601 |
| 3n93 | MAL | A | 111 |
| 3n94 | MAL | A5044 |  |
| 3n95 | MAL | A | 111 |
| 3n96 | MAL | A | 111 |
| 3n98 | BGC | A | 563 |
| 3n9j | EAA | A | 210 |
| 3n9w | PGO | A3002 |  |
| 3n9y | HEM | A | 601 |
| 3n9z | HEM | A | 601 |
| 3na0 | HEM | A | 601 |
| 3na1 | HEM | A | 601 |
| 3na3 | ATP | A | 500 |
| 3nag | ADP | B | 300 |
| 3nai | URF | A | 521 |
| 3nat | CIT | A | 162 |
| 3nb0 | G6P | A | 901 |
| 3nb8 | BLA | A | 249 |
| 3nb9 | BLA | A | 249 |
| 3nbq | URF | A | 400 |
| 3ncr | ADP | A | 120 |
| 3ndi | SAM | A | 601 |
| 3ndi | TMP | A | 901 |
| 3ndj | SAH | A | 415 |
| 3ndx | RIT | A | 100 |
| 3ndz | CT3 | A | 1 |
| 3nec | DTV | A | 164 |
| 3nee | B72 | A | 1 |
| 3nee | B72 | B | 1 |
| 3nem | AMO | A | 439 |
| 3nem | ATP | B1039 |  |
| 3neo | G24 | B | 1 |
| 3nex | G24 | A | 126 |
| 3nf4 | FAD | A | 384 |
| 3nfe | HEM | B | 400 |
| 3nfm | FRU | A | 693 |
| 3ng0 | ANP | A | 474 |
| 3ng4 | MAL | D | 173 |
| 3ng7 | FAD | X | 434 |
| 3ngb | BGC | A | 494 |
| 3ngb | BGC | A | 508 |
| 3ngb | BGC | B | 406 |
| 3ngb | BGC | C | 504 |
| 3ngc | FAD | X | 434 |
| 3ngu | ADP | A | 152 |
| 3nh3 | FAD | X | 434 |
| 3nh9 | ATP | A | 1 |
| 3nha | ADP | A | 2 |
| 3nhb | ADP | A | 1 |
| 3nho | FAD | X | 434 |
| 3nie | ANP | A | 430 |
| 3niz | ADP | A | 314 |
| 3njv | GTR | A | 510 |
| 3njv | GTR | A | 512 |
| 3njz | SAL | A | 370 |
| 3nk0 | FAD | X | 434 |
| 3nk1 | FAD | X | 434 |
| 3nk2 | FAD | X | 434 |
| 3nk2 | LDP | X | 433 |
| 3nk7 | SAM | A | 770 |
| 3nkq | MAN | A | 904 |
| 3nks | ACJ | A | 478 |
| 3nl3 | TPS | A2001 |  |
| 3nl5 | TZE | B | 542 |
| 3nl6 | ACP | A | 799 |
| 3nl6 | TPS | A2001 |  |
| 3nl7 | HEM | A | 142 |
| 3nl7 | HEM | B | 147 |
| 3nlc | FAD | A | 601 |
| 3nld | HEM | B | 500 |
| 3nlh | HEM | B | 500 |
| 3nlj | HEM | A | 750 |
| 3nlk | HEM | A | 750 |
| 3nlm | HEM | A | 750 |
| 3nlo | HEM | A | 750 |
| 3nlp | HEM | A | 750 |
| 3nlq | HEM | A | 750 |
| 3nlr | HEM | A | 750 |
| 3nlt | HEM | B | 500 |
| 3nlv | HEM | A | 750 |
| 3nlw | HEM | A | 750 |
| 3nlx | HEM | A | 750 |
| 3nly | HEM | A | 750 |
| 3nlz | HEM | A | 750 |
| 3nm0 | HEM | A | 750 |
| 3nm1 | IFP | A | 542 |
| 3nm3 | TPS | A2001 |  |
| 3nmi | PXX | A | 151 |
| 3nml | HEM | A | 154 |
| 3nmm | HEM | B | 147 |
| 3nn0 | FAD | X | 434 |
| 3nn0 | NCA | X | 433 |
| 3nn1 | HEM | A | 239 |
| 3nnb | CIT | A | 402 |
| 3nnt | DQA | A | 253 |
| 3nny | HEM | A | 750 |
| 3nnz | HEM | A | 750 |
| 3no2 | CIT | A | 2 |
| 3nod | SCI | A | 906 |
| 3noj | PYR | A | 240 |
| 3nok | DDQ | A2745 |  |
| 3nor | CIT | A | 232 |
| 3npc | B96 | A | 365 |
| 3npd | CXS | A | 3 |
| 3nq1 | KOJ | A1351 |  |
| 3nq3 | DKA | A3053 |  |
| 3nq7 | BMP | A | 229 |
| 3nq9 | OCA | A1111 |  |
| 3nqe | BMP | A | 229 |
| 3nqf | BMP | A | 229 |
| 3nqs | AT2 | A | 906 |
| 3nqs | HEM | A | 901 |
| 3nrc | TCL | A | 262 |
| 3nrr | D16 | A | 520 |
| 3nrr | D16 | A | 530 |
| 3nrz | FAD | B | 606 |
| 3nrz | HPA | C | 1 |
| 3nsg | TAM | A | 390 |
| 3nsn | CTO | A | 1 |
| 3nsr | URF | A | 5 |
| 3nsz | ANP | A1000 |  |
| 3nt1 | HEM | A | 619 |
| 3nt1 | NPS | A | 5 |
| 3nt4 | INS | A | 801 |
| 3ntb | HEM | A | 619 |
| 3ntg | HEM | A | 601 |
| 3ntr | INS | A | 801 |
| 3nua | CIT | A | 243 |
| 3nv2 | NDG | A | 502 |
| 3nv4 | BGC | A | 502 |
| 3nv4 | SIA | A | 500 |
| 3nv8 | PEP | A | 467 |
| 3nvn | NDG | A | 1 |
| 3nvs | GPJ | A | 429 |
| 3nvs | S3P | A | 427 |
| 3nvw | FAD | B | 606 |
| 3nvw | GUN | C | 503 |
| 3nvy | QUE | C1356 |  |
| 3nw2 | HEM | A | 901 |
| 3nwf | MRY | A4000 |  |
| 3nwn | ADP | A | 401 |
| 3nwy | GTP | A | 262 |
| 3nwy | UDP | B | 263 |
| 3nx9 | MAL | A | 249 |
| 3nxu | RIT | A | 600 |
| 3ny8 | OLA | A1204 |  |
| 3nyc | FAD | A1500 |  |
| 3nye | FAD | A1500 |  |
| 3nyf | FAD | A1500 |  |
| 3nyi | STE | A | 301 |
| 3nyq | MCA | A | 486 |
| 3nyr | MLC | A | 486 |
| 3nyv | DTQ | A | 701 |
| 3nz0 | IZA | A | 1 |
| 3nzj | ABN | 3 | 5 |
| 3nzk | C90 | A | 501 |
| 3nzp | PLP | A | 701 |
| 3nzw | ABN | 3 | 5 |
| 3o01 | DXC | B | 1 |
| 3o03 | GCO | A | 274 |
| 3o05 | PLP | A | 1 |
| 3o0n | TTP | A1001 |  |
| 3o0o | TTP | A1001 |  |
| 3o0q | TTP | A1001 |  |
| 3o19 | OLA | A | 200 |
| 3o19 | PLM | A | 201 |
| 3o1a | HEM | A | 385 |
| 3o22 | OLA | A | 191 |
| 3o22 | PLM | A | 192 |
| 3o2k | DST | A | 501 |
| 3o2n | FAD | A | 232 |
| 3o2y | OLA | A | 200 |
| 3o2y | PLM | A | 201 |
| 3o3c | UDP | A | 901 |
| 3o4t | TAR | A | 3 |
| 3o52 | TAR | A7110 |  |
| 3o5r | FK5 | A1001 |  |
| 3o5s | B3P | A | 244 |
| 3o6d | PXP | A | 801 |
| 3o6x | ADP | A4001 |  |
| 3o76 | GTB | A | 210 |
| 3o7b | SAH | A | 301 |
| 3o8d | ADP | A | 875 |
| 3o8e | DTD | A2816 |  |
| 3o8m | BGC | A | 487 |
| 3o8t | BMU | A | 361 |
| 3o94 | NCA | A | 192 |
| 3o97 | IAC | A1001 |  |
| 3o9z | AKG | A | 312 |
| 3oau | MAN | H | 228 |
| 3oay | BDF | H | 229 |
| 3ob7 | GSH | A | 581 |
| 3obd | HEM | A5154 |  |
| 3obh | CIT | A | 201 |
| 3obl | CXS | A | 134 |
| 3obp | URC | A | 302 |
| 3obt | SLB | A | 1 |
| 3obv | SUC | A2001 |  |
| 3obv | SUC | A2005 |  |
| 3occ | DIH | A | 500 |
| 3occ | DIH | D | 500 |
| 3ocj | PLM | A | 305 |
| 3ock | HEM | A4154 |  |
| 3ocm | ADP | A | 470 |
| 3ocp | CMP | A | 250 |
| 3ocu | NMN | A2003 |  |
| 3od0 | PCG | A | 250 |
| 3odg | XAN | A | 288 |
| 3odq | HEM | A | 142 |
| 3odq | HEM | B | 147 |
| 3odu | OLA | A1610 |  |
| 3odv | CIT | B | 40 |
| 3oe2 | TAR | A | 1 |
| 3oe7 | ANP | A | 600 |
| 3oe7 | ANP | D | 600 |
| 3oea | BGC | A | 202 |
| 3oeb | BMA | A | 501 |
| 3oeb | MAN | A | 500 |
| 3oee | ANP | A | 600 |
| 3oee | ANP | D | 600 |
| 3oeh | ANP | A | 600 |
| 3oeh | ANP | D | 600 |
| 3oel | DGL | A | 287 |
| 3oez | STI | A | 601 |
| 3of1 | CMP | A | 601 |
| 3of3 | DIH | D | 500 |
| 3of4 | FAD | C | 250 |
| 3ofk | SAH | A | 217 |
| 3ofn | ANP | A | 600 |
| 3ofn | ANP | D | 600 |
| 3og2 | MAN | A1028 |  |
| 3og2 | MAN | A1040 |  |
| 3og9 | MLT | A | 500 |
| 3ogb | HEM | A | 154 |
| 3ogj | CMP | A | 250 |
| 3ogs | IPT | A1024 |  |
| 3ogv | MAN | A1029 |  |
| 3ogx | H1S | C | 173 |
| 3ohr | ADP | A | 300 |
| 3ohs | 2HA | X1336 |  |
| 3oid | NDP | A | 501 |
| 3oid | TCL | A | 601 |
| 3oif | TCL | A | 502 |
| 3ois | UDP | A | 292 |
| 3ojf | NDP | A | 501 |
| 3ojk | 4NC | A | 601 |
| 3ojy | BMA | A | 556 |
| 3ojy | BMA | B | 539 |
| 3okd | KDO | B | 214 |
| 3okk | KDO | B | 225 |
| 3okl | KDO | B | 216 |
| 3okn | KDO | A | 216 |
| 3oko | KDO | B | 302 |
| 3okw | CIT | A2001 |  |
| 3okx | SAM | A | 201 |
| 3om2 | CIT | A | 485 |
| 3om9 | OXQ | A | 702 |
| 3onb | CIT | A | 800 |
| 3onb | LDT | A | 600 |
| 3onc | CIT | A | 800 |
| 3onc | LDT | A | 600 |
| 3one | ADE | A | 506 |
| 3onz | HEM | B | 148 |
| 3onz | MBN | A | 145 |
| 3onz | MBN | B | 149 |
| 3oo4 | HEM | B | 147 |
| 3ope | SAM | A | 7 |
| 3oph | MA4 | A | 1 |
| 3opl | MA4 | A | 400 |
| 3opp | MA4 | A | 293 |
| 3opr | MA4 | A | 400 |
| 3opt | AKG | A | 501 |
| 3oq2 | CIT | A | 111 |
| 3oq6 | PFB | A | 378 |
| 3oq8 | MLA | A | 440 |
| 3oqv | DTD | A | 248 |
| 3orz | BI4 | A | 360 |
| 3os9 | KN1 | A | 1 |
| 3ot9 | G16 | A | 400 |
| 3otb | DGT | A | 301 |
| 3otf | CMP | A | 725 |
| 3oti | TYD | A | 377 |
| 3otk | UDP | A | 598 |
| 3otx | AP5 | A | 346 |
| 3ou2 | SAH | A | 300 |
| 3ou6 | SAM | A | 300 |
| 3ou7 | SAM | A | 300 |
| 3ouj | AKG | A | 417 |
| 3out | DGL | A | 266 |
| 3ouu | ANP | A | 460 |
| 3ouz | ADP | A | 460 |
| 3ov6 | D12 | A1104 |  |
| 3ovm | FAD | A | 234 |
| 3ovq | 5RP | A | 230 |
| 3ovu | HEM | C | 142 |
| 3owa | FAD | A | 601 |
| 3owb | BSM | A | 1 |
| 3owh | FAD | A | 434 |
| 3ox1 | FAD | A | 232 |
| 3oxf | SAH | A | 437 |
| 3oxg | SAH | A | 432 |
| 3oxh | PMB | A | 301 |
| 3oxl | SAH | A | 432 |
| 3oy8 | GCO | A | 501 |
| 3oyx | GLV | A | 501 |
| 3ozf | HPA | A | 232 |
| 3ozl | FLF | A | 501 |
| 3ozl | FLF | B | 502 |
| 3ozu | FAD | A | 405 |
| 3ozv | ECN | A | 411 |
| 3ozw | DGG | A | 416 |
| 3ozx | ADP | A | 615 |
| 3p03 | CHT | C2486 |  |
| 3p0h | FSE | A | 701 |
| 3p0w | GKR | A | 472 |
| 3p10 | CTN | A | 165 |
| 3p23 | ADP | A1002 |  |
| 3p2e | SAH | A6732 |  |
| 3p2h | MTA | A | 204 |
| 3p2k | SAM | A6735 |  |
| 3p3b | TAR | A | 395 |
| 3p3l | HEM | A | 501 |
| 3p3o | HEM | A | 501 |
| 3p3x | HEM | A | 501 |
| 3p3z | HEM | A | 501 |
| 3p41 | IPE | A | 299 |
| 3p4e | CIT | A | 503 |
| 3p4p | FAD | M | 601 |
| 3p4p | FMR | A | 577 |
| 3p4q | OAA | A | 702 |
| 3p4r | GUA | A | 577 |
| 3p4w | PLC | A | 320 |
| 3p4x | ADP | A | 800 |
| 3p50 | PFL | A | 319 |
| 3p50 | PLC | D | 322 |
| 3p5d | MAN | A | 402 |
| 3p5e | MAN | A | 402 |
| 3p5f | MAN | A | 402 |
| 3p5h | BGC | A | 400 |
| 3p5q | HEM | B | 147 |
| 3p5y | BMP | A | 229 |
| 3p5z | BMP | A | 229 |
| 3p60 | BMP | A | 229 |
| 3p61 | BMP | A | 229 |
| 3p6c | CIT | A | 133 |
| 3p6h | IBP | A | 133 |
| 3p6l | CIT | A | 303 |
| 3p7g | MAN | A | 339 |
| 3p7h | MAL | A | 339 |
| 3p7x | DTV | A | 170 |
| 3p8f | GSH | A1001 |  |
| 3p8g | GSH | A1001 |  |
| 3p93 | KDG | C | 407 |
| 3p9c | DTV | A | 900 |
| 3p9d | ADP | A | 756 |
| 3p9d | ADP | B | 756 |
| 3p9d | ADP | C | 756 |
| 3p9d | ADP | D | 756 |
| 3p9d | ADP | E | 756 |
| 3p9d | ADP | F | 756 |
| 3p9d | ADP | G | 756 |
| 3p9d | ADP | H | 756 |
| 3p9e | ADP | a1756 |  |
| 3p9e | ADP | b1756 |  |
| 3p9e | ADP | e1756 |  |
| 3p9e | ADP | f1756 |  |
| 3p9e | ADP | g1756 |  |
| 3p9e | ADP | h1756 |  |
| 3p9e | ADP | k1756 |  |
| 3p9e | ADP | l1756 |  |
| 3p9t | TCL | A | 221 |
| 3pa1 | A2G | A | 3 |
| 3pa9 | PMP | A | 600 |
| 3paa | PMP | A | 600 |
| 3pak | MAN | A | 500 |
| 3pan | HPA | A | 328 |
| 3pao | ADE | A | 328 |
| 3paq | MMA | A | 501 |
| 3pas | PGO | A | 212 |
| 3pax | 3MB | A | 1 |
| 3pb3 | SAH | A6732 |  |
| 3pb8 | AHN | X | 390 |
| 3pb9 | 1BN | X | 390 |
| 3pbl | MAL | A1500 |  |
| 3pbu | UP6 | A | 229 |
| 3pbv | UP6 | A | 229 |
| 3pbw | UP6 | A | 229 |
| 3pby | UP6 | A | 229 |
| 3pc0 | UP6 | A | 229 |
| 3pc3 | HEM | A | 701 |
| 3pcr | GTP | B1230 |  |
| 3pd2 | A3S | A | 500 |
| 3pd5 | TSB | A | 501 |
| 3pdb | OAA | B | 1 |
| 3pdb | PMP | B | 432 |
| 3pde | IPE | A | 309 |
| 3pdn | SFG | A | 429 |
| 3pdt | ADP | A | 811 |
| 3pe3 | UDP | A1201 |  |
| 3pe4 | UDP | A1212 |  |
| 3pel | HEM | B | 147 |
| 3pen | 5GP | A | 1 |
| 3pf7 | MLA | A1004 |  |
| 3pff | ADP | A | 833 |
| 3pfg | SAM | A | 264 |
| 3pg6 | CIT | A1000 |  |
| 3pgb | MAN | A1104 |  |
| 3pgu | OLA | A | 448 |
| 3pgy | CIT | A | 505 |
| 3phq | KDO | B | 218 |
| 3phz | SIA | A | 303 |
| 3pi8 | HEM | A | 142 |
| 3pi8 | HEM | B | 147 |
| 3pi9 | HEM | B | 147 |
| 3pia | HEM | A | 142 |
| 3pia | HEM | B | 147 |
| 3pij | FRU | A | 527 |
| 3piy | 585 | A | 1 |
| 3pjd | TCL | A | 801 |
| 3pje | TCL | A | 801 |
| 3pjf | TCL | A | 502 |
| 3pjg | CXS | A | 900 |
| 3pjg | UGA | A | 902 |
| 3pko | CIT | A | 326 |
| 3pkp | DTP | A | 500 |
| 3pkq | DGT | A | 500 |
| 3pl2 | CIT | B | 400 |
| 3pl9 | CHL | A | 614 |
| 3pl9 | HTG | A | 631 |
| 3pl9 | HTG | A | 632 |
| 3pls | ANP | A1358 |  |
| 3pm5 | PGO | A | 487 |
| 3pma | SCR | B | 248 |
| 3pna | CMP | A | 250 |
| 3pnd | FAD | A | 500 |
| 3png | HEM | A | 750 |
| 3pnh | HEM | B | 500 |
| 3pnl | ADP | B1211 |  |
| 3pnq | 2HA | D | 511 |
| 3pns | URA | B | 271 |
| 3po7 | FAD | A | 600 |
| 3pp2 | CIT | A | 1 |
| 3pp7 | SVR | B | 499 |
| 3ppl | PLP | A | 500 |
| 3ppp | BET | A | 401 |
| 3ppq | CHT | A | 401 |
| 3pps | MAN | A | 715 |
| 3pqr | PLM | A | 701 |
| 3pqv | TAR | A | 401 |
| 3pr2 | F9F | A | 1 |
| 3pr3 | F6P | A | 580 |
| 3prj | UDX | A | 501 |
| 3prq | DGD | A | 370 |
| 3prq | DGD | C | 490 |
| 3prq | DGD | E | 362 |
| 3prq | DGD | H | 208 |
| 3prq | HEM | F | 85 |
| 3prq | HEM | V | 164 |
| 3prq | LHG | A | 371 |
| 3prq | MST | A | 367 |
| 3prr | DGD | A5370 |  |
| 3prr | DGD | C5490 |  |
| 3prr | DGD | E5362 |  |
| 3prr | DGD | H5208 |  |
| 3prr | HEM | F5085 |  |
| 3prr | HEM | V5164 |  |
| 3prr | LHG | A5371 |  |
| 3prr | MST | A5367 |  |
| 3ps9 | FAD | A | 669 |
| 3ps9 | SAM | A | 670 |
| 3pt1 | F6P | A1001 |  |
| 3pt9 | SAH | A1603 |  |
| 3ptm | G2F | A1000 |  |
| 3ptq | NFG | A1001 |  |
| 3ptz | UDX | A | 501 |
| 3pug | GLV | A | 501 |
| 3puq | AKG | A | 4 |
| 3puw | UMQ | F5004 |  |
| 3pvt | 3HC | A | 310 |
| 3pwh | ZMA | A | 401 |
| 3px2 | SAH | A | 263 |
| 3pxf | 2AN | A | 304 |
| 3pxp | MYR | A | 301 |
| 3pxq | 2AN | A | 300 |
| 3pxz | 2AN | A | 299 |
| 3py0 | SU9 | A | 301 |
| 3py1 | 2AN | A | 301 |
| 3py1 | SU9 | A | 300 |
| 3py6 | TAR | A | 304 |
| 3pyy | STI | A | 3 |
| 3pz2 | GRG | B | 334 |
| 3q12 | PAF | A | 501 |
| 3q27 | MAL | A5044 |  |
| 3q2j | CKI | A | 300 |
| 3q2q | IPE | A | 359 |
| 3q2v | MAN | A | 804 |
| 3q2w | MAN | A | 701 |
| 3q31 | MLT | A | 3 |
| 3q31 | MLT | B | 3 |
| 3q3a | FUL | A | 1 |
| 3q3h | UDP | A | 621 |
| 3q4o | B3P | A | 200 |
| 3q4p | M7G | A | 247 |
| 3q4q | B3P | A | 203 |
| 3q4r | B3P | A | 203 |
| 3q4t | TAK | A | 2 |
| 3q60 | ATP | A | 600 |
| 3q6j | KPC | A | 526 |
| 3q6k | CIT | A | 382 |
| 3q6p | CIT | B | 382 |
| 3q6z | APR | A | 1 |
| 3q78 | FPS | B | 524 |
| 3q7a | 778 | B3012 |  |
| 3q7a | SUC | A3010 |  |
| 3q7g | SCR | A | 1 |
| 3q7i | 6PG | A | 601 |
| 3q86 | GTP | A | 158 |
| 3q87 | SAM | B | 300 |
| 3q8g | PEE | A | 311 |
| 3q8i | IND | A | 127 |
| 3q8u | ADP | A | 158 |
| 3q8v | UDP | A | 158 |
| 3q8x | UD1 | D | 288 |
| 3q99 | HEM | A | 750 |
| 3q9a | HEM | A | 750 |
| 3q9f | CXS | A | 401 |
| 3qae | CIT | A | 502 |
| 3qb0 | ATP | A | 490 |
| 3qc9 | ADP | C | 700 |
| 3qcz | ANP | A | 501 |
| 3qe2 | FAD | A | 752 |
| 3qe2 | FMN | A | 751 |
| 3qe7 | URA | A | 430 |
| 3qed | TAM | C | 347 |
| 3qej | UDP | A | 401 |
| 3qen | UDP | A | 401 |
| 3qeo | UDP | A | 401 |
| 3qf7 | ANP | A | 853 |
| 3qfk | AKG | A | 514 |
| 3qfs | FAD | A | 752 |
| 3qfu | ADP | A | 501 |
| 3qfx | CP6 | A | 602 |
| 3qfx | NDP | A | 601 |
| 3qfy | IFM | A1903 |  |
| 3qg2 | CP6 | B | 709 |
| 3qg2 | NDP | B | 710 |
| 3qgt | CP6 | A | 609 |
| 3qgt | NDP | A | 610 |
| 3qh0 | AKR | A | 2 |
| 3qh0 | PLM | A | 625 |
| 3qhd | MSR | A | 166 |
| 3qhr | ADP | A | 297 |
| 3qhw | ADP | A | 297 |
| 3qi4 | IBM | A | 534 |
| 3qip | NVP | A | 561 |
| 3qj4 | FAD | A | 401 |
| 3qj5 | N2P | A | 379 |
| 3qj7 | SPM | A | 264 |
| 3qj7 | UMP | A | 600 |
| 3qjb | HEM | A | 142 |
| 3qjb | HEM | B | 147 |
| 3qjc | HEM | A | 142 |
| 3qjc | HEM | B | 147 |
| 3qjd | HEM | B | 147 |
| 3qje | HEM | B | 147 |
| 3qkw | UDP | A | 400 |
| 3ql0 | FOL | A | 160 |
| 3ql3 | FOL | A | 161 |
| 3qlm | PLM | A | 127 |
| 3qls | 55V | A | 194 |
| 3qlw | N22 | A | 194 |
| 3qly | 55V | A | 229 |
| 3qme | CYH | A | 200 |
| 3qmk | IDS | A | 1 |
| 3qmk | SGN | A | 2 |
| 3qmn | A3P | O2575 |  |
| 3qmu | NDP | A | 552 |
| 3qnd | SIA | A | 1 |
| 3qnq | CBS | A | 500 |
| 3qnq | CIT | A | 444 |
| 3qom | BGC | A | 479 |
| 3qov | ADP | A | 437 |
| 3qow | SAM | A | 417 |
| 3qp2 | HTF | A | 1 |
| 3qpb | R1P | A1254 |  |
| 3qpb | URA | A1255 |  |
| 3qps | CHD | A | 211 |
| 3qqa | TCH | A | 211 |
| 3qrw | NDP | A | 301 |
| 3qsp | MAN | A | 428 |
| 3qss | MTG | A | 405 |
| 3qtc | ANP | A | 810 |
| 3qtt | ANP | A | 271 |
| 3qu2 | CIT | A | 227 |
| 3quo | ATP | A1260 |  |
| 3quo | FCN | A4001 |  |
| 3qur | ADP | A1260 |  |
| 3qut | MLT | A | 227 |
| 3qv4 | CTO | B2735 |  |
| 3qv4 | DGL | C2992 |  |
| 3qvf | ADP | A1260 |  |
| 3qvh | ADP | A1260 |  |
| 3qvp | FAD | A | 600 |
| 3qvq | G3P | A | 301 |
| 3qwp | SAM | A | 510 |
| 3qwv | SAH | A | 434 |
| 3qx8 | GTG | A | 28 |
| 3qx9 | ATP | A | 1 |
| 3qxc | ATP | A | 221 |
| 3qxh | ADP | A | 222 |
| 3qxj | GTP | A | 221 |
| 3qxs | ANP | A | 222 |
| 3qxt | MTX | A2000 |  |
| 3qxv | MTX | A2000 |  |
| 3qyq | SUC | A | 2 |
| 3qzj | OLA | A | 999 |
| 3qzm | HEM | A | 200 |
| 3qzo | HEM | A | 200 |
| 3r03 | ADP | A | 1 |
| 3r0k | TAR | A | 381 |
| 3r0u | TAR | A | 384 |
| 3r0v | MLT | A | 264 |
| 3r11 | FMR | B | 385 |
| 3r12 | PGO | A | 250 |
| 3r1b | SUC | A | 601 |
| 3r1k | ACM | A | 403 |
| 3r1p | PLM | A | 128 |
| 3r1r | ATP | A | 762 |
| 3r1z | DGL | B | 481 |
| 3r2h | CXS | A | 155 |
| 3r3z | GOA | A | 305 |
| 3r4s | SIA | A6859 |  |
| 3r4s | SLB | A6875 |  |
| 3r5i | HEM | B | 148 |
| 3r5x | ATP | A | 311 |
| 3r69 | CIT | B2502 |  |
| 3r6n | D1D | A | 1 |
| 3r6u | CHT | A | 285 |
| 3r6w | NFZ | A | 213 |
| 3r70 | ADP | A | 401 |
| 3r80 | LLL | A | 302 |
| 3r8d | PNU | A | 1 |
| 3r9u | FAD | A | 501 |
| 3ra7 | DOG | L3123 |  |
| 3rcm | CIT | A | 289 |
| 3rcq | MLT | A | 769 |
| 3rcy | RIB | A | 433 |
| 3rdm | BTN | A | 300 |
| 3rdo | BTN | A | 300 |
| 3rdq | DTB | A | 300 |
| 3reg | GSP | A | 538 |
| 3rep | ATP | A | 502 |
| 3res | ADP | B | 66 |
| 3ret | PYR | A | 301 |
| 3ret | SAL | A | 201 |
| 3rf4 | FUN | A | 201 |
| 3rf6 | G3P | A | 501 |
| 3rfa | SAM | A | 406 |
| 3rfm | CFF | A | 330 |
| 3rfq | B3P | A | 182 |
| 3rg1 | MAN | B | 904 |
| 3rg1 | MAN | B | 905 |
| 3rg9 | NDP | A | 601 |
| 3rg9 | WRA | A | 602 |
| 3rgk | HEM | A | 154 |
| 3rhr | NDP | A | 903 |
| 3rhz | UDP | A7517 |  |
| 3ri3 | EMO | A | 302 |
| 3ri3 | NDP | A | 301 |
| 3rib | SAH | A | 445 |
| 3riv | HEM | A | 305 |
| 3riw | HEM | A | 305 |
| 3rju | CIT | A | 391 |
| 3rjw | SAH | A1198 |  |
| 3rk8 | PYR | A | 292 |
| 3rl4 | 5GP | A | 951 |
| 3rlb | VIB | A | 191 |
| 3rlf | MAL | F2000 |  |
| 3rlf | UMQ | E5004 |  |
| 3rnj | EDT | A | 1 |
| 3rnm | FAD | A | 480 |
| 3ro0 | TPT | A | 480 |
| 3rod | NCA | A | 302 |
| 3rpe | FAD | A | 195 |
| 3rpn | GTX | A | 302 |
| 3rq1 | OAA | A | 422 |
| 3rq1 | OAA | B | 422 |
| 3rq6 | APR | A | 278 |
| 3rq8 | AP5 | A | 278 |
| 3rqi | CIT | A | 182 |
| 3rqt | HIS | A | 471 |
| 3rre | ADP | A | 493 |
| 3rre | ADP | A | 494 |
| 3rrf | ATP | A | 493 |
| 3rrj | AP5 | A | 492 |
| 3rrm | ADP | A | 1 |
| 3rrn | ADP | A | 1 |
| 3rs5 | DMF | A | 201 |
| 3rs8 | APR | A | 493 |
| 3rsj | SIA | A | 5 |
| 3rt6 | FWD | B | 401 |
| 3rt7 | ADQ | A | 493 |
| 3rt7 | ADQ | A | 494 |
| 3rtb | A3P | A | 492 |
| 3rtg | ATP | A | 494 |
| 3rtl | HEM | A | 200 |
| 3rtx | GUN | A | 1 |
| 3ru0 | SFG | A | 437 |
| 3ru2 | NDP | A | 492 |
| 3ruc | UD2 | A | 344 |
| 3ruf | UDP | A | 344 |
| 3rur | HEM | A | 200 |
| 3rv3 | ADP | A1000 |  |
| 3rv5 | DXC | A | 91 |
| 3rxi | TSS | A | 6 |
| 3ry2 | BTN | A5001 |  |
| 3ry6 | FUL | B | 802 |
| 3ry6 | NDG | A | 801 |
| 3ry9 | 1CA | A | 249 |
| 3ryc | GTP | A | 600 |
| 3ryc | GTP | D | 600 |
| 3ryf | GTP | A | 600 |
| 3ryf | GTP | B | 600 |
| 3ryh | G2P | B | 600 |
| 3ryh | GTP | A | 600 |
| 3ryi | GTP | A | 600 |
| 3rys | ADE | A | 345 |
| 3rza | CIT | A | 406 |
| 3s1i | HEM | A | 140 |
| 3s1j | HEM | A | 140 |
| 3s1s | SAH | A | 900 |
| 3s1u | E4P | A | 224 |
| 3s28 | MLA | A | 921 |
| 3s29 | FRU | A | 902 |
| 3s2z | DHC | A | 259 |
| 3s42 | MLA | A | 586 |
| 3s5w | FAD | A | 450 |
| 3s5x | BMA | A | 201 |
| 3s61 | FAD | A | 444 |
| 3s61 | NDP | A | 446 |
| 3s65 | HEM | A | 142 |
| 3s65 | HEM | B | 147 |
| 3s66 | HEM | A | 142 |
| 3s66 | HEM | B | 147 |
| 3s6e | CIT | B | 603 |
| 3s7b | SAM | A1000 |  |
| 3s7d | SAH | A1000 |  |
| 3s7f | SAM | A1000 |  |
| 3s7j | SAM | A1000 |  |
| 3s7z | SUC | B | 302 |
| 3s8f | HEM | A | 800 |
| 3s8h | 3OH | A | 726 |
| 3s99 | ADE | A | 400 |
| 3s9c | BGC | A | 701 |
| 3sbt | PGO | A2093 |  |
| 3sdj | APJ | A1999 |  |
| 3se7 | ATP | A | 400 |
| 3sfd | OAA | A | 701 |
| 3sfe | TMG | C | 1 |
| 3sfx | FII | B | 525 |
| 3sfz | ADP | A1250 |  |
| 3sg8 | TOY | A | 305 |
| 3sgl | SAM | A | 692 |
| 3sgw | MLA | A | 164 |
| 3sh3 | A3B | A | 241 |
| 3shf | ADP | A1250 |  |
| 3shr | CMP | A | 401 |
| 3sit | SIA | A6535 |  |
| 3siz | BMP | A | 229 |
| 3sj3 | BMP | A | 229 |
| 3sj5 | HEM | A | 500 |
| 3sj6 | RIP | A | 249 |
| 3sju | NDP | A | 301 |
| 3sld | A2G | A | 1 |
| 3slf | URA | A | 270 |
| 3sms | ADP | A | 373 |
| 3smt | SAM | A1000 |  |
| 3sn0 | FMR | A | 500 |
| 3sn4 | AKG | A | 500 |
| 3sn5 | HEM | A | 601 |
| 3soc | GVD | A | 1 |
| 3sp3 | SUC | A | 130 |
| 3spk | TPV | A | 100 |
| 3sq6 | EPJ | A | 300 |
| 3sqp | FAD | A | 500 |
| 3ssm | SAH | D | 601 |
| 3ssn | SAH | A | 601 |
| 3sso | SAH | A | 601 |
| 3sso | SAH | C | 601 |
| 3ssx | TAM | N | 1 |
| 3std | MQ0 | A | 503 |
| 3suc | ATP | A | 856 |
| 3svp | HEM | A | 750 |
| 3svq | HEM | A | 750 |
| 3swr | SFG | A | 300 |
| 3sxs | PP2 | A | 1 |
| 3sy0 | KDO | B | 302 |
| 3sy5 | UP6 | A | 229 |
| 3szk | HEM | A | 142 |
| 3szk | HEM | B | 147 |
| 3t02 | PAE | A | 503 |
| 3t0n | PHB | A | 401 |
| 3t18 | PLP | A | 501 |
| 3t1d | DBH | B | 182 |
| 3t1d | DBS | C | 179 |
| 3t2z | BU2 | A | 502 |
| 3t31 | DCQ | A | 502 |
| 3t4j | ZIP | A | 400 |
| 3t4y | KDO | B | 303 |
| 3t65 | KDO | B | 303 |
| 3t6c | GCO | A | 601 |
| 3t6u | SUC | A | 130 |
| 3t77 | KDO | B | 303 |
| 3t7m | UDP | A | 264 |
| 3t7o | UPG | A | 265 |
| 3t8s | I3P | B | 603 |
| 3t96 | IWD | B | 601 |
| 3tem | FAD | A | 232 |
| 3tf6 | DBH | B | 216 |
| 3tfz | CXS | A | 171 |
| 3tg4 | SAM | A | 434 |
| 3the | BCN | A | 500 |
| 3tkd | CYZ | A | 266 |
| 3tku | M77 | A | 418 |
| 3tm0 | ANP | A | 300 |
| 3to3 | ATP | B | 617 |
| 3tos | SAH | A | 258 |
| 3tpq | ATP | A1377 |  |
| 3tq5 | UMP | A | 777 |
| 3tsd | TAR | B | 501 |
| 3tt5 | BER | A1811 |  |
| 3tw5 | CXS | A | 1 |
| 3tw6 | BTI | B2003 |  |
| 3tw6 | PAE | C2003 |  |
| 3tyg | MAN | A | 215 |
| 3tzl | ADP | A | 330 |
| 3u04 | BB2 | A | 210 |
| 3upj | U03 | A | 100 |
| 3vh4 | ATP | A | 702 |
| 3vtk | 5IU | A | 500 |
| 3xis | XYS | A | 393 |
| 3zq9 | BGC | A1520 |  |
| 3zr0 | 8OG | A1157 |  |
| 3zst | ACX | A | 700 |
| 3zt5 | MAL | A | 701 |
| 3zt6 | ACX | A | 701 |
| 3zt6 | MAL | A | 703 |
| 3zt7 | BCD | A | 701 |
| 3zt7 | MAL | A | 703 |
| 3zud | TAM | A1229 |  |
| 3zux | TCH | A1313 |  |
| 3zuy | TCH | A1312 |  |
| 3zv5 | BPY | A1278 |  |
| 3zyb | PHB | I | 300 |
| 3zyf | 147 | A1001 |  |
| 4a34 | FUL | A1142 |  |
| 4aig | FLX | A | 400 |
| 4cpp | ADM | A | 422 |
| 4csm | TSA | A | 400 |
| 4cyh | HIS | A | 201 |
| 4erk | OLO | A | 800 |
| 4fab | FDS | H | 218 |
| 4fua | PGH | A | 217 |
| 4gsp | SGP | A | 106 |
| 4kmb | G4S | 1 | 222 |
| 4lip | CCP | D | 950 |
| 4pah | LNR | A | 600 |
| 4pax | NU1 | A | 1 |
| 4pbg | BGP | A | 469 |
| 4pfk | F6P | A | 323 |
| 4req | MCA | A | 802 |
| 4req | SCA | A | 801 |
| 4rhn | RIB | A | 201 |
| 4sli | CNP | A | 760 |
| 4std | BFS | A | 173 |
| 4tln | LNO | A | 322 |
| 4tmk | T5A | A | 214 |
| 4uag | UAG | A | 460 |
| 4ubp | HAE | C | 800 |
| 4ukd | UDP | A | 196 |
| 4upj | U04 | A | 100 |
| 5acn | TRC | A | 899 |
| 5cpp | ADO | A | 422 |
| 5eau | FFF | A | 800 |
| 5grt | TS4 | A | 17 |
| 5ktq | DCP | A | 901 |
| 5mdh | MAK | A | 335 |
| 5nse | HAR | A | 770 |
| 5p2p | DHG | A | 126 |
| 5pah | LDP | A | 600 |
| 5prc | ATZ | L | 502 |
| 5rhn | 8BR | A | 201 |
| 5std | UNN | B | 173 |
| 5upj | UIN | B | 100 |
| 5yas | FAC | A | 300 |
| 6cgt | DAG | A | 702 |
| 6cgt | OPG | A | 703 |
| 6cox | S58 | A | 701 |
| 6cpa | ZAF | A | 309 |
| 6enl | PGA | A | 442 |
| 6est | DMF | A | 246 |
| 6fit | AMW | A | 148 |
| 6gpb | H2P | A | 998 |
| 6jdw | ABU | A | 500 |
| 6nse | GGB | A | 720 |
| 6pfk | PGA | A | 325 |
| 6std | MS2 | A | 601 |
| 6tim | G3P | B | 300 |
| 7abp | FCA | A | 307 |
| 7abp | FCB | A | 308 |
| 7cpp | NCM | A | 422 |
| 7jdw | DAV | A | 500 |
| 7req | 2CP | A1801 |  |
| 7taa | ABC | A | 479 |
| 7tln | INC | A | 322 |
| 7upj | INU | A | 100 |
| 830c | RS1 | A | 1 |
| 8a3h | IDC | A | 500 |
| 8cgt | TM6 | A | 701 |
| 8cpp | TCM | A | 422 |
| 966c | RS2 | A | 1 |
| 9cgt | TM5 | A | 701 |
| 9nse | ISU | A | 800 |
