# Supplementary material for: SInCRe—structural interactome computational resource for Mycobacterium tuberculosis
Source: Database (Oxford). 2015 Jun 30;2015:bav060. doi: 10.1093/database/bav060 (PMC4485431; doi:10.1093/database/bav060)
Supplement: Supplementary Data [file supp_bav060_suppl_data.zip › SInCRe_supplementary_Table3.docx]

Supplementary Table 3: List of 2516 drug binding sites obtained from Drugport

| PDB_ID | LIG_ID | CHAINID | RESNO |
| --- | --- | --- | --- |
| 11gs | EAA | A | 211 |
| 11gs | EAA | B | 211 |
| 13gs | SAS | A | 211 |
| 13gs | SAS | B | 211 |
| 1a27 | EST | A | 350 |
| 1a28 | STR | A | 1 |
| 1a28 | STR | B | 2 |
| 1a29 | TFP | A | 153 |
| 1a29 | TFP | A | 154 |
| 1a4g | ZMR | A | 466 |
| 1a4g | ZMR | B | 466 |
| 1a4l | DCF | A | 353 |
| 1a4l | DCF | B | 853 |
| 1a4l | DCF | C | 1353 |
| 1a4l | DCF | D | 1853 |
| 1a52 | EST | A | 1 |
| 1a52 | EST | B | 2 |
| 1acj | THA | A | 999 |
| 1acl | DME | A | 999 |
| 1aj0 | SAN | A | 561 |
| 1aj6 | NOV | A | 1 |
| 1am6 | HAE | A | 555 |
| 1aqu | EST | A | 304 |
| 1aqu | EST | B | 303 |
| 1ax9 | EDR | A | 999 |
| 1azm | AZM | A | 262 |
| 1b3n | CER | A | 413 |
| 1bcu | PRL | H | 280 |
| 1bkf | FK5 | A | 108 |
| 1bsx | T3 | A | 1 |
| 1bsx | T3 | B | 2 |
| 1bt5 | IM2 | A | 350 |
| 1bx4 | ADN | A | 350 |
| 1bx4 | ADN | A | 355 |
| 1c3s | SHH | A | 952 |
| 1c51 | PQN | A | 601 |
| 1c51 | PQN | B | 623 |
| 1c6y | MK1 | B | 524 |
| 1c6z | ROC | B | 505 |
| 1c8l | CFF | A | 940 |
| 1c9h | RAP | A | 108 |
| 1cea | ACA | A | 90 |
| 1cea | ACA | B | 90 |
| 1ceb | AMH | A | 90 |
| 1ceb | AMH | B | 90 |
| 1cet | CLQ | A | 1001 |
| 1cil | ETS | A | 263 |
| 1cla | CLM | A | 221 |
| 1cqe | FLP | A | 1650 |
| 1cqe | FLP | B | 2650 |
| 1cqp | 803 | A | 311 |
| 1cqp | 803 | B | 311 |
| 1ctr | TFP | A | 153 |
| 1d4f | ADN | A | 601 |
| 1d4f | ADN | B | 602 |
| 1d4f | ADN | C | 603 |
| 1d4f | ADN | D | 604 |
| 1d4s | TPV | A | 201 |
| 1d4y | TPV | A | 501 |
| 1dbb | STR | H | 229 |
| 1dg5 | TOP | A | 201 |
| 1dgm | ADN | A | 375 |
| 1dmy | AZM | A | 400 |
| 1dmy | AZM | B | 900 |
| 1dtl | BEP | A | 204 |
| 1dtl | BEP | A | 205 |
| 1dtl | BEP | A | 206 |
| 1dvt | FLP | A | 125 |
| 1dvx | DIF | B | 125 |
| 1dwc | MIT | H | 1 |
| 1dx6 | GNT | A | 1537 |
| 1dyr | TOP | A | 407 |
| 1e6w | EST | L | 350 |
| 1e7a | PFL | A | 4001 |
| 1e7a | PFL | A | 4002 |
| 1e7a | PFL | B | 4001 |
| 1e7a | PFL | B | 4002 |
| 1e7b | HLT | A | 4001 |
| 1e7b | HLT | A | 4002 |
| 1e7b | HLT | A | 4003 |
| 1e7b | HLT | B | 4001 |
| 1e7b | HLT | B | 4002 |
| 1e7b | HLT | B | 4003 |
| 1e7c | HLT | A | 4001 |
| 1e7c | HLT | A | 4002 |
| 1e7c | HLT | A | 4004 |
| 1e7c | HLT | A | 4005 |
| 1e7c | HLT | A | 4006 |
| 1e7c | HLT | A | 4007 |
| 1e7c | HLT | A | 4008 |
| 1e9y | HAE | B | 800 |
| 1ea1 | TPF | A | 470 |
| 1ei6 | PPF | A | 410 |
| 1ei6 | PPF | C | 413 |
| 1ei6 | PPF | D | 412 |
| 1eqg | IBP | A | 701 |
| 1eqg | IBP | B | 1701 |
| 1eqh | FLP | A | 701 |
| 1eqh | FLP | B | 1701 |
| 1ere | EST | A | 600 |
| 1ere | EST | B | 600 |
| 1ere | EST | C | 600 |
| 1ere | EST | D | 600 |
| 1ere | EST | E | 600 |
| 1ere | EST | F | 600 |
| 1err | RAL | A | 600 |
| 1err | RAL | B | 600 |
| 1eta | T44 | 1 | 128 |
| 1eta | T44 | 2 | 129 |
| 1etb | T44 | 1 | 128 |
| 1etb | T44 | 2 | 129 |
| 1etr | MIT | H | 1 |
| 1eve | E20 | A | 2001 |
| 1f5l | AMR | A | 301 |
| 1f86 | T44 | A | 428 |
| 1f86 | T44 | B | 528 |
| 1fap | RAP | A | 108 |
| 1fb7 | ROC | A | 100 |
| 1fds | EST | A | 350 |
| 1fdt | EST | A | 350 |
| 1fdu | EST | A | 351 |
| 1fdu | EST | B | 354 |
| 1fdu | EST | C | 353 |
| 1fdu | EST | D | 352 |
| 1fdw | EST | A | 350 |
| 1fiq | SAL | C | 1335 |
| 1fj8 | CER | A | 500 |
| 1fj8 | CER | B | 500 |
| 1fj8 | CER | C | 500 |
| 1fj8 | CER | D | 500 |
| 1fk9 | EFZ | A | 999 |
| 1fkb | RAP | A | 108 |
| 1fkf | FK5 | A | 108 |
| 1fkj | FK5 | A | 108 |
| 1fkl | RAP | A | 108 |
| 1fko | EFZ | A | 999 |
| 1fkp | NVP | A | 999 |
| 1flr | FLU | L | 600 |
| 1fm6 | BRL | D | 503 |
| 1fm6 | BRL | X | 504 |
| 1fmo | ADN | E | 351 |
| 1fo4 | SAL | A | 3005 |
| 1fo4 | SAL | B | 4005 |
| 1fr6 | AZR | A | 362 |
| 1fr6 | AZR | B | 362 |
| 1fwe | HAE | C | 989 |
| 1fxv | PNN | B | 1001 |
| 1g50 | EST | A | 600 |
| 1g50 | EST | B | 1600 |
| 1g50 | EST | C | 2600 |
| 1gfz | CFF | A | 940 |
| 1gm7 | PNN | B | 1577 |
| 1gs4 | ZK5 | A | 1918 |
| 1gse | EAA | A | 224 |
| 1gse | EAA | B | 224 |
| 1gsf | EAA | A | 223 |
| 1gsf | EAA | B | 223 |
| 1gsf | EAA | C | 223 |
| 1gsf | EAA | D | 223 |
| 1gtb | PZQ | A | 901 |
| 1gwr | EST | A | 600 |
| 1gwr | EST | B | 600 |
| 1h5x | IM2 | A | 1267 |
| 1h5x | IM2 | B | 1267 |
| 1h60 | STR | A | 500 |
| 1h61 | PDN | A | 1366 |
| 1h7x | URF | A | 1033 |
| 1h7x | URF | B | 1033 |
| 1h7x | URF | C | 1033 |
| 1h7x | URF | D | 1033 |
| 1h8s | AIC | A | 1000 |
| 1h9z | RWF | A | 3001 |
| 1hk1 | T44 | A | 3001 |
| 1hk1 | T44 | A | 3002 |
| 1hk1 | T44 | A | 3003 |
| 1hk1 | T44 | A | 3004 |
| 1hk2 | T44 | A | 3001 |
| 1hk2 | T44 | A | 3002 |
| 1hk2 | T44 | A | 3003 |
| 1hk2 | T44 | A | 3004 |
| 1hk3 | T44 | A | 3001 |
| 1hk3 | T44 | A | 3002 |
| 1hk3 | T44 | A | 3003 |
| 1hk3 | T44 | A | 3004 |
| 1hk4 | T44 | A | 1008 |
| 1hk5 | T44 | A | 1008 |
| 1ho5 | ADN | A | 1604 |
| 1ho5 | ADN | B | 2604 |
| 1hpv | 478 | B | 200 |
| 1hsg | MK1 | B | 902 |
| 1hsh | MK1 | A | 401 |
| 1hsh | MK1 | C | 402 |
| 1hvy | D16 | A | 414 |
| 1hvy | D16 | B | 415 |
| 1hvy | D16 | C | 416 |
| 1hvy | D16 | D | 417 |
| 1hwi | 115 | A | 2 |
| 1hwi | 115 | B | 1 |
| 1hwi | 115 | C | 4 |
| 1hwi | 115 | D | 3 |
| 1hwk | 117 | A | 2 |
| 1hwk | 117 | B | 1 |
| 1hwk | 117 | C | 4 |
| 1hwk | 117 | D | 3 |
| 1hwl | FBI | A | 2 |
| 1hwl | FBI | B | 1 |
| 1hwl | FBI | C | 4 |
| 1hwl | FBI | D | 3 |
| 1hxb | ROC | A | 100 |
| 1hxw | RIT | B | 301 |
| 1i00 | D16 | A | 315 |
| 1i00 | D16 | B | 409 |
| 1i1e | DM2 | A | 3001 |
| 1i2w | CFX | A | 1300 |
| 1i2w | CFX | B | 2300 |
| 1i6v | RFP | C | 1640 |
| 1ibg | OBN | H | 1 |
| 1ict | T44 | C | 128 |
| 1ict | T44 | D | 129 |
| 1ie4 | T44 | A | 128 |
| 1ie4 | T44 | B | 328 |
| 1iep | STI | A | 201 |
| 1iep | STI | B | 202 |
| 1igj | DGX | B | 228 |
| 1igj | DGX | D | 228 |
| 1ihi | IU5 | A | 326 |
| 1ihi | IU5 | B | 327 |
| 1ikv | EFZ | A | 2000 |
| 1ikw | EFZ | A | 2000 |
| 1iol | EST | A | 400 |
| 1itu | CIL | A | 451 |
| 1itu | CIL | B | 452 |
| 1iwh | PEM | A | 501 |
| 1j36 | LPR | A | 801 |
| 1j36 | LPR | B | 802 |
| 1j37 | MCO | A | 801 |
| 1j37 | MCO | B | 802 |
| 1j3j | CP6 | A | 609 |
| 1j3j | CP6 | B | 709 |
| 1jb0 | PQN | A | 2001 |
| 1jb0 | PQN | B | 2002 |
| 1jd0 | AZM | A | 1400 |
| 1jd0 | AZM | B | 2401 |
| 1jdv | ADN | A | 1260 |
| 1jdv | ADN | B | 2260 |
| 1jdv | ADN | D | 3260 |
| 1jdv | ADN | E | 4260 |
| 1jg2 | ADN | A | 500 |
| 1jg3 | ADN | A | 500 |
| 1jg3 | ADN | B | 550 |
| 1jgl | EST | L | 911 |
| 1jgs | SAL | A | 256 |
| 1jgs | SAL | A | 257 |
| 1jin | KTN | A | 801 |
| 1jip | KTN | A | 801 |
| 1jkh | EFZ | A | 999 |
| 1jlb | NVP | A | 999 |
| 1jlf | NVP | A | 999 |
| 1jnn | EST | H | 350 |
| 1jqe | QUN | A | 500 |
| 1jr1 | MOA | A | 1332 |
| 1jr1 | MOA | B | 1333 |
| 1js3 | 142 | A | 701 |
| 1js3 | 142 | B | 702 |
| 1jt1 | MCO | A | 700 |
| 1jtx | CVI | A | 200 |
| 1ju6 | LYA | A | 317 |
| 1ju6 | LYA | B | 315 |
| 1ju6 | LYA | C | 315 |
| 1ju6 | LYA | D | 315 |
| 1juj | LYA | A | 315 |
| 1juj | LYA | B | 315 |
| 1juj | LYA | C | 315 |
| 1juj | LYA | D | 315 |
| 1jvj | IM2 | A | 300 |
| 1jzs | MRC | A | 1301 |
| 1k4t | TTC | D | 990 |
| 1k6c | MK1 | B | 902 |
| 1ki2 | GA2 | A | 1 |
| 1ki2 | GA2 | B | 2 |
| 1ki3 | PE2 | A | 1 |
| 1ki3 | PE2 | B | 2 |
| 1ki7 | ID2 | A | 1 |
| 1ki7 | ID2 | B | 2 |
| 1kij | NOV | A | 400 |
| 1kij | NOV | B | 444 |
| 1klm | SPP | A | 999 |
| 1kny | KAN | A | 558 |
| 1kny | KAN | B | 559 |
| 1kvl | CLS | A | 371 |
| 1l5q | CFF | A | 863 |
| 1l5q | CFF | A | 864 |
| 1l5q | CFF | B | 1863 |
| 1l5q | CFF | B | 1864 |
| 1l7x | CFF | A | 863 |
| 1l7x | CFF | A | 864 |
| 1l7x | CFF | B | 1863 |
| 1l8t | KAN | A | 1 |
| 1lbc | CYZ | A | 330 |
| 1lbc | CYZ | B | 329 |
| 1lbc | CYZ | C | 331 |
| 1lhu | EST | A | 301 |
| 1lhv | NOG | A | 301 |
| 1lii | ADN | A | 699 |
| 1lik | ADN | A | 699 |
| 1lik | ADN | A | 799 |
| 1lin | TFP | A | 153 |
| 1lin | TFP | A | 154 |
| 1lin | TFP | A | 155 |
| 1lin | TFP | A | 156 |
| 1ll5 | IM2 | A | 370 |
| 1ll5 | IM2 | B | 370 |
| 1ll9 | AXL | B | 964 |
| 1lqp | FCN | A | 4002 |
| 1lqp | FCN | A | 4004 |
| 1lqp | FCN | B | 4001 |
| 1lqp | FCN | B | 4003 |
| 1lw0 | NVP | A | 999 |
| 1lwc | NVP | A | 999 |
| 1lwe | NVP | A | 999 |
| 1lwf | NVP | A | 999 |
| 1m17 | AQ4 | A | 999 |
| 1m2w | MTL | A | 5600 |
| 1m2w | MTL | B | 6600 |
| 1m2x | MCO | A | 811 |
| 1m2x | MCO | B | 812 |
| 1m2x | MCO | C | 813 |
| 1m2x | MCO | D | 814 |
| 1m2z | DEX | A | 301 |
| 1m2z | DEX | D | 401 |
| 1m4d | TOY | A | 500 |
| 1m4d | TOY | B | 501 |
| 1m4i | KAN | A | 500 |
| 1m4i | KAN | B | 501 |
| 1m6e | SAL | X | 2000 |
| 1m8d | CLW | A | 906 |
| 1m8d | CLW | B | 907 |
| 1m9j | CLW | A | 906 |
| 1m9j | CLW | B | 907 |
| 1maa | DME | A | 998 |
| 1maa | DME | B | 996 |
| 1maa | DME | C | 997 |
| 1maa | DME | D | 999 |
| 1me7 | MOA | A | 600 |
| 1meh | MOA | A | 600 |
| 1mei | MOA | A | 600 |
| 1mrg | ADN | A | 300 |
| 1mrj | ADN | A | 300 |
| 1mrl | DOL | A | 300 |
| 1mrl | DOL | B | 301 |
| 1mrl | DOL | C | 302 |
| 1mrq | STR | A | 501 |
| 1mui | AB1 | B | 100 |
| 1muo | ADN | A | 1 |
| 1mx1 | THA | A | 1 |
| 1mx1 | THA | B | 2 |
| 1mx1 | THA | C | 3 |
| 1mx1 | THA | D | 4 |
| 1mx1 | THA | E | 5 |
| 1mx1 | THA | F | 6 |
| 1n0s | FLU | A | 500 |
| 1n0s | FLU | B | 501 |
| 1n3z | ADN | A | 126 |
| 1n49 | RIT | B | 301 |
| 1n49 | RIT | D | 401 |
| 1nd4 | KAN | A | 1300 |
| 1nd4 | KAN | B | 2300 |
| 1nhz | 486 | A | 800 |
| 1nki | PPF | A | 5001 |
| 1nki | PPF | B | 5002 |
| 1nnc | ZMR | A | 479 |
| 1nnf | EDT | A | 400 |
| 1nr6 | DIF | A | 501 |
| 1nx9 | AIC | A | 5001 |
| 1nx9 | AIC | B | 5002 |
| 1nx9 | AIC | C | 5003 |
| 1nx9 | AIC | D | 5004 |
| 1o86 | LPR | A | 702 |
| 1oay | FUR | H | 500 |
| 1oay | FUR | J | 500 |
| 1odi | ADN | A | 1237 |
| 1odi | ADN | B | 1237 |
| 1odi | ADN | C | 1238 |
| 1odi | ADN | D | 1237 |
| 1odi | ADN | E | 1237 |
| 1odi | ADN | F | 1238 |
| 1ohr | 1UN | A | 201 |
| 1opj | STI | A | 3 |
| 1opj | STI | B | 4 |
| 1oq5 | CEL | A | 701 |
| 1os2 | HAE | A | 874 |
| 1os2 | HAE | D | 574 |
| 1ot7 | IU5 | B | 1002 |
| 1oxr | AIN | A | 141 |
| 1oye | CPF | A | 5001 |
| 1p2y | NCT | A | 440 |
| 1p5z | AR3 | B | 304 |
| 1p62 | GEO | B | 302 |
| 1p6k | MTL | A | 870 |
| 1p6k | MTL | B | 871 |
| 1p7r | NCT | A | 440 |
| 1p93 | DEX | A | 1999 |
| 1p93 | DEX | B | 2999 |
| 1p93 | DEX | C | 3999 |
| 1p93 | DEX | D | 4999 |
| 1pb9 | 4AX | A | 901 |
| 1pbc | BHA | A | 396 |
| 1pbf | BHA | A | 396 |
| 1pbk | RAP | A | 225 |
| 1pcg | EST | A | 1 |
| 1pcg | EST | B | 2 |
| 1pg2 | ADN | A | 552 |
| 1phg | MYT | A | 422 |
| 1pk7 | ADN | A | 1245 |
| 1pk7 | ADN | B | 1246 |
| 1pk7 | ADN | C | 1247 |
| 1pth | SAL | A | 710 |
| 1pth | SAL | B | 711 |
| 1pw7 | RAB | A | 645 |
| 1pw7 | RAB | B | 646 |
| 1pw7 | RAB | C | 647 |
| 1pwy | AC2 | E | 290 |
| 1pxx | DIF | A | 701 |
| 1pxx | DIF | B | 1701 |
| 1pxx | DIF | C | 2701 |
| 1pxx | DIF | D | 3701 |
| 1q0y | MOI | H | 401 |
| 1q23 | FUA | A | 702 |
| 1q23 | FUA | B | 703 |
| 1q23 | FUA | C | 701 |
| 1q23 | FUA | D | 705 |
| 1q23 | FUA | E | 706 |
| 1q23 | FUA | F | 704 |
| 1q23 | FUA | G | 708 |
| 1q23 | FUA | H | 709 |
| 1q23 | FUA | I | 707 |
| 1q23 | FUA | J | 711 |
| 1q23 | FUA | K | 712 |
| 1q23 | FUA | L | 710 |
| 1q6i | FK5 | A | 301 |
| 1q6i | FK5 | B | 401 |
| 1q97 | ADN | B | 486 |
| 1qca | FUA | A | 221 |
| 1qhs | CLM | A | 888 |
| 1qhs | CLM | A | 999 |
| 1qhy | CLM | A | 888 |
| 1qhy | CLM | A | 999 |
| 1qkn | RAL | A | 600 |
| 1qkt | EST | A | 600 |
| 1qku | EST | A | 600 |
| 1qku | EST | B | 600 |
| 1qku | EST | C | 600 |
| 1qti | GNT | A | 600 |
| 1qvt | PRL | A | 311 |
| 1qvu | PRL | A | 196 |
| 1qzv | PQN | A | 2001 |
| 1qzv | PQN | B | 2002 |
| 1qzv | PQN | P | 6001 |
| 1qzv | PQN | Q | 6002 |
| 1r55 | 097 | A | 518 |
| 1r9o | FLP | A | 501 |
| 1rj6 | AZM | A | 400 |
| 1rj6 | AZM | B | 401 |
| 1rkw | PNT | A | 225 |
| 1rl8 | RIT | A | 9001 |
| 1rmt | ADN | A | 1501 |
| 1rmt | ADN | B | 1502 |
| 1rmt | ADN | C | 1503 |
| 1rmt | ADN | D | 1504 |
| 1rqj | RIS | A | 901 |
| 1rqj | RIS | B | 903 |
| 1rs6 | MTL | A | 870 |
| 1rs6 | MTL | B | 871 |
| 1rs7 | MTL | B | 871 |
| 1rts | D16 | A | 309 |
| 1rts | D16 | B | 409 |
| 1rv7 | AB1 | B | 1001 |
| 1rxc | URF | B | 2011 |
| 1rxc | URF | C | 2081 |
| 1rxc | URF | D | 2021 |
| 1rxc | URF | E | 2031 |
| 1rxc | URF | F | 2001 |
| 1rxc | URF | I | 2041 |
| 1rxc | URF | K | 2061 |
| 1rxc | URF | L | 2071 |
| 1s14 | NOV | A | 1300 |
| 1s14 | NOV | B | 2300 |
| 1s1u | NVP | A | 999 |
| 1s1x | NVP | A | 999 |
| 1s2a | IMN | A | 2001 |
| 1s2q | RAS | A | 601 |
| 1s2q | RAS | B | 601 |
| 1s9p | DES | A | 459 |
| 1s9p | DES | B | 459 |
| 1s9p | DES | C | 500 |
| 1s9p | DES | D | 600 |
| 1sa1 | POD | B | 700 |
| 1sa1 | POD | D | 701 |
| 1sdt | MK1 | B | 902 |
| 1sdu | MK1 | B | 902 |
| 1sdv | MK1 | B | 902 |
| 1sgu | MK1 | B | 2632 |
| 1sh9 | RIT | B | 301 |
| 1skx | RFP | A | 1 |
| 1sn0 | T44 | B | 602 |
| 1sn0 | T44 | C | 601 |
| 1sn5 | T3 | C | 601 |
| 1sn5 | T3 | D | 602 |
| 1sqn | NDR | A | 1001 |
| 1sqn | NDR | B | 2001 |
| 1sv9 | DIF | A | 701 |
| 1sxk | BHA | A | 251 |
| 1t3r | 017 | A | 1200 |
| 1t46 | STI | A | 3 |
| 1t66 | FLU | C | 301 |
| 1t66 | FLU | L | 300 |
| 1t69 | SHH | A | 379 |
| 1t7i | 017 | A | 200 |
| 1t7j | 478 | A | 200 |
| 1t9u | CPF | A | 5001 |
| 1t9u | CPF | A | 5002 |
| 1t9w | NFN | A | 6001 |
| 1t9w | NFN | A | 6002 |
| 1tbf | VIA | A | 501 |
| 1tco | FK5 | C | 509 |
| 1td7 | NFL | A | 2001 |
| 1tgm | AIN | A | 202 |
| 1th6 | OIN | A | 401 |
| 1tlm | MIL | A | 128 |
| 1tlm | MIL | B | 128 |
| 1tt6 | DES | A | 129 |
| 1tt6 | DES | B | 128 |
| 1tuf | AZ1 | A | 502 |
| 1tuf | AZ1 | B | 503 |
| 1tyl | TYL | C | 100 |
| 1tym | TYL | C | 100 |
| 1tz8 | DES | B | 128 |
| 1tz8 | DES | C | 129 |
| 1tz8 | DES | D | 128 |
| 1u65 | CP0 | A | 1000 |
| 1uay | ADN | A | 1001 |
| 1uay | ADN | B | 1002 |
| 1udt | VIA | A | 1000 |
| 1udu | CIA | A | 1003 |
| 1udu | CIA | B | 2003 |
| 1uho | VDN | A | 1000 |
| 1uob | PNN | A | 1311 |
| 1uof | PNN | A | 1312 |
| 1upf | URF | A | 999 |
| 1upf | URF | B | 999 |
| 1upf | URF | C | 999 |
| 1upf | URF | D | 999 |
| 1usq | CLM | A | 1143 |
| 1usq | CLM | B | 1143 |
| 1usq | CLM | C | 1143 |
| 1usq | CLM | D | 1142 |
| 1usq | CLM | E | 1143 |
| 1usq | CLM | F | 1144 |
| 1utt | HAE | A | 1265 |
| 1utz | HAE | A | 1267 |
| 1utz | HAE | B | 1266 |
| 1uw6 | NCT | A | 1208 |
| 1uw6 | NCT | B | 1206 |
| 1uw6 | NCT | C | 1206 |
| 1uw6 | NCT | D | 1208 |
| 1uw6 | NCT | E | 1206 |
| 1uw6 | NCT | F | 1208 |
| 1uw6 | NCT | G | 1206 |
| 1uw6 | NCT | H | 1206 |
| 1uw6 | NCT | I | 1206 |
| 1uw6 | NCT | J | 1206 |
| 1uw6 | NCT | K | 1206 |
| 1uw6 | NCT | L | 1206 |
| 1uw6 | NCT | M | 1208 |
| 1uw6 | NCT | N | 1208 |
| 1uw6 | NCT | O | 1206 |
| 1uw6 | NCT | P | 1206 |
| 1uw6 | NCT | Q | 1206 |
| 1uw6 | NCT | R | 1208 |
| 1uw6 | NCT | S | 1206 |
| 1uw6 | NCT | T | 1208 |
| 1uwh | BAX | A | 1723 |
| 1uwh | BAX | B | 1723 |
| 1uwj | BAX | A | 1723 |
| 1uwj | BAX | B | 1723 |
| 1uzf | MCO | A | 702 |
| 1v3e | ZMR | A | 1200 |
| 1v3e | ZMR | B | 2200 |
| 1v3q | 2DI | E | 290 |
| 1v8b | ADN | A | 502 |
| 1v8b | ADN | B | 1502 |
| 1v8b | ADN | C | 2502 |
| 1v8b | ADN | D | 3502 |
| 1vhw | ADN | A | 252 |
| 1vhw | ADN | B | 252 |
| 1vhw | ADN | C | 252 |
| 1vhw | ADN | D | 252 |
| 1vhw | ADN | E | 252 |
| 1vhw | ADN | F | 252 |
| 1vm1 | TAZ | A | 504 |
| 1vrt | NVP | A | 999 |
| 1w0f | STR | A | 1499 |
| 1w0g | MYT | A | 1499 |
| 1w3r | 2MN | A | 1198 |
| 1w6f | ISZ | A | 1276 |
| 1w6f | ISZ | B | 1276 |
| 1w6f | ISZ | C | 1276 |
| 1w6f | ISZ | D | 1276 |
| 1w6r | GNT | A | 1536 |
| 1w76 | GNT | A | 1538 |
| 1w76 | GNT | B | 1538 |
| 1wrk | TFP | A | 202 |
| 1wrk | TFP | A | 204 |
| 1wrk | TFP | B | 201 |
| 1wrk | TFP | B | 203 |
| 1wrl | TFP | A | 202 |
| 1wrl | TFP | A | 204 |
| 1wrl | TFP | B | 201 |
| 1wrl | TFP | B | 203 |
| 1wrl | TFP | C | 206 |
| 1wrl | TFP | C | 208 |
| 1wrl | TFP | D | 205 |
| 1wrl | TFP | D | 207 |
| 1wrl | TFP | E | 210 |
| 1wrl | TFP | E | 212 |
| 1wrl | TFP | F | 209 |
| 1wrl | TFP | F | 211 |
| 1wu8 | ADN | A | 500 |
| 1wu8 | ADN | B | 501 |
| 1wu8 | ADN | C | 502 |
| 1wyg | SAL | A | 4005 |
| 1x70 | 715 | A | 801 |
| 1x70 | 715 | B | 801 |
| 1x9q | FLU | A | 1341 |
| 1xbb | STI | A | 1 |
| 1xkk | FMM | A | 91 |
| 1xos | VIA | A | 1 |
| 1xot | VDN | A | 101 |
| 1xot | VDN | B | 102 |
| 1xoz | CIA | A | 501 |
| 1xp0 | VDN | A | 201 |
| 1xql | 4AX | A | 605 |
| 1xql | 4AX | B | 505 |
| 1xr3 | ISZ | A | 263 |
| 1xr3 | ISZ | B | 302 |
| 1xwf | ADN | A | 433 |
| 1xwf | ADN | B | 433 |
| 1xwf | ADN | C | 433 |
| 1xwf | ADN | D | 433 |
| 1xz1 | HLT | A | 2001 |
| 1xz3 | ICF | A | 201 |
| 1xzx | T3 | X | 500 |
| 1y0x | T44 | X | 500 |
| 1y4l | SVR | B | 301 |
| 1y7i | SAL | A | 501 |
| 1y7i | SAL | A | 502 |
| 1y7i | SAL | B | 503 |
| 1y8e | SVR | A | 501 |
| 1y8e | SVR | B | 502 |
| 1y93 | HAE | A | 301 |
| 1ya3 | STR | A | 1001 |
| 1ya3 | STR | B | 2001 |
| 1ya3 | STR | C | 3001 |
| 1ya4 | CTX | A | 11 |
| 1ya4 | CTX | A | 1 |
| 1ya4 | CTX | B | 1283 |
| 1ya4 | CTX | B | 2 |
| 1ya4 | CTX | C | 1383 |
| 1ya4 | CTX | C | 3 |
| 1yat | FK5 | A | 108 |
| 1yda | AZM | A | 264 |
| 1ydb | AZM | A | 264 |
| 1ydd | AZM | A | 264 |
| 1yhl | RIS | A | 1400 |
| 1yhm | AHD | A | 901 |
| 1yhm | AHD | B | 1901 |
| 1yhm | AHD | C | 2901 |
| 1yi4 | ADN | A | 306 |
| 1yki | NFZ | A | 1219 |
| 1yki | NFZ | B | 2219 |
| 1yki | NFZ | C | 3219 |
| 1yki | NFZ | D | 4219 |
| 1ymx | CFX | A | 1001 |
| 1ymx | CFX | B | 1002 |
| 1ynn | RFP | C | 1120 |
| 1yq7 | RIS | A | 901 |
| 1yv5 | RIS | A | 901 |
| 1yvm | TMG | A | 501 |
| 1z11 | 8MO | A | 501 |
| 1z11 | 8MO | B | 501 |
| 1z11 | 8MO | C | 501 |
| 1z11 | 8MO | D | 501 |
| 1z2b | VLB | C | 800 |
| 1z37 | ADN | A | 300 |
| 1z9h | IMN | A | 379 |
| 1z9h | IMN | B | 381 |
| 1z9h | IMN | C | 379 |
| 1z9h | IMN | D | 476 |
| 1z9y | FUN | A | 500 |
| 1zgf | TRU | A | 300 |
| 1zgy | BRL | A | 503 |
| 1zlq | EDT | A | 1513 |
| 1zlq | EDT | B | 1511 |
| 1zr8 | AJM | A | 201 |
| 1zsb | AZM | A | 264 |
| 1zw5 | ZOL | A | 901 |
| 1zz1 | SHH | A | 2452 |
| 1zz1 | SHH | B | 2552 |
| 1zz1 | SHH | C | 2652 |
| 1zz1 | SHH | D | 2752 |
| 1zzq | MTL | A | 870 |
| 1zzq | MTL | B | 871 |
| 1zzu | MTL | A | 870 |
| 1zzu | MTL | B | 871 |
| 2a1h | GBN | A | 502 |
| 2a1h | GBN | B | 501 |
| 2a3a | TEP | A | 1433 |
| 2a3a | TEP | A | 1434 |
| 2a3a | TEP | A | 1435 |
| 2a3a | TEP | A | 1436 |
| 2a3a | TEP | B | 2433 |
| 2a3a | TEP | B | 2434 |
| 2a3a | TEP | B | 2435 |
| 2a3a | TEP | B | 2436 |
| 2a3b | CFF | A | 1433 |
| 2a3b | CFF | A | 1434 |
| 2a3b | CFF | A | 1435 |
| 2a3b | CFF | B | 2433 |
| 2a3b | CFF | B | 2434 |
| 2a3b | CFF | B | 2435 |
| 2a3c | PNX | A | 1434 |
| 2a3c | PNX | A | 1435 |
| 2a3c | PNX | B | 2433 |
| 2a3c | PNX | B | 2434 |
| 2a3r | LDP | A | 297 |
| 2a3r | LDP | B | 297 |
| 2a68 | RBT | C | 8001 |
| 2a68 | RBT | M | 8002 |
| 2a69 | RPT | C | 8001 |
| 2a69 | RPT | M | 8002 |
| 2a7q | CFB | A | 328 |
| 2a8t | ADN | A | 252 |
| 2a8t | ADN | B | 252 |
| 2aa5 | STR | A | 301 |
| 2aa5 | STR | B | 302 |
| 2aa6 | STR | A | 401 |
| 2aa6 | STR | B | 402 |
| 2aax | PDN | A | 502 |
| 2aax | PDN | B | 503 |
| 2ab2 | SNL | A | 502 |
| 2ab2 | SNL | B | 503 |
| 2aba | STR | A | 1500 |
| 2ac7 | ADN | A | 1216 |
| 2ac7 | ADN | B | 1215 |
| 2ack | EDR | A | 999 |
| 2aof | FRD | C | 305 |
| 2aoh | FRD | C | 305 |
| 2aoi | FRD | C | 305 |
| 2aoj | FRD | C | 305 |
| 2aot | 2PM | A | 400 |
| 2aot | 2PM | B | 401 |
| 2aou | CQA | A | 402 |
| 2aou | CQA | A | 403 |
| 2aou | CQA | B | 400 |
| 2aou | CQA | B | 401 |
| 2aow | THA | A | 400 |
| 2aow | THA | B | 401 |
| 2aox | THA | A | 400 |
| 2aox | THA | B | 401 |
| 2aqu | DR7 | B | 300 |
| 2arm | OIN | A | 401 |
| 2avo | MK1 | B | 902 |
| 2avs | MK1 | B | 902 |
| 2avv | MK1 | A | 901 |
| 2avv | MK1 | E | 902 |
| 2axn | EDT | A | 737 |
| 2ayl | FLP | A | 1701 |
| 2ayl | FLP | B | 2701 |
| 2b0q | NMY | A | 305 |
| 2b17 | DIF | A | 701 |
| 2b60 | RIT | B | 100 |
| 2b7z | MK1 | B | 200 |
| 2b82 | ADN | A | 1001 |
| 2b82 | ADN | B | 1002 |
| 2b8j | ADN | B | 331 |
| 2bdm | TMI | A | 501 |
| 2bdm | TMI | A | 502 |
| 2bdm | TMI | A | 503 |
| 2bfm | TOP | A | 1290 |
| 2bfm | TOP | B | 1290 |
| 2bk4 | RAS | A | 601 |
| 2bk4 | RAS | B | 601 |
| 2bl9 | CP6 | A | 1240 |
| 2bla | CP6 | A | 1240 |
| 2bml | XED | B | 1319 |
| 2bnn | FCN | A | 1199 |
| 2bnn | FCN | B | 1199 |
| 2bpx | MK1 | B | 902 |
| 2bxc | P1Z | A | 2001 |
| 2bxc | P1Z | B | 2001 |
| 2bxd | RWF | A | 2001 |
| 2bxd | RWF | B | 2001 |
| 2bxe | 1FL | A | 2001 |
| 2bxe | 1FL | A | 2002 |
| 2bxe | 1FL | A | 2003 |
| 2bxe | 1FL | B | 2001 |
| 2bxe | 1FL | B | 2002 |
| 2bxe | 1FL | B | 2003 |
| 2bxf | DZP | A | 2001 |
| 2bxf | DZP | B | 2001 |
| 2bxg | IBP | A | 2001 |
| 2bxg | IBP | A | 2002 |
| 2bxg | IBP | B | 2001 |
| 2bxg | IBP | B | 2002 |
| 2bxk | IMN | A | 2001 |
| 2bxm | IMN | A | 2001 |
| 2bxm | IMN | A | 2002 |
| 2bxn | IDB | A | 2001 |
| 2bxn | IDB | A | 2002 |
| 2bxp | P1Z | A | 3001 |
| 2bxq | IMN | A | 2001 |
| 2bxq | IMN | A | 2003 |
| 2bxq | P1Z | A | 2002 |
| 2c49 | ADN | A | 1301 |
| 2c49 | ADN | B | 1301 |
| 2c6n | LPR | A | 705 |
| 2c6n | LPR | B | 705 |
| 2cbr | A80 | A | 201 |
| 2ceo | T44 | A | 1395 |
| 2ceo | T44 | B | 1395 |
| 2cml | ZMR | A | 1477 |
| 2cml | ZMR | A | 1478 |
| 2cml | ZMR | B | 2477 |
| 2cml | ZMR | B | 2478 |
| 2cml | ZMR | C | 3477 |
| 2cml | ZMR | C | 3478 |
| 2cml | ZMR | D | 4477 |
| 2cml | ZMR | D | 4478 |
| 2coi | GBN | A | 420 |
| 2coi | GBN | B | 420 |
| 2coj | GBN | A | 420 |
| 2coj | GBN | B | 420 |
| 2d06 | EST | A | 304 |
| 2d06 | EST | B | 305 |
| 2dcf | ACA | A | 501 |
| 2dcf | ACA | A | 502 |
| 2dg3 | RAP | A | 501 |
| 2dg4 | RAP | A | 501 |
| 2dg9 | RAP | A | 501 |
| 2dm6 | IMN | A | 1401 |
| 2doj | ADN | A | 2001 |
| 2dpz | TYL | A | 2001 |
| 2dr6 | DM2 | A | 2002 |
| 2drd | MIY | A | 2001 |
| 2e1q | SAL | A | 2006 |
| 2e1q | SAL | B | 3006 |
| 2e1q | SAL | C | 4006 |
| 2e1q | SAL | D | 5006 |
| 2e91 | ZOL | A | 901 |
| 2e91 | ZOL | B | 902 |
| 2efj | 37T | A | 502 |
| 2ej3 | GBN | A | 2414 |
| 2ej3 | GBN | B | 914 |
| 2ej3 | GBN | C | 1414 |
| 2ejf | ADN | A | 2001 |
| 2ejf | ADN | B | 2002 |
| 2ejg | ADN | A | 1501 |
| 2ejg | ADN | B | 1502 |
| 2eva | ADN | A | 498 |
| 2ex6 | AIC | A | 501 |
| 2ex9 | PNV | A | 501 |
| 2f0z | ZMR | A | 381 |
| 2f16 | BO2 | 2 | 1405 |
| 2f16 | BO2 | H | 1400 |
| 2f16 | BO2 | K | 1402 |
| 2f16 | BO2 | N | 1404 |
| 2f16 | BO2 | V | 1401 |
| 2f16 | BO2 | Y | 1403 |
| 2f38 | 15M | A | 325 |
| 2f80 | 017 | B | 301 |
| 2f81 | 017 | A | 302 |
| 2f89 | 210 | F | 9001 |
| 2f8c | ZOL | F | 9001 |
| 2f8g | 017 | B | 401 |
| 2f8z | ZOL | F | 5001 |
| 2f92 | AHD | F | 9001 |
| 2f94 | BFQ | F | 9001 |
| 2f9k | ZOL | F | 9001 |
| 2fke | FK5 | A | 108 |
| 2fn1 | SAL | A | 506 |
| 2fn1 | SAL | B | 503 |
| 2fqy | ADN | A | 400 |
| 2fu8 | MCO | A | 1410 |
| 2fu8 | MCO | B | 2410 |
| 2fum | MIX | A | 539 |
| 2fum | MIX | B | 1539 |
| 2fum | MIX | C | 2539 |
| 2fum | MIX | D | 3539 |
| 2fxd | DR7 | A | 102 |
| 2fxe | DR7 | A | 102 |
| 2geh | NHY | A | 300 |
| 2gl0 | ADN | A | 901 |
| 2gl0 | ADN | B | 902 |
| 2gl0 | ADN | C | 903 |
| 2gl0 | ADN | D | 904 |
| 2gl0 | ADN | E | 905 |
| 2gl0 | ADN | F | 906 |
| 2gqg | 1N1 | A | 501 |
| 2gqg | 1N1 | B | 502 |
| 2gss | EAA | A | 0 |
| 2gss | EAA | B | 0 |
| 2gvc | MMZ | A | 501 |
| 2gvc | MMZ | B | 501 |
| 2gvc | MMZ | D | 501 |
| 2gvc | MMZ | E | 501 |
| 2h42 | VIA | A | 901 |
| 2h42 | VIA | B | 902 |
| 2h42 | VIA | C | 903 |
| 2h4n | AZM | A | 264 |
| 2h77 | T3 | A | 1 |
| 2h79 | T3 | A | 1 |
| 2h9t | SVR | H | 301 |
| 2ha2 | SCK | A | 901 |
| 2ha2 | SCK | B | 951 |
| 2ha6 | SCK | A | 901 |
| 2ha6 | SCK | A | 902 |
| 2ha6 | SCK | B | 951 |
| 2ha6 | SCK | B | 952 |
| 2hcj | TAC | B | 888 |
| 2hdn | TAC | B | 1888 |
| 2hdn | TAC | D | 2888 |
| 2hdn | TAC | F | 3888 |
| 2hdn | TAC | H | 4888 |
| 2hdn | TAC | J | 5888 |
| 2hdn | TAC | L | 6888 |
| 2hnd | NVP | A | 999 |
| 2hny | NVP | A | 999 |
| 2hs1 | 017 | A | 201 |
| 2hs1 | 017 | B | 203 |
| 2hs2 | 017 | A | 201 |
| 2hs2 | 017 | B | 203 |
| 2htq | ZMR | A | 472 |
| 2hu6 | HAE | A | 269 |
| 2hw2 | RFP | A | 1200 |
| 2hyy | STI | A | 600 |
| 2hyy | STI | B | 600 |
| 2hyy | STI | C | 600 |
| 2hyy | STI | D | 600 |
| 2hzq | STR | A | 300 |
| 2i2z | SAL | A | 1100 |
| 2i30 | SAL | A | 1100 |
| 2i30 | SAL | A | 1200 |
| 2i6z | CPT | A | 300 |
| 2idw | 017 | B | 401 |
| 2ien | 017 | B | 402 |
| 2ieo | 017 | A | 402 |
| 2ij7 | TPF | A | 2472 |
| 2ij7 | TPF | B | 2470 |
| 2ij7 | TPF | C | 2471 |
| 2ij7 | TPF | D | 2473 |
| 2ij7 | TPF | F | 2474 |
| 2ipf | FFA | A | 3 |
| 2ipf | FFA | B | 4 |
| 2ipg | FFA | A | 3 |
| 2ipg | FFA | B | 4 |
| 2ipj | FFA | A | 325 |
| 2ipj | FFA | B | 326 |
| 2it4 | PPF | A | 500 |
| 2ito | IRE | A | 2020 |
| 2ity | IRE | A | 2020 |
| 2itz | IRE | A | 2021 |
| 2iyf | ERY | A | 1400 |
| 2iyf | ERY | B | 1399 |
| 2j0d | ERY | A | 1498 |
| 2j7x | EST | A | 1454 |
| 2j7y | E3O | A | 1454 |
| 2jap | J01 | A | 1249 |
| 2jap | J01 | B | 1249 |
| 2jap | J01 | C | 1249 |
| 2jap | J01 | D | 1249 |
| 2jc9 | ADN | A | 1497 |
| 2jc9 | ADN | A | 1498 |
| 2jfa | RAL | A | 600 |
| 2jfa | RAL | B | 600 |
| 2jih | 097 | A | 1001 |
| 2jih | 097 | B | 1001 |
| 2jj8 | AZZ | A | 1211 |
| 2jj8 | AZZ | B | 1211 |
| 2jj8 | AZZ | C | 1211 |
| 2jj8 | AZZ | D | 1211 |
| 2jkj | CLM | A | 1141 |
| 2jkj | CLM | B | 1141 |
| 2jkj | CLM | C | 1141 |
| 2jkj | CLM | D | 1142 |
| 2jkj | CLM | E | 1141 |
| 2jkj | CLM | F | 1143 |
| 2jkl | CLM | A | 1144 |
| 2jkl | CLM | B | 1144 |
| 2jkl | CLM | C | 1143 |
| 2jkl | CLM | D | 1145 |
| 2jkl | CLM | E | 1143 |
| 2jkl | CLM | F | 1143 |
| 2kce | D16 | A | 566 |
| 2kce | D16 | B | 568 |
| 2ki5 | AC2 | A | 1 |
| 2ki5 | AC2 | B | 2 |
| 2nmy | ROC | A | 401 |
| 2nmz | ROC | B | 401 |
| 2nni | MTK | A | 501 |
| 2nnj | 225 | A | 501 |
| 2nnk | ROC | A | 401 |
| 2nnp | ROC | A | 401 |
| 2no0 | GEO | A | 302 |
| 2no0 | GEO | B | 302 |
| 2no6 | ETV | A | 302 |
| 2no6 | ETV | B | 302 |
| 2noa | 3TC | A | 302 |
| 2noa | 3TC | B | 302 |
| 2nuv | 2TN | A | 2001 |
| 2nyr | SVR | B | 401 |
| 2o01 | PQN | A | 5001 |
| 2o01 | PQN | B | 5002 |
| 2o1o | RIS | A | 400 |
| 2o1o | RIS | B | 400 |
| 2o4k | DR7 | A | 301 |
| 2o4l | TPV | A | 403 |
| 2o4n | TPV | A | 300 |
| 2o4p | TPV | A | 300 |
| 2o4s | AB1 | A | 400 |
| 2o5y | STR | H | 249 |
| 2o7o | DXT | A | 222 |
| 2oa1 | ADN | B | 2005 |
| 2oax | SNL | A | 1001 |
| 2oax | SNL | B | 2001 |
| 2oax | SNL | C | 3001 |
| 2oax | SNL | D | 4001 |
| 2oax | SNL | E | 5001 |
| 2oax | SNL | F | 6001 |
| 2ocf | EST | A | 596 |
| 2ocu | TYL | A | 3001 |
| 2oiq | STI | A | 1001 |
| 2otf | 2TN | A | 201 |
| 2oth | IMN | A | 301 |
| 2oub | 2TN | A | 134 |
| 2ow9 | HAE | A | 502 |
| 2ow9 | HAE | B | 502 |
| 2ozr | HAE | C | 3001 |
| 2ozr | HAE | D | 3002 |
| 2ozr | HAE | E | 3003 |
| 2ozr | HAE | F | 3004 |
| 2pgf | ADN | A | 501 |
| 2pgr | DCF | A | 501 |
| 2ph9 | GNT | A | 301 |
| 2ph9 | GNT | C | 301 |
| 2ph9 | GNT | D | 301 |
| 2ph9 | GNT | E | 301 |
| 2piv | T3 | A | 933 |
| 2piw | T3 | A | 932 |
| 2piw | T3 | A | 933 |
| 2pk4 | ACA | A | 100 |
| 2pkm | ADN | A | 501 |
| 2pl0 | STI | A | 200 |
| 2pnc | CLU | A | 808 |
| 2pnc | CLU | B | 809 |
| 2pou | I7A | A | 1000 |
| 2pps | PQN | B | 2007 |
| 2prg | BRL | A | 1 |
| 2prg | BRL | B | 2 |
| 2pws | IBP | A | 3960 |
| 2pym | 1UN | A | 1001 |
| 2pyn | 1UN | A | 1001 |
| 2q1v | PDN | A | 248 |
| 2q58 | ZOL | A | 1 |
| 2q58 | ZOL | B | 2 |
| 2q5k | AB1 | A | 201 |
| 2q63 | 1UN | A | 1001 |
| 2q64 | 1UN | B | 1001 |
| 2q6h | CXX | A | 801 |
| 2q6h | CXX | A | 802 |
| 2q6k | ADN | A | 699 |
| 2q72 | IXX | A | 801 |
| 2q72 | IXX | A | 802 |
| 2q83 | ADN | A | 1 |
| 2q83 | ADN | B | 2 |
| 2qak | 1UN | A | 1001 |
| 2qb4 | DSM | A | 801 |
| 2qb4 | DSM | A | 802 |
| 2qds | MCO | A | 501 |
| 2qei | CXX | A | 801 |
| 2qei | CXX | A | 802 |
| 2qeo | LNR | A | 200 |
| 2qeo | LNR | B | 200 |
| 2qhc | AB1 | B | 9001 |
| 2qis | RIS | A | 901 |
| 2qju | DSM | A | 801 |
| 2qmz | LDP | A | 501 |
| 2qmz | LDP | B | 502 |
| 2qo3 | CER | A | 960 |
| 2qo3 | CER | B | 960 |
| 2qqt | AIN | A | 596 |
| 2que | AJM | A | 201 |
| 2qxs | RAL | A | 600 |
| 2qxs | RAL | B | 600 |
| 2r5p | MK1 | B | 902 |
| 2r5p | MK1 | D | 902 |
| 2r5q | 1UN | B | 900 |
| 2r5q | 1UN | D | 900 |
| 2rdd | AIC | A | 1109 |
| 2rdd | AIC | A | 1110 |
| 2riw | T44 | A | 1395 |
| 2rk8 | PPF | A | 3969 |
| 2rk8 | PPF | B | 3969 |
| 2rkf | AB1 | A | 501 |
| 2rkg | AB1 | B | 501 |
| 2rox | T44 | A | 128 |
| 2rox | T44 | B | 128 |
| 2trt | TAC | A | 222 |
| 2tsr | D16 | A | 309 |
| 2tsr | D16 | B | 409 |
| 2tsr | D16 | C | 509 |
| 2tsr | D16 | D | 609 |
| 2uxo | TAC | B | 1211 |
| 2uxp | CLM | A | 1211 |
| 2uxp | CLM | B | 1211 |
| 2uy4 | AZM | A | 1311 |
| 2v0z | C41 | C | 1328 |
| 2v0z | C41 | O | 1327 |
| 2v2e | ISZ | A | 1296 |
| 2v3d | NBV | A | 1503 |
| 2v3d | NBV | B | 1504 |
| 2v57 | PRL | A | 1188 |
| 2v57 | PRL | C | 1187 |
| 2vcf | ISZ | X | 1252 |
| 2vcf | ISZ | X | 1253 |
| 2vcn | ISZ | A | 1254 |
| 2vcn | ISZ | A | 1255 |
| 2vcn | ISZ | A | 1256 |
| 2vcs | ISZ | A | 1252 |
| 2vcs | ISZ | A | 1253 |
| 2vdb | NPS | A | 1591 |
| 2vdm | AGG | B | 1462 |
| 2vfu | MTL | A | 1419 |
| 2vin | 505 | A | 1247 |
| 2vke | TAC | A | 222 |
| 2vkz | CER | A | 2748 |
| 2vkz | CER | B | 2748 |
| 2vkz | CER | C | 2748 |
| 2vn1 | FK5 | A | 501 |
| 2vn1 | FK5 | B | 501 |
| 2vpp | GEO | A | 1210 |
| 2vpp | GEO | B | 1207 |
| 2vq5 | LDP | B | 1197 |
| 2vqy | PAR | A | 1201 |
| 2vrv | CL6 | A | 1410 |
| 2vuf | FUA | A | 2001 |
| 2vuf | FUA | A | 2002 |
| 2vuf | FUA | B | 2001 |
| 2vuf | FUA | B | 2002 |
| 2w03 | ADN | A | 1588 |
| 2w03 | ADN | B | 1588 |
| 2w26 | RIV | A | 1001 |
| 2w3a | TOP | A | 1190 |
| 2w3a | TOP | B | 1189 |
| 2w3v | TOP | A | 1169 |
| 2w4x | STZ | A | 1591 |
| 2w8y | 486 | A | 1000 |
| 2w8y | NDR | B | 1000 |
| 2w98 | P1Z | A | 1351 |
| 2w98 | P1Z | A | 1352 |
| 2w98 | P1Z | A | 1353 |
| 2w98 | P1Z | B | 1356 |
| 2w98 | P1Z | B | 1357 |
| 2w98 | P1Z | B | 1358 |
| 2w98 | P1Z | B | 1359 |
| 2w9g | TOP | A | 1159 |
| 2w9h | TOP | A | 1160 |
| 2w9s | TOP | A | 1160 |
| 2w9s | TOP | B | 1160 |
| 2w9s | TOP | C | 1160 |
| 2w9s | TOP | D | 1158 |
| 2w9s | TOP | E | 1160 |
| 2w9s | TOP | F | 1159 |
| 2wd9 | IBP | A | 1570 |
| 2wd9 | IBP | B | 1570 |
| 2wd9 | IBP | C | 1570 |
| 2wek | DIF | A | 1373 |
| 2wek | DIF | A | 1374 |
| 2wek | DIF | A | 1375 |
| 2wek | DIF | A | 1376 |
| 2wek | DIF | B | 1373 |
| 2wek | DIF | B | 1374 |
| 2wek | DIF | B | 1375 |
| 2wek | DIF | B | 1376 |
| 2wey | EV1 | A | 1771 |
| 2wey | EV1 | B | 1771 |
| 2wm3 | NFL | A | 1300 |
| 2wm3 | NFL | A | 1301 |
| 2wq5 | MIY | A | 1120 |
| 2wsc | PQN | A | 1802 |
| 2wsc | PQN | B | 1773 |
| 2wse | PQN | A | 1801 |
| 2wse | PQN | B | 1774 |
| 2wsf | PQN | A | 1802 |
| 2wsf | PQN | B | 1773 |
| 2wuz | TPF | A | 1460 |
| 2wuz | TPF | B | 1460 |
| 2wv2 | TPF | A | 1 |
| 2wx2 | TPF | A | 1460 |
| 2wx2 | TPF | B | 1460 |
| 2x0p | ADN | A | 1607 |
| 2x0y | X0T | A | 1625 |
| 2x0y | X0T | B | 1625 |
| 2x1l | ADN | A | 601 |
| 2x1l | ADN | B | 601 |
| 2x1l | ADN | C | 601 |
| 2x2n | X2N | A | 1480 |
| 2x2n | X2N | B | 1479 |
| 2x2n | X2N | C | 1479 |
| 2x2n | X2N | D | 1479 |
| 2x7h | PFN | A | 1372 |
| 2x7h | PFN | A | 1374 |
| 2x7h | PFN | A | 1375 |
| 2x7h | PFN | A | 1376 |
| 2x7h | PFN | B | 1372 |
| 2x7h | PFN | B | 1374 |
| 2x8o | OIN | A | 1314 |
| 2x8o | OIN | A | 1317 |
| 2x8p | OIN | A | 1313 |
| 2x8p | OIN | A | 1315 |
| 2x91 | LPR | A | 1615 |
| 2x9g | LYA | A | 1270 |
| 2x9g | LYA | B | 1270 |
| 2x9g | LYA | C | 1270 |
| 2x9g | LYA | D | 1270 |
| 2x9v | TMQ | A | 1270 |
| 2x9v | TMQ | B | 1270 |
| 2x9v | TMQ | C | 1270 |
| 2x9v | TMQ | D | 1270 |
| 2xat | CLM | A | 301 |
| 2xf3 | J01 | A | 500 |
| 2xf3 | J01 | A | 600 |
| 2xf3 | J01 | B | 500 |
| 2xf3 | J01 | B | 600 |
| 2xfh | CL6 | A | 1413 |
| 2xfh | CL6 | A | 1414 |
| 2xfq | RAS | A | 601 |
| 2xfq | RAS | B | 601 |
| 2xfs | J01 | A | 500 |
| 2xfs | J01 | A | 600 |
| 2xfs | J01 | B | 500 |
| 2xfs | J01 | B | 600 |
| 2xh9 | J01 | A | 1436 |
| 2xh9 | J01 | A | 1437 |
| 2xh9 | J01 | B | 1436 |
| 2xh9 | J01 | B | 1437 |
| 2xkw | P1B | A | 1478 |
| 2xkw | P1B | B | 1475 |
| 2xn3 | ID8 | A | 1356 |
| 2xn5 | FUN | A | 1356 |
| 2xn6 | T44 | A | 1370 |
| 2xn7 | T44 | A | 1355 |
| 2xpv | MIY | A | 1209 |
| 2xpw | OTC | A | 222 |
| 2xrl | DXT | A | 1211 |
| 2xtk | AZM | A | 1339 |
| 2xtk | AZM | B | 1339 |
| 2xud | DME | A | 1544 |
| 2y00 | Y00 | A | 601 |
| 2y00 | Y00 | B | 601 |
| 2y01 | Y00 | A | 601 |
| 2y01 | Y00 | B | 601 |
| 2y04 | 68H | A | 601 |
| 2y04 | 68H | B | 601 |
| 2y05 | RAL | A | 801 |
| 2y05 | RAL | A | 802 |
| 2y6o | 1N1 | A | 1892 |
| 2y7j | B49 | A | 1294 |
| 2y7j | B49 | B | 1294 |
| 2y7j | B49 | C | 1294 |
| 2y7j | B49 | D | 1294 |
| 2y7k | SAL | A | 1302 |
| 2y7k | SAL | A | 1303 |
| 2y7k | SAL | B | 1304 |
| 2y7k | SAL | B | 1305 |
| 2y7k | SAL | C | 1302 |
| 2y7k | SAL | D | 1300 |
| 2y7p | SAL | A | 1000 |
| 2y7p | SAL | A | 1001 |
| 2y7w | SAL | A | 1300 |
| 2y7w | SAL | B | 1300 |
| 2y7w | SAL | C | 1300 |
| 2y8d | DHL | A | 3630 |
| 2y8d | DHL | A | 3631 |
| 2ya7 | ZMR | A | 1776 |
| 2ya7 | ZMR | B | 1776 |
| 2ya7 | ZMR | C | 1776 |
| 2ya7 | ZMR | D | 1776 |
| 2ydo | ADN | A | 400 |
| 2yja | EST | B | 1550 |
| 2yk1 | NCT | H | 300 |
| 2yoe | FL7 | C | 1318 |
| 2z2p | DOL | A | 2002 |
| 2z2p | DOL | B | 2003 |
| 2z54 | AB1 | A | 200 |
| 2z71 | PNV | A | 903 |
| 2z71 | PNV | C | 904 |
| 2zb8 | IMN | A | 800 |
| 2zbu | ADN | A | 501 |
| 2zbu | ADN | B | 502 |
| 2zbu | ADN | C | 503 |
| 2zbu | ADN | D | 504 |
| 2zbv | ADN | A | 501 |
| 2zbv | ADN | B | 502 |
| 2zbv | ADN | C | 503 |
| 2zgw | ADN | A | 1301 |
| 2zgw | ADN | B | 1302 |
| 2zi9 | CL9 | A | 401 |
| 2zi9 | CL9 | B | 401 |
| 2zia | CL9 | A | 401 |
| 2zia | CL9 | B | 401 |
| 2zm7 | ACA | A | 501 |
| 2zm7 | ACA | A | 502 |
| 2zm8 | ACA | A | 511 |
| 2zm8 | ACA | A | 512 |
| 2zm9 | ACA | A | 501 |
| 2zm9 | ACA | A | 502 |
| 2zma | ACA | A | 501 |
| 2zma | ACA | A | 502 |
| 2zq9 | CLS | A | 11 |
| 2zqc | AZR | A | 301 |
| 2zva | 1N1 | A | 513 |
| 3a2q | ACA | A | 601 |
| 3a2q | ACA | A | 602 |
| 3a3y | OBN | A | 6000 |
| 3a65 | ACA | A | 601 |
| 3a66 | ACA | A | 601 |
| 3ads | IMN | A | 1 |
| 3ads | IMN | A | 2 |
| 3ads | IMN | B | 3 |
| 3adx | IMN | A | 2 |
| 3adx | IMN | B | 3 |
| 3ai8 | HNQ | A | 255 |
| 3ai8 | HNQ | B | 255 |
| 3aob | RFP | C | 2002 |
| 3aoc | ERY | C | 3402 |
| 3aod | MIY | A | 2001 |
| 3aod | RFP | C | 2002 |
| 3apv | TP0 | A | 190 |
| 3apv | TP0 | B | 190 |
| 3apw | DP0 | A | 190 |
| 3apw | DP0 | B | 190 |
| 3apx | Z80 | A | 190 |
| 3arq | DM5 | A | 606 |
| 3arr | PNX | A | 606 |
| 3arr | PNX | A | 607 |
| 3aru | PNX | A | 606 |
| 3aru | PNX | A | 607 |
| 3aru | PNX | A | 608 |
| 3ax7 | SAL | A | 1336 |
| 3ax7 | SAL | B | 1336 |
| 3ax9 | SAL | A | 1341 |
| 3ax9 | SAL | B | 1340 |
| 3axz | ADN | A | 401 |
| 3ay0 | ADN | A | 401 |
| 3ay0 | ADN | B | 402 |
| 3b0w | DGX | A | 1 |
| 3b0w | DGX | B | 1 |
| 3b2r | VDN | A | 1 |
| 3b2r | VDN | B | 1 |
| 3b6h | MXD | A | 551 |
| 3b6h | MXD | B | 551 |
| 3b7e | ZMR | A | 1001 |
| 3b7e | ZMR | B | 1002 |
| 3b9l | AZZ | A | 1009 |
| 3b9l | AZZ | A | 1010 |
| 3b9m | AZZ | A | 1009 |
| 3b9m | SAL | A | 1100 |
| 3b9m | SAL | A | 1200 |
| 3ba0 | HAE | A | 477 |
| 3bbt | FMM | B | 91 |
| 3bbt | FMM | D | 91 |
| 3bcr | AZZ | A | 940 |
| 3bf6 | SVR | H | 301 |
| 3bfc | IM2 | A | 301 |
| 3bfc | IM2 | B | 302 |
| 3bfc | IM2 | C | 303 |
| 3bfc | IM2 | D | 304 |
| 3bgd | PM6 | A | 301 |
| 3bgd | PM6 | A | 302 |
| 3bgd | PM6 | B | 301 |
| 3bgd | PM6 | B | 302 |
| 3bjw | SVR | A | 508 |
| 3bjw | SVR | B | 501 |
| 3bjw | SVR | B | 512 |
| 3bjw | SVR | C | 505 |
| 3bjw | SVR | C | 507 |
| 3bjw | SVR | E | 503 |
| 3bjw | SVR | E | 510 |
| 3bjw | SVR | F | 502 |
| 3bjw | SVR | F | 509 |
| 3bjw | SVR | G | 506 |
| 3bjw | SVR | H | 504 |
| 3bjw | SVR | H | 511 |
| 3bl1 | BL1 | A | 300 |
| 3bpx | SAL | A | 257 |
| 3bpx | SAL | B | 258 |
| 3bvb | 017 | B | 401 |
| 3c0z | SHH | A | 301 |
| 3c0z | SHH | B | 301 |
| 3c0z | SHH | C | 301 |
| 3c9j | 308 | B | 101 |
| 3caj | EZL | A | 265 |
| 3cbi | AJM | A | 203 |
| 3cbi | AJM | B | 202 |
| 3cbi | AJM | C | 201 |
| 3cbi | AJM | D | 204 |
| 3ce6 | ADN | A | 500 |
| 3ce6 | ADN | B | 500 |
| 3ce6 | ADN | C | 500 |
| 3ce6 | ADN | D | 500 |
| 3cfl | 5CH | A | 693 |
| 3cfq | DIF | A | 1 |
| 3cfq | DIF | B | 1 |
| 3ckz | ZMR | A | 469 |
| 3cla | CLM | A | 221 |
| 3clb | TMQ | A | 611 |
| 3clb | TMQ | B | 612 |
| 3clb | TMQ | C | 613 |
| 3clb | TMQ | D | 614 |
| 3cmf | PDN | A | 946 |
| 3cmf | PDN | B | 945 |
| 3cot | STR | A | 1501 |
| 3cot | STR | B | 1500 |
| 3cr4 | PNT | X | 101 |
| 3cr4 | PNT | X | 102 |
| 3cr5 | PNT | X | 94 |
| 3cr5 | PNT | X | 95 |
| 3cs8 | BRL | A | 503 |
| 3cs9 | NIL | A | 600 |
| 3cs9 | NIL | B | 600 |
| 3cs9 | NIL | C | 600 |
| 3cs9 | NIL | D | 600 |
| 3csj | CBL | B | 211 |
| 3cyw | 017 | A | 201 |
| 3cyx | ROC | A | 201 |
| 3czv | AZM | A | 263 |
| 3czv | AZM | B | 263 |
| 3d1x | ROC | A | 201 |
| 3d1y | ROC | A | 201 |
| 3d1z | 017 | B | 201 |
| 3d20 | 017 | A | 201 |
| 3d2t | 1FL | A | 502 |
| 3d2t | 1FL | B | 500 |
| 3d41 | FCN | A | 4001 |
| 3d4s | TIM | A | 401 |
| 3d90 | NOG | A | 1001 |
| 3d90 | NOG | B | 2001 |
| 3d91 | REM | A | 350 |
| 3d91 | REM | B | 350 |
| 3dc3 | AZM | A | 263 |
| 3dcw | EZL | A | 301 |
| 3dd0 | EZL | A | 301 |
| 3dd1 | CFF | A | 903 |
| 3dd1 | CFF | B | 903 |
| 3dds | CFF | A | 904 |
| 3dds | CFF | B | 903 |
| 3ddw | CFF | A | 903 |
| 3ddw | CFF | B | 903 |
| 3deu | SAL | A | 301 |
| 3deu | SAL | A | 303 |
| 3deu | SAL | A | 305 |
| 3deu | SAL | B | 302 |
| 3deu | SAL | B | 304 |
| 3deu | SAL | B | 306 |
| 3dgq | EAA | A | 211 |
| 3dji | TYL | B | 613 |
| 3dji | TYL | B | 614 |
| 3dpf | HAE | A | 3 |
| 3dye | LNR | A | 600 |
| 3e22 | LOC | B | 700 |
| 3e22 | LOC | D | 700 |
| 3e4e | 4PZ | A | 501 |
| 3e4e | 4PZ | B | 501 |
| 3eau | PDN | A | 501 |
| 3eau | PDN | A | 601 |
| 3eb3 | PDN | A | 601 |
| 3eb4 | PDN | A | 501 |
| 3ebz | 017 | B | 201 |
| 3ekp | 478 | B | 200 |
| 3ekp | 478 | C | 200 |
| 3ekq | ROC | A | 100 |
| 3ekt | 017 | B | 200 |
| 3ekt | 017 | D | 200 |
| 3ekv | 478 | A | 200 |
| 3ekw | DR7 | B | 100 |
| 3ekx | 1UN | B | 201 |
| 3eky | DR7 | A | 100 |
| 3el0 | 1UN | A | 201 |
| 3el1 | DR7 | A | 100 |
| 3el4 | ROC | A | 100 |
| 3el5 | 1UN | B | 201 |
| 3el9 | DR7 | A | 100 |
| 3em3 | 478 | B | 200 |
| 3em4 | DR7 | A | 100 |
| 3em4 | DR7 | V | 100 |
| 3em6 | 017 | A | 200 |
| 3eor | CFV | A | 901 |
| 3erd | DES | A | 600 |
| 3erd | DES | B | 800 |
| 3ete | H3P | A | 552 |
| 3ete | H3P | B | 552 |
| 3ete | H3P | C | 552 |
| 3ete | H3P | C | 554 |
| 3ete | H3P | D | 552 |
| 3ete | H3P | F | 552 |
| 3ez3 | ZOL | A | 397 |
| 3ez3 | ZOL | B | 397 |
| 3ez3 | ZOL | C | 397 |
| 3ez3 | ZOL | D | 397 |
| 3f33 | PFL | A | 2001 |
| 3f4x | KLT | A | 300 |
| 3f78 | ICF | A | 1 |
| 3f78 | ICF | B | 2 |
| 3f8f | DM1 | A | 127 |
| 3f8w | ADN | A | 300 |
| 3f8w | ADN | B | 301 |
| 3f8w | ADN | C | 302 |
| 3fg5 | AJM | A | 134 |
| 3fl9 | TOP | A | 200 |
| 3fl9 | TOP | B | 200 |
| 3fl9 | TOP | C | 200 |
| 3fl9 | TOP | D | 200 |
| 3fl9 | TOP | E | 200 |
| 3fl9 | TOP | F | 200 |
| 3fl9 | TOP | G | 200 |
| 3fl9 | TOP | H | 200 |
| 3fo7 | IMN | A | 301 |
| 3frb | TOP | X | 300 |
| 3fre | TOP | X | 300 |
| 3frq | ERY | A | 195 |
| 3frq | ERY | B | 195 |
| 3fuu | ADN | A | 0 |
| 3fw1 | STI | A | 233 |
| 3fw3 | ETS | A | 302 |
| 3fw3 | ETS | B | 303 |
| 3g0e | B49 | A | 9000 |
| 3g0f | B49 | A | 9001 |
| 3g0f | B49 | B | 9001 |
| 3g1r | FIT | B | 327 |
| 3g1u | ADN | A | 438 |
| 3g1u | ADN | B | 438 |
| 3g1u | ADN | C | 438 |
| 3g1u | ADN | D | 438 |
| 3g5d | 1N1 | A | 1 |
| 3g5d | 1N1 | B | 1 |
| 3g6m | CFF | A | 1 |
| 3g6m | CFF | A | 427 |
| 3gan | SVR | A | 158 |
| 3gan | SVR | A | 159 |
| 3gcl | AIN | A | 609 |
| 3gcs | BAX | A | 401 |
| 3gf2 | SAL | A | 147 |
| 3ggu | 017 | B | 201 |
| 3gkz | B40 | A | 500 |
| 3glq | RAB | A | 602 |
| 3glq | RAB | B | 602 |
| 3gn8 | DEX | A | 247 |
| 3gn8 | DEX | B | 247 |
| 3gp0 | NIL | A | 1 |
| 3grv | ADN | A | 300 |
| 3gss | EAA | A | 212 |
| 3gss | EAA | B | 211 |
| 3gvu | STI | A | 1001 |
| 3gvu | STI | A | 1002 |
| 3gws | T3 | X | 500 |
| 3gwu | SRE | A | 801 |
| 3gwv | RFX | A | 801 |
| 3gy3 | PNT | A | 246 |
| 3h0a | 9RA | A | 500 |
| 3h1x | IMN | A | 301 |
| 3h52 | 486 | A | 3 |
| 3h52 | 486 | B | 1 |
| 3h52 | 486 | C | 4 |
| 3h52 | 486 | D | 2 |
| 3h6t | CYZ | A | 265 |
| 3h6t | CYZ | B | 265 |
| 3h6t | CYZ | C | 265 |
| 3h9u | ADN | A | 439 |
| 3h9u | ADN | B | 438 |
| 3h9u | ADN | C | 438 |
| 3h9u | ADN | D | 438 |
| 3hav | SRY | A | 403 |
| 3hav | SRY | B | 403 |
| 3hav | SRY | C | 403 |
| 3hbb | TMQ | A | 611 |
| 3hbb | TMQ | B | 612 |
| 3hbb | TMQ | C | 613 |
| 3hbb | TMQ | D | 614 |
| 3hcd | LNR | A | 2001 |
| 3hcd | LNR | B | 2002 |
| 3hec | STI | A | 1 |
| 3heg | BAX | A | 1 |
| 3hgx | SAL | A | 102 |
| 3hgx | SAL | B | 104 |
| 3hii | PNT | A | 901 |
| 3hii | PNT | B | 901 |
| 3hjo | EAA | A | 211 |
| 3hjo | EAA | B | 211 |
| 3hku | TOR | A | 300 |
| 3hlw | CE3 | A | 301 |
| 3hlw | CE3 | A | 303 |
| 3hlw | CE3 | B | 302 |
| 3hlw | CE3 | B | 304 |
| 3hm1 | J3Z | A | 2 |
| 3hm1 | J3Z | B | 1 |
| 3hmj | CER | A | 2748 |
| 3hmj | CER | B | 2748 |
| 3hmj | CER | C | 2748 |
| 3hp1 | LLT | A | 401 |
| 3hs4 | AZM | A | 701 |
| 3hs4 | AZM | A | 702 |
| 3hs4 | AZM | A | 703 |
| 3hth | PRL | A | 201 |
| 3hth | PRL | B | 201 |
| 3huo | PNN | A | 300 |
| 3huo | PNN | A | 302 |
| 3huo | PNN | A | 303 |
| 3huo | PNN | A | 304 |
| 3huo | PNN | B | 301 |
| 3hvt | NVP | A | 557 |
| 3hy7 | 097 | A | 801 |
| 3hy7 | 097 | B | 801 |
| 3i6n | ISZ | A | 607 |
| 3iai | AZM | A | 263 |
| 3iai | AZM | B | 263 |
| 3iai | AZM | C | 263 |
| 3iai | AZM | D | 263 |
| 3iak | EV1 | A | 415 |
| 3iaz | AIN | A | 1202 |
| 3ib0 | DIF | A | 701 |
| 3ib1 | IMN | A | 701 |
| 3ib2 | IBP | A | 3960 |
| 3iba | ZOL | A | 901 |
| 3ihz | FK5 | A | 501 |
| 3ijx | HCZ | B | 800 |
| 3ijx | HCZ | D | 800 |
| 3ijx | HCZ | H | 800 |
| 3ik6 | HCZ | B | 262 |
| 3ik6 | HCZ | B | 800 |
| 3ik6 | HCZ | H | 800 |
| 3ilt | TRU | B | 800 |
| 3ilt | TRU | E | 800 |
| 3ilt | TRU | H | 800 |
| 3ilu | HFZ | B | 800 |
| 3ilu | HFZ | E | 800 |
| 3ilu | HFZ | H | 800 |
| 3ita | AIC | D | 501 |
| 3iwx | CPT | A | 69 |
| 3jq7 | DX2 | A | 271 |
| 3jq7 | DX2 | B | 271 |
| 3jq7 | DX2 | C | 270 |
| 3jq7 | DX2 | D | 270 |
| 3jqa | DX4 | A | 270 |
| 3jqa | DX4 | B | 270 |
| 3jqa | DX4 | C | 270 |
| 3jqa | DX4 | D | 270 |
| 3jqz | LQZ | A | 586 |
| 3jus | ECL | A | 600 |
| 3jus | ECL | B | 600 |
| 3jvy | 017 | B | 401 |
| 3jw2 | 017 | A | 401 |
| 3jw3 | TOP | A | 208 |
| 3jw3 | TOP | B | 208 |
| 3jw5 | TOP | A | 208 |
| 3jw5 | TOP | B | 208 |
| 3jwq | VIA | A | 901 |
| 3jwq | VIA | B | 901 |
| 3jwq | VIA | C | 901 |
| 3jwq | VIA | D | 901 |
| 3jz0 | CLY | A | 900 |
| 3jz0 | CLY | B | 900 |
| 3k2h | LYA | A | 513 |
| 3k2h | LYA | A | 514 |
| 3k2h | LYA | B | 513 |
| 3k2h | LYA | B | 514 |
| 3k4v | ROC | B | 201 |
| 3k4v | ROC | D | 201 |
| 3k54 | 1N1 | A | 1 |
| 3k5v | STI | A | 2 |
| 3k5v | STI | B | 2 |
| 3kec | HAE | A | 272 |
| 3kec | HAE | B | 271 |
| 3khm | TPF | A | 501 |
| 3kiv | ACA | A | 100 |
| 3kk6 | CEL | A | 701 |
| 3kk6 | CEL | B | 1701 |
| 3km6 | EAA | A | 222 |
| 3km6 | EAA | B | 222 |
| 3kmo | EAA | A | 214 |
| 3kmo | EAA | B | 213 |
| 3ko0 | TFP | A | 201 |
| 3ko0 | TFP | A | 202 |
| 3ko0 | TFP | B | 201 |
| 3ko0 | TFP | B | 202 |
| 3ko0 | TFP | C | 201 |
| 3ko0 | TFP | C | 202 |
| 3ko0 | TFP | D | 201 |
| 3ko0 | TFP | D | 202 |
| 3ko0 | TFP | E | 201 |
| 3ko0 | TFP | E | 202 |
| 3ko0 | TFP | F | 201 |
| 3ko0 | TFP | F | 202 |
| 3ko0 | TFP | G | 201 |
| 3ko0 | TFP | G | 202 |
| 3ko0 | TFP | H | 201 |
| 3ko0 | TFP | H | 202 |
| 3ko0 | TFP | I | 201 |
| 3ko0 | TFP | I | 202 |
| 3ko0 | TFP | J | 201 |
| 3ko0 | TFP | J | 202 |
| 3ko0 | TFP | K | 201 |
| 3ko0 | TFP | K | 202 |
| 3ko0 | TFP | L | 201 |
| 3ko0 | TFP | L | 202 |
| 3ko0 | TFP | M | 201 |
| 3ko0 | TFP | M | 202 |
| 3ko0 | TFP | N | 201 |
| 3ko0 | TFP | N | 202 |
| 3ko0 | TFP | O | 201 |
| 3ko0 | TFP | O | 202 |
| 3ko0 | TFP | P | 201 |
| 3ko0 | TFP | P | 202 |
| 3ko0 | TFP | Q | 201 |
| 3ko0 | TFP | Q | 202 |
| 3ko0 | TFP | R | 201 |
| 3ko0 | TFP | R | 202 |
| 3ko0 | TFP | S | 201 |
| 3ko0 | TFP | S | 202 |
| 3ko0 | TFP | T | 201 |
| 3ko0 | TFP | T | 202 |
| 3kp2 | PNN | A | 5001 |
| 3kp2 | PNN | B | 5002 |
| 3kp3 | AIC | B | 2001 |
| 3kp3 | AIC | B | 2002 |
| 3kp4 | MII | A | 2001 |
| 3kp4 | MII | B | 2002 |
| 3kp5 | KAN | A | 2001 |
| 3kp5 | KAN | B | 2002 |
| 3kp6 | SAL | A | 3002 |
| 3kp6 | SAL | A | 3005 |
| 3kp6 | SAL | B | 3001 |
| 3kp6 | SAL | B | 3003 |
| 3kp6 | SAL | B | 3004 |
| 3kp6 | SAL | B | 3006 |
| 3kp6 | SAL | B | 3007 |
| 3kp6 | SAL | B | 3008 |
| 3kvr | URF | A | 2001 |
| 3kvr | URF | B | 2011 |
| 3kvv | URF | A | 254 |
| 3kvv | URF | B | 254 |
| 3kvv | URF | C | 254 |
| 3kvv | URF | D | 254 |
| 3kvv | URF | E | 254 |
| 3kvv | URF | F | 254 |
| 3kw2 | ADN | A | 300 |
| 3kw2 | ADN | B | 300 |
| 3kw4 | TIC | A | 600 |
| 3kz7 | RAP | A | 225 |
| 3l4d | TPF | A | 490 |
| 3l4d | TPF | B | 490 |
| 3l4d | TPF | C | 490 |
| 3l4d | TPF | D | 490 |
| 3l4w | MIG | A | 1001 |
| 3l7k | EDT | A | 739 |
| 3l7k | EDT | D | 735 |
| 3l7m | EDT | A | 738 |
| 3l7m | EDT | D | 735 |
| 3ldw | ZOL | A | 397 |
| 3ldw | ZOL | B | 397 |
| 3ldw | ZOL | C | 397 |
| 3ldw | ZOL | D | 397 |
| 3lfa | 1N1 | A | 361 |
| 3lik | HAE | A | 302 |
| 3lil | HAE | A | 302 |
| 3ljg | HAE | A | 301 |
| 3lk0 | Z80 | B | 92 |
| 3lk0 | Z80 | D | 92 |
| 3lka | HAE | A | 269 |
| 3lmy | CP6 | A | 562 |
| 3lmy | CP6 | B | 563 |
| 3ln1 | CEL | A | 682 |
| 3ln1 | CEL | B | 682 |
| 3ln1 | CEL | C | 682 |
| 3ln1 | CEL | D | 682 |
| 3lp0 | NVP | A | 701 |
| 3lp1 | NVP | A | 701 |
| 3lps | NOV | A | 901 |
| 3ls4 | TCI | H | 220 |
| 3lt5 | BLQ | A | 215 |
| 3lt5 | BLQ | A | 216 |
| 3ltw | HLZ | A | 300 |
| 3ltw | HLZ | A | 302 |
| 3lus | MCO | B | 1001 |
| 3lw5 | PQN | A | 5001 |
| 3lw5 | PQN | B | 5002 |
| 3lxe | TOR | A | 262 |
| 3lxe | TOR | B | 262 |
| 3lzs | 017 | A | 200 |
| 3lzu | 017 | A | 200 |
| 3lzv | 017 | A | 200 |
| 3m0w | P77 | A | 203 |
| 3m0w | P77 | B | 203 |
| 3m0w | P77 | B | 204 |
| 3m0w | P77 | C | 203 |
| 3m0w | P77 | D | 203 |
| 3m0w | P77 | E | 203 |
| 3m0w | P77 | F | 203 |
| 3m0w | P77 | G | 203 |
| 3m0w | P77 | H | 203 |
| 3m0w | P77 | I | 203 |
| 3m0w | P77 | I | 204 |
| 3m0w | P77 | J | 203 |
| 3m6b | 1RG | A | 308 |
| 3m6b | 1RG | A | 309 |
| 3mdt | VOR | A | 506 |
| 3mdt | VOR | B | 506 |
| 3mdv | CL6 | A | 506 |
| 3mdv | CL6 | B | 506 |
| 3mdz | EZL | A | 264 |
| 3mes | DME | A | 427 |
| 3mes | DME | B | 427 |
| 3mg0 | BO2 | 2 | 1405 |
| 3mg0 | BO2 | H | 1400 |
| 3mg0 | BO2 | K | 1402 |
| 3mg0 | BO2 | N | 1404 |
| 3mg0 | BO2 | V | 1401 |
| 3mg0 | BO2 | Y | 1403 |
| 3miy | B49 | A | 1 |
| 3miy | B49 | B | 2 |
| 3mjr | AC2 | A | 301 |
| 3mjr | AC2 | B | 401 |
| 3mjr | AC2 | D | 601 |
| 3ml5 | AZM | A | 264 |
| 3mne | DEX | A | 784 |
| 3mno | DEX | A | 784 |
| 3mnp | DEX | A | 784 |
| 3ms9 | STI | A | 1 |
| 3ms9 | STI | B | 1 |
| 3mss | STI | A | 1 |
| 3mss | STI | B | 1 |
| 3mss | STI | C | 1 |
| 3mss | STI | D | 1 |
| 3mws | 017 | B | 201 |
| 3mze | CFX | A | 364 |
| 3mzf | IM2 | A | 364 |
| 3n0h | TOP | A | 187 |
| 3n0h | TOP | A | 191 |
| 3n23 | OBN | A | 1 |
| 3n23 | OBN | C | 1 |
| 3n3i | ROC | A | 201 |
| 3n45 | ZOL | F | 354 |
| 3n46 | ZOL | F | 354 |
| 3n58 | ADN | A | 500 |
| 3n58 | ADN | C | 500 |
| 3n58 | ADN | D | 500 |
| 3n62 | MTL | A | 870 |
| 3n62 | MTL | B | 870 |
| 3n65 | MTL | B | 870 |
| 3n66 | MTL | B | 870 |
| 3n7w | AXL | A | 1 |
| 3n8w | FLP | A | 701 |
| 3n8y | DIF | A | 701 |
| 3n8y | DIF | B | 585 |
| 3n8y | SAL | B | 900 |
| 3n8z | FLP | A | 701 |
| 3n8z | FLP | B | 1701 |
| 3n9j | EAA | A | 210 |
| 3n9j | EAA | B | 214 |
| 3nai | URF | A | 521 |
| 3nai | URF | B | 521 |
| 3nai | URF | C | 521 |
| 3nbl | DXF | A | 1 |
| 3nbq | URF | A | 400 |
| 3nbq | URF | B | 400 |
| 3nbq | URF | C | 400 |
| 3nbq | URF | D | 400 |
| 3ndt | ROC | A | 101 |
| 3ndt | ROC | D | 100 |
| 3ndu | ROC | A | 101 |
| 3ndu | ROC | D | 100 |
| 3ndv | AIC | A | 375 |
| 3ndv | AIC | B | 376 |
| 3ndv | AIC | C | 375 |
| 3ndv | AIC | D | 374 |
| 3ndw | RIT | A | 100 |
| 3ndx | RIT | A | 100 |
| 3njz | SAL | A | 370 |
| 3nk2 | LDP | X | 433 |
| 3nrr | D16 | A | 520 |
| 3nrr | D16 | A | 530 |
| 3nrr | D16 | B | 520 |
| 3nrr | D16 | B | 530 |
| 3ns1 | PM6 | C | 1 |
| 3ns1 | PM6 | L | 1 |
| 3nsr | URF | A | 5 |
| 3nsr | URF | B | 1 |
| 3nsr | URF | B | 7 |
| 3nsr | URF | C | 4 |
| 3nsr | URF | D | 2 |
| 3nsr | URF | E | 6 |
| 3nsr | URF | F | 8 |
| 3nsr | URF | H | 3 |
| 3nt1 | NPS | A | 5 |
| 3nt1 | NPS | B | 4 |
| 3nu3 | 478 | B | 401 |
| 3nu4 | 478 | B | 401 |
| 3nu5 | 478 | B | 401 |
| 3nu6 | 478 | B | 401 |
| 3nu9 | 478 | A | 401 |
| 3nuj | 478 | B | 401 |
| 3nuo | 478 | B | 478 |
| 3nvc | SAL | A | 370 |
| 3nxu | RIT | A | 600 |
| 3nxu | RIT | B | 600 |
| 3ny4 | SMX | A | 308 |
| 3ny4 | SMX | A | 309 |
| 3ny4 | SMX | A | 310 |
| 3ny4 | SMX | A | 311 |
| 3ny4 | SMX | A | 312 |
| 3o0q | ADN | A | 1004 |
| 3o0q | ADN | B | 1004 |
| 3o1c | ADN | A | 127 |
| 3o1x | ADN | A | 1450 |
| 3o5r | FK5 | A | 1001 |
| 3oct | 1N1 | A | 663 |
| 3oez | STI | A | 601 |
| 3oez | STI | B | 601 |
| 3ogp | 017 | A | 200 |
| 3ogp | 017 | B | 200 |
| 3ogq | AB1 | A | 200 |
| 3ogw | IMN | A | 597 |
| 3oht | 1N1 | A | 1000 |
| 3oht | 1N1 | A | 2000 |
| 3oht | 1N1 | B | 1000 |
| 3oht | 1N1 | B | 2000 |
| 3oi8 | ADN | A | 2 |
| 3oi8 | ADN | B | 1 |
| 3oll | EST | A | 600 |
| 3oll | EST | B | 600 |
| 3ols | EST | A | 600 |
| 3ols | EST | B | 600 |
| 3ond | ADN | A | 506 |
| 3ond | ADN | B | 507 |
| 3osh | OIN | A | 5811 |
| 3owx | XRA | A | 233 |
| 3owx | XRA | B | 233 |
| 3oxc | ROC | A | 401 |
| 3oxv | 478 | A | 200 |
| 3oxv | 478 | B | 200 |
| 3oxv | 478 | C | 200 |
| 3oxw | 017 | B | 200 |
| 3oxw | 017 | D | 200 |
| 3oxx | DR7 | A | 100 |
| 3oxx | DR7 | C | 100 |
| 3oy4 | 017 | B | 200 |
| 3ozk | T44 | A | 128 |
| 3ozk | T44 | B | 128 |
| 3p4w | DSF | A | 319 |
| 3p4w | DSF | B | 319 |
| 3p4w | DSF | C | 320 |
| 3p4w | DSF | D | 319 |
| 3p4w | DSF | E | 319 |
| 3p50 | PFL | A | 319 |
| 3p50 | PFL | B | 319 |
| 3p50 | PFL | C | 319 |
| 3p50 | PFL | D | 320 |
| 3p50 | PFL | E | 319 |
| 3p6h | IBP | A | 133 |
| 3pbq | IM2 | A | 999 |
| 3pbs | AZR | A | 999 |
| 3pcq | PQN | A | 847 |
| 3pcq | PQN | B | 842 |
| 3peo | CU9 | A | 301 |
| 3peo | CU9 | B | 301 |
| 3peo | CU9 | C | 301 |
| 3peo | CU9 | D | 301 |
| 3peo | CU9 | F | 220 |
| 3peo | CU9 | F | 301 |
| 3peo | CU9 | H | 301 |
| 3peo | CU9 | J | 301 |
| 3pgh | FLP | A | 701 |
| 3pgh | FLP | B | 701 |
| 3pgh | FLP | C | 701 |
| 3pgh | FLP | D | 701 |
| 3pgl | RZX | A | 257 |
| 3po7 | ZON | A | 601 |
| 3po7 | ZON | B | 601 |
| 3pp7 | SVR | B | 499 |
| 3prs | RIT | A | 1001 |
| 3pwm | 017 | B | 402 |
| 3pwr | ROC | A | 401 |
| 3pww | ROC | A | 1001 |
| 3py4 | TYL | A | 598 |
| 3pyy | STI | A | 3 |
| 3pyy | STI | B | 4 |
| 3q07 | WPP | A | 300 |
| 3q07 | WPP | B | 400 |
| 3q1e | T44 | C | 128 |
| 3q1e | T44 | D | 328 |
| 3q1l | DHL | A | 367 |
| 3q1l | DHL | B | 367 |
| 3q1l | DHL | C | 367 |
| 3q1l | DHL | D | 367 |
| 3q5p | TAC | A | 7101 |
| 3q70 | RIT | A | 2001 |
| 3q81 | IM2 | A | 254 |
| 3q81 | IM2 | B | 254 |
| 3qeo | LLT | A | 261 |
| 3qeo | LLT | B | 261 |
| 3qf1 | PZE | A | 6951 |
| 3qfx | CP6 | A | 602 |
| 3qfx | CP6 | B | 702 |
| 3qg2 | CP6 | A | 609 |
| 3qg2 | CP6 | B | 709 |
| 3qgt | CP6 | A | 609 |
| 3qgt | CP6 | B | 609 |
| 3qgz | ADN | A | 127 |
| 3qip | NVP | A | 561 |
| 3qlg | 1N1 | A | 601 |
| 3qlg | 1N1 | B | 601 |
| 3qoz | 017 | A | 201 |
| 3qt0 | 486 | A | 4 |
| 3quo | FCN | A | 4001 |
| 3qwu | ADN | A | 501 |
| 3qwu | ADN | B | 501 |
| 3r43 | ID8 | A | 332 |
| 3r4x | PZA | A | 597 |
| 3r4x | PZA | A | 598 |
| 3r55 | PZA | A | 597 |
| 3r55 | PZA | A | 598 |
| 3r58 | NPS | A | 332 |
| 3r6i | JMS | A | 332 |
| 3r6w | NFZ | A | 213 |
| 3r6w | NFZ | A | 214 |
| 3r7m | SUZ | A | 332 |
| 3r9c | ECL | A | 451 |
| 3r9c | ECL | A | 452 |
| 3rav | RAV | A | 183 |
| 3rd0 | EDP | A | 175 |
| 3re0 | CPT | B | 201 |
| 3re0 | CPT | B | 202 |
| 3re0 | CPT | C | 201 |
| 3rem | SAL | A | 301 |
| 3rem | SAL | B | 301 |
| 3req | ADN | A | 801 |
| 3ret | SAL | A | 201 |
| 3ret | SAL | B | 201 |
| 3rf4 | FUN | A | 201 |
| 3rf4 | FUN | B | 202 |
| 3rf4 | FUN | C | 203 |
| 3rfm | CFF | A | 330 |
| 3rgf | BAX | A | 465 |
| 3rhw | IVM | A | 348 |
| 3rhw | IVM | B | 348 |
| 3rhw | IVM | B | 349 |
| 3rhw | IVM | D | 348 |
| 3rhw | IVM | D | 349 |
| 3ri5 | IVM | A | 349 |
| 3ri5 | IVM | B | 349 |
| 3ri5 | IVM | C | 350 |
| 3ri5 | IVM | D | 349 |
| 3ri5 | IVM | E | 349 |
| 3ri5 | RI5 | E | 351 |
| 3ria | IVM | A | 348 |
| 3ria | IVM | C | 349 |
| 3ria | IVM | C | 350 |
| 3ria | IVM | E | 348 |
| 3ria | IVM | E | 349 |
| 3rif | IVM | A | 402 |
| 3rif | IVM | A | 403 |
| 3rif | IVM | B | 403 |
| 3rif | IVM | D | 402 |
| 3rif | IVM | E | 402 |
| 3rnj | EDT | A | 1 |
| 3rox | TEP | A | 266 |
| 3rx3 | SUZ | A | 317 |
| 3rze | D7V | A | 1201 |
| 3s20 | CER | A | 1 |
| 3s20 | CER | B | 1 |
| 3s21 | CER | A | 359 |
| 3s23 | CER | A | 359 |
| 3s3g | TLT | A | 317 |
| 3s3v | TOP | A | 187 |
| 3s3v | TOP | A | 193 |
| 3s43 | 478 | A | 401 |
| 3s45 | 478 | B | 201 |
| 3s53 | 017 | A | 201 |
| 3s53 | 017 | B | 203 |
| 3s54 | 017 | B | 201 |
| 3s56 | ROC | A | 201 |
| 3s56 | ROC | B | 203 |
| 3s68 | TCW | A | 227 |
| 3s7s | EXM | A | 601 |
| 3san | ZMR | A | 901 |
| 3san | ZMR | B | 901 |
| 3sdr | 210 | A | 822 |
| 3sdt | AHD | A | 822 |
| 3sdv | 911 | A | 822 |
| 3sfe | TMG | C | 1 |
| 3sfu | RBV | A | 601 |
| 3sfu | RBV | B | 601 |
| 3sfu | RBV | C | 601 |
| 3sg8 | TOY | A | 305 |
| 3sg8 | TOY | B | 305 |
| 3sg9 | KAN | A | 304 |
| 3sg9 | KAN | B | 305 |
| 3sm2 | 478 | A | 126 |
| 3so9 | 017 | A | 100 |
| 3som | DHL | A | 601 |
| 3som | DHL | C | 601 |
| 3som | DHL | D | 601 |
| 3som | DHL | F | 601 |
| 3som | DHL | G | 601 |
| 3som | DHL | H | 601 |
| 3som | DHL | I | 601 |
| 3som | DHL | J | 601 |
| 3som | DHL | K | 601 |
| 3som | DHL | L | 601 |
| 3som | DHL | M | 601 |
| 3som | DHL | N | 601 |
| 3som | DHL | O | 601 |
| 3som | DHL | P | 601 |
| 3spk | TPV | A | 100 |
| 3spk | TPV | B | 100 |
| 3sv6 | SV6 | A | 1 |
| 3sv7 | SV6 | A | 1 |
| 3sv8 | SV6 | A | 1 |
| 3sv9 | SV6 | A | 1 |
| 3sxr | 1N1 | A | 1 |
| 3sxr | 1N1 | B | 2 |
| 3t01 | PPF | A | 503 |
| 3t3c | 017 | A | 201 |
| 3t3q | 9PL | A | 501 |
| 3t3q | 9PL | B | 501 |
| 3t3q | 9PL | C | 501 |
| 3t3q | 9PL | D | 501 |
| 3t3r | 9PL | A | 501 |
| 3t3r | 9PL | B | 501 |
| 3t3r | 9PL | C | 501 |
| 3t3r | 9PL | D | 501 |
| 3t3s | 9PL | A | 1 |
| 3t3s | 9PL | B | 1 |
| 3t3s | 9PL | C | 1 |
| 3t3s | 9PL | D | 1 |
| 3t3s | 9PL | E | 1 |
| 3t3s | 9PL | F | 1 |
| 3t3z | 9PL | A | 501 |
| 3t3z | 9PL | B | 501 |
| 3t3z | 9PL | C | 501 |
| 3t3z | 9PL | D | 501 |
| 3t8n | EDT | D | 135 |
| 3taj | NBO | A | 700 |
| 3tbg | RTZ | A | 1 |
| 3tbg | RTZ | A | 2 |
| 3tbg | RTZ | B | 1 |
| 3tbg | RTZ | B | 2 |
| 3tbg | RTZ | C | 1 |
| 3tbg | RTZ | C | 2 |
| 3tbg | RTZ | D | 1 |
| 3tbg | RTZ | D | 2 |
| 3ti1 | B49 | A | 299 |
| 3ti5 | ZMR | A | 1002 |
| 3ti5 | ZMR | B | 1002 |
| 3tic | ZMR | A | 1002 |
| 3tic | ZMR | B | 1002 |
| 3tic | ZMR | C | 1002 |
| 3tic | ZMR | D | 1002 |
| 3tkd | CYZ | A | 266 |
| 3tkd | CYZ | B | 267 |
| 3tkg | ROC | B | 801 |
| 3tkg | ROC | C | 901 |
| 3tkw | 017 | B | 401 |
| 3tl9 | ROC | A | 401 |
| 3tmz | 06X | A | 503 |
| 3tmz | 06X | A | 504 |
| 3tne | RIT | A | 401 |
| 3tne | RIT | B | 401 |
| 3tq8 | TOP | A | 2001 |
| 3ttp | 017 | A | 201 |
| 3ttr | LQZ | A | 90 |
| 3tuw | PZA | A | 598 |
| 3tvx | PNX | A | 902 |
| 3tvx | PNX | B | 902 |
| 3twp | SAL | A | 404 |
| 3twp | SAL | B | 404 |
| 3twp | SAL | C | 404 |
| 3twp | SAL | D | 404 |
| 3tye | YTZ | B | 902 |
| 3tzf | 08D | B | 280 |
| 3u2c | SUZ | A | 2001 |
| 3u40 | ADN | A | 251 |
| 3u40 | ADN | B | 251 |
| 3u40 | ADN | C | 251 |
| 3u40 | ADN | D | 251 |
| 3u40 | ADN | E | 251 |
| 3u40 | ADN | F | 251 |
| 3u5j | 08H | A | 1 |
| 3u5k | 08J | A | 1 |
| 3u5k | 08J | B | 2 |
| 3u5k | 08J | C | 3 |
| 3u5k | 08J | D | 4 |
| 3u6t | KAN | A | 4699 |
| 3u72 | ISZ | A | 4611 |
| 3u7s | 017 | A | 201 |
| 3u7s | 017 | A | 202 |
| 3u9f | CLM | A | 221 |
| 3u9f | CLM | B | 221 |
| 3u9f | CLM | C | 221 |
| 3u9f | CLM | D | 221 |
| 3u9f | CLM | E | 221 |
| 3u9f | CLM | F | 221 |
| 3u9f | CLM | G | 221 |
| 3u9f | CLM | H | 221 |
| 3u9f | CLM | I | 221 |
| 3u9f | CLM | J | 221 |
| 3u9f | CLM | K | 221 |
| 3u9f | CLM | L | 221 |
| 3u9f | CLM | M | 221 |
| 3u9f | CLM | N | 221 |
| 3u9f | CLM | O | 221 |
| 3u9f | CLM | P | 221 |
| 3u9f | CLM | R | 221 |
| 3u9f | CLM | S | 221 |
| 3ua1 | 08Y | A | 600 |
| 3ua5 | 06X | A | 501 |
| 3ua5 | 06X | A | 502 |
| 3ua5 | 06X | B | 501 |
| 3ua5 | 06X | B | 502 |
| 3uaw | ADN | A | 236 |
| 3uay | ADN | A | 236 |
| 3ub9 | NHY | A | 301 |
| 3ub9 | NHY | B | 301 |
| 3ubo | ADN | A | 353 |
| 3ubo | ADN | B | 353 |
| 3ucb | 017 | A | 201 |
| 3ucb | 017 | B | 202 |
| 3ucj | AZM | A | 229 |
| 3ucj | AZM | B | 229 |
| 3udx | IM2 | A | 998 |
| 3udx | IM2 | B | 999 |
| 3ue0 | AZR | A | 998 |
| 3ue0 | AZR | B | 999 |
| 3uf8 | FK5 | A | 114 |
| 3ufn | ROC | A | 401 |
| 3ufn | ROC | A | 402 |
| 3ug2 | IRE | A | 1 |
| 3ug8 | IMN | A | 2001 |
| 3ugr | IMN | A | 2001 |
| 3uh9 | FCN | A | 151 |
| 3uh9 | FCN | B | 151 |
| 3uiv | 308 | H | 1008 |
| 3um5 | CP6 | A | 609 |
| 3um5 | CP6 | B | 709 |
| 3una | SAL | A | 1340 |
| 3una | SAL | B | 1340 |
| 3unc | SAL | A | 1338 |
| 3unc | SAL | B | 1338 |
| 3uni | SAL | A | 1344 |
| 3uni | SAL | B | 1345 |
| 3upn | IM2 | A | 800 |
| 3upn | IM2 | B | 800 |
| 3upr | 1KX | A | 277 |
| 3upr | 1KX | C | 277 |
| 3uq6 | ADN | A | 401 |
| 3uq6 | ADN | B | 401 |
| 3uqa | FK5 | A | 114 |
| 3uqb | FK5 | A | 114 |
| 3ur0 | SVR | B | 516 |
| 3ur0 | SVR | C | 516 |
| 3ur0 | SVR | C | 517 |
| 3ut5 | LOC | B | 502 |
| 3ut5 | LOC | D | 502 |
| 3uud | EST | A | 600 |
| 3uud | EST | B | 600 |
| 3uvv | T3 | A | 501 |
| 3v35 | NTI | A | 317 |
| 3v5w | 8PR | A | 701 |
| 3vaw | FK5 | A | 114 |
| 3vet | TOY | A | 604 |
| 3vhu | SNL | A | 1001 |
| 3vkx | T3 | A | 301 |
| 3vn2 | TLS | A | 501 |
| 3vri | 1KX | A | 301 |
| 3vrj | 1KX | A | 301 |
| 3znc | BZ1 | A | 500 |
| 3ztv | ADN | A | 1600 |
| 4a79 | P1B | A | 601 |
| 4a79 | P1B | B | 601 |
| 4a7b | HAE | A | 1273 |
| 4a7b | HAE | B | 1270 |
| 4a97 | ZPC | A | 1318 |
| 4a97 | ZPC | B | 1318 |
| 4a97 | ZPC | C | 1318 |
| 4a97 | ZPC | D | 1318 |
| 4a97 | ZPC | E | 1318 |
| 4a97 | ZPC | F | 1318 |
| 4a97 | ZPC | G | 1318 |
| 4a97 | ZPC | H | 1318 |
| 4a97 | ZPC | I | 1318 |
| 4a97 | ZPC | J | 1318 |
| 4a9j | TYL | A | 1188 |
| 4a9j | TYL | B | 1187 |
| 4a9j | TYL | C | 1184 |
| 4a9k | TYL | A | 2200 |
| 4a9k | TYL | B | 2198 |
| 4af0 | MOA | A | 1526 |
| 4af0 | MOA | B | 1526 |
| 4afg | QMR | A | 1214 |
| 4afg | QMR | B | 1214 |
| 4afg | QMR | C | 1214 |
| 4afg | QMR | D | 1214 |
| 4afg | QMR | E | 1214 |
| 4aft | QMR | A | 301 |
| 4aft | QMR | B | 301 |
| 4aft | QMR | C | 301 |
| 4aft | QMR | D | 301 |
| 4aft | QMR | E | 301 |
| 4agd | B49 | A | 2000 |
| 4amj | CVD | A | 1359 |
| 4amj | CVD | B | 1360 |
| 4asd | BAX | A | 1500 |
| 4b7n | ZMR | A | 601 |
| 4b7q | ZMR | A | 601 |
| 4b7q | ZMR | B | 601 |
| 4b7q | ZMR | C | 601 |
| 4b7q | ZMR | D | 601 |
| 4cla | CLM | A | 221 |
| 4cox | IMN | A | 701 |
| 4cox | IMN | B | 701 |
| 4cox | IMN | C | 701 |
| 4cox | IMN | D | 701 |
| 4d9h | ADN | A | 301 |
| 4da6 | GA2 | A | 301 |
| 4da7 | AC2 | A | 301 |
| 4daj | 0HK | A | 2000 |
| 4daj | 0HK | B | 2000 |
| 4daj | 0HK | C | 2000 |
| 4daj | 0HK | D | 2000 |
| 4dd4 | CPT | A | 201 |
| 4dd4 | CPT | A | 202 |
| 4dd6 | CPT | A | 211 |
| 4dd6 | CPT | A | 212 |
| 4dd7 | QPT | A | 201 |
| 4dd7 | QPT | A | 202 |
| 4dd9 | QPT | A | 201 |
| 4dd9 | QPT | A | 202 |
| 4ddb | CPT | A | 201 |
| 4ddb | CPT | A | 202 |
| 4ddc | CPT | A | 201 |
| 4ddc | CPT | A | 202 |
| 4ddc | CPT | B | 201 |
| 4ddc | CPT | B | 202 |
| 4dfb | KAN | A | 401 |
| 4dfb | KAN | B | 401 |
| 4dfu | KAN | A | 401 |
| 4dfu | KAN | B | 402 |
| 4dl5 | CPT | T | 101 |
| 4dqb | 017 | B | 101 |
| 4dqc | 017 | A | 101 |
| 4dqe | 017 | B | 101 |
| 4dqf | 017 | B | 101 |
| 4dqh | 017 | B | 101 |
| 4dt8 | ADN | A | 401 |
| 4dt8 | ADN | B | 401 |
| 4dta | ADN | A | 401 |
| 4dta | ADN | B | 401 |
| 4dtz | LDP | A | 501 |
| 4dtz | LDP | B | 501 |
| 4du2 | LDP | A | 501 |
| 4du2 | LDP | B | 501 |
| 4dub | LDP | A | 501 |
| 4dub | LDP | B | 501 |
| 4dx5 | MIY | B | 1103 |
| 4dx7 | DM2 | A | 1105 |
| 4dx7 | DM2 | A | 1106 |
| 4dx7 | DM2 | B | 1104 |
| 4dxu | ACA | A | 711 |
| 4dz2 | FK5 | A | 200 |
| 4dz2 | FK5 | B | 200 |
| 4dz3 | FK5 | A | 201 |
| 4dz3 | FK5 | B | 201 |
| 4e3a | ADN | A | 500 |
| 4e3a | ADN | B | 500 |
| 4e8j | LN0 | A | 202 |
| 4e8j | LN0 | B | 202 |
| 4eb4 | D16 | A | 402 |
| 4eb4 | D16 | B | 402 |
| 4eb4 | D16 | C | 402 |
| 4eb4 | D16 | D | 402 |
| 4eb6 | VLB | C | 503 |
| 4ebk | TOY | A | 301 |
| 4ebk | TOY | B | 302 |
| 4eey | CPT | T | 101 |
| 4eix | IMN | A | 202 |
| 4ejg | NCT | A | 501 |
| 4ejg | NCT | B | 501 |
| 4ejg | NCT | C | 501 |
| 4ejg | NCT | D | 501 |
| 4ejj | NCT | A | 501 |
| 4ejj | NCT | B | 501 |
| 4ejj | NCT | C | 501 |
| 4ejj | NCT | D | 501 |
| 4enh | FVX | A | 602 |
| 4eoh | TEP | A | 402 |
| 4eoh | TEP | B | 402 |
| 4eq4 | SAL | A | 601 |
| 4eq4 | SAL | B | 601 |
| 4eql | SAL | A | 602 |
| 4eql | SAL | B | 602 |
| 4evy | TOY | A | 201 |
| 4evy | TOY | B | 201 |
| 4ey6 | GNT | A | 604 |
| 4ey6 | GNT | B | 605 |
| 4ey7 | E20 | A | 604 |
| 4ey7 | E20 | B | 605 |
| 4eyb | 0WO | A | 303 |
| 4eyb | 0WO | B | 301 |
| 4eyl | 0RV | A | 301 |
| 4eyl | 0RV | B | 301 |
| 4f8h | RKE | A | 401 |
| 4f8h | RKE | B | 401 |
| 4f8h | RKE | C | 401 |
| 4f8h | RKE | D | 401 |
| 4f8h | RKE | E | 401 |
| 4f92 | SAN | B | 2201 |
| 4f93 | SAN | B | 3004 |
| 4fe1 | PQN | A | 846 |
| 4fe1 | PQN | B | 840 |
| 4feu | KAN | A | 301 |
| 4feu | KAN | B | 301 |
| 4feu | KAN | C | 301 |
| 4feu | KAN | D | 301 |
| 4feu | KAN | E | 301 |
| 4feu | KAN | F | 301 |
| 4fev | KAN | A | 301 |
| 4fev | KAN | B | 301 |
| 4fev | KAN | C | 301 |
| 4fev | KAN | D | 301 |
| 4fev | KAN | E | 301 |
| 4fev | KAN | F | 301 |
| 4few | KAN | A | 301 |
| 4few | KAN | B | 301 |
| 4few | KAN | C | 301 |
| 4few | KAN | D | 301 |
| 4few | KAN | E | 301 |
| 4few | KAN | F | 301 |
| 4fex | KAN | A | 301 |
| 4fex | KAN | B | 301 |
| 4fex | KAN | C | 301 |
| 4fex | KAN | D | 301 |
| 4fex | KAN | E | 301 |
| 4fgz | CQA | A | 301 |
| 4fgz | CQA | A | 302 |
| 4fgz | CQA | B | 301 |
| 4fgz | CQA | B | 302 |
| 4fim | CEL | A | 711 |
| 4fjp | NPS | A | 711 |
| 4fo4 | MOA | A | 502 |
| 4fo4 | MOA | B | 502 |
| 4for | FLP | A | 711 |
| 4fr8 | TNG | A | 601 |
| 4fxs | MOA | A | 702 |
| 4g24 | ACA | A | 1004 |
| 4g2z | ID8 | A | 711 |
| 4g49 | CPT | A | 205 |
| 4g49 | CPT | A | 206 |
| 4g4a | CPT | A | 204 |
| 4g4a | CPT | A | 205 |
| 4g4b | CPT | A | 201 |
| 4g4b | CPT | A | 203 |
| 4g4c | QPT | A | 202 |
| 4g4c | QPT | A | 203 |
| 4g4h | QPT | A | 205 |
| 4g4h | QPT | A | 206 |
| 4gkh | KAN | A | 301 |
| 4gkh | KAN | B | 301 |
| 4gkh | KAN | C | 301 |
| 4gkh | KAN | D | 301 |
| 4gkh | KAN | E | 301 |
| 4gkh | KAN | F | 301 |
| 4gkh | KAN | G | 301 |
| 4gkh | KAN | H | 301 |
| 4gkh | KAN | I | 301 |
| 4gkh | KAN | J | 301 |
| 4gkh | KAN | K | 301 |
| 4gkh | KAN | L | 301 |
| 4gki | KAN | A | 301 |
| 4gki | KAN | B | 301 |
| 4gki | KAN | C | 301 |
| 4gki | KAN | D | 301 |
| 4gki | KAN | E | 301 |
| 4gki | KAN | F | 301 |
| 4gki | KAN | G | 301 |
| 4gki | KAN | H | 301 |
| 4gki | KAN | I | 301 |
| 4gki | KAN | J | 301 |
| 4gki | KAN | K | 301 |
| 4gki | KAN | L | 301 |
| 4gn6 | TYL | A | 621 |
| 4grk | KTR | A | 713 |
| 4gtb | D16 | A | 303 |
| 4h9m | HAE | A | 929 |
| 4pah | LNR | A | 600 |
| 4ubp | HAE | C | 800 |
| 5apr | DHL | I | 9 |
| 5pah | LDP | A | 600 |
