# Supplementary material for: SInCRe—structural interactome computational resource for Mycobacterium tuberculosis
Source: Database (Oxford). 2015 Jun 30;2015:bav060. doi: 10.1093/database/bav060 (PMC4485431; doi:10.1093/database/bav060)
Supplement: Supplementary Data [file supp_bav060_suppl_data.zip › SInCRe_supplementary_Table1.docx]

Supplementary Table 1: List of 132 FDA-approved drugs identified against 56 potential Mtb proteins

| RV ID | DrugBank ID | Drug Name |
| --- | --- | --- |
| Rv0005 | DB01051 | Novobiocin |
| Rv0006 | DB00218 | Moxifloxacin |
| Rv0006 | DB00467 | Enoxacin |
| Rv0006 | DB00487 | Pefloxacin |
| Rv0006 | DB00537 | Ciprofloxacin |
| Rv0006 | DB00685 | Trovafloxacin |
| Rv0006 | DB00817 | Rosoxacin |
| Rv0006 | DB00978 | Lomefloxacin |
| Rv0006 | DB01044 | Gatifloxacin |
| Rv0006 | DB01059 | Norfloxacin |
| Rv0006 | DB01137 | Levofloxacin |
| Rv0006 | DB01155 | Gemifloxacin |
| Rv0006 | DB01165 | Ofloxacin |
| Rv0006 | DB01208 | Sparfloxacin |
| Rv0006 | DB04576 | Fleroxacin |
| Rv0006 | DB06771 | Besifloxacin |
| Rv0016c | DB00274 | Cefmetazole |
| Rv0016c | DB00303 | Ertapenem |
| Rv0016c | DB00430 | Cefpiramide |
| Rv0016c | DB00438 | Ceftazidime |
| Rv0016c | DB00535 | Cefdinir |
| Rv0016c | DB00671 | Cefixime |
| Rv0016c | DB00923 | Ceforanide |
| Rv0016c | DB00948 | Mezlocillin |
| Rv0016c | DB01000 | Cyclacillin |
| Rv0016c | DB01053 | Penicillin G |
| Rv0016c | DB01147 | Cloxacillin |
| Rv0016c | DB01163 | Amdinocillin |
| Rv0016c | DB01326 | Cefamandole |
| Rv0016c | DB01327 | Cefazolin |
| Rv0016c | DB01328 | Cefonicid |
| Rv0016c | DB01329 | Cefoperazone |
| Rv0016c | DB01413 | Cefepime |
| Rv0016c | DB01415 | Ceftibuten |
| Rv0016c | DB01598 | Imipenem |
| Rv0016c | DB01607 | Ticarcillin |
| Rv0050 | DB00229 | Cefotiam |
| Rv0050 | DB00267 | Cefmenoxime |
| Rv0050 | DB00274 | Cefmetazole |
| Rv0050 | DB00301 | Flucloxacillin |
| Rv0050 | DB00303 | Ertapenem |
| Rv0050 | DB00319 | Piperacillin |
| Rv0050 | DB00415 | Ampicillin |
| Rv0050 | DB00417 | Penicillin V |
| Rv0050 | DB00430 | Cefpiramide |
| Rv0050 | DB00438 | Ceftazidime |
| Rv0050 | DB00447 | Loracarbef |
| Rv0050 | DB00456 | Cefalotin |
| Rv0050 | DB00485 | Dicloxacillin |
| Rv0050 | DB00493 | Cefotaxime |
| Rv0050 | DB00567 | Cephalexin |
| Rv0050 | DB00607 | Nafcillin |
| Rv0050 | DB00671 | Cefixime |
| Rv0050 | DB00713 | Oxacillin |
| Rv0050 | DB00739 | Hetacillin |
| Rv0050 | DB00833 | Cefaclor |
| Rv0050 | DB00923 | Ceforanide |
| Rv0050 | DB00948 | Mezlocillin |
| Rv0050 | DB01000 | Cyclacillin |
| Rv0050 | DB01060 | Amoxicillin |
| Rv0050 | DB01061 | Azlocillin |
| Rv0050 | DB01066 | Cefditoren |
| Rv0050 | DB01112 | Cefuroxime |
| Rv0050 | DB01139 | Cephapirin |
| Rv0050 | DB01140 | Cefadroxil |
| Rv0050 | DB01147 | Cloxacillin |
| Rv0050 | DB01150 | Cefprozil |
| Rv0050 | DB01163 | Amdinocillin |
| Rv0050 | DB01326 | Cefamandole |
| Rv0050 | DB01327 | Cefazolin |
| Rv0050 | DB01328 | Cefonicid |
| Rv0050 | DB01329 | Cefoperazone |
| Rv0050 | DB01331 | Cefoxitin |
| Rv0050 | DB01332 | Ceftizoxime |
| Rv0050 | DB01413 | Cefepime |
| Rv0050 | DB01414 | Cefacetrile |
| Rv0050 | DB01415 | Ceftibuten |
| Rv0050 | DB01598 | Imipenem |
| Rv0050 | DB01602 | Bacampicillin |
| Rv0050 | DB01603 | Meticillin |
| Rv0050 | DB01604 | Pivampicillin |
| Rv0050 | DB01605 | Pivmecillinam |
| Rv0050 | DB01607 | Ticarcillin |
| Rv0050 | DB08795 | Azidocillin |
| Rv0118c | DB00336 | Nitrofurazone |
| Rv0120c | DB02703 | Fusidic Acid |
| Rv0136 | DB00196 | Fluconazole |
| Rv0136 | DB00251 | Terconazole |
| Rv0136 | DB00582 | Voriconazole |
| Rv0136 | DB01026 | Ketoconazole |
| Rv0136 | DB01153 | Sertaconazole |
| Rv0136 | DB01263 | Posaconazole |
| Rv0136 | DB04794 | Bifonazole |
| Rv0248c | DB00730 | Thiabendazole |
| Rv0306 | DB00698 | Nitrofurantoin |
| Rv0327c | DB00196 | Fluconazole |
| Rv0327c | DB00251 | Terconazole |
| Rv0327c | DB00582 | Voriconazole |
| Rv0327c | DB01026 | Ketoconazole |
| Rv0327c | DB01153 | Sertaconazole |
| Rv0327c | DB01263 | Posaconazole |
| Rv0327c | DB04794 | Bifonazole |
| Rv0399c | DB00456 | Cefalotin |
| Rv0568 | DB00196 | Fluconazole |
| Rv0568 | DB00251 | Terconazole |
| Rv0568 | DB00582 | Voriconazole |
| Rv0568 | DB01026 | Ketoconazole |
| Rv0568 | DB01153 | Sertaconazole |
| Rv0568 | DB01263 | Posaconazole |
| Rv0568 | DB04794 | Bifonazole |
| Rv0651 | DB00778 | Roxithromycin |
| Rv0651 | DB01190 | Clindamycin |
| Rv0651 | DB01211 | Clarithromycin |
| Rv0651 | DB01369 | Quinupristin |
| Rv0651 | DB01627 | Lincomycin |
| Rv0682 | DB00452 | Framycetin |
| Rv0682 | DB00479 | Amikacin |
| Rv0682 | DB00560 | Tigecycline |
| Rv0682 | DB00684 | Tobramycin |
| Rv0682 | DB00798 | Gentamicin |
| Rv0682 | DB00919 | Spectinomycin |
| Rv0682 | DB00955 | Netilmicin |
| Rv0682 | DB00994 | Neomycin |
| Rv0682 | DB01082 | Streptomycin |
| Rv0682 | DB01172 | Kanamycin |
| Rv0682 | DB06696 | Arbekacin |
| Rv0683 | DB00759 | Tetracycline |
| Rv0684 | DB02703 | Fusidic Acid |
| Rv0700 | DB00698 | Nitrofurantoin |
| Rv0700 | DB01421 | Paromomycin |
| Rv0701 | DB01256 | Retapamulin |
| Rv0702 | DB00199 | Erythromycin |
| Rv0702 | DB00207 | Azithromycin |
| Rv0702 | DB01321 | Josamycin |
| Rv0702 | DB01361 | Troleandomycin |
| Rv0705 | DB00560 | Tigecycline |
| Rv0705 | DB00759 | Tetracycline |
| Rv0706 | DB00199 | Erythromycin |
| Rv0706 | DB00207 | Azithromycin |
| Rv0706 | DB01369 | Quinupristin |
| Rv0707 | DB00759 | Tetracycline |
| Rv0708 | DB00446 | Chloramphenicol |
| Rv0718 | DB00759 | Tetracycline |
| Rv0764c | DB00196 | Fluconazole |
| Rv0764c | DB00251 | Terconazole |
| Rv0764c | DB00582 | Voriconazole |
| Rv0764c | DB01026 | Ketoconazole |
| Rv0764c | DB01153 | Sertaconazole |
| Rv0764c | DB01263 | Posaconazole |
| Rv0764c | DB04794 | Bifonazole |
| Rv0907 | DB00456 | Cefalotin |
| Rv1207 | DB00250 | Dapsone |
| Rv1207 | DB00259 | Sulfanilamide |
| Rv1207 | DB00263 | Sulfisoxazole |
| Rv1207 | DB00576 | Sulfamethizole |
| Rv1207 | DB00634 | Sulfacetamide |
| Rv1207 | DB01015 | Sulfamethoxazole |
| Rv1207 | DB01298 | Sulfacytine |
| Rv1207 | DB01581 | Sulfamerazine |
| Rv1207 | DB01582 | Sulfamethazine |
| Rv1207 | DB06729 | Sulfaphenazole |
| Rv1240 | DB00336 | Nitrofurazone |
| Rv1315 | DB00828 | Fosfomycin |
| Rv1526c | DB00512 | Vancomycin |
| Rv1536 | DB00410 | Mupirocin |
| Rv1552 | DB00730 | Thiabendazole |
| Rv1595 | DB00730 | Thiabendazole |
| Rv1666c | DB00196 | Fluconazole |
| Rv1666c | DB00251 | Terconazole |
| Rv1666c | DB00582 | Voriconazole |
| Rv1666c | DB01026 | Ketoconazole |
| Rv1666c | DB01153 | Sertaconazole |
| Rv1666c | DB01263 | Posaconazole |
| Rv1666c | DB04794 | Bifonazole |
| Rv1730c | DB00355 | Aztreonam |
| Rv1730c | DB00456 | Cefalotin |
| Rv1730c | DB01147 | Cloxacillin |
| Rv1820 | DB00336 | Nitrofurazone |
| Rv1850 | DB00551 | Acetohydroxamic Acid |
| Rv1922 | DB00355 | Aztreonam |
| Rv1922 | DB00456 | Cefalotin |
| Rv1922 | DB01147 | Cloxacillin |
| Rv2056c | DB00560 | Tigecycline |
| Rv2056c | DB00759 | Tetracycline |
| Rv2068c | DB00766 | Clavulanate |
| Rv2068c | DB01598 | Imipenem |
| Rv2068c | DB01606 | Tazobactam |
| Rv2163c | DB00267 | Cefmenoxime |
| Rv2163c | DB00274 | Cefmetazole |
| Rv2163c | DB00303 | Ertapenem |
| Rv2163c | DB00319 | Piperacillin |
| Rv2163c | DB00355 | Aztreonam |
| Rv2163c | DB00415 | Ampicillin |
| Rv2163c | DB00417 | Penicillin V |
| Rv2163c | DB00430 | Cefpiramide |
| Rv2163c | DB00438 | Ceftazidime |
| Rv2163c | DB00456 | Cefalotin |
| Rv2163c | DB00485 | Dicloxacillin |
| Rv2163c | DB00493 | Cefotaxime |
| Rv2163c | DB00535 | Cefdinir |
| Rv2163c | DB00567 | Cephalexin |
| Rv2163c | DB00607 | Nafcillin |
| Rv2163c | DB00671 | Cefixime |
| Rv2163c | DB00713 | Oxacillin |
| Rv2163c | DB00739 | Hetacillin |
| Rv2163c | DB00923 | Ceforanide |
| Rv2163c | DB00948 | Mezlocillin |
| Rv2163c | DB01000 | Cyclacillin |
| Rv2163c | DB01053 | Penicillin G |
| Rv2163c | DB01066 | Cefditoren |
| Rv2163c | DB01140 | Cefadroxil |
| Rv2163c | DB01147 | Cloxacillin |
| Rv2163c | DB01150 | Cefprozil |
| Rv2163c | DB01163 | Amdinocillin |
| Rv2163c | DB01212 | Ceftriaxone |
| Rv2163c | DB01326 | Cefamandole |
| Rv2163c | DB01327 | Cefazolin |
| Rv2163c | DB01328 | Cefonicid |
| Rv2163c | DB01329 | Cefoperazone |
| Rv2163c | DB01331 | Cefoxitin |
| Rv2163c | DB01332 | Ceftizoxime |
| Rv2163c | DB01413 | Cefepime |
| Rv2163c | DB01415 | Ceftibuten |
| Rv2163c | DB01416 | Cefpodoxime |
| Rv2163c | DB01598 | Imipenem |
| Rv2163c | DB01603 | Meticillin |
| Rv2163c | DB01607 | Ticarcillin |
| Rv2163c | DB08795 | Azidocillin |
| Rv2864c | DB00274 | Cefmetazole |
| Rv2864c | DB00303 | Ertapenem |
| Rv2864c | DB00319 | Piperacillin |
| Rv2864c | DB00415 | Ampicillin |
| Rv2864c | DB00430 | Cefpiramide |
| Rv2864c | DB00438 | Ceftazidime |
| Rv2864c | DB00456 | Cefalotin |
| Rv2864c | DB00485 | Dicloxacillin |
| Rv2864c | DB00493 | Cefotaxime |
| Rv2864c | DB00567 | Cephalexin |
| Rv2864c | DB00607 | Nafcillin |
| Rv2864c | DB00713 | Oxacillin |
| Rv2864c | DB00739 | Hetacillin |
| Rv2864c | DB00923 | Ceforanide |
| Rv2864c | DB00948 | Mezlocillin |
| Rv2864c | DB01000 | Cyclacillin |
| Rv2864c | DB01066 | Cefditoren |
| Rv2864c | DB01140 | Cefadroxil |
| Rv2864c | DB01147 | Cloxacillin |
| Rv2864c | DB01150 | Cefprozil |
| Rv2864c | DB01163 | Amdinocillin |
| Rv2864c | DB01212 | Ceftriaxone |
| Rv2864c | DB01326 | Cefamandole |
| Rv2864c | DB01327 | Cefazolin |
| Rv2864c | DB01328 | Cefonicid |
| Rv2864c | DB01329 | Cefoperazone |
| Rv2864c | DB01331 | Cefoxitin |
| Rv2864c | DB01413 | Cefepime |
| Rv2864c | DB01415 | Ceftibuten |
| Rv2864c | DB01598 | Imipenem |
| Rv2864c | DB01603 | Meticillin |
| Rv2864c | DB01607 | Ticarcillin |
| Rv2864c | DB08795 | Azidocillin |
| Rv2911 | DB00274 | Cefmetazole |
| Rv2911 | DB00303 | Ertapenem |
| Rv2911 | DB00319 | Piperacillin |
| Rv2911 | DB00415 | Ampicillin |
| Rv2911 | DB00430 | Cefpiramide |
| Rv2911 | DB00438 | Ceftazidime |
| Rv2911 | DB00447 | Loracarbef |
| Rv2911 | DB00456 | Cefalotin |
| Rv2911 | DB00485 | Dicloxacillin |
| Rv2911 | DB00567 | Cephalexin |
| Rv2911 | DB00607 | Nafcillin |
| Rv2911 | DB00713 | Oxacillin |
| Rv2911 | DB00739 | Hetacillin |
| Rv2911 | DB00766 | Clavulanate |
| Rv2911 | DB00833 | Cefaclor |
| Rv2911 | DB00948 | Mezlocillin |
| Rv2911 | DB01000 | Cyclacillin |
| Rv2911 | DB01140 | Cefadroxil |
| Rv2911 | DB01147 | Cloxacillin |
| Rv2911 | DB01163 | Amdinocillin |
| Rv2911 | DB01329 | Cefoperazone |
| Rv2911 | DB01330 | Cefotetan |
| Rv2911 | DB01331 | Cefoxitin |
| Rv2911 | DB01598 | Imipenem |
| Rv2911 | DB01603 | Meticillin |
| Rv2911 | DB01606 | Tazobactam |
| Rv2911 | DB08795 | Azidocillin |
| Rv3003c | DB00336 | Nitrofurazone |
| Rv3034c | DB01764 | Dalfopristin |
| Rv3059 | DB00196 | Fluconazole |
| Rv3059 | DB00251 | Terconazole |
| Rv3059 | DB00582 | Voriconazole |
| Rv3059 | DB01026 | Ketoconazole |
| Rv3059 | DB01153 | Sertaconazole |
| Rv3059 | DB01263 | Posaconazole |
| Rv3059 | DB04794 | Bifonazole |
| Rv3318 | DB00730 | Thiabendazole |
| Rv3330 | DB00274 | Cefmetazole |
| Rv3330 | DB00303 | Ertapenem |
| Rv3330 | DB00319 | Piperacillin |
| Rv3330 | DB00415 | Ampicillin |
| Rv3330 | DB00430 | Cefpiramide |
| Rv3330 | DB00438 | Ceftazidime |
| Rv3330 | DB00447 | Loracarbef |
| Rv3330 | DB00456 | Cefalotin |
| Rv3330 | DB00485 | Dicloxacillin |
| Rv3330 | DB00567 | Cephalexin |
| Rv3330 | DB00607 | Nafcillin |
| Rv3330 | DB00713 | Oxacillin |
| Rv3330 | DB00739 | Hetacillin |
| Rv3330 | DB00833 | Cefaclor |
| Rv3330 | DB00948 | Mezlocillin |
| Rv3330 | DB01000 | Cyclacillin |
| Rv3330 | DB01140 | Cefadroxil |
| Rv3330 | DB01147 | Cloxacillin |
| Rv3330 | DB01163 | Amdinocillin |
| Rv3330 | DB01329 | Cefoperazone |
| Rv3330 | DB01330 | Cefotetan |
| Rv3330 | DB01331 | Cefoxitin |
| Rv3330 | DB01603 | Meticillin |
| Rv3330 | DB08795 | Azidocillin |
| Rv3442c | DB00254 | Doxycycline |
| Rv3442c | DB00256 | Lymecycline |
| Rv3442c | DB00453 | Clomocycline |
| Rv3442c | DB00560 | Tigecycline |
| Rv3442c | DB00595 | Oxytetracycline |
| Rv3442c | DB00618 | Demeclocycline |
| Rv3442c | DB01017 | Minocycline |
| Rv3442c | DB01301 | Rolitetracycline |
| Rv3458c | DB00254 | Doxycycline |
| Rv3458c | DB00256 | Lymecycline |
| Rv3458c | DB00453 | Clomocycline |
| Rv3458c | DB00595 | Oxytetracycline |
| Rv3458c | DB00618 | Demeclocycline |
| Rv3458c | DB01017 | Minocycline |
| Rv3460c | DB00560 | Tigecycline |
| Rv3470c | DB00336 | Nitrofurazone |
| Rv3608c | DB00250 | Dapsone |
| Rv3608c | DB00259 | Sulfanilamide |
| Rv3608c | DB00263 | Sulfisoxazole |
| Rv3608c | DB00576 | Sulfamethizole |
| Rv3608c | DB00634 | Sulfacetamide |
| Rv3608c | DB01015 | Sulfamethoxazole |
| Rv3608c | DB01298 | Sulfacytine |
| Rv3608c | DB01581 | Sulfamerazine |
| Rv3608c | DB01582 | Sulfamethazine |
| Rv3608c | DB06729 | Sulfaphenazole |
| Rv3682 | DB00229 | Cefotiam |
| Rv3682 | DB00267 | Cefmenoxime |
| Rv3682 | DB00274 | Cefmetazole |
| Rv3682 | DB00301 | Flucloxacillin |
| Rv3682 | DB00319 | Piperacillin |
| Rv3682 | DB00415 | Ampicillin |
| Rv3682 | DB00417 | Penicillin V |
| Rv3682 | DB00430 | Cefpiramide |
| Rv3682 | DB00438 | Ceftazidime |
| Rv3682 | DB00447 | Loracarbef |
| Rv3682 | DB00456 | Cefalotin |
| Rv3682 | DB00485 | Dicloxacillin |
| Rv3682 | DB00493 | Cefotaxime |
| Rv3682 | DB00567 | Cephalexin |
| Rv3682 | DB00607 | Nafcillin |
| Rv3682 | DB00713 | Oxacillin |
| Rv3682 | DB00739 | Hetacillin |
| Rv3682 | DB00833 | Cefaclor |
| Rv3682 | DB00948 | Mezlocillin |
| Rv3682 | DB01000 | Cyclacillin |
| Rv3682 | DB01060 | Amoxicillin |
| Rv3682 | DB01061 | Azlocillin |
| Rv3682 | DB01066 | Cefditoren |
| Rv3682 | DB01112 | Cefuroxime |
| Rv3682 | DB01139 | Cephapirin |
| Rv3682 | DB01140 | Cefadroxil |
| Rv3682 | DB01147 | Cloxacillin |
| Rv3682 | DB01150 | Cefprozil |
| Rv3682 | DB01163 | Amdinocillin |
| Rv3682 | DB01327 | Cefazolin |
| Rv3682 | DB01329 | Cefoperazone |
| Rv3682 | DB01331 | Cefoxitin |
| Rv3682 | DB01602 | Bacampicillin |
| Rv3682 | DB01603 | Meticillin |
| Rv3682 | DB01604 | Pivampicillin |
| Rv3682 | DB01605 | Pivmecillinam |
| Rv3682 | DB01607 | Ticarcillin |
| Rv3682 | DB08795 | Azidocillin |
